# Tetrazolylidene-Stabilized Gold(I) Complexes: Synthesis and Evaluation of Anticancer Activity *In Vitro*

**DOI:** 10.1021/acs.inorgchem.6c00049

**Published:** 2026-04-15

**Authors:** Simon Stifel, Claudia Schmidt, Leon F. Richter, Alexander Pöthig, Angela Casini, Fritz E. Kühn

**Affiliations:** † 163254 Technical University of Munich, School of Natural Sciences, Department of Chemistry, Molecular Catalysis, Lichtenbergstr. 4, 85748 Garching bei München, Germany; ‡ 9184 Technical University of Munich, School of Natural Sciences, Department of Chemistry, Chair of Medicinal and Bioinorganic Chemistry, Lichtenbergstr. 4, 85748 Garching bei München, Germany; § Technical University of Munich, School of Natural Sciences, Catalysis Research Center (CRC), Ernst-Otto-Fischer Str. 1, 85748 Garching bei München, Germany

## Abstract

The first comprehensive study of a series of seven mesoionic tetrazolylidene gold­(I) chloride complexes (**1**–**7**) featuring a range of alkyl and aryl substituents (Me, *t*-Bu, iPr, Ph, Tol, Dipp, Mes) is reported. Three synthetic pathways enabling access to scarcely explored *abnormal* 1,3-disubstituted tetrazolium ligand precursors (**L**_**1**_–**L**_**7**_) have been established. All complexes are characterized by NMR spectroscopy, mass spectrometry, and elemental analysis, confirming their composition and purity. Single-crystal X-ray crystallography of six gold­(I) complexes (**1**–**6**) reveals nearly linear coordination (176.49(11)–179.0(2)°) at the gold­(I) center and a distinct geometric arrangement across the series. NMR stability studies with model nucleophiles *L-*cysteine (Cys) and glutathione (GSH) support the structural findings, demonstrating rapid and complete reaction of complexes **1**–**7** with thiols, as confirmed by ^1^H NMR and ESI-MS. The antiproliferative activity of the obtained complexes (**1**–**7**) and selected precursors (**L**_**2**_, **L**_**3**_, **L**_**5**_, **L**_**7**_) has been evaluated using MTT assays against human A2780 (ovarian) and A549 (lung) cancer cell lines, alongside noncancerous VERO E6 kidney cells for comparison. Most of the complexes display high selectivity indices (SI^A2780^ = 63.2–86.7) and potent antiproliferative effects in the low submicromolar range against A2780, outperforming cisplatin and matching the activity of auranofin. Overall, the results presented here demonstrate the potential of gold­(I) tetrazolylidene-based complexes for medicinal applications.

## Graphical abstract

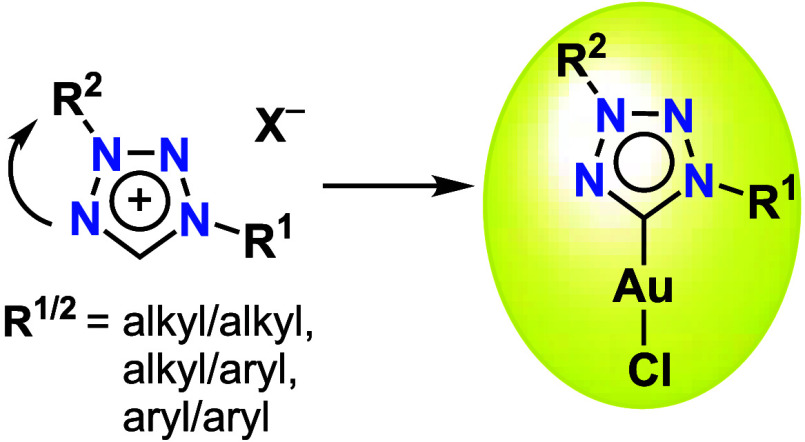

## Introduction

In recent years, gold­(I) *N* **-**heterocyclic carbene (NHC) complexes have attracted considerable attention due to their potent antiproliferative properties.^1–6^ While imidazole**-**based carbene complexes have long dominated gold­(I) chemistry, other azole**-**derived systems such as triazole**-**based NHCs are only beginning to gain attention for their promising characteristics.^7–10^ In particular, tetrazole**-**based systems remain largely underrepresented, despite their proven utility in coordination chemistry, materials science, and medicinal chemistry.^11–14^ Tetrazoles are chemically intriguing five**-**membered aromatic heterocycles, notable due to their high nitrogen content, low basicity, and relatively pronounced acidity (p*K*_a_ ∼ 5).^15,16^ Based on these unique features, tetrazoles are frequently employed as carboxylic acid bioisosteres, offering improved metabolic stability and lipophilicity.^17–19^ Several examples can be found in purely organic FDA**-**approved drugs (Losartan, Valsartan, BMS**-**183920), wherein tetrazoles are under investigation for diverse applications, including antihypertensive, antibiotic, peptidase**-**inhibiting, and anticancer effects.^15,20–25^ Although their coordination chemistry was first investigated over half a century ago, synthetic challenges—such as low stability and limited derivatization options—have hindered their broader application in organometallic chemistry.^26–28^ Thus, only few synthetic and theoretical studies have been conducted to date on transition metal complexes of this ligand family.^27,29–32^ While tetrazole**-**based complexes of some transition metals such as Fe, Rh, Co, Zn, and Pd were reported decades ago,^26,33–36^ the first gold­(I) tetrazolylidene complexes were only recently described by Raubenheimer and co**-**workers.^27,30^

Figure 1 provides an overview of previously reported tetrazolylidene-based gold­(I) complexes, along with those investigated in this work.

**Figure 1:**
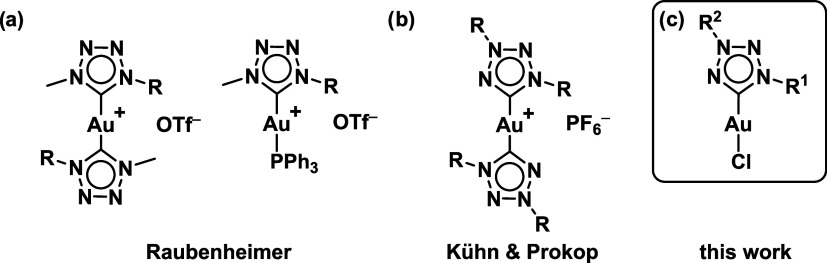
Structures of literature known Au­(I) tetrazolylidene-based metal complexes reported by the groups of Raubenheimer^27,30^ (Adapted with permission from ref 27, copyright 2009 Royal Society of Chemistry and ref [Bibr ref30], 2012 Elsevier, respectively) and Kühn & Prokop^37^ (Reproduced from ref 37. Available under a CC-BY-NC-ND 4.0 license. Copyright 2024 Bannwart et al.) as well as a general scheme of the ones developed in this work.

In a previous work on tetrazolylidene derivatives, some of us demonstrated that *abnormal* 1,3-dimethyl-tetrazolylidenes exhibit stronger electron-donating properties and greater stability than their *normal* 1,4-dimethyl counterparts, enabling the successful synthesis of late-transition metal complexes (Ir, Mo, Ag) bearing such ligands.^28^ Building on these findings, two gold­(I) bis-NHC complexes bearing either dialkyl or diaryl substituted *abnormal* tetrazolylidene ligands were synthesized. Both complexes exhibit pronounced antiproliferative activity against leukemia cells (Nalm-6), inducing apoptosis at low nanomolar concentrations (IC_50_ ≤ 0.017 μM).^37^ Notably, the latter exhibits exceptional efficacy in overcoming drug resistance in several cancer cell lines (NiWi-Dau, BiBo, LiKa) while sparing healthy leukocytes, underscoring its potential clinical relevance. Encouraged by these findings and the promising pharmacological properties of this ligand class, the investigation has now been expanded to mono-tetrazolylidene gold­(I) derivatives to further explore their therapeutic potential. In this work, a series of seven *abnormal* tetrazolium salts featuring diverse alkyl and aryl substituents is examined, which can be applied as precursors to novel mesoionic tetrazolylidene gold­(I) chloride complexes (**1**–**7**). The obtained compounds have been structurally characterized by a variety of methods, including X-ray diffraction. Moreover, the anticancer potential of the gold­(I) compounds has been evaluated by assessing their antiproliferative profile in a small panel of human cancer cells vs nontumorigenic cells *in vitro*.

## Results and Discussion

### Synthetic Pathways to Abnormal 1,3-Tetrazolium Salts and Mesoionic Tetrazolylidene Gold­(I) Chloride Complexes **1–7**

With the aim of investigating how steric and electronic properties may affect their *in vitro* antiproliferative potential and selectivity against human cancer cells, we focused on three classes of tetrazolium-based ligand precursors and their respective gold­(I) chloride complexes. In particular, *abnormal* 1,3-dialkyl, diaryl and 1-alkyl-3-aryl substituted tetrazolium salts were in the scope of this investigation. A brief description of the synthetic strategy is provided to serve as an overview of this scarcely explored ligand class. The synthetic approaches can be divided into three main categories: (a) the 5-step buildup of the diaryl heterocyclic structure starting from respective arylhydrazines and arylisothiocyanates (**L**_**1–4**_), (b) the heterocyclization of polynitrogeneous linear structures with consecutive modification and (**L**_**5–6**_) (c) the dialkylation of 1*H*-tetrazole (**L**_**7**_), as depicted in Scheme 1.^37–39^ NMR characterization was performed after each synthetic step, confirming successful formation of all intermediates (Figures S1–S36).

**Scheme 1:**
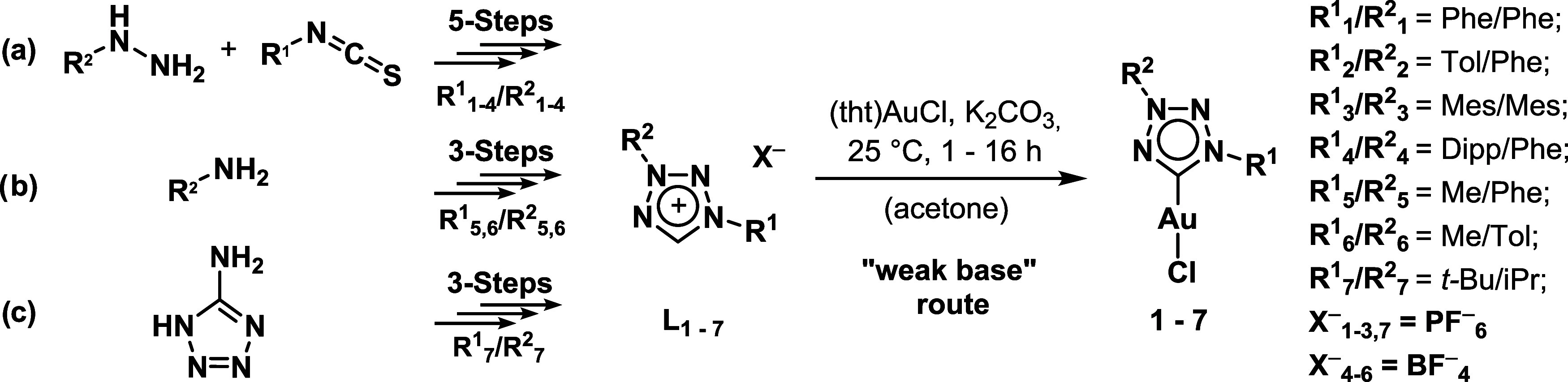
Overview of the Three Synthetic Pathways Applied to Obtain Abnormal 1,3-Tetrazolium-Based Ligand Precursors **L**_**1–7**_ Bearing Different Substitution Patterns (a) Diaryl; (b) 1-Alkyl-3-aryl; (c) Dialkyl and Synthesis of the Corresponding Gold­(I) Carbonic Complexes **1–7** ^37–39^

For the gold­(I) complexation reaction, the “weak base” approach was selected, as the relatively acidic tetrazolium proton can be readily deprotonated by inorganic bases such as potassium carbonate.^40^ The resulting carbenium ion readily forms a stable bond with the gold­(I) center under mild conditions (Scheme 1), while also facilitating a straightforward work**-**up by isolating the complex with dichloromethane from insoluble residues. The structure and purity of the complexes has been confirmed by NMR, ESI-MS, and elemental analysis (Figures S37–S69).

#### *Abnormal* 1,3-Diaryl Tetrazolium Salts **L**_**1–4**_

As recently reported, the synthesis of diaryl**-**substituted tetrazolium salts **L**_**1–4**_ utilizes phenylhydrazine and aryl**-**isothiocyanate in a modified ring closure synthetic pathway. With five literature known steps, the corresponding tetrazolium salts can be obtained (Scheme 2).^37^

**Scheme 2:**
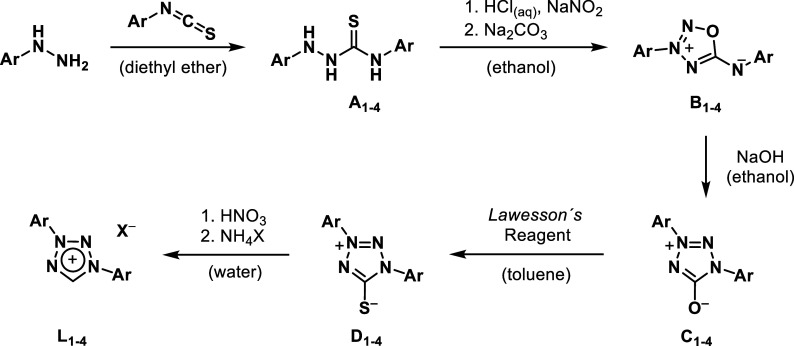
General 5-Step Synthetic Pathway toward 1,3-Diaryl Tetrazolium Salts **L**_**1–4**_

In the first step, the hydrazinecarbothioamides **A**_**1–4**_ are obtained by reacting the respective aryl**-**hydrazine with aryl-isothiocyanate in toluene in a straightforward condensation reaction. The crude product is obtained by filtration and is subjected to recrystallization with ethanol to obtain the product in high purity. In the second step, a ring closing reaction is performed using sodium nitrite to obtain the oxatriazole moiety **B**_**1–4**_. An acidic environment introduced by concentrated hydrochloric acid is essential in this reaction, as it facilitates the formation of the reactive nitrosyl cations responsible for cyclization. It additionally aids in the purification, as the product is only soluble in ethanol within a specific pH range (∼1–4). Therefore, the product is precipitated by elevating the pH with sodium carbonate after separating insoluble side products via filtration. Notably, we recommend the use of recently acquired sodium nitrite, as in our experience, aged or degraded batches lead to failing reactions, most likely due to reduced reactivity and insufficient generation of the nitrosylating species. In the following step, a rearrangement reaction to the respective tetrazolium**-**olate **C**_**1–4**_ derivative is performed upon heating in alkalized ethanol. In the next step, the tetrazolium**-**olate is converted to the tetrazolium**-**thiolate moiety **D**_**1–4**_ using *Lawesson′s* reagent, a typical thiation reagent widely used in organic chemistry.^41^ This step is important, as it provides the transformation to an easily reducible thiolate derivative, which can be removed by concentrated nitric acid in the final step. Precipitation with either NH_4_PF_6_ or NH_4_BF_4_ finally yields the diaryl tetrazolium ligand precursors **L**_**1–4**_.

#### *Abnormal* 1-Alkyl-3-aryl Tetrazolium Salts L_5–6_

Additionally, disubstituted tetrazolium salts bearing both aryl and alkyl substituents (**L**_**5–6**_) have been synthesized following the alkyl-aryl route, enabling a more direct comparison between the two substituent types. According to previous experience, methylation of 2-substituted tetrazoles yields 1,3-disubstituted tetrazolium salts as a single product, whereas the methylation of 1-substituted tetrazoles always produces a mixture of 1,3- and 1,4-disubstituted tetrazolium salts.^8^ The ratio of these two isomers can be controlled by variation of the reaction conditions. Initially, the nitrogen-based heterocycle needs to be constructed. Therefore, the corresponding arylamine is nitrated to the aryldiazonium salt, which is stabilized by the counterion BF_4_^–^. In the second step, the heterocyclization via [2 + 3] cycloaddition between the aryldiazonium salt and trimethylsilyldiazomethan is performed, yielding the 2-aryl-2*H*-tetrazoles species.^42^ In the final step, alkylation with the strong alkylating agent trimethyl oxonium tetrafluoroborate affords the 1-alkyl-3-aryl disubstituted tetrazolium salts (Scheme 3). A similar synthetic approach for the alkylation of 2-aryl-2*H*-tetrazoles was also recently described in a study by Baschieri et al.^39^

**Scheme 3:**
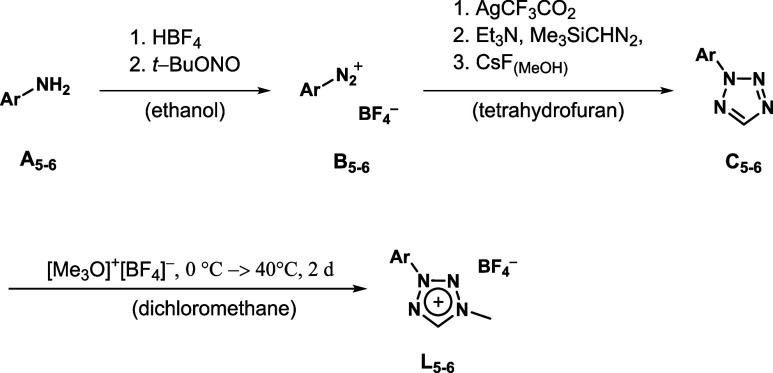
Complete Synthesis of 1-Alkyl-3-Aryl Tetrazolium Salts **L**_**5–6**_ Starting from the Respective Aryl Aniline

#### *Abnormal* 1,3-Dialkyl Tetrazolium Salts L_7_

Due to the limited commercial availability of tetrazole precursors, the synthesis of the alkyl-alkyl ligand precursor **L**_**7**_ requires the prior preparation of 1*H*-tetrazole **A**_**7**_. The reductive deamination of 5-aminotetrazole monohydrate proposed by Henry et al. was therefore chosen, using sodium nitrite in aqueous hypophosphorous acid.^43^ Hypophosphorous acid plays a dual role: it provides the acidic conditions necessary for the formation of nitrosyl cations and simultaneously reduces the highly unstable diazonium intermediate *in situ*. It is important to handle these compounds with caution due to their high nitrogen content, which imparts a well-known dense energetic character.^44^ The subsequent alkylation of ambident 1*H*-tetrazole poses a challenge in regioselectivity, as both the N2 and N4 nitrogen atoms exhibit comparable nucleophilicities. As a result, conventional alkylating agents such as alkyl halides or alkyl sulfonates commonly yield mixtures of 1- and 2-substituted regioisomers.^45,46^ To overcome this unselective reactivity, a strategy developed by Koren et al. has been adopted, which leverages the weakly basic nature of the five-membered ring and its high stability under strong acidic conditions.^47^ Unlike alkylation in organic media, the use of strong mineral acids (e.g., perchloric acid, sulfuric acid, tetrafluoro boric acid) revealed that blocking the most nucleophilic N4 atom by protonation allows very good selectivity avoiding the formation of the 4-alkylated tetrazolium species.^38^ Furthermore, alkylation of tetrazoles effectively takes place by reaction with alcohols that generate stable carbocations (e.g., isopropanol, *t*-butanol).^47–49^ Sequential alkylation under these conditions produces 1,3-dialkylated tetrazolium salts (Scheme 4).

**Scheme 4:**
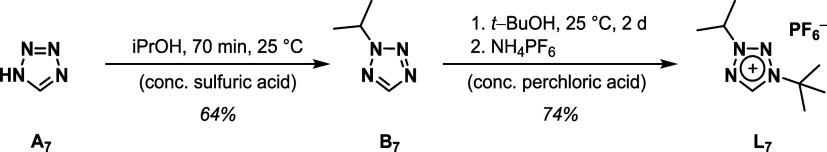
Synthesis of 1-*t*-Butyl-3-isopropyl-2*H*-tetrazolium Hexafluorophosphate **L**_**7**_ by Quaternization of 1*H*-Tetrazole by the Respective Alcohols in Concentrated Sulfuric Acid

An isopropyl carbocation, formed via acid-mediate dehydration of isopropanol, was employed as the electrophile to selectively generate 2-isopropyl-2*H*-tetrazole **B**_**7**_. Subsequent alkylation with *t*-butanol in perchloric acid, followed by precipitation with ammonium hexafluorophosphate from a water/acetone mixture, afforded the desired *abnormal* 1,3-dialkylated tetrazolium salt **L**_**7**_.

##### Gold­(I) Complexation Reactions

For the synthesis of the respective gold­(I) chloride complexes, the weak base synthetic approach was chosen, which is commonly applied for NHC gold­(I) complexes (Scheme 5).^40^

**Scheme 5:**
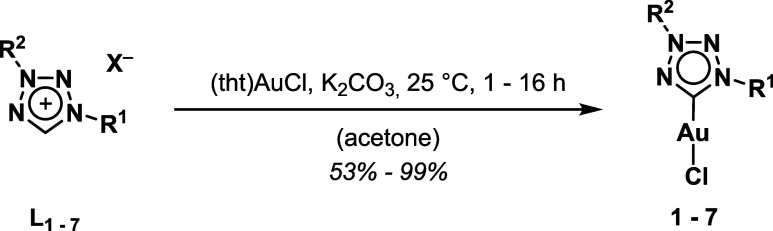
Synthesis of 1,3-Tetrazolylidene Gold­(I) Mono-Complexes **1–7** Following the Weak Base Route

Therefore, the gold precursor and the tetrazolium proligand were dissolved in acetone in equimolar stoichiometry and potassium carbonate is added. After stirring for 2 h at 25 °C, a color change of the solution was observed, and the insoluble residues turned dark blue. The reaction progress was monitored by ESI-MS and showed completion after ca. 16 h, as no free ligand precursor was detected. The ESI-MS further confirmed the formation of the mono-NHC gold­(I) complex, as well as, in most cases, the formation of the cationic bis-NHC complex. In case of **4**, only the mono-NHC complex forms, indicating that the steric demanding diisopropylphenyl moiety hinders the coordination of an additional ligand to the metal center. After the reaction time, the solvent is removed *in vacuo* and the residue was suspended in dichloromethane and filtered over Celite, followed by precipitation with *n*-pentane. Further examination of the crude products by TLC indicates a strong difference in polarity between the mono- and bis-NHC complexes; therefore, purification by simple filtration over a plug of silica was sufficient to isolate the desired complexes in moderate to high yields.

The disappearance of the characteristic signal corresponding to the H_tetr_ moiety at around 9.62–11.31 ppm in the ^1^H NMR spectrum indicates the successful synthesis of complexes **1**–**7**. Interestingly, the carbene carbons of the complexes show chemical shifts in the range of 176.7–180.3 ppm that are significantly downfield shifted when compared to those of other mesoionic carbenes such as 1,2,3-triazolylidene or imidazol**-**4**-**ylidene gold­(I)**-**chloride complexes.^50–52^ This indicates that *abnormal* tetrazolylidenes notably vary in their electronic properties and exhibit an electronic profile closer to that of classical, *normal* substituted imidazolylidenes.^53^

### X-ray-Structure Determination

Single crystals suitable for X–ray diffraction of six tetrazolylidene gold­(I) chloride complexes (**1**–**6**) were grown by slow diffusion of diethyl ether into a saturated acetonitrile solution of the complex at room temperature and under the exclusion of light. Figure 2 displays the ORTEP style crystal structures of compounds **1**–**6**.

**Figure 2:**
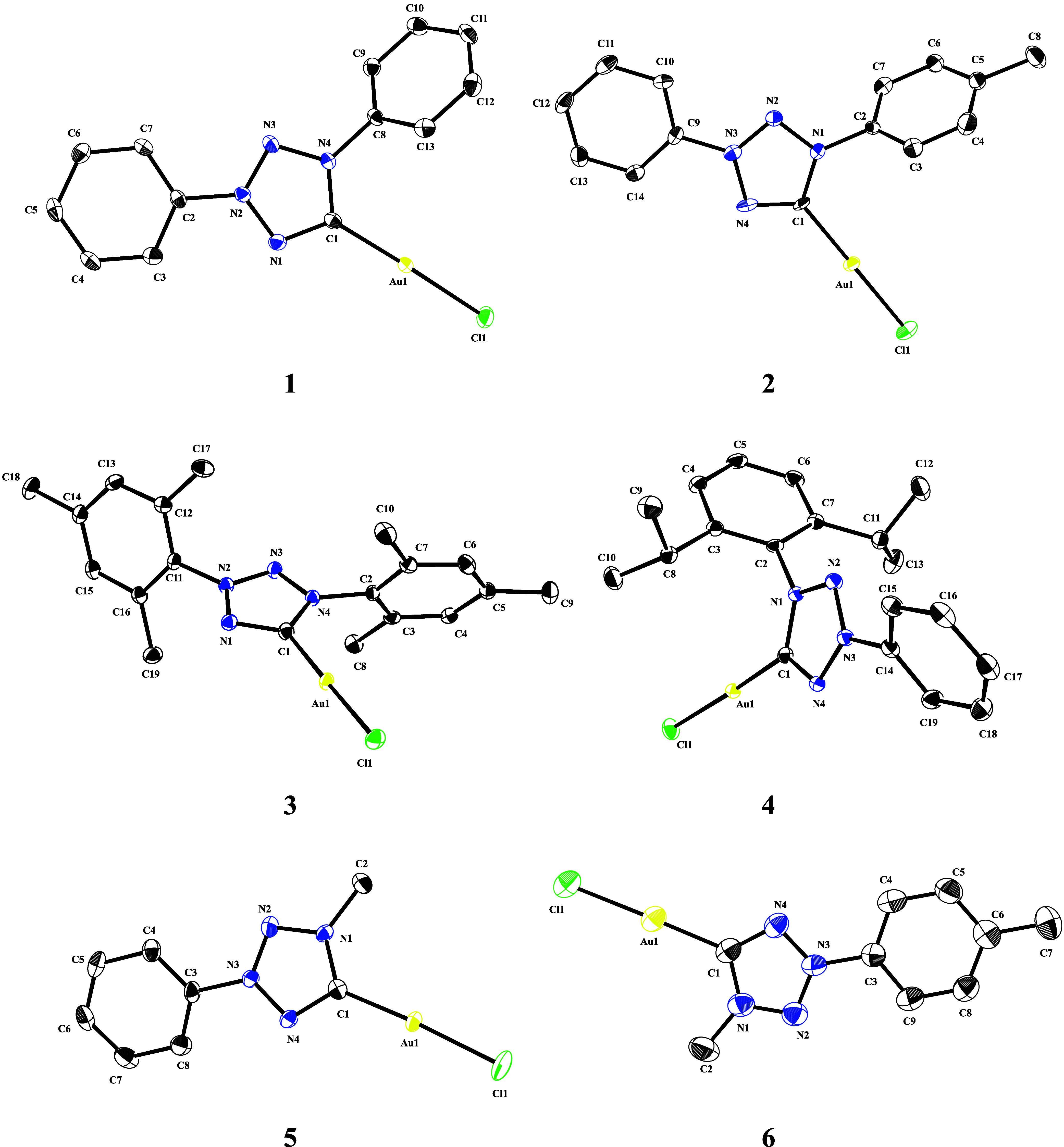
ORTEP style representation of tetrazolylidene gold­(I) complexes **1**–**6**, with ellipsoids shown at a 50% probability level. Hydrogen atoms and counteranions are omitted for clarity.

For comparison to related structures, selected bond lengths and angles are provided in Table 1.

**Table 1:** Selected Bond Lengths (Å) and Angles (°) of Compounds **1**–**6**

Compound	**1**	**2**	**3**	**4**	**5**	**6**
C_carbene_–Au	1.977(3)	1.996(5)	1.972(2)	1.966(2)	1.978(4)	1.976(7)
Au–Cl	2.2790(6)	2.2914(13)	2.2746(5)	2.2792(5)	2.2895(10)	2.2749(18)
C_carbene_–Au–Cl	177.86(7)	178.02(17)	177.93(6)	176.59(6)	176.49(11)	179.0(2)
N_1_–C_carbene_–N_4_	106.1(2)	107.7(4)	105.71(17)	105.65(18)	106.6(3)	105.4(6)

The tetrazolylidene metal bond distances in all complexes are in the range from 1.966(2) Å for **4** to 1.996(5) Å in **2**, comparable to other examples of NHC gold­(I) chloride complexes.^50,51,54^ The Au–Cl bond lengths range from 2.2749(18) Å in complex **6** to 2.2914(13) Å in complex **2**, which is in good agreement with similar imidazolylidene and triazolylidene gold­(I) chloride compounds.^51,54–56^ The steric demand of the bulky substituents leads to slight deviations from a perfect linear geometry around the gold­(I) center ranging from 176.49(11)° for **5** to 179.0(2)° for **6**, which is in alignment with other closely related compounds.^54^ The geometry of the five**-**membered heterocycle of the *abnormal* tetrazolylidene complexes **1**–**6** is close to a regular pentagon (all angles around 106°), while it is somewhat distorted in the *normal* tetrazolylidene complex (N–C–N 101.3°) as previously reported.^28^ This significant difference, in line with previous reports of Cr, Ir, and Ag tetrazolylidene complexes, can be attributed to the steric demand of the wingtip substituents, as well as electronic differences between the two substitution patterns.^28^ Overall, the structural data suggest that the electronic properties across this new ligand class are broadly comparable. Although subtle variations in the Au–C and Au–Cl bond lengths were observed, such small differences fall within the typical range reported for NHC**-**gold­(I) complexes and are therefore unlikely to reflect major changes in electronic character.^57,58^ This further supports the previous finding showing that *abnormal* tetrazolylidene ligands exhibit σ**-**donor strengths similar to those of *normal* imidazolylidenes.^31^

### Stability Studies

The stability of the 1,3**-**disubstituted tetrazolylidene gold­(I) complexes (**1**–**7**) was investigated in the presence of biologically relevant thiols, using *L* **-**cysteine (Cys) and glutathione (GSH) as model nucleophiles. Reactivity studies were carried out using a solvent system consisting of DMSO**-** *d*_6_ and D_2_O (4:1), suitable to dissolve both the complex and the respective thiol species. The progress was monitored via ^1^H NMR spectroscopy, with the primary objective of assessing the susceptibility of these complexes to ligand exchange processes simulating thiol**-**rich biological environments. Prior to the addition of the thiol-containing compound, the complexes were examined for solvent stability. All seven complexes show no sign of decomposition in a 4:1 mixture of DMSO**-** *d*_6_ and D_2_O at 37 °C, over 72 h (Figures S76–S82). After addition of *L* **-**cysteine (Cys, 2.00 equiv), new signals appear instantaneously in the ^1^H**-**NMR spectrum at 25 °C (Figures S83–S89), indicating rapid coordination of the thiol to the gold­(I) center and formation of the corresponding thiolate complex as shown for complex **5** in Figure 3. Identical reactivity is observed with GSH (2.00 equiv) (Figures S98–S104). The formation of the respective *L* **-**cysteinate and tetrazolylidene complexes **1**–**7­(Cys)** was further confirmed by ESI**-**MS (Figures S91–S97) and the general reaction scheme is depicted in Figure 3A.

**Figure 3:**
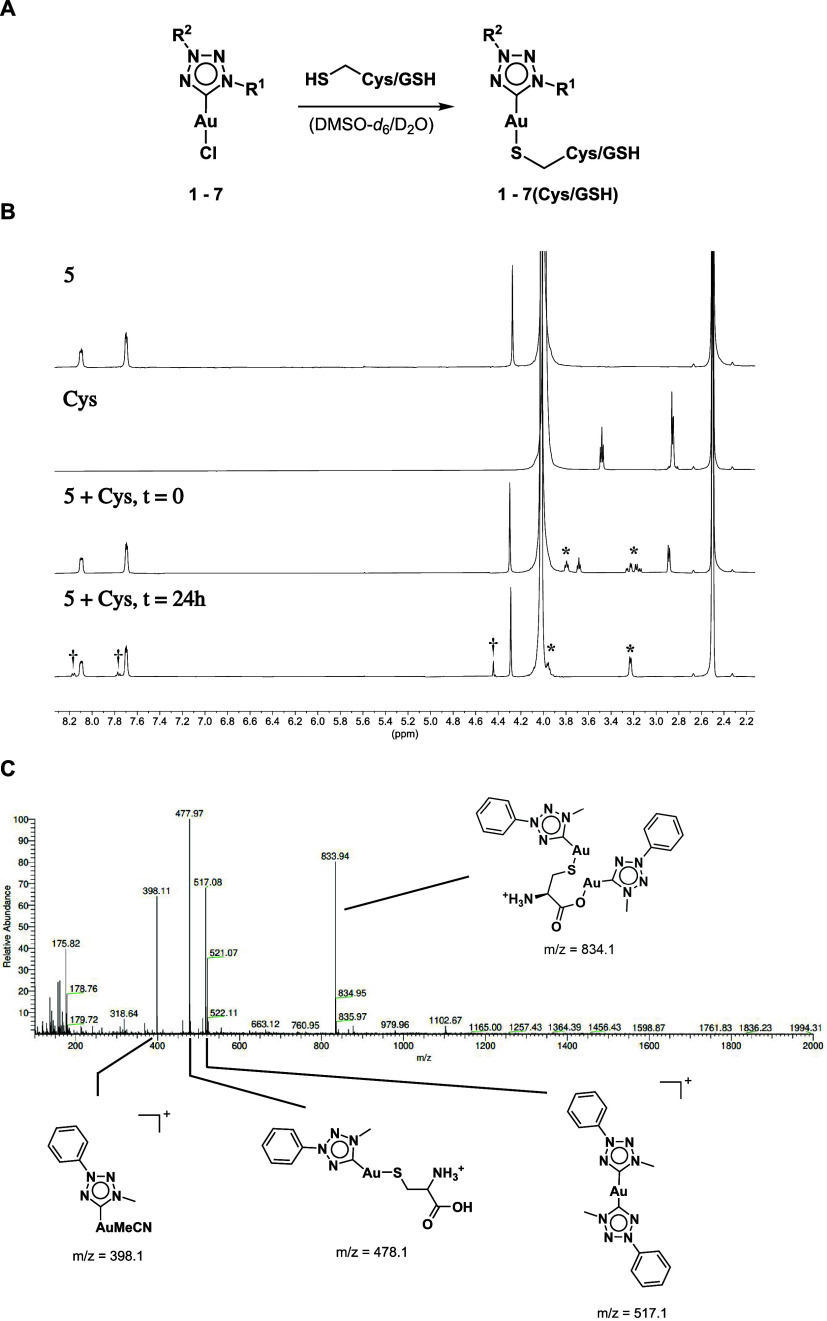
(A) Reaction of the compounds of interest **1**–**7** with the respective model nucleophiles to form the thiolate complexes **1**–**7­(Cys/GSH)**. (B) ^1^H NMR spectra in DMSO-*d*_6_/D_2_O (4:1) of complex **5** and after the addition of 2.00 equiv Cys at 0 and 24 h incubation at 37 °C; the Cys spectrum is also reported as a reference. Newly formed peaks are labeled with * for the signals corresponding to the coordinating cysteinate and with † for signals corresponding to the gold­(I) bis**-**NHC complex. (C) ESI**-**MS of the reaction mixture after 72 h.

Interestingly, the thiolate complexes gradually convert to the corresponding gold­(I) bis**-**NHC complexes (**1**–**7­(bis)**) over 72 h incubation, as exemplified for complex **5** in Figure 3B. It should be noted that excess cysteine signals decreases due to autoxidation to cystine, which is insoluble in the solvent system used, as evidenced by the corresponding blank measurement (Figure S90).^59^ ESI**-**MS confirms the formation of the bis**-**NHC Au­(I) complex detected at 517.1 *m*/*z* (Figure 3C).

For a comparative analysis across the series, all complexes (**1**–**7**) were incubated with Cys (2.00 equiv) for 72 h at 37 °C. The relative formation of the bis**-**NHC Au­(I) complexes (Figure 4A) was quantified by integrating characteristic regions of the ^1^H NMR spectra (Figures S83–S89) corresponding to each species. The resulting time**-**dependent evolution of **1**–**7­(bis)** is shown in Figure 4B.

**Figure 4:**
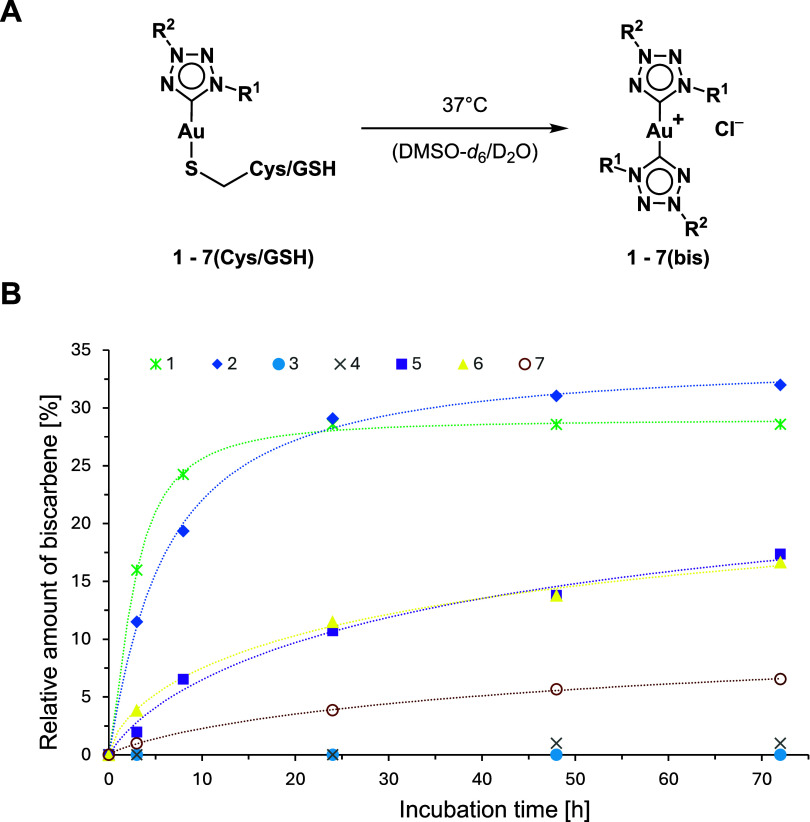
(A) General reaction of **1**–**7­(bis)** formation from incubation of **1**–**7­(Cys/GSH)** in DMSO-*d*_6_/D_2_O. (B) Evolution of **1**–**7­(bis)** after incubation of **1**–**7** with 2.00 equiv of Cys in DMSO-*d*_6_/D_2_O (4:1) over 72 h.

Analysis of the reaction kinetics reveals distinct trends that correlate with the ligand substitution pattern and related steric effects. Complexes **3** and **4**, bearing bulky wingtip substituents, exhibit negligible conversion to the corresponding bis-NHC species, whereas diaryl-substituted complexes with phenyl wingtips (**1** and **2**) show substantial formation of **1**–**2­(bis)**, reaching up to 32% after 72 h. Complexes **5** and **6**, featuring small methyl wingtips, display moderate reactivity with approximately 17% conversion, while the *t*-butyl substituted complex **7** shows only a minor degree of bis-NHC formation (≈ 7%). The fact that bis-NHC formation occurs only after coordination of the thiolate, indicates that nucleophilic activation of the gold center is essential for such ligand scrambling processes, as the solvent stability studies shows no such reactivity even after 72 h (Figures S76–S82). In all cases, the reaction progress reached a plateau after approximately 72 h.

### *In Vitro* Antiproliferative Activity Studies

To evaluate the potential as anticancer agents, complexes **1**–**7** and selected tetrazolylidene precursors (**L**_**2**_, **L**_**3**_, **L**_**5**_, **L**_**7**_) were investigated using a cell proliferation assay (MTT) against A2780 (ovarian carcinoma), A549 (lung carcinoma), and noncancerogenic VERO E6 (kidney epithelial cells). Cisplatin and auranofin were included as benchmarks. EC_50_ values and corresponding selectivity indices (SI = EC_50_^noncancerogenic^/EC_50_^cancerogenic^) are summarized in Table 2.

**Table 2:** MTT Assay after 72 h of Incubation (*n* ≥ 3)^a^

Compound	**A2780**	**A549**	**VERO E6**	**SI** ^**A2780**^, **SI** ^**A549**^
Cisplatin	0.46 ± 0.12	6.07 ± 1.14	1.56 ± 0.33	3.40, 0.257
Auranofin	0.21 ± 0.03	7.14 ± 1.61	5.46 ± 1.71	26, 0.765
**L**_**2**_	42.71 ± 10.53	>100	>100	–
**L**_**3**_	13.05 ± 2.22	21.95 ± 5.59	47.27 ± 4.03	3.62, 2.15
**L**_**5**_	>100	>100	>100	–
**L**_**7**_	>100	>100	>100	–
**1**	0.35 ± 0.20	3.35 ± 0.39	25.87 ± 2.18	73.9, 7.72
**2**	0.34 ± 0.17	3.71 ± 0.77	21.48 ± 3.46	63.2, 5.79
**3**	0.23 ± 0.13	10.89 ± 1.15	19.95 ± 4.64	86.7, 1.83
**4**	0.88 ± 0.12	13.34 ± 3.71	10.23 ± 3.23	11.6, 0.767
**5**	0.15 ± 0.03	27.22 ± 0.63	11.01 ± 2.95	73.4, 0.404
**6**	0.39 ± 0.24	24.44 ± 9.30	10.84 ± 1.67	27.8, 0.444
**7**	0.27 ± 0.17	41.34 ± 14.61	22.47 ± 2.67	83.2, 0.544

a EC_50_ are Given in μM. SI: Selectivity Index = EC_50_^non‑cancerogenic^/EC_50_^cancerogenic^.

The disubstituted gold­(I) complexes **1**–**7** exhibit outstanding antiproliferative effects against A2780 cells, with EC_50_ values ranging from 0.15 ± 0.03 μM to 0.88 ± 0.12 μM, in some instances outperforming cisplatin (0.46 ± 0.12 μM) and matching the potency of auranofin (0.21 ± 0.03 μM). Importantly, most of these compounds show high selectivity indices toward non**-**cancerogenic cells (SI^A2780^ = 63.2–86.7), demonstrating reduced antiproliferative activity against normal kidney VERO E6 cells (EC_50_ = 19.95 ± 4.64 μM–25.87 ± 2.18 μM). While most of the complexes were markedly less active against A549 cells, complexes **1** and **2** also maintain notable activity (EC_50_ = 3.35 ± 0.39–3.71 ± 0.77 μM), exceeding potency and selectivity (SI^A549^ = 5.79–7.72) compared to both reference compounds cisplatin and auranofin.

Regarding the precursors, **L**_**2**_, **L**_**5**_, and **L**_**7**_ show low to no measurable activity (EC_50_ > 100 μM for **L**_**5**_ and **L**_**7**_, 42.71 ± 10.53 μM for **L**_**2**_), confirming that coordination to the gold­(I) center is crucial for inducing an antiproliferative effect. **L**_**3**_ shows moderate activity against A2780 (13.05 ± 2.22 μM) and A549 (21.95 ± 5.59 μM), with only slight selectivity (SI^A2780^ = 3.62, SI^A549^ = 2.15), reinforcing the key role of the gold­(I)-NHC scaffold in the overall bioactivity.

Previously reported classical imidazolylidene and benzimidazolylidene gold­(I) chlorido mono-NHC complexes bearing modifications at the wingtip or backbone positions, such as halogen, phenyl, or alkyl substituents, did not necessarily exhibit pronounced selectivity between cancerous and noncancerous cell lines. Across the respective cell panels, these complexes typically displayed EC_50_ values in the low micromolar range (≈ 1–17 μM). Differences in antiproliferative activity were generally modest between the tested cell origins, and significant selectivity toward noncancerous cells was not observed.^60–62^ In contrast, a mono-NHC gold­(I) complex bearing pyrrole and benzyl *N*-substituents exhibited selectivity toward A2780 ovarian cancer cells and their resistant counterpart, with EC_50_ values in the moderate range (23–28 μM), while no activity was detected against liver cancer cell lines (HepG2/HepAD38) or the noncancerogenic kidney cell line MDCK.^63^ By exchanging the wingtip moieties for a methyl and quinolone scaffold, the respective mono-NHC complex gained activity toward ovarian cancer cell lines (EC_50_ = 3–9 μM), which is still up to 24 times less active than complexes **1** and **2** reported in this work. However, this modification additionally resulted in reduced selectivity toward the liver cancer cell lines and the noncancerogenic kidney cell line.

This example illustrates how challenging the fine-tuning process is when aiming to achieve high activity in the submicromolar range for a cell line of a certain origin while maintaining distinct differences in EC_50_ values across different cell lines and pronounced selectivity for cancerous over noncancerous cells. Although the specific cell lines employed in these studies mostly differ from those investigated in the present work, the overall trend of either comparable antiproliferative effects with low selectivity or markedly lower activities accompanied by a high selectivity remained consistent after 3 days of exposure. Further structural variations toward more nitrogen-rich analogues of benzimidazolylidene mono-NHC complexes, such as methylcaffeineylidene ligands, resulted in reduced antiproliferative activity under identical incubation conditions. In these cases, moderate EC_50_ values of 23–37 μM were reported against A2780 ovarian and A549 lung cancer cell lines, indicating substantially lower activity than that observed for the complexes described in this work.^64^

Gold tetrazolylidene mono-NHC complexes remain comparatively rare in the literature, and only a limited number have been evaluated for their antiproliferative activity against cancer cell lines. Consequently, very few structurally related mono-NHC complexes are available for direct comparison. One literature example that bears structural similarity to the mono-NHC complexes described herein is a phosphine­(tetrazolate) gold­(I) complex, in which the metal center is coordinated through nitrogen rather than carbon within the four-nitrogen-membered heterocycle. This mono-NHC complex exhibited a low micromolar EC_50_ value of 1.3 μM against HeLa cervical cancer cells and showed a modest selectivity index of 2 toward healthy human lymphocytes.^30^

In contrast, the tetrazolylidene gold­(I) mono-NHC complexes described in this work exhibit distinct advantages, most notably the large differences in activity across cancer cell lines (≈ 10–180-fold) when comparing ovarian A2780 and lung A549 cancer cells. Moreover, high selectivity indices of 12–87 were observed, indicating a pronounced preference for cancer cells over nontumorigenic cells (Table 2). Submicromolar EC_50_ values against ovarian cancer cells further underscore a substantially improved selectivity profile relative to previously reported NHC-gold­(I) mono-NHC complexes and highlight the potential of this ligand class to fine-tune anticancer activity toward specific tumor origins in future studies.

To further investigate the influence of structural rearrangements and interactions with nucleophiles and thiols present in the medium on antiproliferative activity, the two most active compounds toward A549 lung cancer cells (mono-NHCs **1** and **2**) were dissolved in DMF and subsequently diluted in DMEM. One sample contained FCS (corresponding to the standard conditions of the 72 h MTT assay), while the other was prepared without FCS. The solutions were preincubated at 37 °C for 48 h, then further diluted with FCS-containing medium and immediately applied to A549 cells, followed by incubation for an additional 24 h under standard conditions. (Figure 5 and Table S3).

**Figure 5:**
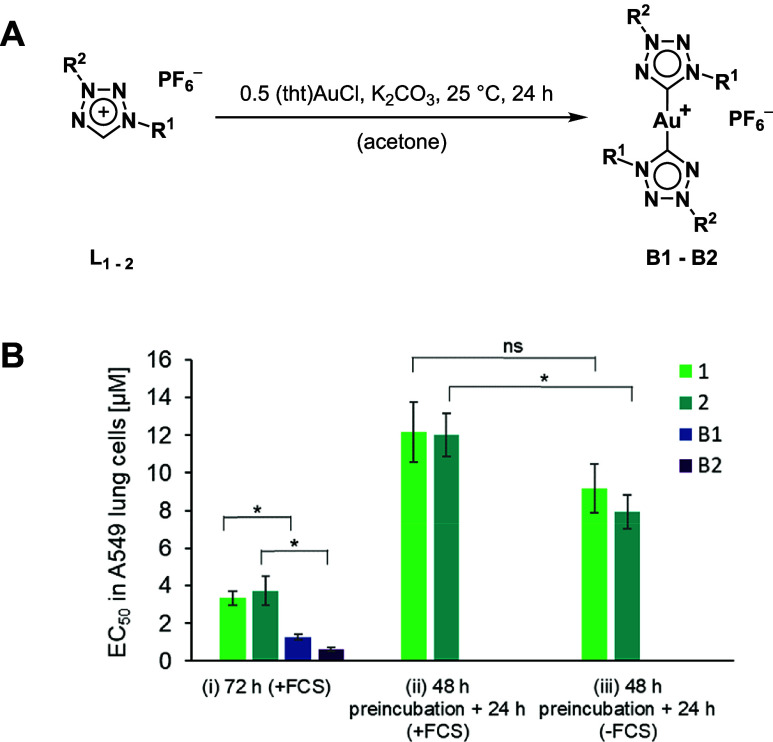
(A) Reaction scheme for the synthesis of bis-NHC complexes **B1** and **B2**. (B) EC_50_ values (μM) for A549 cells of mono-NHC complexes (**1** and **2**) and bis-NHC complexes (**B1** and **B2**), determined in triplicate under different incubation conditions: (i) 72 h continuous exposure; (ii) 48 h preincubation in medium containing FCS followed by 24 h incubation on cells; and (iii) 48 h preincubation in medium without FCS followed by 24 h incubation on cells. Data represent the mean ± standard deviation of three independent experiments. An asterisk (*) indicates a statistically significant difference (*p* < 0.05) between EC_50_ values as determined by a two-tailed Student’s *t* test (ns = not significant).

Comparison with the 72 h incubation data shows that preincubation in the FCS-containing medium led to a pronounced inactivation of both complexes **1** and **2**. Overall, their activity was reduced by approximately 3- to 4-fold. When preincubation was performed in the absence of FCS, the reduction in activity was limited to approximately 50–70%. These results indicate that prolonged exposure to serum components, particularly albumin as the major constituent, significantly contributes to the observed inactivation of the mono-NHC complexes.

The remaining loss of activity is likely attributable to additional processes, such as compound decomposition, precipitation, interactions with thiol-containing amino acids present in the medium, or rearrangement reactions leading to the formation of other, as yet uncharacterized species. These findings are consistent with previously published data on gold­(I) mono- and bis-NHC complexes of the imidazolylidene/benzimidazolylidene type.^61,62,65^ Interestingly, the cationic gold­(I) bis-NHC analogues of **1** and **2** (**B1** and **B2**) exhibit 3- to nearly 7-fold higher antiproliferative activity than their mono-NHC derivatives after direct 72 h incubation with the cells (Table S3). In general, gold­(I) bis-NHC complexes of the imidazolylidene and tetrazolylidene types are delocalized lipophilic cations (DLCs) known to readily cross cellular membranes, accumulate in subcellular compartments such as mitochondria, and therefore display enhanced antiproliferative activities toward cancer cells *in vitro*, and greater kinetic stability compared to mono-NHC analogues.^37,61,63,65^ Hence, bis-NHC species show less reactivity toward nucleophiles, a higher cellular uptake and can be significantly more active than the respective mono-NHC complexes.^37,65^ If substantial structural rearrangement toward the bis-NHC complex would occur in the cell culture, this would result in increased antiproliferative activity, contrary to the present observations (Figure 5). Therefore, it can be assumed that, prior to any significant bis-NHC complex formation in the biological medium, faster inactivation processes predominate and are primarily responsible for the observed activity of complexes **1** and **2**.

## Conclusion and Outlook

A comprehensive study of mesoionic tetrazolylidene gold­(I) chloride complexes (**1**–**7**), integrating synthesis, structure, and preliminary biological evaluation, has been conducted. The established synthetic routes provide access to *abnorma*l 1,3-disubstituted tetrazolylidene ligands with diverse steric profiles. Crystallographic and spectroscopic data, along with the previous investigations into the electronic and steric effects of *abnormal* tetrazolylidenes, suggest that these ligands possess electronic properties comparable to those of classical NHCs, but exhibit a distinctly different steric profile.^31^ This open environment leads to rapid and complete reaction with biologically relevant thiols, leading to the formation of gold-thiolate species under mild conditions. Biological screening demonstrated potent and selective antiproliferative activity, particularly against the A2780 ovarian cancer cell line, with several complexes surpassing the efficacy of cisplatin and matching that of auranofin. Interestingly, the cationic gold­(I) bis-NHC analogues **B1** and **B2** exhibit pronounced antiproliferative effects in A549 cells, and their greater stability under physiological conditions may be advantageous. These findings establish *abnormal* tetrazolylidenes as a promising and versatile ligand class for the design of gold-based anticancer agents with high reactivity and selectivity.

## Experimental Section

### General

Unless otherwise noted, all reactions were conducted without taking special precautions to exclude air and water. Solvents were distilled prior to use. Unless otherwise noted, chemical reagents and solvents were purchased from commercial suppliers (Sigma-Aldrich, TCI, Acros, Fisher Scientific) and used as received. Anhydrous solvents were obtained water- and oxygen-free from a *M. Braun* SPS purification system and stored over molecular sieves 3 Å. Column chromatography was carried out using silica gel 60 (*Acros*, 0.060–0.200 mm). For thin layer chromatography TLC silica gel 60 F_254_ was used. The spots were visualized with UV light (254 nm). ^1^H and ^13^C­{^1^H} NMR spectra were recorded on a *Bruker* AV400-US or a *Bruker* AV 500 Cryo spectrometer. All ^1^H and ^13^C­{^1^H} chemical shifts are reported in parts per million [ppm] and were referenced to the residual signal of the deuterated solvents (MeCN-*d*_3_, ≥ 99.8 atom %); (DMSO-*d*_6_, ≥ 99.8 atom). As for correlation of the signals and their multiplicities, the following abbreviations were used: s—singlet, d—doublet, t—triplet, q—quartet, qu—quintet, m—multiplet. The stated coupling constants *J* are denoted as the average of the experimentally found values and are given in hertz [Hz]. Electrospray ionization-mass spectrometry (ESI-MS) data were acquired on a *Thermo Fisher* Ultimate 3000 using acetonitrile as eluent additive. The elemental analyses were carried out on a Vario EL from the company *Elementar* at the Catalysis Research Center of the *Technischen Universität München* in the microanalytical laboratory. SC-XRD Data were collected on a *Bruker* D8 Venture single crystal X-ray diffractometer equipped with a CMOS detector (*Bruker* Photon-100), an IMS microfocus source with Mo Kα radiation (λ = 0.71073 Å) and a Helios optic using the APEX4 software package. Measurements were performed on single crystals coated with perfluorinated ether. The crystals were fixed on top of a Kapton micro sampler and frozen under a stream of cold nitrogen. A matrix scan was used to determine the initial lattice parameters. Reflections were corrected for Lorentz and polarization effects, scan speed, and background using SAINT. Absorption corrections including odd and even ordered spherical harmonics were performed using SADABS. Space group assignments were based upon systematic absences, E statistics, and successful refinement of the structures. The structures were dissolved using SHELXT with the aid of successive difference Fourier maps and were refined against all data using SHELXL in conjunction with SHELXLE. Hydrogen atoms were placed in calculated positions and refined using a riding model, with methylene, aromatic, and other C–H distances of 0.99 Å, 0.95 Å and 1.00 Å, respectively, and U_iso_(H) = 1.2 U_eq(C)_. Non-hydrogen atoms were refined with anisotropic displacement parameters.

Full-matrix least-squares refinements were performed by minimizing ∑*w*(*F*_*c*_^2^ – *F*_*o*_^2^)^2^ with the SHELXL weighting scheme. Neutral atom scattering factors for all atoms and anomalous dispersion corrections for the non-hydrogen atoms were taken from International Tables for Crystallography. Images of the crystal structures were generated with Ortep3. Full experimental procedures, including reagent quantities, purification details, and characterization data, are provided in the Supporting Information. The identity and purity (>95%) of all biologically studied compounds were confirmed with elemental analysis and NMR spectroscopy.

### Synthetic Procedures

#### General Procedures for the Synthesis of 1,3-Diaryl Substituted Ligand Precursors **L**_**1–4**_

*N*-2-diaryl-hydrazinecarbothioamides (**A**_**1–4**_), 5-arylimino-3-aryl-[1,2,3,4]-oxatriazolium betaines (**B**_**1–4**_), 1,3-diaryl-1,2,3,4-tetrazolium-5-olates (**C**_**1–4**_), 1,3-diaryl-1,2,3,4-tetrazolium-5-thiolates (**D**_**1–4**_) and 1,3-diaryl-tetrazolium salts (**L**_**1–4**_) are synthesized according to modified literature procedures.^37^

#### General Procedure for the Synthesis of 1-Alkyl-3-aryl Substituted Ligand Precursors **L**_**5–6**_

Aryldiazonium tetrafluoroborate salts **L**_**5–6**_ are prepared by diazotization of the corresponding arylamines with *t*-butyl nitrite in the presence of aqueous tetrafluoroboric acid at 0 °C. The obtained diazonium salts (**A**_**5–6**_) are then converted to 2-aryl-2*H*-tetrazoles following a [3 + 2] cycloaddition reaction using trimethylsilyldiazomethane in the presence of silver triflate and triethylamine at low temperature. After workup and purification by either column chromatography (**B**_**5**_) or sublimation (**B**_**6**_), the corresponding tetrazoles are isolated as crystalline solids. Subsequent *N*-methylation of the 2-aryl-2*H*-tetrazoles with trimethyloxonium tetrafluoroborate in dichloromethane under inert atmosphere affords the respective 1-methyl-3-aryl-2*H*-tetrazol-4-ium tetrafluoroborate salts **L**_**5–6**_.

#### Procedure for the Synthesis of 1,3-Dialkyl Substituted Ligand Precursor **L**_**7**_

The 1,3-dialkyl-substituted ligand precursor **L**_**7**_ is obtained via acid-catalyzed alkylation of 1*H*-tetrazole with the corresponding alcohol in concentrated sulfuric acid at ambient temperature. After completion, the reaction mixtures is neutralized and extracted with organic solvent to afford the desired 2-alkyl-2*H*-tetrazole as a brown oil. Further alkylation of the 2-alkyl-2*H*-tetrazole with a tertiary alcohol under strongly acidic conditions yields the corresponding 1,3-dialkyl-2*H*-tetrazolium salt **L**_**7**_. The crude product is converted to the hexafluorophosphate salt by anion exchange with ammonium hexafluorophosphate, giving the dialkyl-substituted tetrazolium salt **L**_**7**_ as stable crystalline solid.

#### General Procedure for the Synthesis of 1,3-Disubstituted-tetrazolylidene Gold­(I) Chloride Complexes **1**–**7**

The 1,3-disubstituted tetrazolium salts **L**_**1–7**_ are deprotonated in the presence of potassium carbonate and reacted with equimolar amounts of chloro­(tetrahydrothiophene)­gold­(I) in acetone at room temperature. After completion, the reaction mixtures are filtered, concentrated, and the crude products are precipitated with *n*-pentane. The resulting solids are purified by filtration through a short silica plug with DCM to afford the respective 1,3-disubstituted tetrazolylidene gold­(I)-chloride complexes **1**–**7**.

#### General Procedure for the Synthesis of 1,3-Disubstituted-tetrazolylidene Gold­(I) Bis-NHC Complexes **B1**–**B2**

The 1,3-disubstituted tetrazolium salts **L**_**1–2**_ are deprotonated in the presence of potassium carbonate and reacted with half the equivalents of chloro­(tetrahydrothiophene)­gold­(I) in acetone at room temperature. After completion, the reaction mixtures are filtered, concentrated, and the crude products are precipitated with *n*-pentane. The resulting solids are purified by filtration through a short silica plug with DCM to remove formed mono-NHC complex followed by flushing with DCM/MeOH to afford the respective 1,3-disubstituted tetrazolylidene gold­(I) bis-complexes **B1**–**B2** as previously reported.^37^

### Stability Studies

All manipulations were performed using degassed solvents to reduce fast oxidation of the model nucleophiles. Complexes **1**–**7** were individually dissolved in 500 μL of a DMSO-*d*_6_/D_2_O mixture (4:1, v/v). For solvent stability tests, sealed samples were incubated at 37 °C, and ^1^H NMR spectra were recorded at different time points up to 72 h to monitor any decomposition or chemical shifts indicative of structural changes over time. Reactivity toward biological relevant thiol species was assessed at 25 °C. Complexes **1**–**7** were dissolved in 500 μL of DMSO-*d*_6_/D_2_O (4:1 v/v) and reactions were initiated by adding *L*-cysteine or glutathione (GSH) (2.00 equiv). ^1^H NMR spectra were immediately acquired postaddition to capture rapid exchange processes. Afterward the samples were incubated at 37 °C and ^1^H NMR spectra were recorded at different time points up to 72 h for Cys and 24 h for GSH. The reaction mixtures were subsequently analyzed by ESI-MS to identify gold–thiolate adducts and other species.

### Cell Culture Maintenance

A2780 human ovarian cancer cells, A549 human lung adenocarcinoma cells and VERO E6 noncancerogenic epithelial cells from *Chlorocebus sabaeus* (Western Vervet Monkey) were maintained in either RPMI 1640 (A2780) or Dulbecco’s modified Eagle’s medium (high glucose, pyruvate, no glutamine) (A549/VERO E6), which were both supplemented with heat- inactivated fetal bovine serum (qualified, South American origin, 10% v/v), gentamicin sulfate solution 50 mg/mL (1% v/v, 50 mg/L end concentration), and *L*-glutamine solution 200 NM (1% v/v, 2 nM end concentration) and were passaged twice a week. All reagents were purchased from GibcoTM at Thermo Scientific, and solvents from Sigma-Aldrich if not stated differently. Ultrapure water (18.2 MΩ/0.56 μS at 25 °C) was provided by a BerryPuremini from Berrytec. A2780 and VERO E6 cells were obtained from Cytion Cell Line Service (CLS, Eppelheim, Germany) and A549 cells from the DSMZ Helmholtz Institute for Infection Research (HZI, Braunschweig, Germany).

### Antiproliferative Effects in Cancerous and Non-Tumorigenic Cells

A2780 cells (6,000 cells/well), A549 cell (6,000 cells/well) or VERO E6 cells (8,000 cells/well) were transferred to a flat-bottom 96-well microtiter plate and incubated at 37 °C/5% CO_2_ for 24 h. Stock solutions of the compounds were freshly prepared in DMF and diluted with the respective cell culture medium to graded concentrations (final concentration of DMF: 0.1–0.2% v/v, depending on the solubility of the compounds). After 72 h of exposure the cell culture medium was disposed, and the remaining cell biomass of living cells was quantified by 3-(4,5-dimethylthiazol-2-yl)-2,5-diphenyl tetrazolium bromide (MTT) (Sigma-Aldrich) staining and the absorptions were calculated as the delta of two measurements of the same well at wavelengths 570 (MTT) and 690 nm (background) by an Infinite 200pro plate reader (Tecan). The EC_50_ values (half-maximal effective concentrations) were determined as the concentration that caused 50% inhibition of cell proliferation compared to untreated control (DMF in RPMI/DMEM). The data represent the mean of at least three independent experiments.

### Stability Toward Biomolecules and Bis-NHC Formation in Cell Culture Medium

Complexes **1** and **2** were dissolved in DMF and subsequently diluted with DMEM. Two preparations were generated: one containing fetal calf serum (FCS; corresponding to standard MTT assay conditions for 72 h) and one without FCS. The solutions were preincubated at 37 °C for 48 h in a ThermoMixer C incubator (Eppendorf) under constant shaking. Following preincubation, the samples were diluted with FCS-containing medium to the desired final test concentrations and immediately added to A549 lung cancer cells. Cells were incubated for an additional 24 h under standard culture conditions. Cell viability was determined by quantification of the remaining biomass of viable cells using the MTT assay as described above, and data were analyzed accordingly.

## Supplementary Material



## Notes

General materials and methods, synthetic procedures, NMR and ESI-MS spectra of the newly synthesized compounds, ^1^H NMR stability studies and corresponding ESI-MS spectra, crystallographic data, stability toward biomolecules, and cytotoxicity of bis-NHC complexes (PDF)
