# Supplementary material for: Tetrazolylidene-Stabilized Gold(I) Complexes: Synthesis and Evaluation of Anticancer Activity In Vitro
Source: Inorg Chem. 2026 Apr 15;65(16):8915–27. doi: 10.1021/acs.inorgchem.6c00049 (PMC13126643; doi:10.1021/acs.inorgchem.6c00049)
Supplement: Supplementary file 1 [file ic6c00049_si_001.pdf]

Supporting Information (SI)

Tetrazolylidene-Stabilized Gold(I) Complexes: Synthesis and Evaluation of Anticancer Activity *In Vitro*

Simon Stifel[a],[c], Claudia Schmidt[b], Leon F. Richter[a],[c], Alexander Pöthig[c], Angela Casini*[b] and Fritz E. Kühn*[a],[c]

[a] Technical University of Munich, School of Natural Sciences, Department of Chemistry, Molecular Catalysis, Lichtenbergstr. 4, 85748 Garching bei München, Germany.
E-Mail: fritz.kuehn@ch.tum.de
[b] Technical University of Munich, School of Natural Sciences, Department of Chemistry, Chair of Medicinal and Bioinorganic Chemistry, Lichtenbergstr. 4, 85748 Garching bei München, Germany.
E-Mail: angela.casini@tum.de
[c] Technical University of Munich, School of Natural Sciences, Catalysis Research Center (CRC), Ernst-Otto-Fischer Str. 1, 85748 Garching bei München, Germany.
E-Mail: alexander.poethig@tum.de

*Corresponding Authors.

Table of contents

1 General 6

2 Synthetic Procedures 8

2.1 General Procedures for the Synthesis of 1,3-Diaryl substituted Ligand Precursors $L_{1–4}$ 8

2.1.1 Synthesis of *N*-2-Diarylhydrazine-1-carbothioamides $A_{1–4}$ 8

2.1.1.1 *N*-2-Diphenylhydrazinecarbothioamide A_1 8

2.1.1.2 *N*-(*p*-Tolyl)-2-phenylhydrazinecarbothioamide A_2 9

2.1.1.3 *N*-2-Dimesitylhydrazinecarbothioamide A_3 9

2.1.1.4 *N*-(2,6-diisopropylphenyl)-2-phenylhydrazinecarbothioamide A_4 9

2.1.2 Synthesis of 5-Aryl-3-aryl-[1,2,3,4]-oxatriazolium Betaines $B_{1–4}$ 10

2.1.2.1 5-Anilino-3-phenyl-[1,2,3,4]-oxatriazolium Betaine B_1 10

2.1.2.2 5-(4-Methyl-anilino)-3-phenyl-[1,2,3,4]-oxatriazolium Betaine B_2 10

2.1.2.3 5-Mesityl-3-mesityl-[1,2,3,4]-oxatriazolium Betaine B_3 11

2.1.2.4 *5*-(2,6-Diisopropylphenyl)-3-phenyl-[1,2,3,4]-oxatriazolium Betaine B_4 ... 11

2.1.3 Synthesis of 1,3-Diaryltetrazolium-5-olates $C_{1–4}$ 11

2.1.3.1 2,4-Diphenyl-1*H*-tetrazol-2-ium-5-olate C_1 12

2.1.3.2 3-Phenyl-1-(*p*-tolyl)-1*H*-tetrazole-3-ium-5-olate C_2 12

2.1.3.3 1,3-Dimesityl-1*H*-tetrazol-3-ium-5-olate C_3 12

2.1.3.4 5-(2,6-Diisopropylphenyl)-3-phenyl-1*H*-tetrazol-3-ium-5-olate C_4 12

2.1.4 Synthesis of 1,3-Diaryltetrazolium-5-thiolates $D_{1–4}$ 13

2.1.4.1 1,3-Diphenyl-tetrazolium-5-thiolate D_1 13

2.1.4.2 3-Phenyl-1-(*p*-tolyl)-1*H*-tetrazole-3-ium-5-thiolate D_2 13

2.1.4.3 1,3-Dimesityl-1*H*-tetrazol-3-ium-5-thiolate D_3 14

2.1.4.4 3-Phenyl-1-(2,6-diisopropylphenyl)-1*H*-tetrazole-3-ium-5-thiolate D_4 14

2.1.5 Synthesis of 1,3-Diaryltetrazolium Salts $L_{1–4}$ 14

2.1.5.1 1,3-Diphenyl-1*H*-tetrazole-3-ium Hexafluorophosphate L_1 15

2.1.5.2 3-Phenyl-1-(*p*-tolyl)-1*H*-tetrazole-3-ium Hexafluorophosphate L_2 15

2.1.5.3 1,3-Dimesityl-1*H*-tetrazolium Hexafluorophosphate L_3 15

2.1.5.4 3-Phenyl-1-(2,6-diisopropylphenyl)-1*H*-tetrazole-3-ium Tetrafluoroborate L_4 16

2.2 General Procedure for the Synthesis of 1-Alkyl-3-aryl Substituted Ligand Precursors $L_{5–6}$ 16

2.2.1 Synthesis of Aryldiazonium Tetrafluoroborate Salts $A_{5–6}$ 16

2.2.1.1 Phenyldiazonium Tetrafluoroborate A_5 17

2.2.1.2 4-Methylbenzenediazonium Tetrafluoroborate A_6 17

2.2.2 Synthesis of 2-Aryltetrazole $B_{5–6}$ 17

2.2.2.1 2-Phenyl-2*H*-tetrazole B_5 18

2.2.2.2 2-(*p*-Tolyl)-2*H*-tetrazole B_6 18

2.2.3 Synthesis of 1-Methyl-3-aryltetrazolium Salts $L_{5–6}$ 18

2.2.3.1 1-Methyl-3-phenyl-2*H*-tetrazol-4-ium Tetrafluoroborate L_5 19

2.2.3.2 1-Methyl-3-tolyl-2*H*-tetrazol-4-ium Tetrafluoroborate L_6 19

2.3 Procedure for the Synthesis of 1,3-Dialkyl Substituted Ligand Precursor L_7 20

2.3.1 Synthesis of 1*H*-Tetrazole A_7 20

2.3.2 Synthesis of 2-Isopropyl-2*H*-tetrazole B_7 20

2.3.3 Synthesis of 1-*t*-Butyl-3-isopropyl-2*H*-tetrazolium Hexafluorophosphate L_7 21

2.4 General Procedure for the Synthesis of 1,3-Disubstituted-tetrazolylidene Gold(I) Chloride Complexes 1–7. 22

2.4.1 1,3-Diphenyl-tetrazolylidene Gold(I) Chloride 1 22

2.4.2 1-(*p*-Tolyl)-3-phenyl-tetrazolylidene Gold(I) Chloride 2 23

2.4.3 1,3-Dimesityl-tetrazolylidene Gold(I) Chloride 3 23

2.4.4 1-(2,6-Diisopropylphenyl)-3-phenyl-tetrazolylidene Gold(I) Chloride 4 24

2.4.5 1-Methyl-3-phenyl-tetrazolylidene Gold(I) Chloride 5 24

2.4.6 1-Methyl-3-*p*-tolyl-tetrazolylidene Gold(I) Chloride 6 25

2.4.7 1-*t*-Butyl-3-isopropyl-tetrazolylidene Gold(I) Chloride 7 25

2.5 General Procedure for the Synthesis of 1,3-Disubstituted-tetrazolylidene Gold(I) Bis-NHC Complexes B1–B2. 26

2.5.1 Bis-(1,3-diphenyl-tetrazolylidene) Gold(I) Hexafluorophosphate B1 26

2.5.2 Bis-(1-(*p*-tolyl)-3-phenyl-tetrazolylidene) Gold(I) Hexafluorophosphate B2 27

3 Analytic Data 28

3.1 *N*-2-Diphenylhydrazinecarbothioamide A_1 28

3.2 *N*-(*p*-Polyl)-2-phenylhydrazinecarbothioamide A_2 29

3.3 *N*-2-Dimesitylhydrazinecarbothioamide A_3 30

3.4 *N*-(2,6-Diisopropylphenyl)-2-phenylhydrazinecarbothioamide A_4 31

3.5 5-Anilino-3-phenyl-[1,2,3,4]-oxatriazolium betaine B_1 32

3.6 5-(4-Methyl-anilino)-3-phenyl-[1,2,3,4]-oxatriazolium Betaine B_2 33

3.7 5-Mesityl-3-mesityl-[1,2,3,4]-oxatriazolium Betaine B_3 34

3.8 5-(2,6-Diisopropylphenyl)-3-phenyl-[1,2,3,4]-oxatriazolium Betaine B_4 35

3.9 2,4-Diphenyl-1*H*-tetrazol-2-ium-5-olate C_1 36

3.10 3-Phenyl-1-(*p*-tolyl)-1*H*-tetrazole-3-ium-5-olate C_2 37

3.11 1,3-Dimesityl-1*H*-tetrazol-3-ium-5-olate C_3 38

3.12 5-(2,6-Diisopropylphenyl)-3-phenyl-1*H*-tetrazol-3-ium-5-olate C_4 39

3.13 1,3-Diphenyl-tetrazolium-5-thiolate D_1 40

3.14 3-Phenyl-1-(*p*-tolyl)-1*H*-tetrazole-3-ium-5-thiolate D_2 41

3.15 1,3-Dimesityl-1*H*-tetrazol-3-ium-5-thiolate D_3 42

3.16 3-Phenyl-1-(2,6-diisopropylphenyl)-1*H*-tetrazole-3-ium-5-thiolate D_4 43

3.17 1,3-Diphenyl-1*H*-tetrazole-3-ium Hexafluorophosphate L_1 44

3.18 3-Phenyl-1-(*p*-tolyl)-1*H*-tetrazole-3-ium Hexafluorophosphate L_2 46

3.19 1,3-Dimesityl-1*H*-tetrazolium Hexafluorophosphate L_3 48

3.20 3-Phenyl-1-(2,6-diisopropylphenyl)-1*H*-tetrazole-3-ium Tetrafluoroborate L_4 50

3.21 Phenylbenzenediazonium Tetrafluoroborate A_5 52

3.22 4-Methylbenzenediazonium Tetrafluoroborate A_6 53

3.23 2-Phenyl-2*H*-tetrazole B_5 54

3.24 2-(*p*-Tolyl)-2*H*-tetrazole B_6 55

3.25 1-Methyl-3-phenyl-2*H*-tetrazol-4-ium Tetrafluoroborate L_5 56

3.26 1-Methyl-3-tolyl-2*H*-tetrazol-4-ium Tetrafluoroborate L_6 58

3.27 1*H*-Tetrazole A_7 60

3.28 2-Isopropyl-2*H*-tetrazole B_7 61

3.29 1-*t*-Butyl-3-isopropyl-2*H*-tetrazolium Hexafluorophosphate L_7 62

3.30 1,3-Diphenyl-tetrazolylidene Gold(I) Chloride 1 64

3.31 1-(*p*-Tolyl)-3-phenyl-tetrazolylidene Gold(I) Chloride 2 66

3.32 1,3-Dimesityl-tetrazolylidene Gold(I) Chloride 3 70

3.33 1-(2,6-Diisopropylphenyl)-3-phenyl-tetrazolylidene Gold(I) Chloride 4 73

3.34 1-Methyl-3-phenyl-tetrazolylidene Gold(I) Chloride 5 75

3.35 1-Methyl-3-*p*-tolyl-tetrazolylidene Gold(I) Chloride 6 78

3.36 1-*t*-Butyl-3-isopropyl-tetrazolylidene Gold(I) Chloride 7 82

3.37 Bis-(1,3-diphenyl-tetrazoylylidene) Gold(I) Hexafluorophosphate B1 86

3.38 Bis-(1-(*p*-tolyl)-3-phenyl-tetrazolylidene) Gold(I) Hexafluorophosphate B2 88

4 Stability Studies 90

4.1 Analysis of the Stability of Compounds 1-7 against *L*-Cysteine and GSH 95

5 Crystallographic Data 113

6 Stability Towards Biomolecules and Bis-NHC Formation in Cell Culture Medium 116

1 General

Unless otherwise noted, all reactions were conducted without taking special precautions to exclude air and water. Solvents were distilled prior to use. Unless otherwise noted, chemical reagents and solvents were purchased from commercial suppliers (Sigma-Aldrich, TCI, Acros, Fisher Scientific) and used as received. Anhydrous solvents were obtained water- and oxygen-free from a *M. Braun* SPS purification system and stored over molecular sieves 3 Å. Column chromatography was carried out using silica gel 60 (*Acros*, 0.060-0.200 mm). For thin layer chromatography TLC silica gel 60 F_{254} was used. The spots were visualized with UV light (254 nm). ^{1}H and $^{13}C\{^{1}H\}$ NMR spectra were recorded on a *Bruker* AV400-US or a *Bruker* AV 500 Cryo spectrometer. All ^{1}H and $^{13}C\{^{1}H\}$ chemical shifts are reported in parts per million [ppm] and were referenced to the residual signal of the deuterated solvents (MeCN-d_3, ≥ 99.8 atom%; (DMSO-d_6, ≥ 99.8 atom). As for correlation of the signals and their multiplicities, the following abbreviations were used: s – singlet, d – doublet, t – triplet, q – quartet, qu – quintet, m – multiplet. The stated coupling constants *J* are denoted as the average of the experimentally found values and are given in hertz [Hz]. Electrospray ionisation-mass spectrometry (ESI-MS) data were acquired on a *Thermo Fisher* Ultimate 3000 using acetonitrile as eluent additive. The elemental analyses were carried out on a Vario EL from the company *Elementar* at the Catalysis Research Center of the *Technischen Universität München* in the microanalytical laboratory. SC-XRD Data were collected on a *Bruker* D8 Venture single crystal X-ray diffractometer equipped with a CMOS detector (*Bruker* Photon-100), an IMS microfocus source with MoK_{α} radiation (λ = 0.71073 Å) and a Helios optic using the APEX4 software package. Measurements were performed on single crystals coated with perfluorinated ether. The crystals were fixed on top of a Kapton micro sampler and frozen under a stream of cold nitrogen. A matrix scan was used to determine the initial lattice parameters. Reflections were corrected for Lorentz and polarisation effects, scan speed, and background using SAINT. Absorption corrections including odd and even ordered spherical harmonics were performed using SADABS. Space group assignments were based upon systematic absences, E statistics, and successful refinement of the structures. The structures were dissolved using SHELXT with the aid of successive difference Fourier maps and were refined against all data using SHELXL in conjunction with SHELXLE. Hydrogen atoms were placed in calculated positions and refined using a riding model, with methylene, aromatic, and other C–H distances of 0.99 Å, 0.95 Å and 1.00 Å, respectively, and $U_{iso}(H) = 1.2\ U_{eq(C)}$. Non-hydrogen atoms were refined with anisotropic displacement parameters. Full-matrix least-squares refinements were performed by minimizing $\sum w(F_c{}^2 - F_o{}^2)^2$ with the SHELXL weighting scheme. Neutral atom scattering factors for all

atoms and anomalous dispersion corrections for the non-hydrogen atoms were taken from International Tables for Crystallography. Images of the crystal structures were generated with Ortep3.

2 Synthetic Procedures

2.1 General Procedures for the Synthesis of 1,3-Diaryl substituted Ligand Precursors $\mathbf{L}_{1-4}$

N-2-diaryl-hydrazinecarbothioamides ($\mathbf{A}_{1-4}$), 5-arylimino-3-aryl-[1,2,3,4]-oxatriazolium betaines ($\mathbf{B}_{1-4}$), 1,3-diaryl-1,2,3,4-tetrazolium-5-olates ($\mathbf{C}_{1-4}$), 1,3-diaryl-1,2,3,4-tetrazolium-5-thiolates($\mathbf{D}_{1-4}$) and 1,3-diaryl-tetrazolium salts ($\mathbf{L}_{1-4}$) were synthesized according to modified literature procedures.[1] The identity and purity (>95%) of all biologically studied compounds were confirmed with elemental analysis and NMR spectroscopy.

2.1.1 Synthesis of *N*-2-Diarylhydrazine-1-carbothioamides $\mathbf{A}_{1-4}$

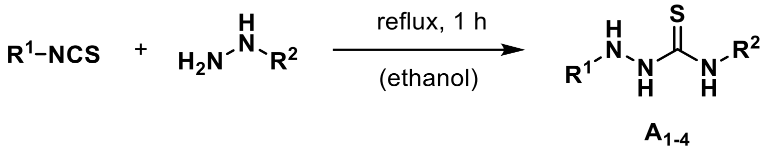

Scheme S1: General synthesis of 1,3-diaryl substituted ligand precursors $\mathbf{A}_{1-4}$.

In a pressure tube, 1.00 eq. arylhydrazine is dissolved in either 25 mL toluene or diethylether and 1.00 eq. 1-isothiocyanato-4-aryl is added slowly. If the hydrochloride salt of the arylhydrazine is used, 2.00 eq. triethylamine are additionally added. The reaction mixture is stirred for 1 hour under reflux. After cooling to 0 °C, the precipitate is filtered and washed with cold toluene (1 × 20 mL) and *n*-pentane (3 × 20 mL). The crude residue is recrystallized from ethanol to yield the corresponding *N*-(aryl)-2-arylhydrazinecarbothioamide $\mathbf{A}_{1-4}$.

2.1.1.1 *N*-2-Diphenylhydrazinecarbothioamide $\mathbf{A}_1$

Compound $\mathbf{A}_1$ is obtained as a white solid with a yield of 88%.

^{1}H NMR (400 MHz, DMSO-d_6, 298 K): δ [ppm] = 9.82 (s, 1H, SCNH), 9.70 (s, 1H, NNH), 8.06 (s, 1H, NNH), 7.53 (t, ^3J = 7.3 Hz, 2H, $H_{o\text{–}phenyl}$), 7.29 (t, ^3J = 7.8 Hz, 2H, $H_{m\text{–}phenyl}$), 7.23 (t, ^3J = 8.0 Hz, 2H, $H_{m\text{–}phenyl}$), 7.12 (t, ^3J = 7.4 Hz, 1H, $H_{p\text{–}phenyl}$), 6.82 (t, ^3J = 7.3 Hz, 1H, $H_{p\text{–}phenyl}$), 6.76–6.67 (m, 2H, $H_{o\text{–}phenyl}$).

2.1.1.2 *N*-(*p*-Tolyl)-2-phenylhydrazinecarbothioamide A2

Compound **A2** is obtained as a white solid with a yield of 76%.

^{1}H NMR (400 MHz, DMSO-d_6, 298 K): δ [ppm] = 9.71 (s, 1H, $H_{NHN\textit{H}CS}$), 9.62 (s, 1H, $H_{N\textit{H}NHCS}$), 8.02 (s, 1H, $H_{SCN\textit{H}C}$), 7.37 (d, ^3J = 8.3 Hz, 2H, $H_{o\text{–phenyl}}$), 7.22 (t, ^3J = 7.8 Hz, 2H, $H_{m\text{–phenyl}}$), 7.09 (d, ^3J = 8.6 Hz, 2H, $H_{o\text{–tolyl}}$), 6.81 (t, ^3J = 6.9 Hz, 1H, $H_{p\text{–phenyl}}$), 6.76 (d, ^3J = 7.8 Hz, 2H, $H_{m\text{–tolyl}}$), 2.26 (s, 3H, CH_3).

2.1.1.3 *N*-2-Dimesitylhydrazinecarbothioamide A3

Compound **A3** is obtained as white solid with a yield of 63%.

^{1}H NMR (400 MHz, DMSO-d_6, 294 K): δ [ppm] = 9.33 (s, 1H, $H_{SCN\textit{H}C}$), 9.22 (s, 1H, $H_{NHN\textit{H}CS}$), 6.85 (s, 2H, CH), 6.77 (s, 2H, CH), 6.73 (s, 1H, $H_{N\textit{H}NHCS}$), 2.30 (s, 6H, $CH_{3,\,o\text{–mes}}$), 2.23 (s, 3H, $CH_{3,\,p\text{–mes}}$), 2.18 (m, 9H, $CH_{3,\,o\text{–},p\text{–mes}}$).

^{13}C NMR (101 MHz, DMSO-d_6, 303 K): δ [ppm] = 179.8 (s, $C_{N\textit{C}SN}$), 140.6 (s, C_{ar}), 136.2 (s, C_{ar}), 135.3 (s, C_{ar}), 134.9 (s, C_{ar}), 131.4 (s, C_{ar}), 129.3 (s, C_{ar}), 128.1 (s, C_{ar}), 20.6 (s, CH_3), 20.2 (s, CH_3), 18.6 (s, CH_3), 18.4 (s, CH_3).

EA (%): calcd. C 69.68, H 7.69, N 12.83, S 9.79; found C 69.65, H 7.73, N 12.70, S 9.26.

R_f = 0.11 (DCM) [UV].

2.1.1.4 *N*-(2,6-diisopropylphenyl)-2-phenylhydrazinecarbothioamide A4

Compound **A4** is obtained as yellow solid with a yield of 85%.

^{1}H NMR (400 MHz, DMSO-d_6, 294 K): δ [ppm] = 9.52 (s, 1H, $H_{SCN\textit{H}C}$), 9.37 (s, 1H, $H_{NHN\textit{H}CS}$), 8.09 (s, 1H, $H_{SCN\textit{H}C}$), 7.22 (q, ^3J = 8.2 Hz, 3H, $CH_{m\text{–},p\text{–Dipp}}$), 7.10 (s, 1H, CH), 7.08 (s, 1H, CH), 3.04 (q, ^3J = 7.1 Hz, 2H, C*H*CH_3), 1.20 (d, ^3J = 6.9 Hz, 6H, CH_3), 1.00 (d, ^3J = 6.9 Hz, 6H, CH_3).

2.1.2 Synthesis of 5-Aryl-3-aryl-[1,2,3,4]-oxatriazolium Betaines $B_{1–4}$

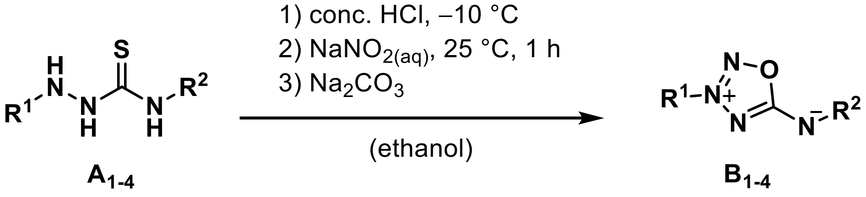

Scheme S2: General synthesis of 5-aryl-3-aryl-[1,2,3,4]-oxatriazolium betaines $\mathbf{B_{1–4}}$.

In a 50 mL round-bottom flask, 1.00 eq. of *N*-(aryl)-2-arylhydrazinecarbothioamide ($\mathbf{A_{1–4}}$) is suspended in 13 mL ethanol and cooled to –10 °C. The suspension is acidified with 1 mL concentrated aqueous HCl to pH 2, and 1.50 eq. of sodium nitrite solution (1 M) is added slowly. The mixture is stirred at 25 °C for 1 h, then filtered. The filtrate is alkalized to pH 11 using sodium carbonate and cooled overnight at 4 °C. The resulting precipitate is filtered, washed with water (3 × 15 mL), and dried *in vacuo* to obtain the corresponding aryl-(3-aryl-1,2,3,4-oxatriazolium betaine $\mathbf{B_{1–4}}$.

2.1.2.1 5-Anilino-3-phenyl-[1,2,3,4]-oxatriazolium Betaine B_1

Compound $\mathbf{B_1}$ is obtained as a red solid with a yield of 61%.

^{1}H NMR (400 MHz, DMSO-d_6, 298 K): δ [ppm] = 8.14–8.11 (m, 2H, $H_{o\text{–phenyl}}$), 7.82 (t, ^3J = 7.5 Hz, 1H, $H_{p\text{–phenyl}}$), 7.75 (t, ^3J = 7.64 Hz, 2H, $H_{m\text{–phenyl}}$), 7.31 (t, ^3J = 7.6 Hz, 1H, $H_{m\text{–phenyl}}$), 7.25–7.24 (m, 2H, $H_{o\text{–phenyl}}$), 7.02 (t, ^3J = 7.3 Hz, 1H, $H_{p\text{–phenyl}}$).

2.1.2.2 5-(4-Methyl-anilino)-3-phenyl-[1,2,3,4]-oxatriazolium Betaine B_2

Compound $\mathbf{B_2}$ is obtained as a red solid with a yield of 91%.

^{1}H NMR (400 MHz, DMSO-d_6, 299 K): δ [ppm] = 8.11 (d, ^3J = 7.8 Hz, 2H, $H_{o\text{–phenyl}}$), 7.82 (t, 1H, ^3J = 7.4 Hz, $H_{p\text{–phenyl}}$), 7.75 (t, ^3J = 7.8 Hz, 2H, $H_{m\text{–phenyl}}$), 7.14–7.10 (m, 4H, $H_{o\text{–}, m\text{–tolyl}}$), 2.27 (s, 3H, CH_3).

R_f = 0.52 (DCM/MeOH = 35:1) [UV].

2.1.2.3 5-Mesityl-3-mesityl-[1,2,3,4]-oxatriazolium Betaine $\mathbf{B_3}$

Compound $\mathbf{B_3}$ is obtained as an orange solid with a yield of 82%.

^{1}H NMR (400 MHz, DMSO-d_6, 295 K): δ [ppm] = 7.21 (s, 2H, CH), 6.81 (s, 2H, CH), 2.34 (s, 3H, $CH_{3,\,p\text{–mes}}$), 2.18 (s, 9H, $CH_{3,\,o\text{–},\,p\text{–mes}}$), 2.07 (s, 6H, $CH_{3,\,p\text{–mes}}$).

^{13}C NMR (101 MHz, DMSO-d_6, 294 K): δ [ppm] = 142.7 (s, C_{NCON}), 133.3 (s, C_{ar}), 131.2 (s, C_{ar}), 130.7 (s, C_{ar}), 130.3 (s, C_{ar}), 129.6 (s, C_{ar}), 128.4 (s, C_{ar}), 128.3 (s, C_{ar}), 20.8 (s, CH_3), 20.4 (s, CH_3), 17.9 (s, CH_3), 16.1 (s, CH_3).

EA (%): calcd. C 70.78, H 6.88, N 17.38, O 4.96; found C 69.39, H 6.97, N 16.63, S 1.97.

R_f = 0.36 (DCM) [UV].

2.1.2.4 5-(2,6-Diisopropylphenyl)-3-phenyl-[1,2,3,4]-oxatriazolium Betaine $\mathbf{B_4}$

Compound $\mathbf{B_4}$ is obtained as a red oil with a yield of 81%.

^{1}H NMR (400 MHz, DMSO-d_6, 294 K): δ [ppm] = 8.09 (d, ^3J = 8.0 Hz, 3H, $CH_{o\text{–phenyl}}$), 7.83–7.78 (m, 1H, $CH_{p\text{–Dipp}}$), 7.74–7.69 (m, 2H, $CH_{m\text{–phenyl}}$), 7.10–7.08 (m, 2H, $CH_{m\text{–Dipp}}$), 7.03–6.99 (m, 1H, $CH_{p\text{–Dipp}}$), 3.08 (q, ^3J = 6.8 Hz, 2H, C*H*CH_3), 1.11 (d, ^3J = 6.9 Hz, 12H, CH_3).

2.1.3 Synthesis of 1,3-Diaryltetrazolium-5-olates $\mathbf{C_{1–4}}$

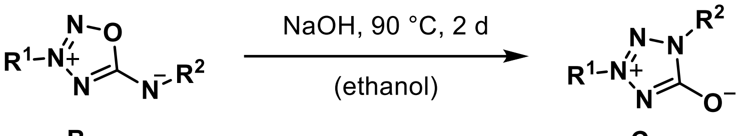

$\mathbf{B_{1\text{-}4}}$ $\mathbf{C_{1\text{-}4}}$

Scheme S3: General synthesis of 1,3-diaryltetrazolium-5-olates $\mathbf{C_{1–4}}$.

In a 100 mL round-bottom flask, 1.00 eq. of the oxatriazolium intermediate ($\mathbf{B_{1–4}}$) is dissolved in 23 mL ethanol and alkalized to pH 11 using a few drops of aqueous NaOH (2 M). The mixture is stirred at 80 °C for 2.5 h, then cooled to 25 °C and diluted with 90 mL water. The resulting precipitate is filtered, washed with water (3 × 10 mL), and dried *in vacuo* to yield 1,3-diaryltetrazolium-5-olate $\mathbf{C_{1–4}}$.

2.1.3.1 2,4-Diphenyl-1*H*-tetrazol-2-ium-5-olate C_1

Compound $\mathbf{C_1}$ is obtained as a beige solid with a yield of 70%.

^{1}H NMR (400 MHz, DMSO-d_6, 298 K): δ [ppm] = 8.14–8.12 (m, 2H, $H_{o\text{–phenyl}}$), 8.07–8.15 (m, 2H, $H_{o\text{–phenyl}}$), 7.72–7.69 (m, 3H, $H_{m\text{–}, p\text{–phenyl}}$), 7.63 (t, ^3J = 7.8 Hz, 2H, $H_{m\text{–phenyl}}$), 7.52 (t, ^3J = 7.5 Hz, 1H, $H_{p\text{–phenyl}}$).

2.1.3.2 3-Phenyl-1-(*p*-tolyl)-1*H*-tetrazole-3-ium-5-olate C_2

Compound $\mathbf{C_2}$ is obtained as a beige solid with a yield of 89%.

^{1}H NMR (400 MHz, DMSO-d_6, 299 K): δ [ppm] = 8.13–8.11 (m, 2H, $H_{o\text{–phenyl}}$), 7.94 (d, ^3J = 8.4 Hz, 2H, $H_{o\text{–tolyl}}$), 7.72–7.69 (m, 3H, $H_{m\text{–},p\text{–phenyl}}$), 7.43 (d, ^3J = 8.6 Hz, 2H, $H_{m\text{–tolyl}}$), 2.39 (s, 3H, CH_3).

R_f = 0.30 (DCM/MeOH = 35:1) [UV].

2.1.3.3 1,3-Dimesityl-1*H*-tetrazol-3-ium-5-olate C_3

Compound $\mathbf{C_3}$ is obtained as an orange solid with a yield of 95%.

^{1}H NMR (400 MHz, DMSO-d_6, 294 K): δ [ppm] = 7.18 (s, 2H, CH), 7.14 (s, 2H, CH), 2.35 (s, 3H, $CH_{3,p\text{–mes}}$), 2.33 (s, 3H, $CH_{3,p\text{–mes}}$), 2.14 (s, 6H, $CH_{3,o\text{–mes}}$), 2.11 (s, 6H, $CH_{3,o\text{–mes}}$).

^{13}C NMR (101 MHz, DMSO-d_6, 294 K): δ [ppm] = 159.8 (s, C_{NCON}), 141.7 (s, C_{ar}), 140.6 (s, C_{ar}), 135.5 (s, C_{ar}), 133.9 (s, C_{ar}), 133.6 (s, C_{ar}), 129.4 (s, C_{ar}), 129.2 (s, C_{ar}), 127.9 (s, C_{ar}), 20.8 (s, CH_3), 20.7 (s, CH_3), 17.1 (s, CH_3), 16.3 (s, CH_3).

EA (%): calcd. C 70.78, H 6.88, N 17.38, O 4.96; found C 70.66, H 6.85, N 17.21.

R_f = 0.05 (DCM); 0.79 (EtOAc) [UV].

2.1.3.4 5-(2,6-Diisopropylphenyl)-3-phenyl-1*H*-tetrazol-3-ium-5-olate C_4

Compound $\mathbf{C_4}$ is obtained as red solid with a yield of 80%.

^{1}H NMR (400 MHz, $CDCl_3$, 294 K): δ [ppm] = 8.15–8.13 (m, 2H, $CH_{o\text{–phenyl}}$), 7.61–7.57 (m, 3H, $CH_{p\text{–Dipp}, m\text{–phenyl}}$), 7.52 (t, ^3J = 7.8 Hz, 1H, $CH_{p\text{–phenyl}}$), 7.34 (s, 1H, CH), 7.32 (s, 1H, CH), 2.67 (q, ^3J = 6.8 Hz, 2H, C*H*CH_3), 1.27 (d, ^3J = 6.9 Hz, 6H, CH_3), 1.17 (d, ^3J = 6.9 Hz, 6H, CH_3).

2.1.4 Synthesis of 1,3-Diaryltetrazolium-5-thiolates $\mathbf{D_{1–4}}$

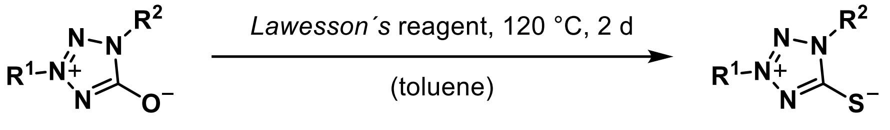

Scheme S4: General synthesis of 1,3-diaryltetrazolium-5-thiolates $\mathbf{D_{1–4}}$.

In a 50 mL round-bottom flask, 1.00 eq. of the tetrazolium-5-olate ($\mathbf{C_{1–4}}$) is suspended in 20 mL toluene. After the addition of 1.00 eq. *Lawesson's* reagent, the mixture is stirred at 120 °C for 3 d under reflux. The solvent is then removed *in vacuo* and the crude product is purified by silica gel column chromatography (DCM → DCM/MeOH = 30:1) to obtain 1,3-dimesitylte-trazolium-5-thiolate $\mathbf{D_{1–4}}$.

2.1.4.1 1,3-Diphenyl-tetrazolium-5-thiolate $\mathbf{D_1}$

Compound $\mathbf{D_1}$ is purified by recrystallization and is obtained as a yellow solid with a yield of 56%.

^{1}H NMR (400 MHz, DMSO-d_6, 298 K): δ [ppm] = 8.15–8.12 (m, 2H, $H_{o\text{–phenyl}}$), 8.03–8.00 (m, 2H, $H_{o\text{–phenyl}}$), 7.74–7.72 (m, 3H, $H_{m\text{–},p\text{–phenyl}}$), 7.69–7.64 (m, 3H, $H_{m\text{–},p\text{–phenyl}}$).

2.1.4.2 3-Phenyl-1-(*p*-tolyl)-1*H*-tetrazole-3-ium-5-thiolate $\mathbf{D_2}$

Compound $\mathbf{D_2}$ is purified by column chromatography and is obtained as a white solid with a yield of 98%.

^{1}H NMR (400 MHz, DMSO-d_6, 299 K): δ [ppm] = 8.14–8.11 (m, 2H, $H_{o\text{–phenyl}}$), 7.89 (d, ^3J = 8.3 Hz, 2H, $H_{o\text{–tolyl}}$), 7.73–7.72 (m, 3H, $H_{m\text{–},p\text{–phenyl}}$), 7.46 (d, ^3J = 8.4 Hz, 2H, $H_{m\text{–tolyl}}$), 2.43 (s, 3H, CH_3).

R_f = 0.40 (DCM) [UV].

2.1.4.3 1,3-Dimesityl-1*H*-tetrazol-3-ium-5-thiolate D_3

Compound $\mathbf{D_3}$ is purified by recrystallisation and is obtained as a yellow solid with a yield of 70%.

^{1}H NMR (400 MHz, DMSO-d_6, 294 K): δ [ppm] = 7.24 (s, 2H, CH), 7.17 (s, 2H, CH), 2.37 (s, 3H, $CH_{3,\,p\text{–mes}}$), 2.35 (s, 3H, $CH_{3,\,p\text{–mes}}$), 2.14 (s, 6H, $CH_{3,\,o\text{–mes}}$), 2.07 (s, 6H, $CH_{3,\,o\text{–mes}}$).

^{13}C NMR (101 MHz, DMSO-d_6, 294 K): δ [ppm] = 175.3 (s, C_{NCON}), 142.4 (s, C_{ar}), 141.3 (s, C_{ar}), 135.3 (s, C_{ar}), 134.0 (s, C_{ar}), 132.3 (s, C_{ar}), 129.4 (s, C_{ar}), 129.0 (s, C_{ar}), 20.8 (s, CH_3), 20.8 (s, CH_3), 17.1 (s, CH_3), 16.3 (s, CH_3).

EA (%): calcd. C 67.42, H 6.55, N 16.55, S 9.47; found C 67.15, H 6.68, N 16.46, S 9.21.

R_f = 0.94 (EtOAc) [UV].

2.1.4.4 3-Phenyl-1-(2,6-diisopropylphenyl)-1*H*-tetrazole-3-ium-5-thiolate D_4

Compound $\mathbf{D_4}$ is purified by column chromatography and is obtained as a yellow solid with a yield of 27%.

^{1}H NMR (400 MHz, $CDCl_3$, 294 K): δ [ppm] = 8.19–8.17 (m, 2H, $CH_{o\text{–phenyl}}$), 7.65–7.62 (m, 3H, $CH_{p\text{–Dipp},\,m\text{–phenyl}}$), 7.59 (t, ^{3}J = 7.8 Hz, 1H, $CH_{p\text{–phenyl}}$), 7.38 (s, 1H, CH), 7.36 (s, 1H, CH), 2.54 (q, ^{3}J = 6.8 Hz, 2H, C*H*CH_3), 1.33 (d, ^{3}J = 6.7 Hz, 6H, CH_3), 1.14 (d, ^{3}J = 6.9 Hz, 6H, CH_3).

2.1.5 Synthesis of 1,3-Diaryltetrazolium Salts $L_{1–4}$

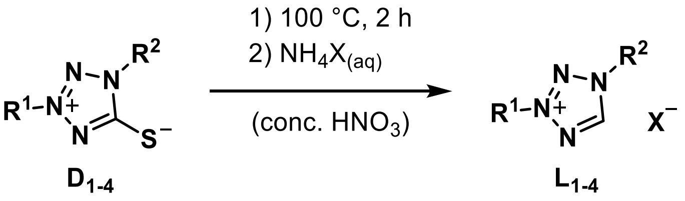

Scheme S5: General synthesis of 1,3-diaryltetrazolium salts $\mathbf{L_{1–4}}$.

In a 25 mL round-bottom flask, 1.00 eq. of the tetrazolium-5-thiolate ($\mathbf{D_{1–4}}$) is dissolved in 1 mL concentrated aqueous HNO_3 and stirred at 100 °C for 3 h under reflux. While it is still hot, 1.50 eq. of aqueous ammonium hexafluorophosphate or ammonium tetrafluoroborate is added. The reaction mixture is cooled overnight at 4 °C. The resulting precipitate is filtered, washed with water (3 × 2 mL) and a DCM/MeOH mixture (10:1, 3 × 4 mL), then dried *in vacuo* to yield the 1,3-dimesityltetrazolium salt $\mathbf{L_{1–4}}$.

2.1.5.1 1,3-Diphenyl-1*H*-tetrazole-3-ium Hexafluorophosphate $\mathbf{L_1}$

Compound $\mathbf{L_1}$ is obtained as a white solid with a yield of 83%.

^{1}H NMR (400 MHz, MeCN-d_3, 298 K): δ [ppm] = 10.13 (s, 1H, H_{tetr}), 8.30 (d, ^3J = 8.0 Hz, 2H, $H_{o\text{–phenyl}}$), 8.01 (d, ^3J = 7.3 Hz, 2H, $H_{o\text{–phenyl}}$), 7.89–7.80 (m, 6H, $H_{m\text{–}, p\text{–phenyl}}$).

^{13}C NMR (101 MHz, MeCN-d_3, 303 K):147.7 (s, C_{tetr}), 136.0 (s, C_{ar}), 135.1 (s, C_{ar}), 134.3 (s, C_{ar}), 133.1 (s, C_{ar}), 131.9 (d, C_{ar}), 123.5 (s, C_{ar}), 122.4 (s, C_{ar}).

EA (%): calcd. C 42.40, H 3.01, N 15.22, F 30.96, P 8.41; found C 42.66, H 2.70, N 15.32.

2.1.5.2 3-Phenyl-1-(*p*-tolyl)-1*H*-tetrazole-3-ium Hexafluorophosphate $\mathbf{L_2}$

Compound $\mathbf{L_2}$ is obtained as a white solid with a yield of 82%.

^{1}H NMR (400 MHz, MeCN-d_3, 300 K): δ [ppm] = 10.12 (s, 1H, H_{tetr}), 8.29 (d, ^3J = 7.3 Hz, 2H, $H_{o\text{–phenyl}}$), 7.90–7.79 (m, 5H, $H_{m\text{–}, p\text{–phenyl}, o\text{–tolyl}}$), 7.61 (d, ^3J = 8.7 Hz, 2H, $H_{m\text{–tolyl}}$), 2.52 (s, 3H, CH_3).

^{13}C NMR (101 MHz, MeCN-d_3, 303 K): δ [ppm] = 147.6 (s, C_{tetr}), 145.6 (s, C_{ar}), 136.1 (s, C_{ar}), 135.0 (s, C_{ar}), 132.3 (s, C_{ar}), 131.9 (s, C_{ar}), 130.7 (s, C_{ar}), 123.2 (s, C_{ar}), 122.4 (s, C_{ar}), 21.5 (s, CH_3).

ESI-MS (m/z): [$\mathbf{L_2}$ – PF_6]$^+$ calcd, 237.11; found, 237.12.

EA (%): calcd. C 43.99, H 3.43, N 14.66, F 29.82, P 8.10; found C 49.96, H 4.40, N 18.64.

2.1.5.3 1,3-Dimesityl-1*H*-tetrazolium Hexafluorophosphate $\mathbf{L_3}$

Compound $\mathbf{L_3}$ is obtained as a white solid with a yield of 61%.

^{1}H NMR (400 MHz, MeCN-d_3, 294 K): δ [ppm] = 10.12 (s, 1H, H_{tetr}), 7.28 (s, 4H, CH), 2.43 (d, 3H, $CH_{3, p\text{–mes}}$), 2.42 (s, 3H, $CH_{3, p\text{–mes}}$), 2.17 (s, 6H, $CH_{3, o\text{–mes}}$), 2.16 (s, 6H, $CH_{3, o\text{–mes}}$).

^{13}C NMR (101 MHz, DMSO-d_6, 294 K): δ [ppm] = 152.1 (s, C_{NCON}), 143.8 (s, C_{ar}), 143.3 (s, C_{ar}), 134.7 (s, C_{ar}), 134.5 (s, C_{ar}), 131.4 (s, C_{ar}), 130.2 (s, C_{ar}), 128.8 (s, C_{ar}), 128.0 (s, C_{ar}), 124.0 (s, C_{ar}), 20.9 (s, CH_3), 20.9 (s, CH_3), 17.3 (s, CH_3), 17.0 (s, CH_3).

EA (%): calcd. C 50.45, H 5.12, N 12.39, F 25.20, P 6.85; found C 50.61, H 4.84, N 12.59.

R_f = 0.10 (DCM); 0.66 (DCM/MeOH = 10:2) [UV].

2.1.5.4 3-Phenyl-1-(2,6-diisopropylphenyl)-1*H*-tetrazole-3-ium Tetrafluoroborate L_4

Compound $\mathbf{L_4}$ is obtained as an orange solid with a yield of 61%.

^{1}H NMR (400 MHz, MeCN-d_3, 294 K): δ [ppm] = 10.23 (s, 1H, H_{tetr}), 8.32 (d, ^{3}J = 8.0 Hz, 2H, $CH_{o\text{–phenyl}}$), 7.89–7.85 (m, 1H, $CH_{p\text{–phenyl}}$), 7.83–7.77 (m, 3H, $CH_{p\text{–Dipp},\ m\text{–phenyl}}$), 7.58 (s, 1H, CH), 7.56 (s, 1H, CH), 2.41 (q, ^{3}J = 6.8 Hz, 2H, C*H*CH_3), 1.23 (d, ^{3}J = 6.8 Hz, 6H, CH_3), 1.17 (d, ^{3}J = 6.8 Hz, 6H, CH_3).

^{13}C NMR (101 MHz, MeCN-d_3, 303 K): δ [ppm] = 151.7 (s, $C_{o\text{–Dipp}}$), 147.0 (s, C_{tetr}), 136.5 (s, C_{ar}), 135.2 (s, C_{ar}), 135.0 (s, C_{ar}), 131.2 (s, C_{ar}), 128.5 (s, C_{ar}), 126.3 (s, C_{ar}), 121.6 (s, C_{ar}), 29.4 (s, CH_3), 24.6 (s, CH), 23.6 (s, CH).

EA (%): calcd. C 57.89, H 5.12, N, 14.21, B, 2.74; F, 19.28; found C 59.03, H 5.87, N 15.58.

2.2 General Procedure for the Synthesis of 1-Alkyl-3-aryl Substituted Ligand Precursors $L_{5–6}$

2.2.1 Synthesis of Aryldiazonium Tetrafluoroborate Salts $A_{5–6}$

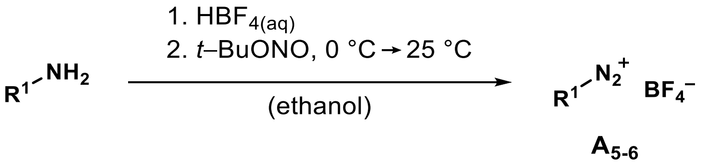

Scheme S6: General synthesis of aryldiazonium tetrafluoroborate salts $\mathbf{A_{5–6}}$.

In a round-bottom flask, 1.00 eq. of the respective arylamine is dissolved in ethanol (3 mL), and aqueous tetrafluoroboric acid (50 wt%, 4.00 eq.) is added slowly under stirring. The reaction mixture is cooled to 0 °C in an ice bath, and 4.00 eq. *t*-butyl nitrite are added dropwise. After stirring for 1 hour at room temperature (25 °C), the mixture is diluted with diethyl ether (30 mL). The resulting precipitate is collected by filtration, washed with cold diethyl ether (2 × 10 mL) until the yellow color disappears, and all volatiles are removed *in vacuo* to afford the corresponding aryldiazonium tetrafluoroborate $\mathbf{A_{5–6}}$.

2.2.1.1 Phenyldiazonium Tetrafluoroborate A_5

Compound $\mathbf{A_5}$ is obtained as a white solid with a yield of 75%.

^{1}H NMR (400 MHz, D_2O, 300 K): δ [ppm] = 8.58 (d, ^3J = 7.9 Hz, 2H, $H_{o\text{–phenyl}}$), 8.33–8.29 (m, 1H, $H_{m\text{–phenyl}}$), 8.00–7.96 (m, 2H, $H_{m\text{–phenyl}}$).

2.2.1.2 4-Methylbenzenediazonium Tetrafluoroborate A_6

Compound $\mathbf{A_6}$ is obtained as a white solid with a yield of 81%.

^{1}H NMR (400 MHz, D_2O, 300 K): δ [ppm] = 8.45 (d, ^3J = 8.8 Hz, 2H, $H_{o\text{–tolyl}}$), 7.80 (d, ^3J = 8.3 Hz, 2H, $H_{m\text{–tolyl}}$), 2.65 (s, 3H, CH_3).

2.2.2 Synthesis of 2-Aryltetrazole $B_{5–6}$

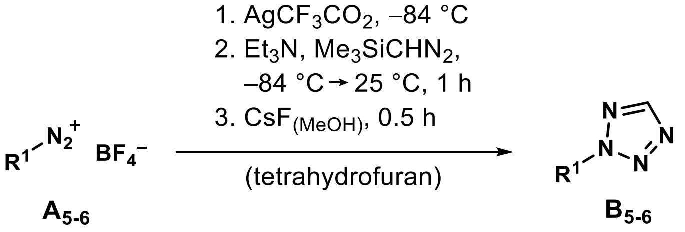

Scheme S7: General synthesis of 2-aryltetrazole $\mathbf{B_{5–6}}$.

Under an inert atmosphere, 1.00 eq. aryldiazonium tetrafluoroborate ($\mathbf{A_{5–6}}$) and 1.20 eq. silver trifluoromethanesulfonate are dissolved in anhydrous tetrahydrofuran (10 mL) and the solution is cooled to –84 °C. 1.50 eq. dry triethylamine is added dropwise, and the mixture is stirred for 10 min at this temperature. Then, 1.10 eq. trimethylsilyldiazomethane (2 M in *n*-hexane) is added dropwise, and the reaction mixture is stirred for an additional hour at –84 °C. The mixture is then allowed to warm gradually to 25 °C, followed by the addition of 2.00 eq. caesium fluoride dissolved in methanol (4.5 mL). After stirring for further 30 min, the reaction mixture is diluted with ethyl acetate (50 mL) and transferred to a separatory funnel. The organic layer is washed successively with 1 M aqueous HCl (10 mL) and brine (15 mL), dried over anhydrous magnesium sulfate, filtered, and concentrated under reduced pressure to afford a dark brown oil. The crude product is extracted with hot *n*-hexane (3 × 6 mL), and the combined extracts are concentrated *in vacuo*. Purification is achieved by column chromatography on silica gel ($\mathbf{B_5}$) or by sublimation ($\mathbf{B_6}$) to obtain the respective 2-aryl-2*H*-tetrazole.

2.2.2.1 2-Phenyl-2*H*-tetrazole $\mathbf{B_5}$

Compound $\mathbf{B_5}$ is obtained as a yellow oil with a yield of 30%.

^{1}H NMR (400 MHz, $CDCl_3$, 300 K): δ [ppm] = 8.66 (s, 1H, H_{tetr}), 8.15 (d, ^3J = 7.2 Hz, 2H, $H_{o\text{–}phenyl}$), 7.57 (t, ^3J = 7.5 Hz, 2H, $H_{m\text{–}phenyl}$), 7.51 (t, ^3J = 7.3 Hz, 1H, $H_{p\text{–}phenyl}$).

$\mathbf{R_f}$ = 0.70 (*n*-hexane /EtOAc = 7:3) [UV].

2.2.2.2 2-(*p*-Tolyl)-2*H*-tetrazole $\mathbf{B_6}$

Compound $\mathbf{B_6}$ is obtained as an orange solid with a yield of 44%.

^{1}H NMR (400 MHz, $CDCl_3$, 300 K): δ [ppm] = 8.63 (s, 1H, H_{tetr}), 8.02 (d, ^3J = 8.7 Hz, 2H, $H_{o\text{–}tolyl}$), 7.36 (d, ^3J = 8.6 Hz, 2H, $H_{m\text{–}tolyl}$), 2.45 (s, 3H, CH_3).

2.2.3 Synthesis of 1-Methyl-3-aryltetrazolium Salts $\mathbf{L_{5–6}}$

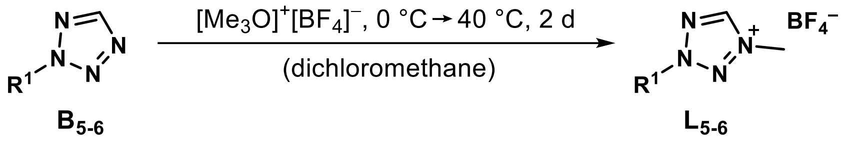

Scheme S8: General synthesis of 1-methyl-3-aryltetrazolium salts $\mathbf{L_{5–6}}$.

In a pressure tube under inert gas atmosphere, 1.00 eq. trimethyloxonium tetrafluoroborate are suspended in 4 mL dichloromethane and cooled to –40 °C. The cold suspension is slowly added to a solution of 1.00 eq. 2-aryl-2*H*-tetrazole ($\mathbf{B_{5–6}}$) in 4 mL dichloromethane which is cooled to the same temperature. The reaction mixture is allowed to reach 25 °C and is further stirred for 2 d at 45 °C reflux. After the reaction time is over, the reaction mixture is cooled overnight at – 34 °C for complete crystallization. Subsequently, the precipitate is filtered, washed with cold dichloromethane (1 × 3 mL) and dried *in vacuo* to obtain the respective 1-methyl-3-aryl-2*H*-tetrazol-4-ium tetrafluoroborate salt $\mathbf{L_{5–6}}$.

2.2.3.1 1-Methyl-3-phenyl-2*H*-tetrazol-4-ium Tetrafluoroborate L_5

Compound L_5 is obtained as a white solid with a yield of 43%.

^{1}H NMR (400 MHz, MeCN-d_3, 300 K): δ [ppm] = 9.66 (s, 1H, H_{tetr}), 8.16 (d, ^3J = 8.2 Hz, 2H, $H_{o\text{–phenyl}}$), 7.82–7.77 (m, 3H, $H_{m\text{–},p\text{–phenyl}}$), 4.46 (s, 3H, CH_3).

^{13}C NMR (101 MHz, MeCN-d_3, 300 K): δ [ppm] = 150.3 (s, C_{tetr}), 136.1 (s, C_{ar}), 134.6 (s, C_{ar}), 131.7 (s, C_{ar}), 122.2 (s, C_{ar}), 39.6 (s, CH_3).

ESI-MS (m/z): $[L_5 – BF_4]^+$ calcd, 161.08; found, 161.03.

EA (%): calcd. C 38.75, H 3.66, N 22.59, B 4.36, F 30.64; found C 38.52, H 3.53, N 22.26.

2.2.3.2 1-Methyl-3-tolyl-2*H*-tetrazol-4-ium Tetrafluoroborate L_6

Compound L_6 is obtained as a white solid with a yield of 63%.

^{1}H NMR (400 MHz, MeCN-d_3, 300 K): δ [ppm] = 9.62 (s, 1H, H_{tetr}), 8.04 (d, ^3J = 8.8 Hz, 2H, $H_{o\text{–tolyl}}$), 7.56 (d, ^3J = 8.6 Hz, 2H, $H_{m\text{–tolyl}}$), 4.44 (s, 3H, NCH_3), 2.50 (s, 3H, CCH_3).

^{13}C NMR (101 MHz, MeCN-d_3, 300 K): δ [ppm] = 150.1 (s, C_{tetr}), 146.0 (s, C_{ar}), 133.8 (s, C_{ar}), 132.1 (s, C_{ar}), 122.0 (s, C_{ar}), 39.5 (s, NCH_3), 21.5 (s, CCH_3).

ESI-MS (m/z): $[L_6 – BF_4]^+$ calcd, 175.10; found, 175.04.

EA (%): calcd. C 41.26, H 4.23, N 21.38, B 4.13, F 29.00; found C 40.58, H 3.70, N 20.43.

2.3 Procedure for the Synthesis of 1,3-Dialkyl Substituted Ligand Precursor L7

2.3.1 Synthesis of 1*H*-Tetrazole A7

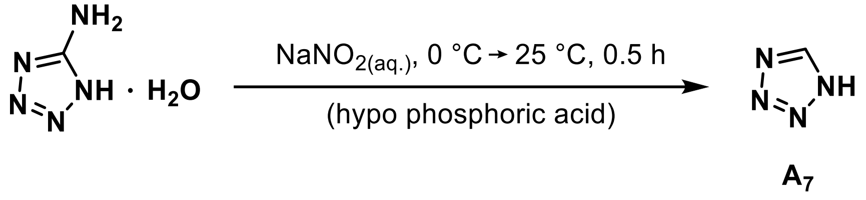

Scheme S9: Synthesis of 1*H*-tetrazole **A7.**

In a 50 mL round bottom flask, 1.00 eq. 5-aminotetrazole are suspended in a mixture of 1.10 eq. hypo phosphoric acid (50 wt%) and 10 mL of water. Subsequently, a solution of 1.00 eq. sodium nitrite in water is added dropwise while maintaining the temperature below 35 °C. After 30 min, the pH value is controlled and set to 3–4 through addition of a few drops of an aqueous sodium hydride solution (50 wt%). Afterwards, the mixture is extracted with ethyl acetate (4 × 15 mL) and the combined organic phases are washed with water (1 × 10 mL) and dried over $MgSO_4$. The solvent is removed *in vacuo* to afford 1*H*-tetrazole with a yield of 51% as a yellow solid **A7**.

^{1}H NMR (400 MHz, $(CD_3)_2SO$, 295 K): δ [ppm] = 9.41 (s, 1H, H_{tetr}).

EA (%): calcd. C 17.15, H 2.88, N 79.98; found C 17.01, H 2.72, N 77.01.

2.3.2 Synthesis of 2-Isopropyl-2*H*-tetrazole B7

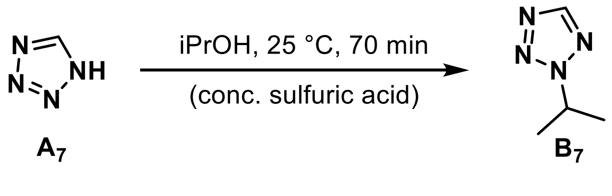

Scheme S10: Synthesis of 2-isopropyl-2*H*-tetrazole **B7**.

To a suspension of 1.00 eq. 1*H*-tetrazole (**A7**) in concentrated sulfuric acid, 1.10 eq. isopropanol is added slowly at 20–25 °C. After stirring for 70 min, the solution is poured into ice-cold water. The mixture is extracted with dichloromethane (3 × 15 mL), and the combined organic layers are washed with water and saturated sodium hydrogen carbonate, dried over $MgSO_4$, and all volatiles are removed *in vacuo* to afford 2-isopropyl-2*H*-tetrazole (**B7**) as a slightly brown oil with a yield of 60%.

^{1}H NMR (400 MHz, DMSO-d_6, 295 K): δ [ppm] = 8.94 (s, 1H, H_{tetr}), 5.15 (hept, ^3J = 6.7 Hz, 1H, CH), 1.56 (d, ^3J = 6.72 Hz, 6H, CH_3).

2.3.3 Synthesis of 1-*t*-Butyl-3-isopropyl-2*H*-tetrazolium Hexafluorophosphate $\mathbf{L_7}$

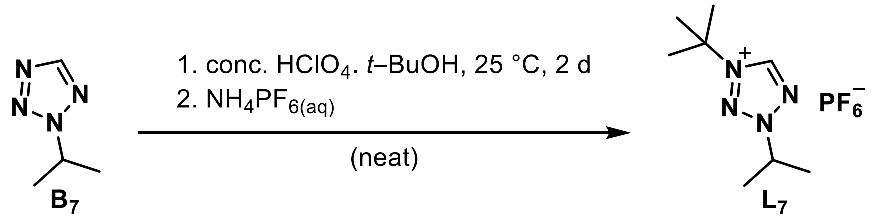

Scheme S11: Synthesis of 1-*t*-butyl-3-isopropyl-2*H*-tetrazolium perchlorate $\mathbf{L_7}$.

To a mixture of 1.00 eq. 2-isopropyl-2*H*-tetrazole ($\mathbf{B_7}$) and perchloric acid (60 wt%), 1.00 eq. *t*-butanol is added slowly at 20–25 °C. The reaction mixture is stirred for 2 d and then poured into ice-cold water. After cooling at 0 °C for 2 d, the precipitate is filtered, washed with water, and all volatiles are removed *in vacuo*. The solid is resuspended in 2 mL of a mixture of acetone and water (1:1) and precipitated upon the addition of 1.5 eq. NH_4PF_6. The solid is removed by filtration and washed with water (3 × 5 mL) to give 1-*t*-butyl-3-isopropyl-2*H*-tetrazolium hexafluorophosphate as a white solid with a yield of 74% $\mathbf{L_7}$.

^{1}**H NMR** (400 MHz, MeCN-d_3, 295 K): δ [ppm] = 9.54 (s, 1H, Htetr), 5.28 (hept, ^{3}J = 6.7 Hz, 1H, CH), 1.77 (s, 9H, $CH_{3,t\text{–butyl}}$), 1.73 (d, 2 J = ^{3}J = 6.7 Hz, 6H, $CH_{3,\text{isopropyl}}$).

^{13}**C NMR** (101 MHz, MeCN-d_3, 295 K): δ [ppm] = 147.2 (s, C_{tetr}), 68.3 (s, $C_{t\text{–butyl}}$), 63.8 (s, CH), 29.0 (s, $C_{t\text{–butyl}}$), 21.5 (s, $C_{\text{isopropyl}}$).

EA (%): calcd. C 30.58, H 5.45, N 17.83, P 9.86, F 36.28; found C 30.99, H 5.48, N 17.70.

2.4 General Procedure for the Synthesis of 1,3-Disubstituted-tetrazolylidene Gold(I) Chloride Complexes 1–7.

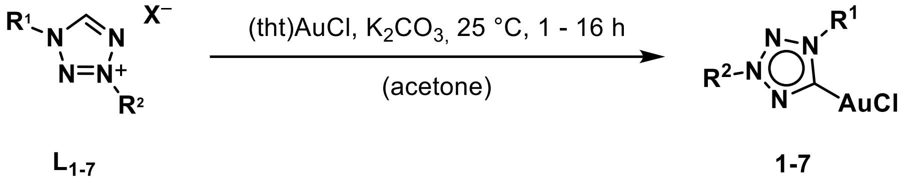

Scheme S12: General synthesis of 1,3-disubstituted tetrazolylidene-gold(I)-chloride complexes **1–4**.

In a glass vial, 1.00 eq. of the tetrazolium proligand (**L**$_{1–7}$), 1.00 eq. chloro(tetrahydrothiophene)gold(I) and 3.00 eq. potassium carbonate are dissolved in 4 mL acetone and stirred for 16 h at 25 °C. After the reaction time, the solvent is removed *in vacuo* and the crude residue is suspended in dichloromethane and filtered over Celite®. Subsequently, the filtrate is concentrated *in vacuo* (1–2 mL), precipitated with *n*-pentane (8 mL) and afterwards stored overnight at –34 °C. The precipitate is filtered, washed with *n*-pentane (2 × 3 mL) and dried *in vacuo*. The resulting crude is purified by filtration over a silica plug to obtain the respective 1,3-disubstituted tetrazolylidene gold(I) chloride complexes **1–7**.

2.4.1 1,3-Diphenyl-tetrazolylidene Gold(I) Chloride 1

Compound **1** is obtained as a white solid with a yield of 67%.

^{1}H NMR (400 MHz, MeCN-d_3, 301 K): δ [ppm] = 8.25–8.23 (m, 2H, $H_{o\text{–phenyl}}$), 8.06–8.04 (m, 2H, $H_{o\text{–phenyl}}$), 7.77–7.72 (m, 6H, $H_{m–, p\text{–phenyl}}$).

^{13}C NMR (101 MHz, MeCN-d_3, 300 K): 176.8 (s, C_{tetr}), 136.5 (s, C_{ar}), 136.3 (s, C_{ar}), 133.6 (s, C_{ar}), 132.7 (s, C_{ar}), 131.4 (s, C_{ar}), 131.5 (s, C_{ar}), 125.8 (s, C_{ar}), 122.1 (s, C_{ar}).

ESI-MS (m/z): [(**L**$_1$ – PF_6)Au – Cl + MeCN]$^+$ calcd, 460.10; found, 460.08; [(**L**$_1$ – PF_6)$_2$$Au_2$Cl]$^+$ calcd, 873.10; found, 873.07.

EA (%): calcd. C 34.34, H 2.22, N 12.32, Au 43.32, Cl 7.80; found C 34.17, H 2.02, N 12.19.

2.4.2 1-(*p*-Tolyl)-3-phenyl-tetrazolylidene Gold(I) Chloride 2

Compound **2** is obtained as a white solid with a yield of 92%.

^{1}H NMR (400 MHz, MeCN-d_3, 301 K): δ [ppm] = 8.23 (d, ^3J = 7.0 Hz, 2H, $H_{o\text{–phenyl}}$), 7.92 (d, ^3J = 7.9 Hz, 2H, $H_{o\text{–tolyl}}$), 7.75 (s, 3H, $H_{m\text{–},\, p\text{–phenyl}}$), 7.53 (d, 2H, ^3J = 8.0 Hz, $H_{m\text{–tolyl}}$), 2.49 (s, 3H, CH_3).

^{13}C NMR (101 MHz, MeCN-d_3, 300 K): 176.7 (s, C_{tetr}), 143.5 (s, C_{ar}), 136.5 (s, C_{ar}), 133.7 (s, C_{ar}), 133.5 (s, C_{ar}), 131.3 (s, C_{ar}), 125.5 (s, C_{ar}), 122.0 (s, C_{ar}), 21.3 (s, CH_3).

ESI-MS (m/z): [(**L2** – PF_6)Au – Cl + MeCN]$^+$ calcd, 474.10; found, 474.56; [(**L2** – PF_6)$_2$Au$_2$Cl]$^+$ calcd, 901.11; found, 900.71.

EA (%): calcd. C 35.88, H 2.58, N 11.95, Au 42.02, Cl 7.56; found C 36.02, H 2.30, N 11.88.

R*f* = 0.37 (DCM) [UV].

2.4.3 1,3-Dimesityl-tetrazolylidene Gold(I) Chloride 3

Compound **3** is obtained as a white solid with a yield of 67%.

^{1}H NMR (400 MHz, MeCN-d_3, 294 K): δ [ppm] = 7.22 (s, 2H, CH), 7.21 (s, 2H, CH), 2.42 (s, 3H, $CH_{3,\, p\text{–mes}}$), 2.41 (s, 3H, $CH_{3,\, p\text{–mes}}$), 2.09 (s, 6H, $CH_{3,\, o\text{–mes}}$), 2.07 (s, 6H, $CH_{3,\, o\text{–mes}}$).

^{13}C NMR (125 MHz, MeCN-d_3, 300 K): 180.2 (s, C_{tetr}), 144.4 (s, C_{ar}), 143.8 (s, C_{ar}), 136.0 (s, C_{ar}), 135.5 (s, C_{ar}), 133.2 (s, C_{ar}), 132.1 (s, C_{ar}), 130.7 (d, C_{ar}), 21.3 (s, CH_3), 17.9 (s, CH_3), 17.3 (s, CH_3).

ESI-MS (m/z): [(**L3** – PF_6)Au – Cl + MeCN]$^+$ calcd, 544.20; found, 543.97.

EA (%): calcd. C 42.35, H 4.21, N 10.40, Au 36.55, Cl 6.58; found C 42.28, H 3.91, N 10.35.

R*f* = 0.47 (DCM) [UV].

2.4.4 1-(2,6-Diisopropylphenyl)-3-phenyl-tetrazolylidene Gold(I) Chloride 4

Compound **4** is obtained as an orange solid with a yield of 99%.

^{1}H NMR (400 MHz, MeCN-d_3, 301 K): δ [ppm] = 8.27 (d, ^3J = 5.9 Hz, 2H, $H_{o\text{–phenyl}}$), 7.75–7.71 (m, 4H, $H_{m\text{–}, p\text{–phenyl}, p\text{–Dipp}}$), 7.51 (d, ^3J = 7.8 Hz, 2H, $H_{m\text{–Dipp}}$), 2.34 (m, 2H, CH), 1.28 (s, 3H, CH_3), 1.22 (s, 3H, CH_3).

^{13}C NMR (126 MHz, MeCN-d_3, 300 K): 180.4 (s, C_{tetr}), 147.1 (s, C_{ar}), 136.8 (s, C_{ar}), 133.7 (d, C_{ar}), 131.5 (s, C_{ar}), 131.3 (s, C_{ar}), 125.8 (s, C_{ar}), 122.2 (s, C_{ar}), 29.3 (s, CH), 24.3 (s, CH_3), 24.1 (s, CH_3).

ESI-MS (m/z): $[(\mathbf{L_4} - BF_4)Au - Cl + MeCN]^+$ calcd, 544.18; found, 544.66; $[(\mathbf{L_4} - BF_4)_2Au_2Cl]^+$ calcd, 1041.27; found, 1041.25.

EA (%): calcd. C 42.35, H 4.12, N 10.40, Au 36.55, Cl 6.58; found C 42.41, H 4.04, N 10.36.

R_f = 0.50 (DCM) [UV].

2.4.5 1-Methyl-3-phenyl-tetrazolylidene Gold(I) Chloride 5

Compound **5** is obtained as a white solid with a yield of 84%.

^{1}H NMR (400 MHz, MeCN-d_3, 299 K): δ [ppm] = 8.13 (d, ^3J = 8.2 Hz, 2H, $H_{o\text{–phenyl}}$), 7.71–7.69 (m, 3H, $H_{m\text{–}, p\text{–phenyl}}$), 4.32 (s, 3H, CH_3).

^{13}C NMR (101 MHz, MeCN-d_3, 300 K): 178.3 (s, C_{tetr}), 136.6 (s, C_{ar}), 133.2 (s, C_{ar}), 131.3 (s, C_{ar}), 121.9 (s, C_{ar}), 39.3 (s, CH_3).

ESI-MS (m/z): $[(\mathbf{L_5} - BF_4)Au - Cl + MeCN]^+$ calcd, 398.07; found, 398.03; $[(\mathbf{L_5} - BF_4)_2Au - Cl]^+$ calcd, 517.12; found, 517.07; $[(\mathbf{L_5} - BF_4)_2Au_2Cl]^+$ calcd, 749.05; found, 748.94.

EA (%): calcd. C 24.48, H 2.05, N 14.27, Au 50.17, Cl 9.03; found C 24.81, H 1.90, N 13.93.

R_f = 0.37 (DCM) [UV].

2.4.6 1-Methyl-3-*p*-tolyl-tetrazolylidene Gold(I) Chloride 6

Compound **6** is obtained as a white solid with a yield of 53%.

^{1}H NMR (400 MHz, MeCN-d_3, 301 K): δ [ppm] = 8.00 (d, ^{3}J = 8.4 Hz, 2H, $H_{o\text{–tolyl}}$), 7.50 (d, ^{3}J = 8.9 Hz, 2H, $H_{m\text{–tolyl}}$), 4.30 (s, 3H, NCH_3), 2.47 (s, 3H, CCH_3).

^{13}C NMR (126 MHz, MeCN-d_3, 300 K): 177.9 (s, C_{tetr}), 144.2 (s, C_{ar}), 134.3 (s, C_{ar}), 131.6 (s, C_{ar}), 121.6 (s, C_{ar}), 39.1 (s, NCH_3), 21.3 (s, CCH_3).

ESI-MS (m/z): [(**L$_6$** – BF_4)Au – Cl + MeCN]$^+$ calcd, 412.08; found, 412.02; [(**L$_6$** – BF_4)$_2$Au – Cl]$^+$ calcd, 545.15; found, 545.06; [(**L$_6$** – BF_4)$_2$$Au_2$Cl]$^+$ calcd, 777.08; found, 776.92.

EA (%): calcd. C 26.58, H 2.48, N 13.78, Au 48.44, Cl 8.72; found C 26.89, H 2.30, N 13.55.

R$_f$ = 0.39 (DCM) [UV].

2.4.7 1-*t*-Butyl-3-isopropyl-tetrazolylidene Gold(I) Chloride 7

Compound **7** is obtained as a white solid with a yield of 69%.

^{1}H NMR (400 MHz, MeCN-d_3, 294 K): δ [ppm] = 5.09 (hept, ^{3}J = 6.7 Hz, 1H, CH), 1.90 (s, 9H, $CH_{3,\, t\text{–butyl}}$), 1.66 (s, 3H, $CH_{3,\, o\text{–isopropyl}}$), 1.64 (s, 3H, $CH_{3,\, o\text{–isopropyl}}$).

^{13}C NMR (101 MHz, MeCN-d_3, 295 K): 174.2 (s, C_{tetr}), 65.0 (s, $C_{t\text{–butyl}}$), 60.7 (s, CH), 30.5 (s, $C_{t\text{–butyl}}$), 21.7 (s, $C_{isopropyl}$).

ESI-MS (m/z): [(**L$_7$** – PF6)Au – Cl + MeCN]$^+$ calcd, 406.30; found, 406.04; [(**L$_7$** – PF_6)$_2$$Au_2$Cl]$^+$ calcd, 765.2; found, 764.95.

EA (%): calcd. C 23.98, H 4.03, N 13.98, Au 49.16, Cl 8.85; found C 24.34, H 3.90, N 13.60.

R$_f$ = 0.40 (DCM) [UV].

2.5 General Procedure for the Synthesis of 1,3-Disubstituted-tetrazolylidene Gold(I) Bis-NHC Complexes B1–B2.

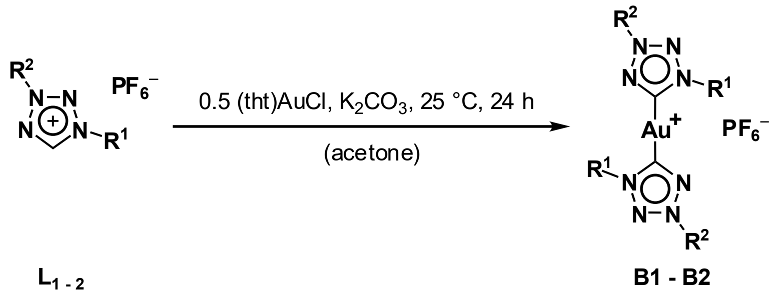

Scheme S13: General synthesis of 1,3-disubstituted tetrazolylidene gold(I) bis-NHC complexes **B1–B2**.

In a glass vial, 2.00 eq. of the tetrazolium proligand (**L**$_{1\text{–}2}$), 1.00 eq. chloro(tetrahydrothiophene)gold(I) and 3.00 eq. potassium carbonate are dissolved in 4 mL acetone and stirred for 24 h at 25 °C. After the reaction time, the solvent is removed *in vacuo* and the crude residue is suspended in dichloromethane and filtered over Celite®. Subsequently, the filtrate is concentrated *in vacuo* (1–2 mL), precipitated with *n*-pentane (8 mL). The resulting crude is purified by filtration over a silica plug to remove formed mono-NHC complex followed by flushing with DCM/MeOH (10:1) to obtain the respective 1,3-disubstituted tetrazolylidene gold(I) bis-NHC complexes **B1–B2**.

2.5.1 Bis-(1,3-diphenyl-tetrazolylidene) Gold(I) Hexafluorophosphate B1

Compound **B1** is obtained as an off-white solid with a yield of 47%.

^{1}H NMR (400 MHz, MeCN-*d*$_3$, 301 K): δ [ppm] = 8.28–8.25 (m, 4H, $H_{o\text{–phenyl}}$), 7.97–7.96 (m, 4H, $H_{o\text{–phenyl}}$), 7.79–7.73 (m, 8H, $H_{m\text{–phenyl}}$), 7.79–7.73 (m, 4H, $H_{p\text{–phenyl}}$).

^{13}C NMR (101 MHz, MeCN-*d*$_3$, 300 K): 188.6 (s, C_{tetr}), 136.4 (s, C_{ar}), 136.1 (s, C_{ar}), 133.8 (s, C_{ar}), 132.9 (s, C_{ar}), 131.5 (s, C_{ar}), 131.1 (s, C_{ar}), 125.6 (s, C_{ar}), 122.2 (s, C_{ar}).

ESI-MS (m/z): [(**L**$_1$ – PF_6)2Au]$^+$ calcd, 641.15; found, 641.17.

EA (%): calcd. C 39.71, H 2.56, N 14.25, Au 25.05, P 3.94, F 14.49; found C 39.96, H 2.58, N 13.87.

2.5.2 Bis-(1-(*p*-tolyl)-3-phenyl-tetrazolylidene) Gold(I) Hexafluorophosphate B2

Compound **B2** is obtained as an off-white solid with a yield of 33%.

^{1}H NMR (400 MHz, MeCN-d_3, 301 K): δ [ppm] = 8.26 (d, ^3J = 7.3 Hz, 4H, $H_{o\text{–phenyl}}$), 7.85 (d, ^3J = 8.0 Hz, 4H, $H_{o\text{–tolyl}}$), 7.77–7.75 (m, 6H, $H_{m\text{–}, p\text{–phenyl}}$), 7.46 (d, 4H, ^3J = 8.1 Hz, $H_{m\text{–tolyl}}$), 2.49 (s, 6H, CH_3).

^{13}C NMR (101 MHz, MeCN-d_3, 300 K): 188.4 (s, C_{tetr}), 143.6 (s, C_{ar}), 136.3 (s, C_{ar}), 133.7 (s, C_{ar}), 133.6 (s, C_{ar}), 131.4 (d, C_{ar}), 125.3 (s, C_{ar}), 122.0 (s, C_{ar}), 21.3 (s, CH_3).

ESI-MS (m/z): [(**L2** – PF_6)2Au]$^+$ calcd, 669.18; found, 669.07.

EA (%): calcd. C 41.29, H 2.97, N 13.76, Au 24.18 P 3.80, F 14.00; found C 41.37, H 3.07, N 13.47.

3 Analytic Data

3.1 *N*-2-Diphenylhydrazinecarbothioamide A1

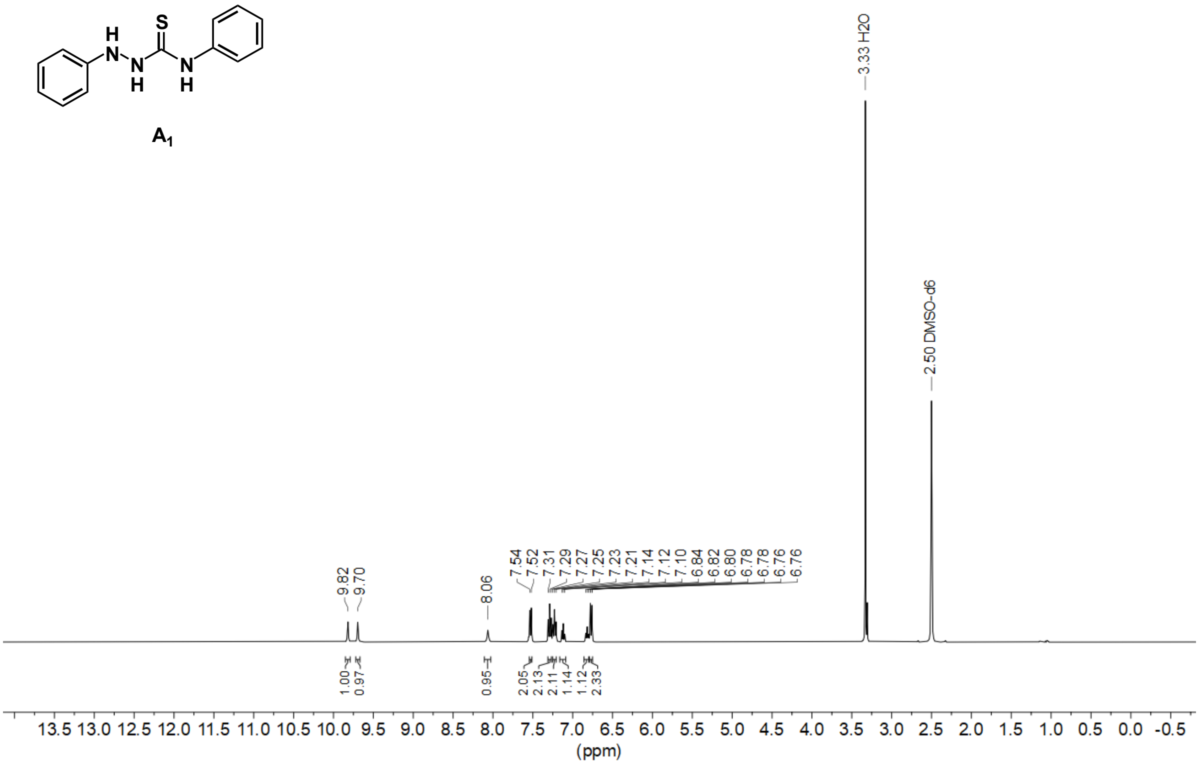

Figure S1: ^{1}H-NMR spectrum of **A1** in DMSO-d_6.

3.2 *N*-(*p*-Polyl)-2-phenylhydrazinecarbothioamide A2

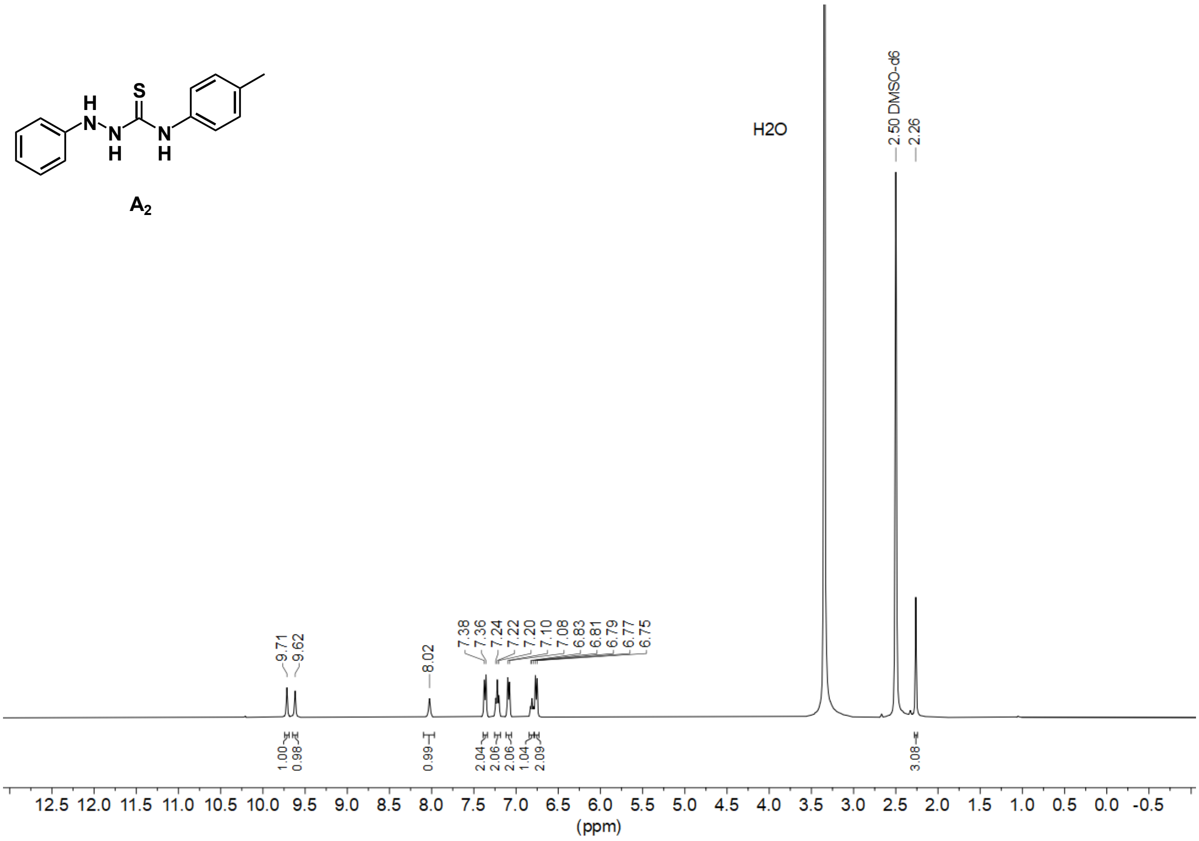

Figure S2: ^{1}H-NMR spectrum of **A2** in DMSO-d_6.

3.3 *N*-2-Dimesitylhydrazinecarbothioamide A$_3$

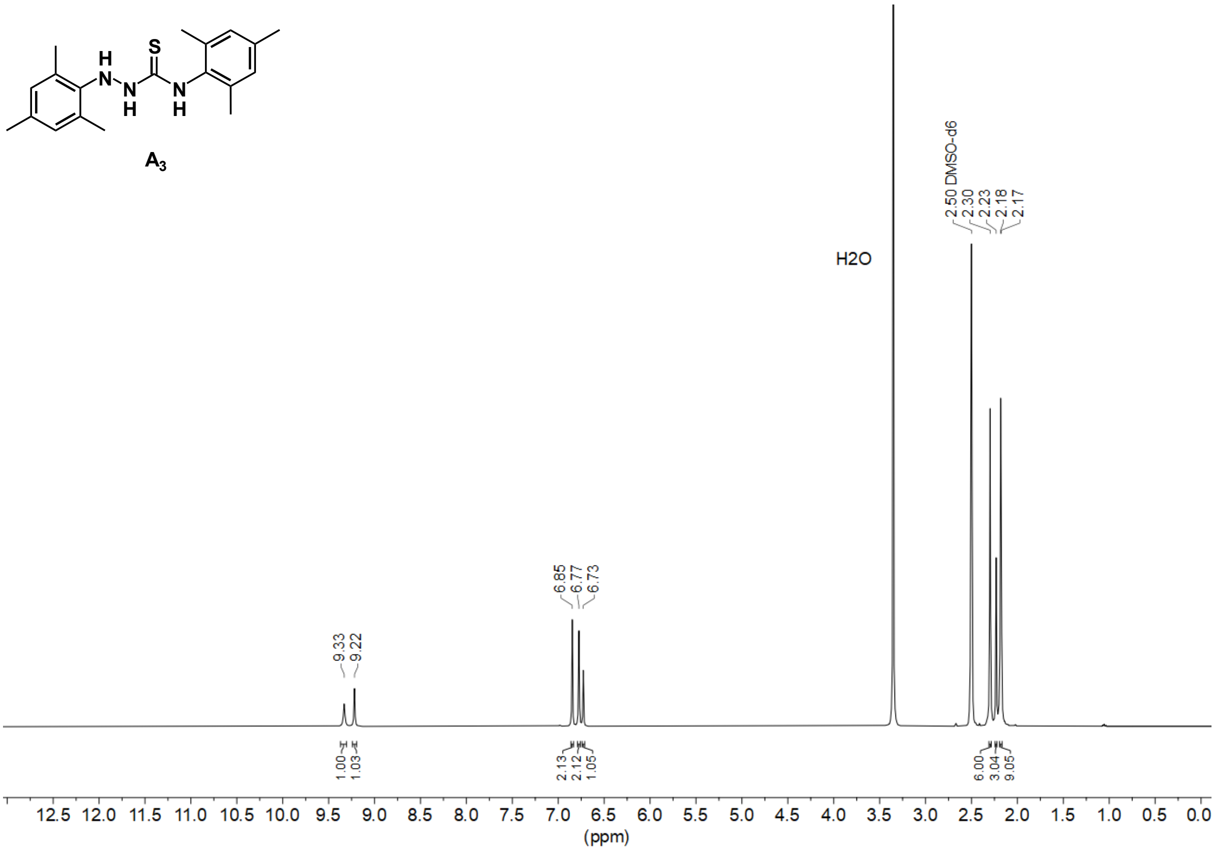

Figure S3: ^{1}H-NMR spectrum of **A$_3$** in DMSO-d_6.

3.4 *N*-(2,6-Diisopropylphenyl)-2-phenylhydrazinecarbothioamide A$_4$

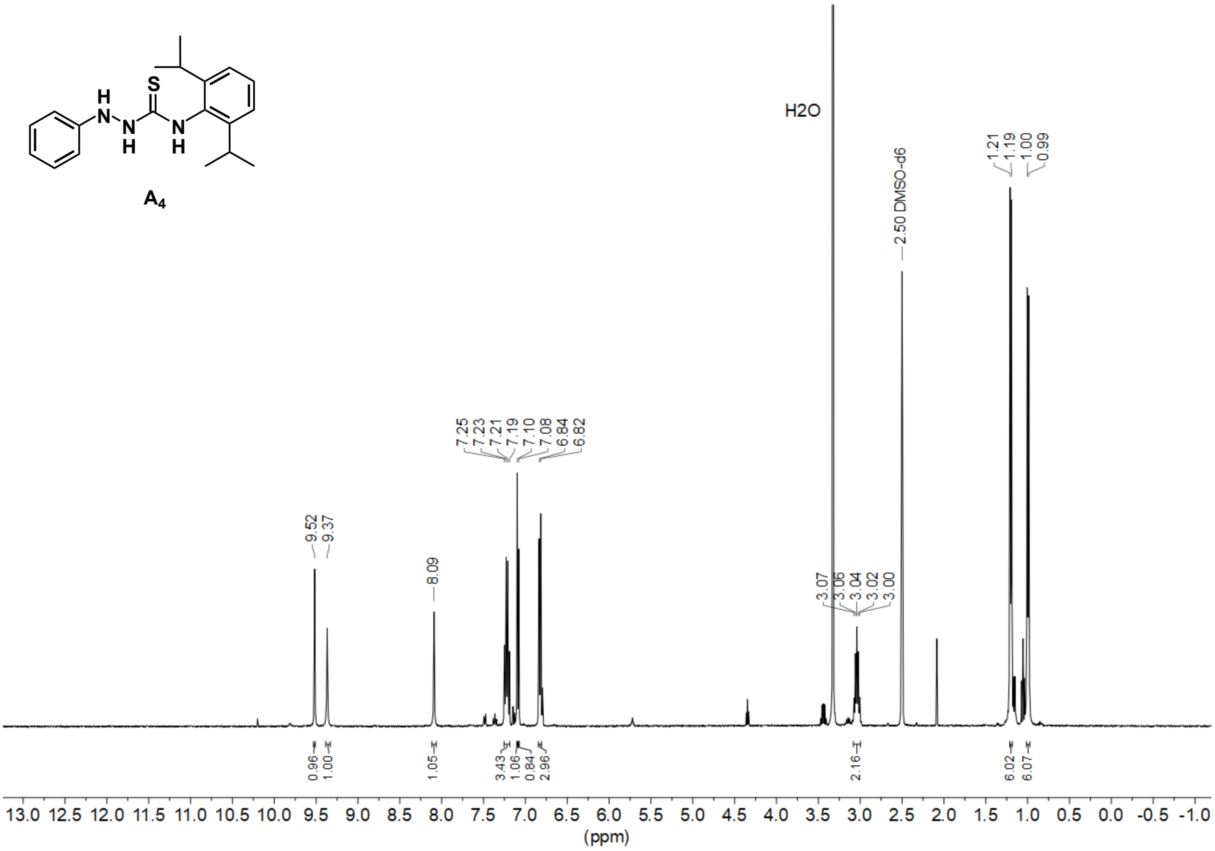

Figure S4: ^{1}H-NMR spectrum of **A$_4$** in DMSO-d_6.

3.5 5-Anilino-3-phenyl-[1,2,3,4]-oxatriazolium betaine B_1

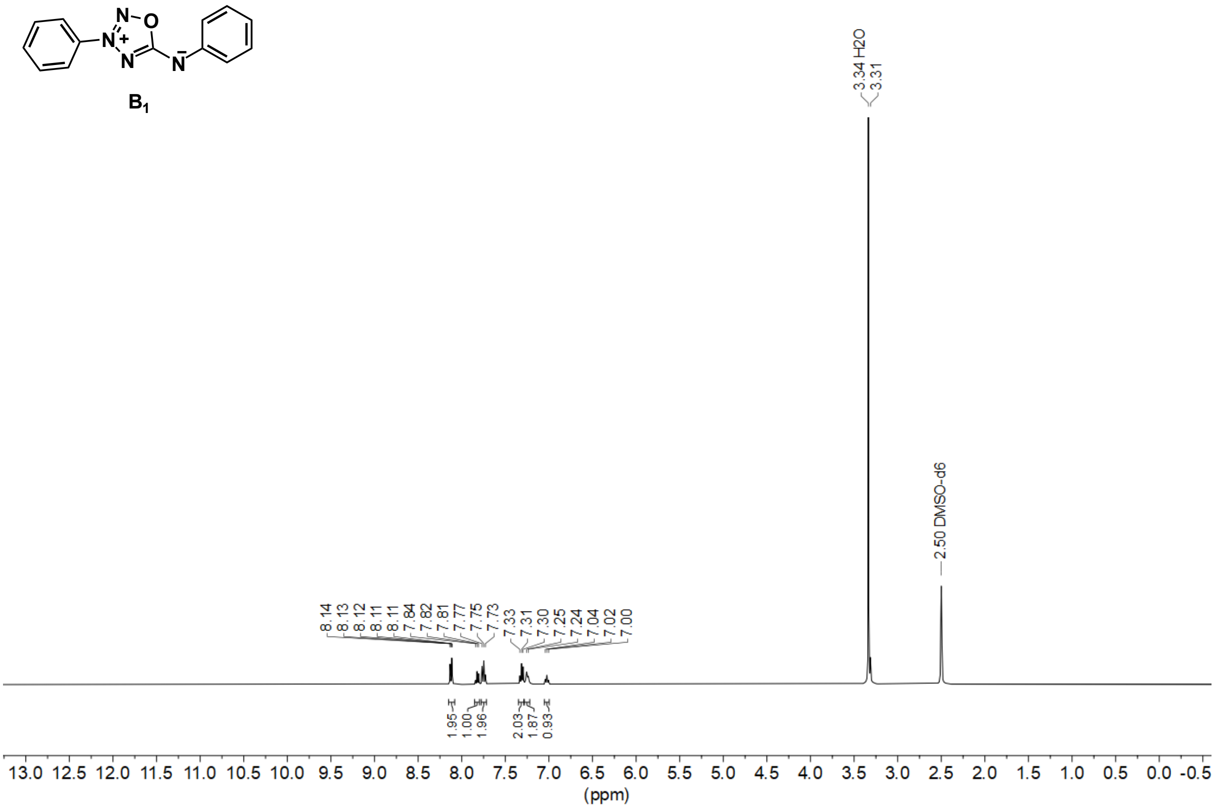

Figure S5: ^{1}H-NMR spectrum of **B₁** in DMSO-d_6.

3.6 5-(4-Methyl-anilino)-3-phenyl-[1,2,3,4]-oxatriazolium Betaine B$_2$

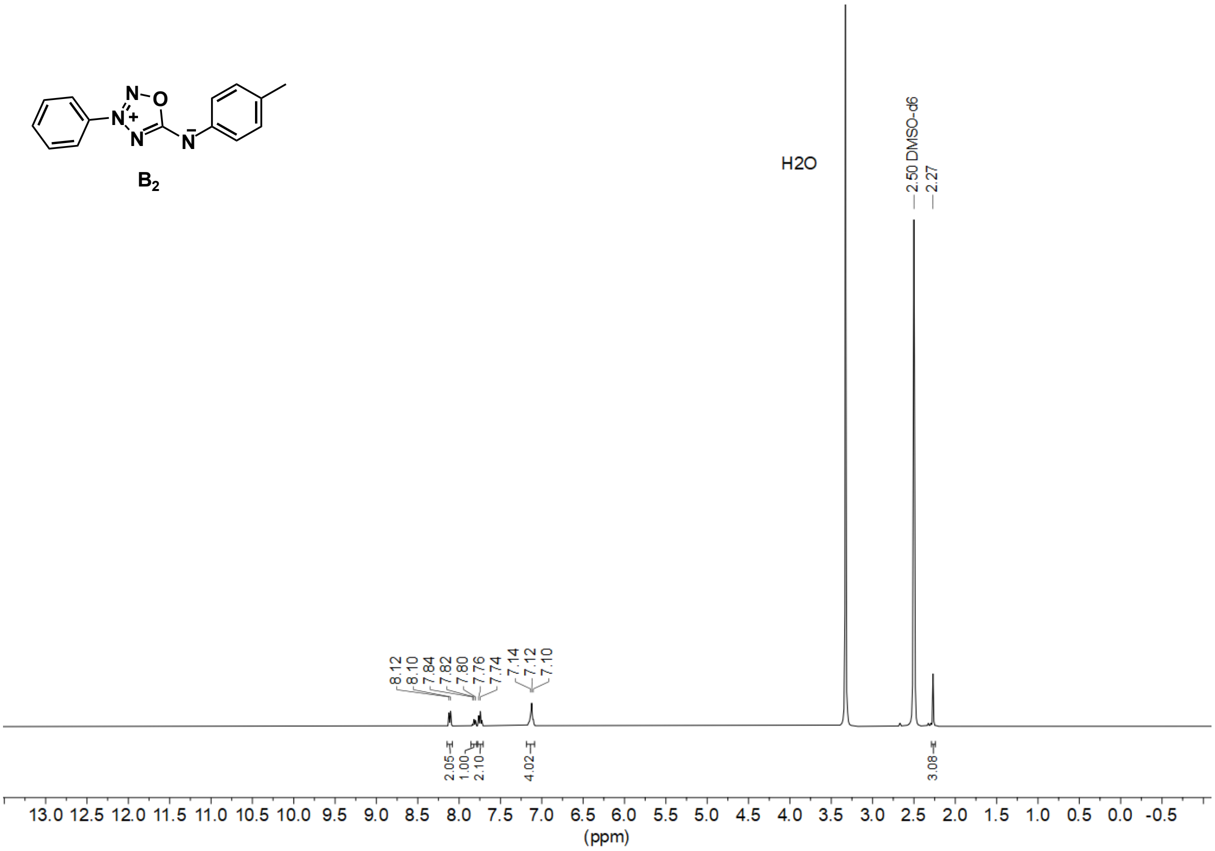

Figure S6: ^{1}H-NMR spectrum of **B$_2$** in DMSO-d_6.

3.7 5-Mesityl-3-mesityl-[1,2,3,4]-oxatriazolium Betaine B3

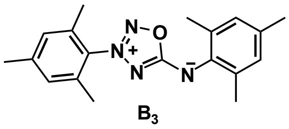

B$_3$

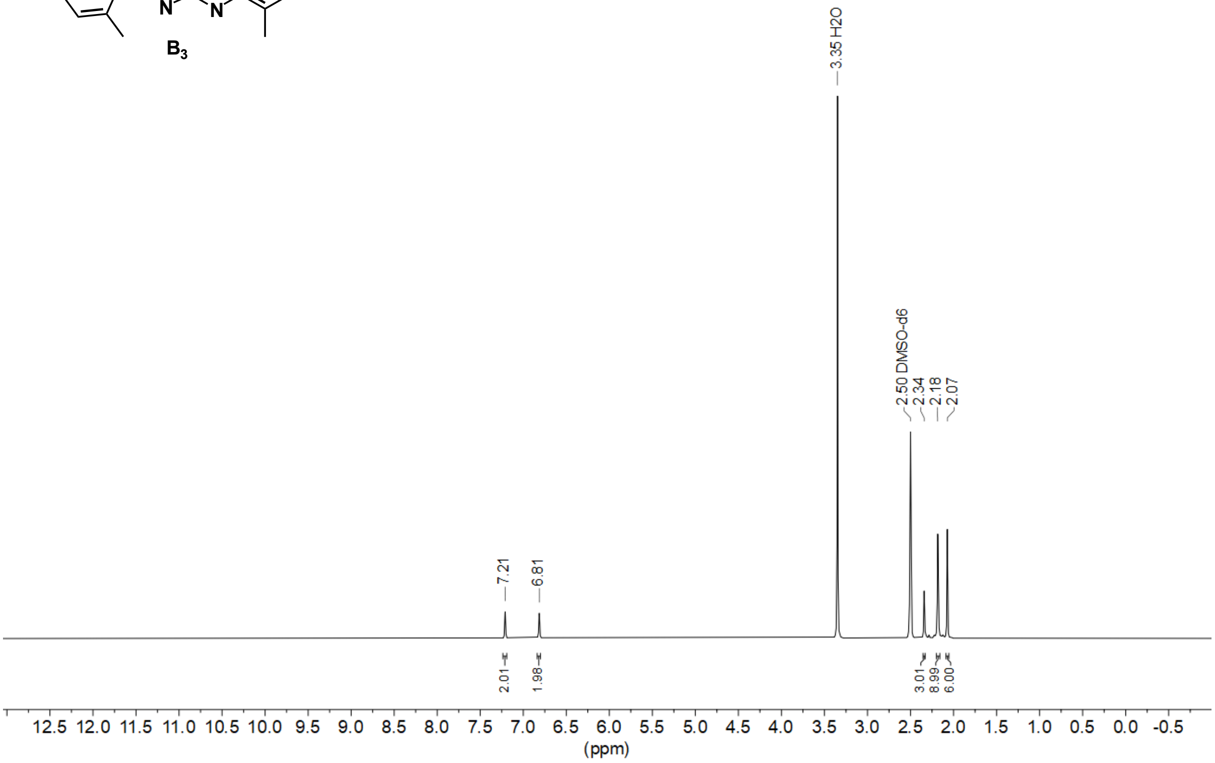

Figure S7: ^{1}H-NMR spectrum of **B$_3$** in DMSO-d_6.

3.8 *5*-(2,6-Diisopropylphenyl)-3-phenyl-[1,2,3,4]-oxatriazolium Betaine B4

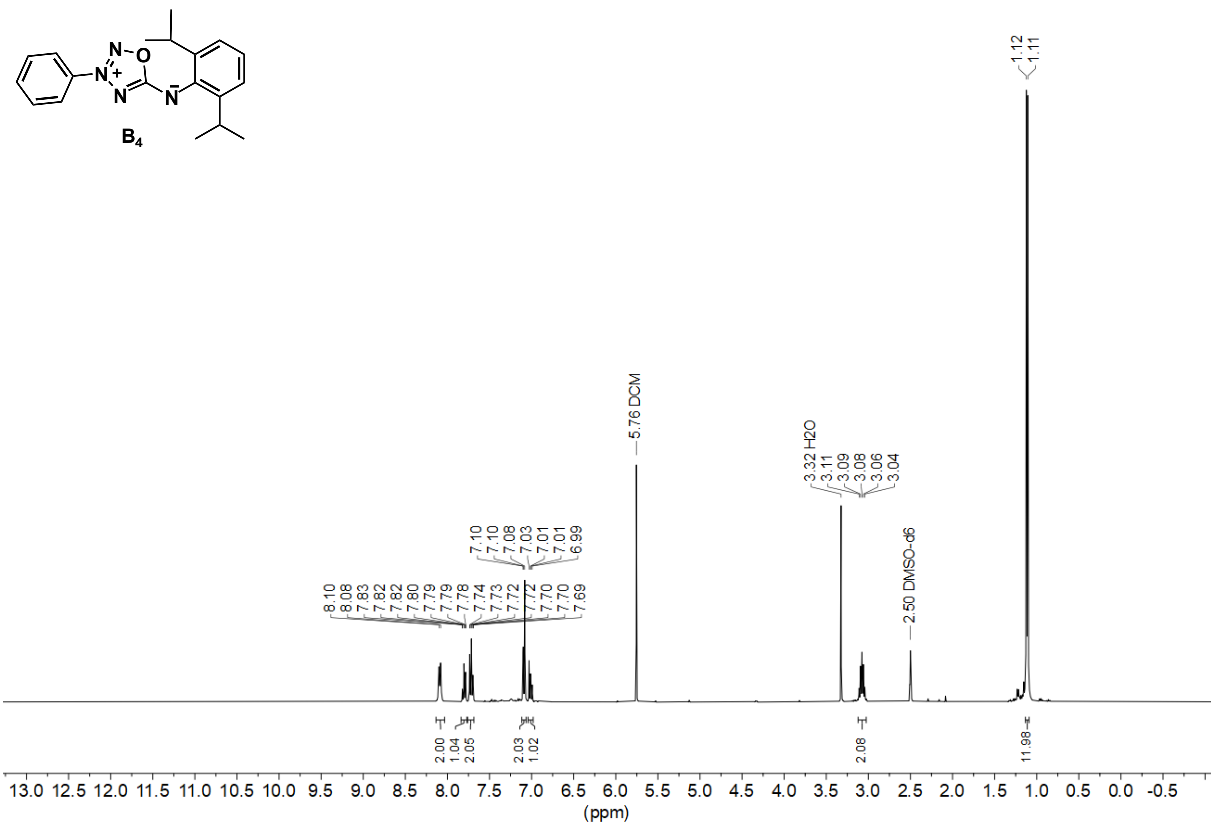

Figure S8: ^{1}H-NMR spectrum of **B4** in DMSO-d_6.

3.9 2,4-Diphenyl-1*H*-tetrazol-2-ium-5-olate C_1

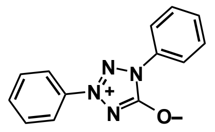

C_1

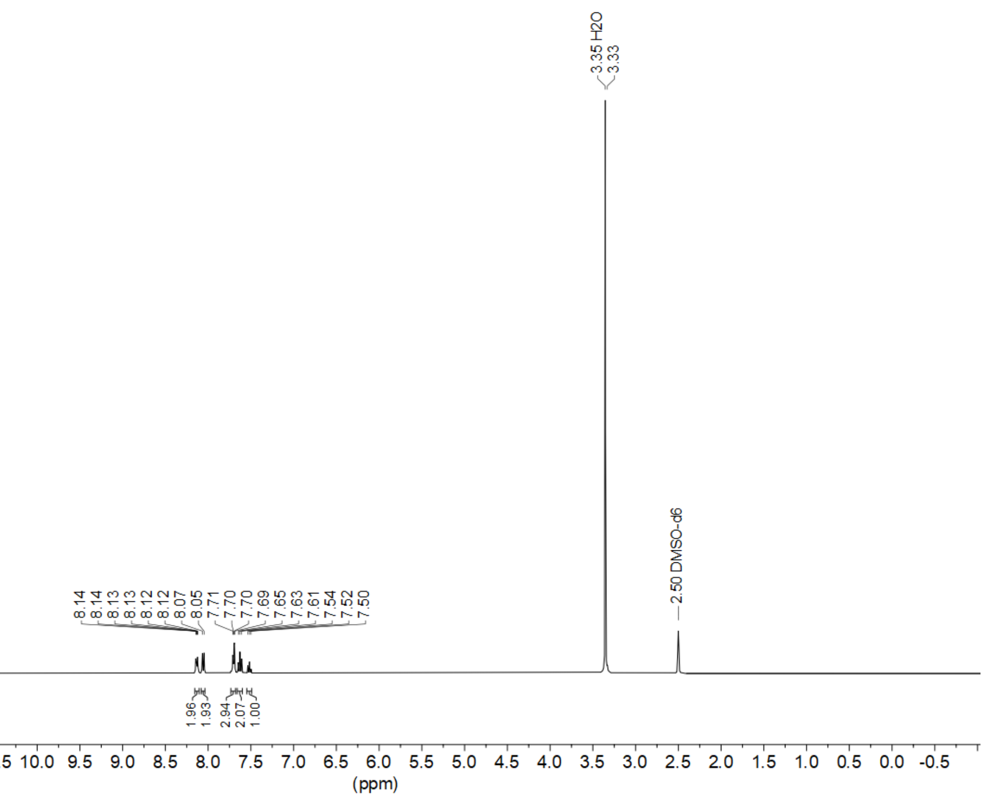

Figure S9: ^{1}H-NMR spectrum of **C_1** in DMSO-d_6.

3.10 3-Phenyl-1-(*p*-tolyl)-1*H*-tetrazole-3-ium-5-olate C₂

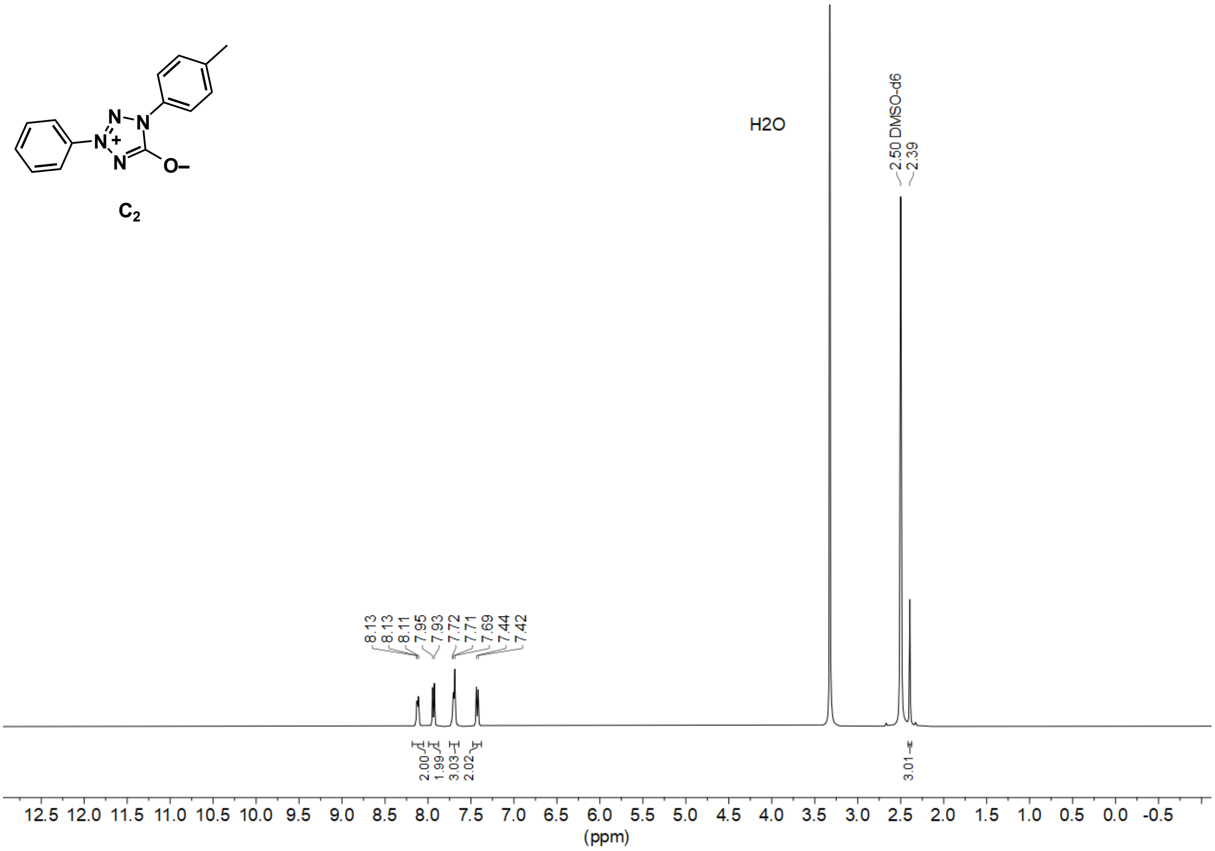

Figure S10: ^{1}H-NMR spectrum of **C$_2$** in DMSO-d_6.

3.11 1,3-Dimesityl-1*H*-tetrazol-3-ium-5-olate C₃

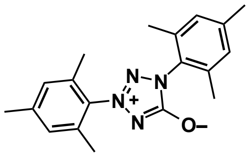

$\mathbf{C_3}$

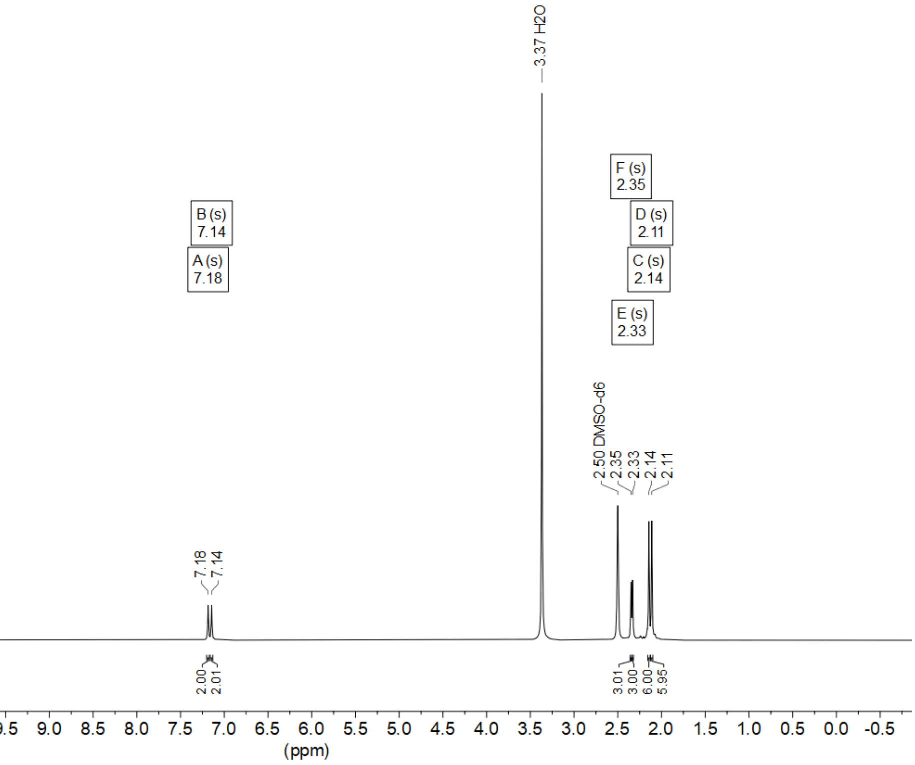

Figure S11: ^{1}H-NMR spectrum of $\mathbf{C_3}$ in DMSO-d_6.

3.12 *5*-(2,6-Diisopropylphenyl)-3-phenyl-1*H*-tetrazol-3-ium-5-olate C4

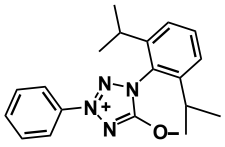

C4

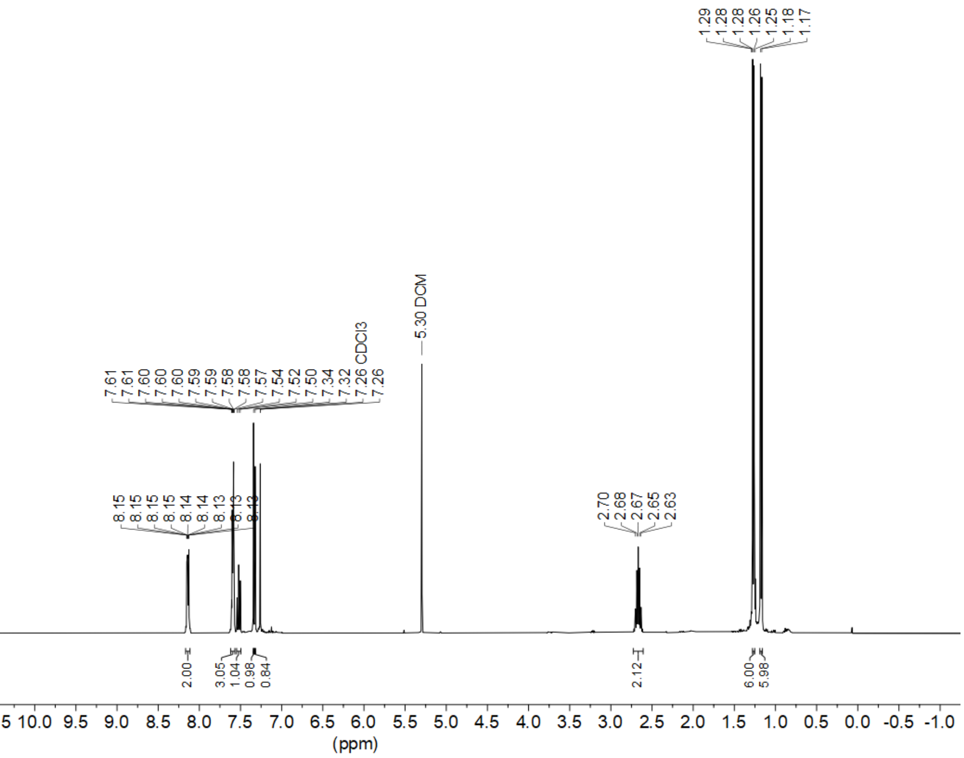

Figure S12: ^{1}H-NMR spectrum of **C4** in DMSO-d_6.

3.13 1,3-Diphenyl-tetrazolium-5-thiolate D$_1$

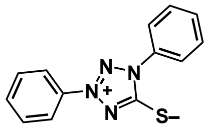

D$_1$

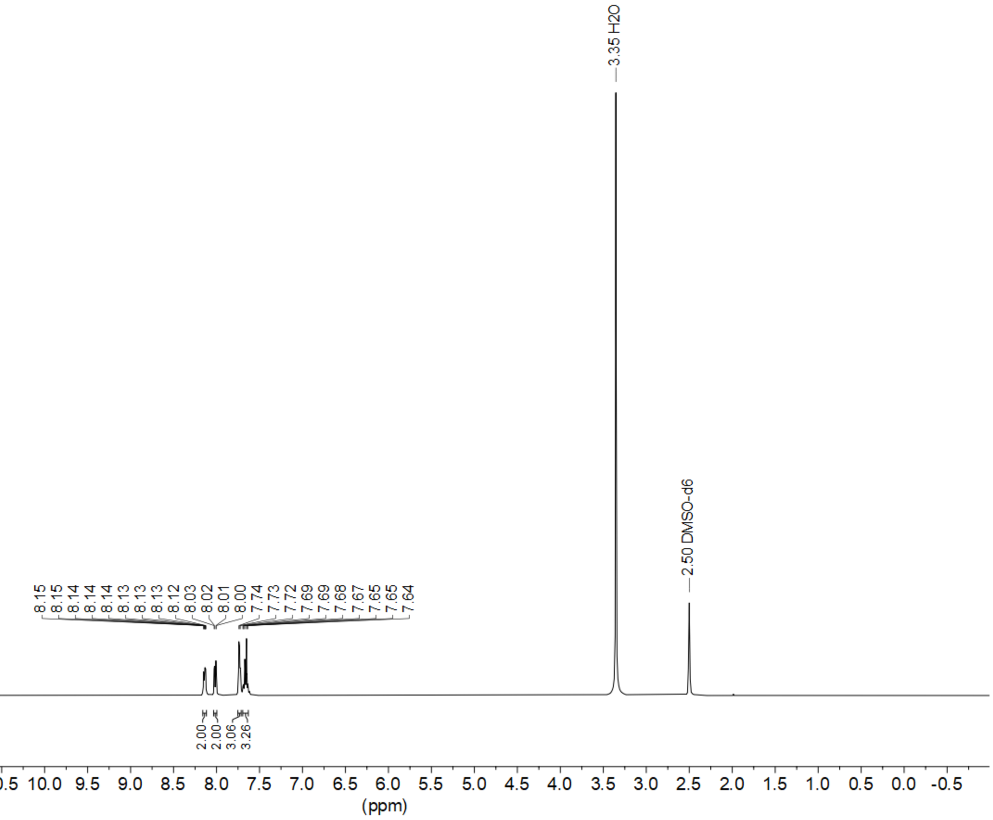

Figure S13: ^{1}H-NMR spectrum of **D$_1$** in DMSO-d_6.

3.14 3-Phenyl-1-(*p*-tolyl)-1*H*-tetrazole-3-ium-5-thiolate D_2

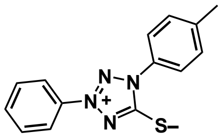

D_2

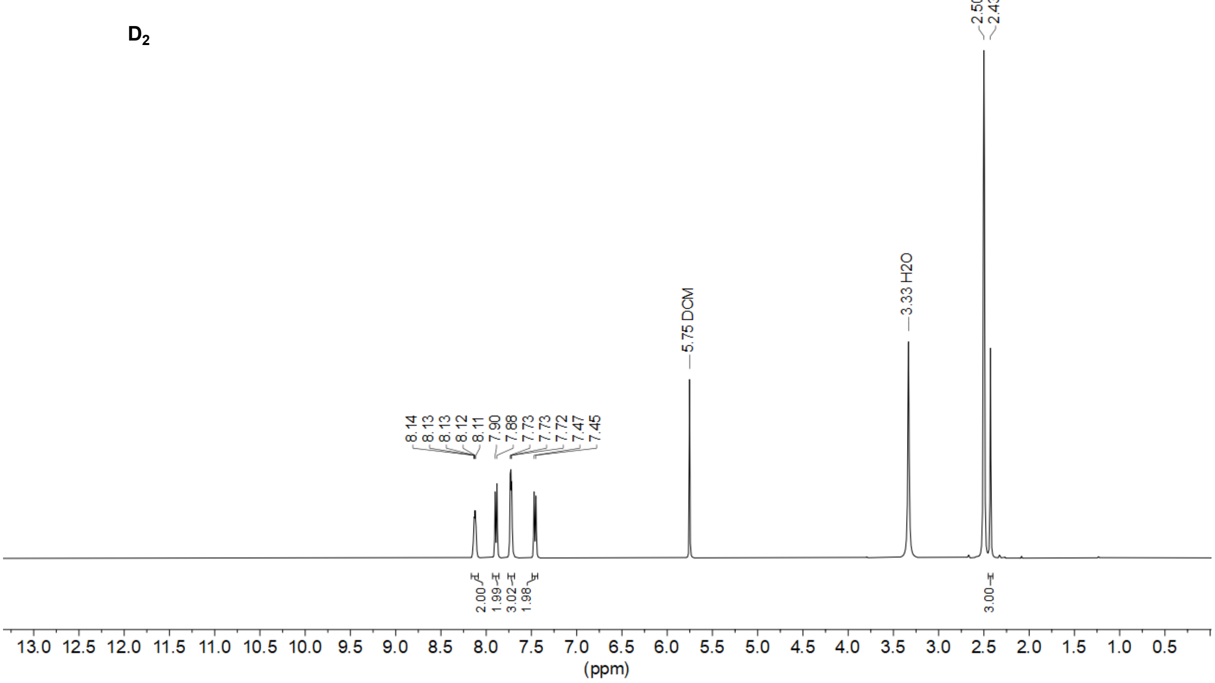

Figure S14: ^{1}H-NMR spectrum of **D_2** in DMSO-d_6.

3.15 1,3-Dimesityl-1*H*-tetrazol-3-ium-5-thiolate D$_3$

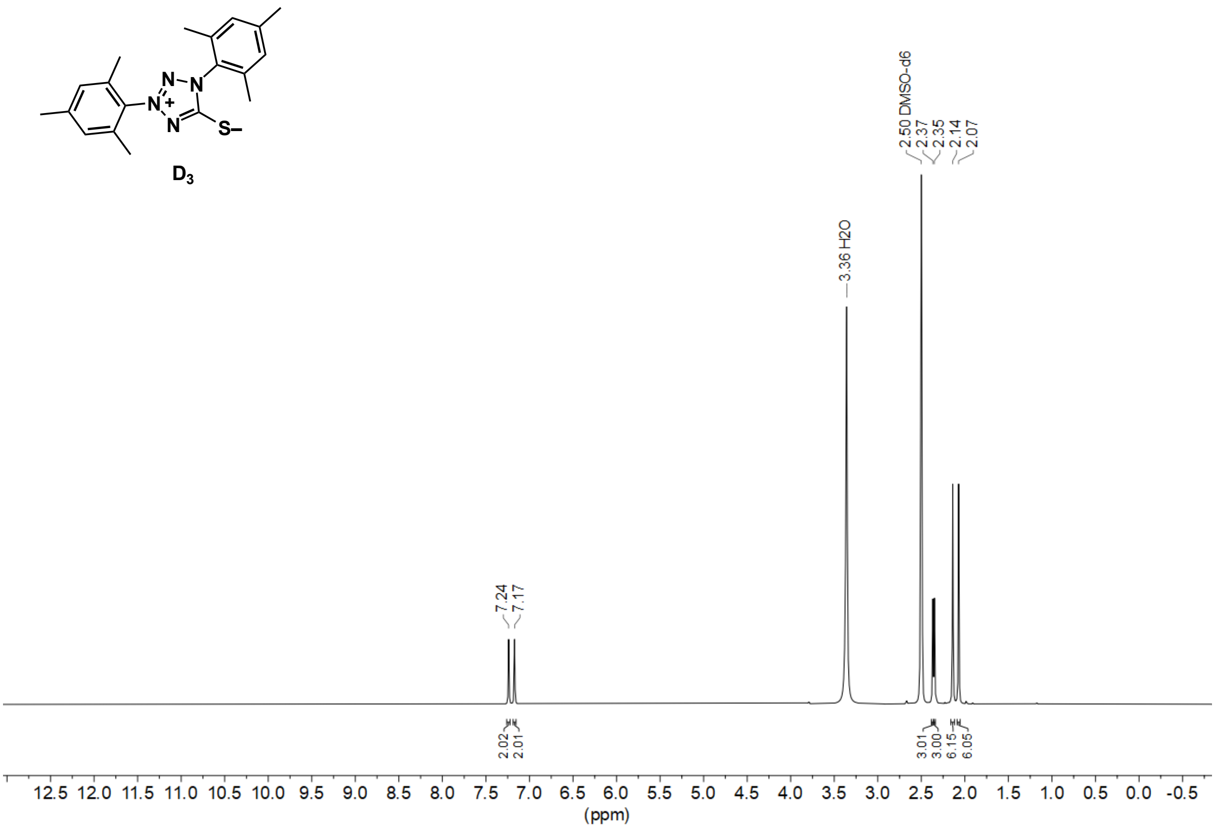

Figure S15: ^{1}H-NMR spectrum of **D$_3$** in DMSO-d_6.

3.16 3-Phenyl-1-(2,6-diisopropylphenyl)-1*H*-tetrazole-3-ium-5-thiolate D$_4$

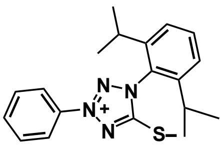

D$_4$

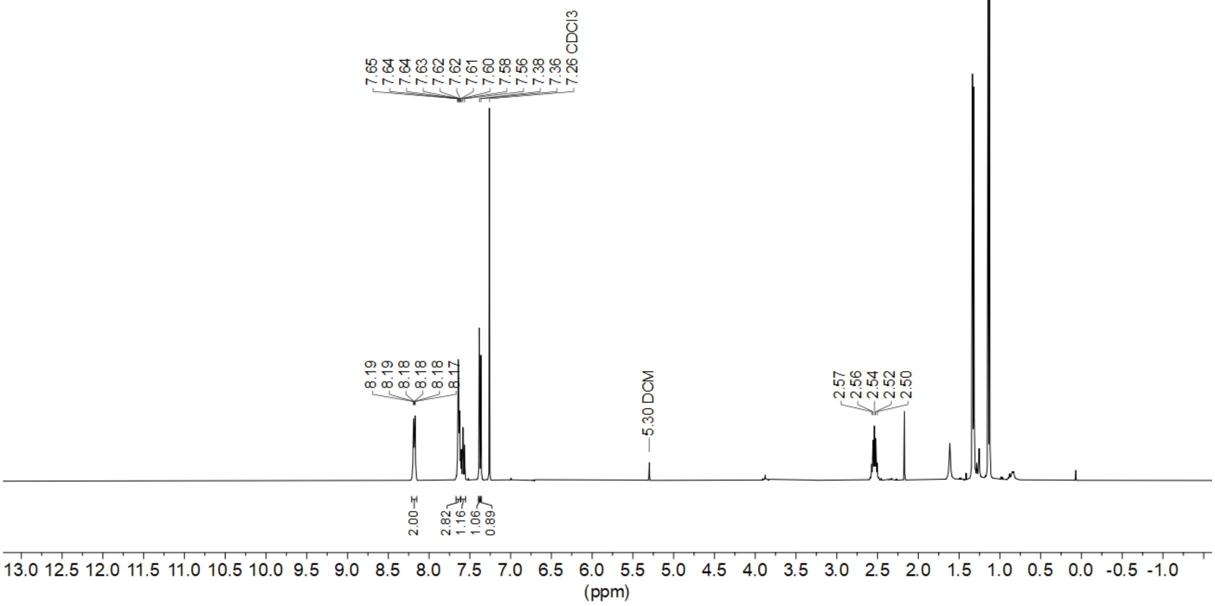

Figure S16: ^{1}H-NMR spectrum of **D$_4$** in DMSO-d_6.

3.17 1,3-Diphenyl-1*H*-tetrazole-3-ium Hexafluorophosphate L1

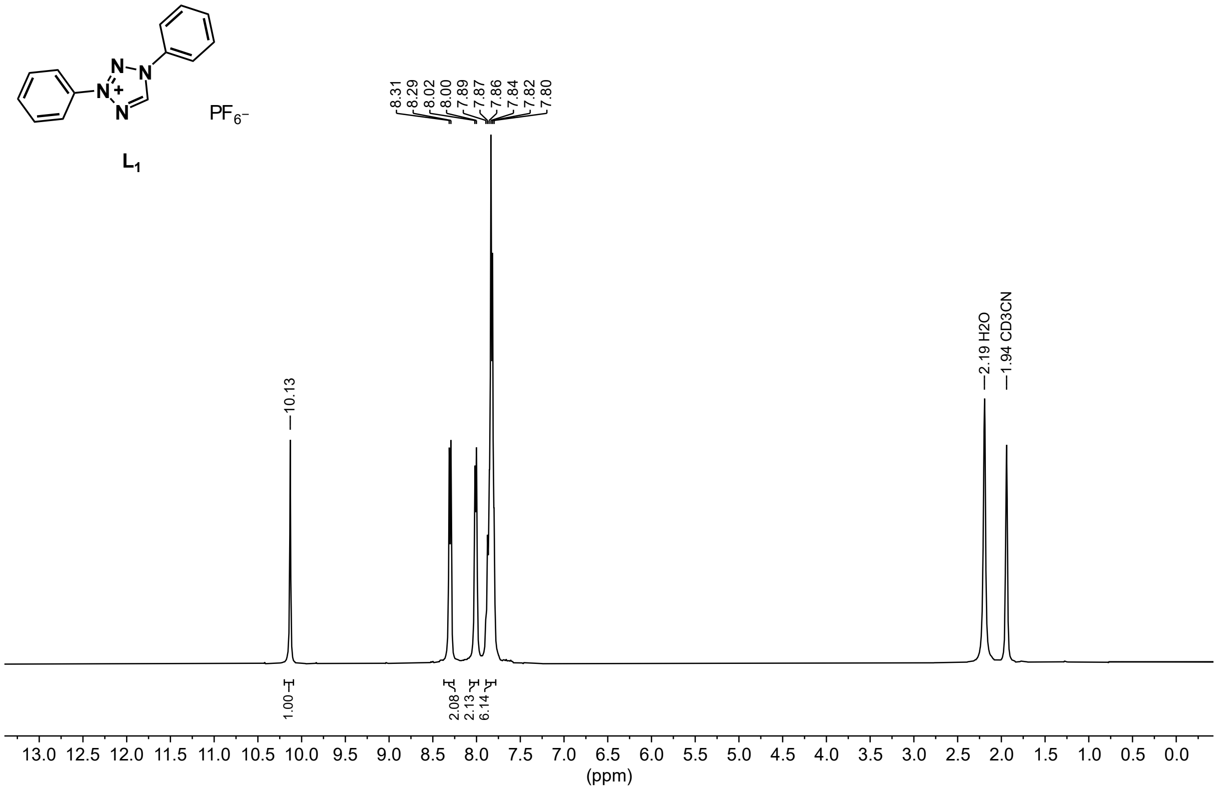

Figure S17: ^{1}H-NMR spectrum of **L1** in MeCN-d_3.

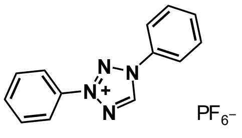

L1

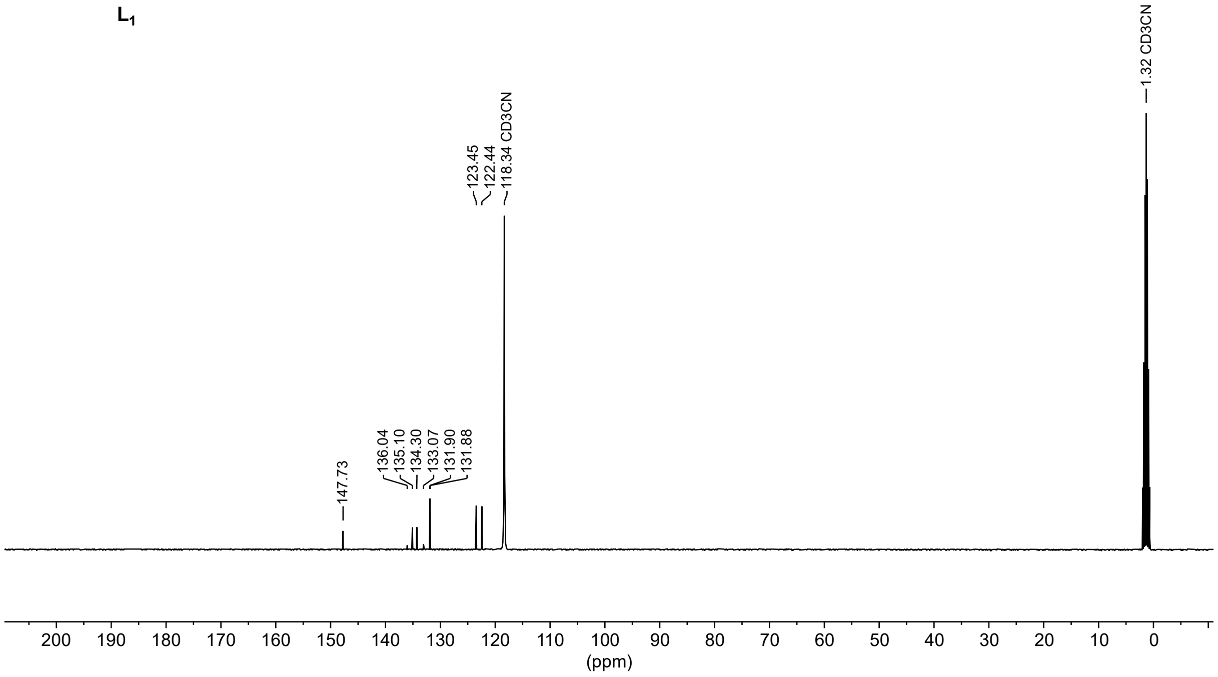

Figure S18: ^{13}C-NMR spectrum of **L1** in MeCN-d_3.

3.18 3-Phenyl-1-(*p*-tolyl)-1*H*-tetrazole-3-ium Hexafluorophosphate L_2

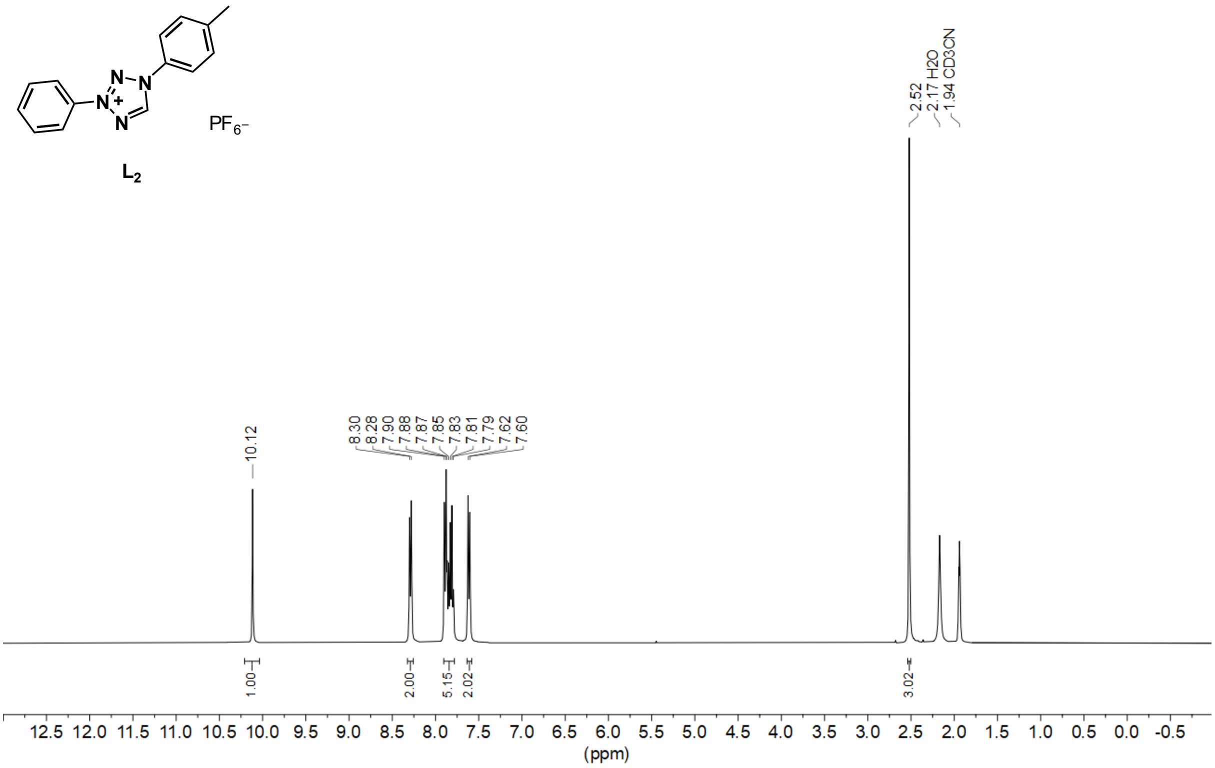

Figure S19: ^{1}H-NMR spectrum of **L_2** in MeCN-*d*$_3$.

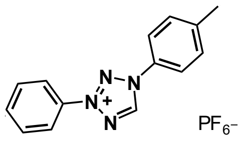

L_2

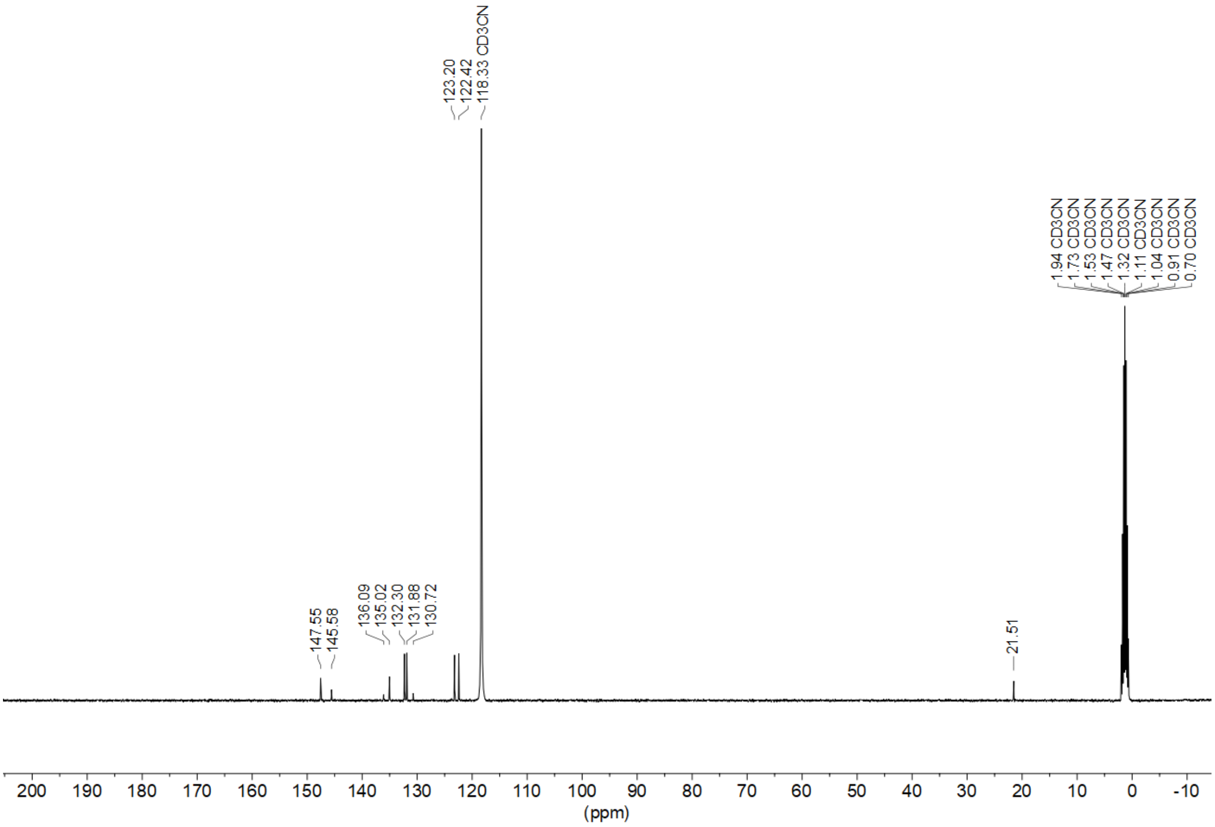

Figure S20: ^{13}C-NMR spectrum of **L_2** in MeCN-d_3.

3.19 1,3-Dimesityl-1*H*-tetrazolium Hexafluorophosphate L_3

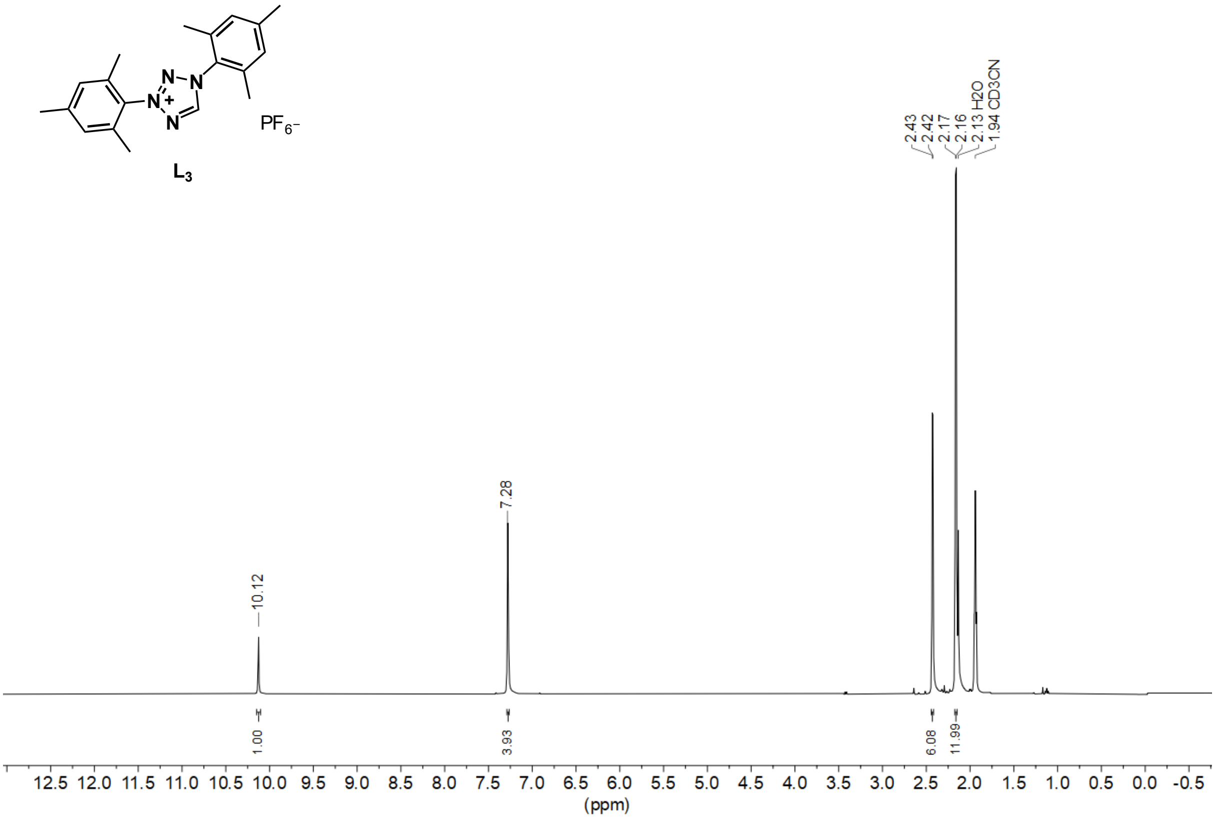

Figure S21: ^{1}H-NMR spectrum of **L3** in MeCN-*d*3.

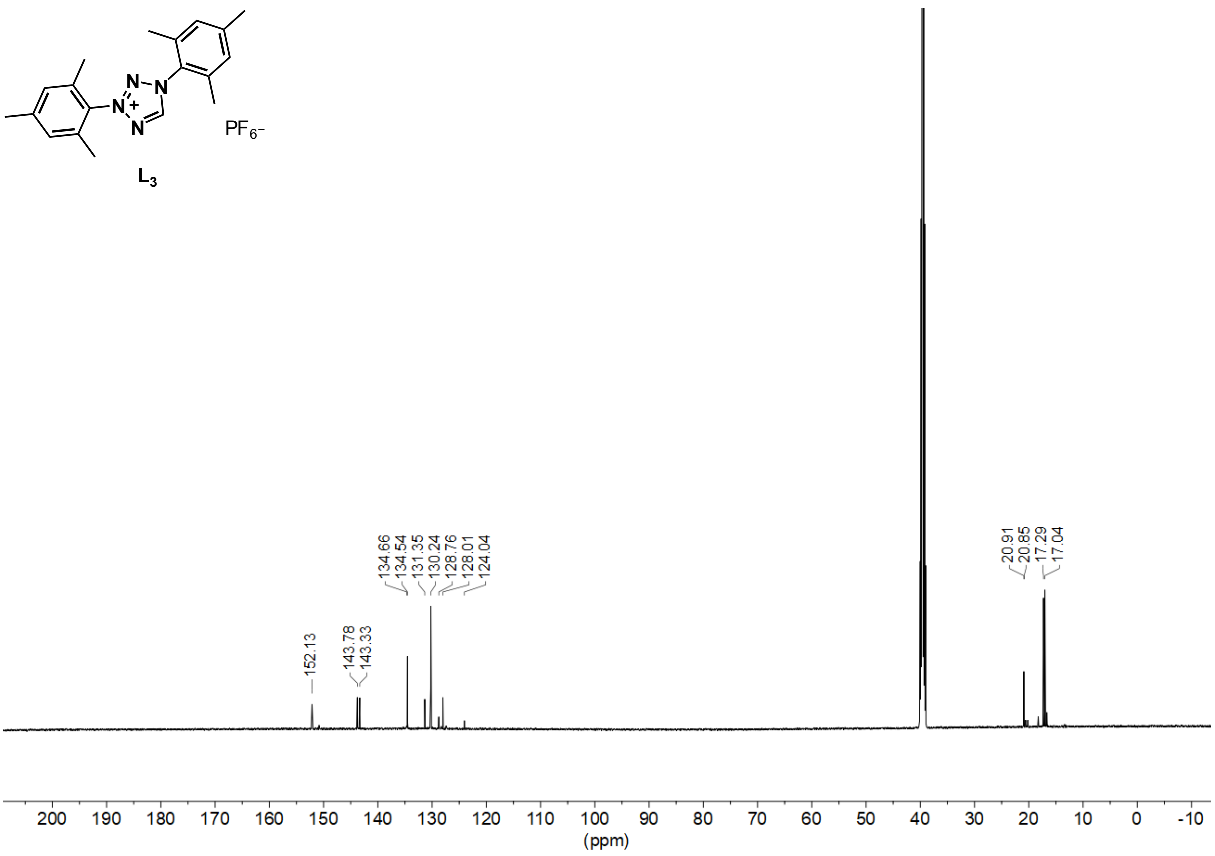

Figure S22: ^{13}C-NMR spectrum of **L$_3$** in MeCN-d_3.

3.20 3-Phenyl-1-(2,6-diisopropylphenyl)-1*H*-tetrazole-3-ium Tetrafluoroborate **L₄**

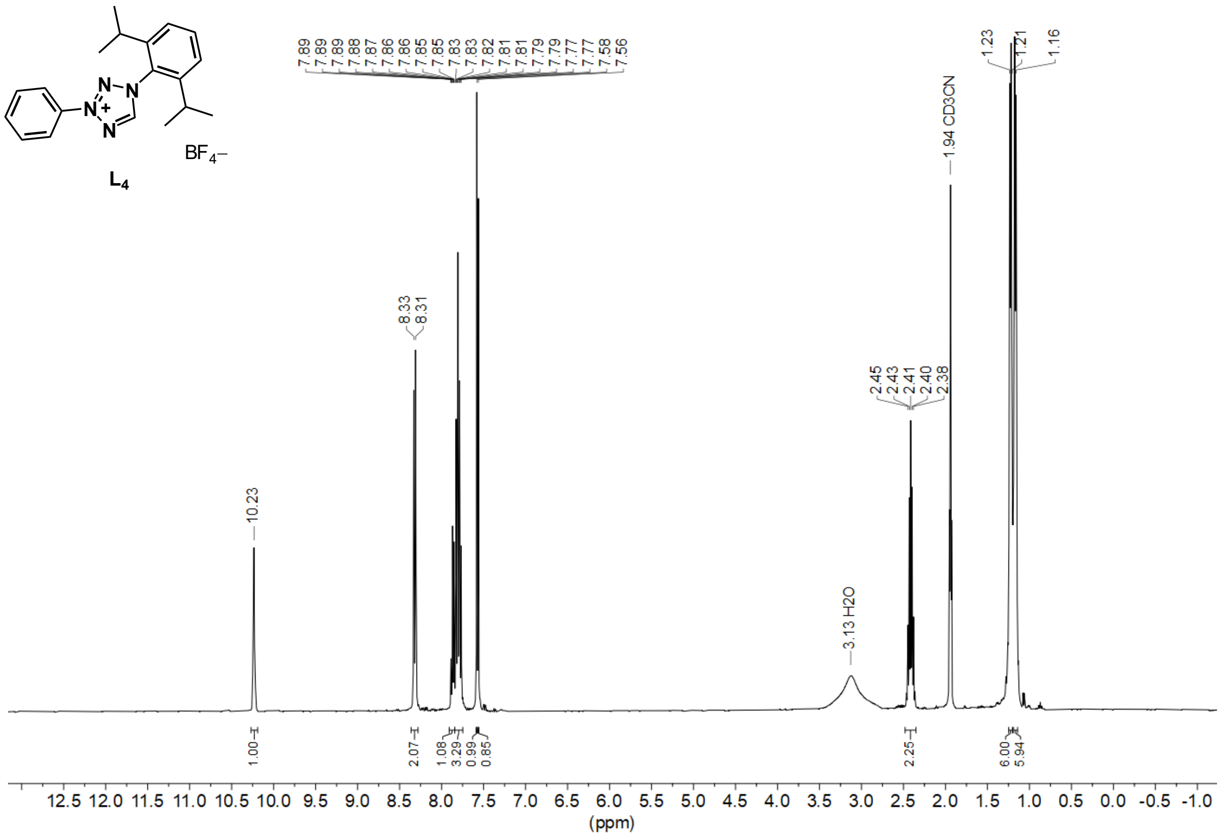

Figure S23: ^{1}H-NMR spectrum of **L$_4$** in MeCN-d_3.

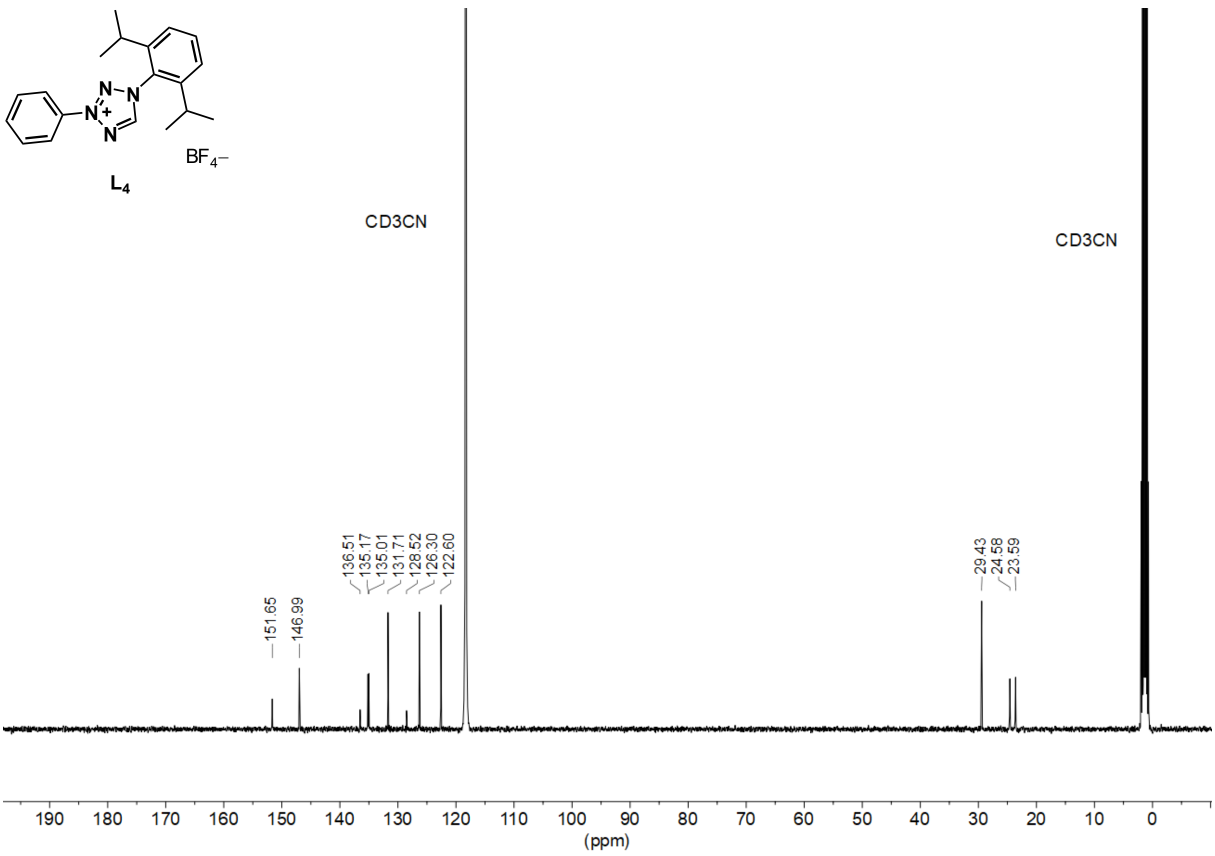

Figure S24: ^{13}C-NMR spectrum of **L_4** in MeCN-d_3.

3.21 Phenylbenzenediazonium Tetrafluoroborate A_5

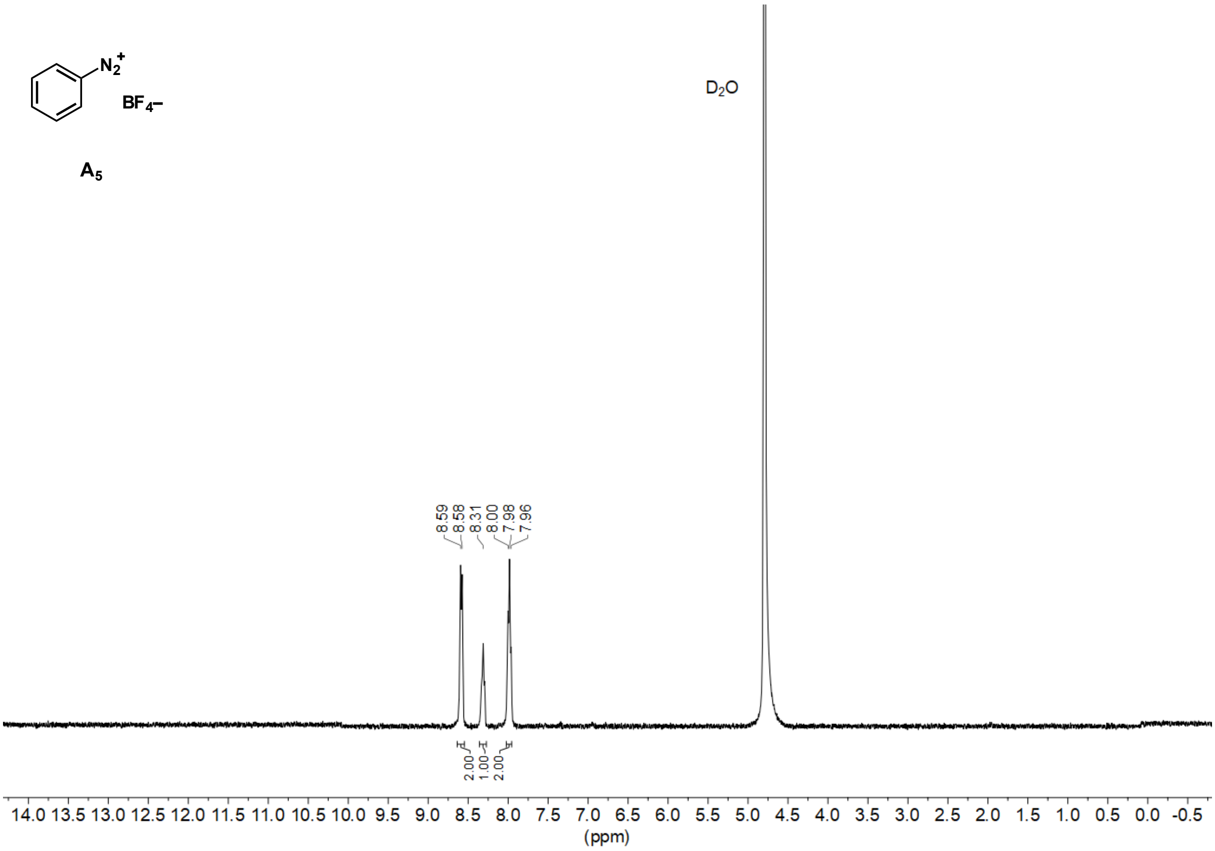

Figure S25: ^{1}H-NMR spectrum of **A_5** in D_2O.

3.22 4-Methylbenzenediazonium Tetrafluoroborate A_6

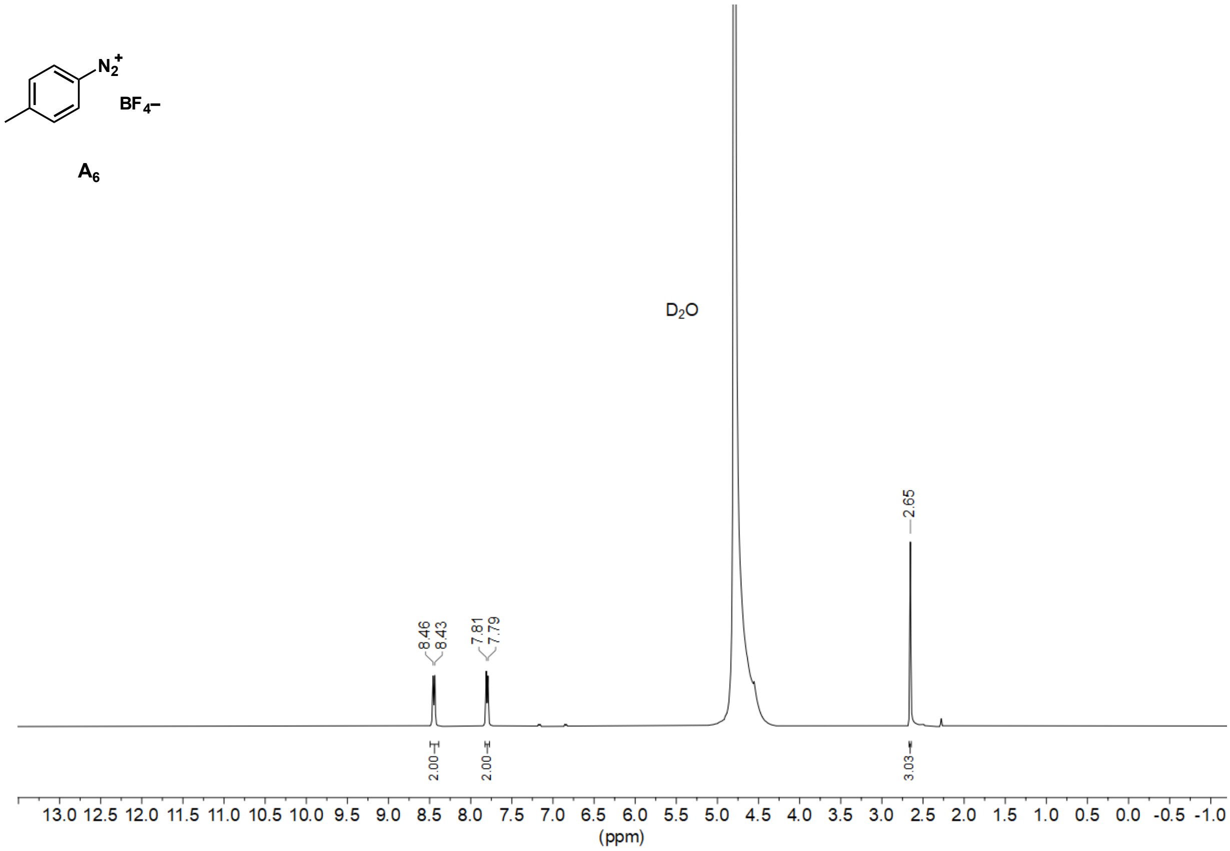

Figure S26: ^{1}H-NMR spectrum of **A_6** in D_2O.

3.23 2-Phenyl-2*H*-tetrazole B$_5$

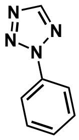

B$_5$

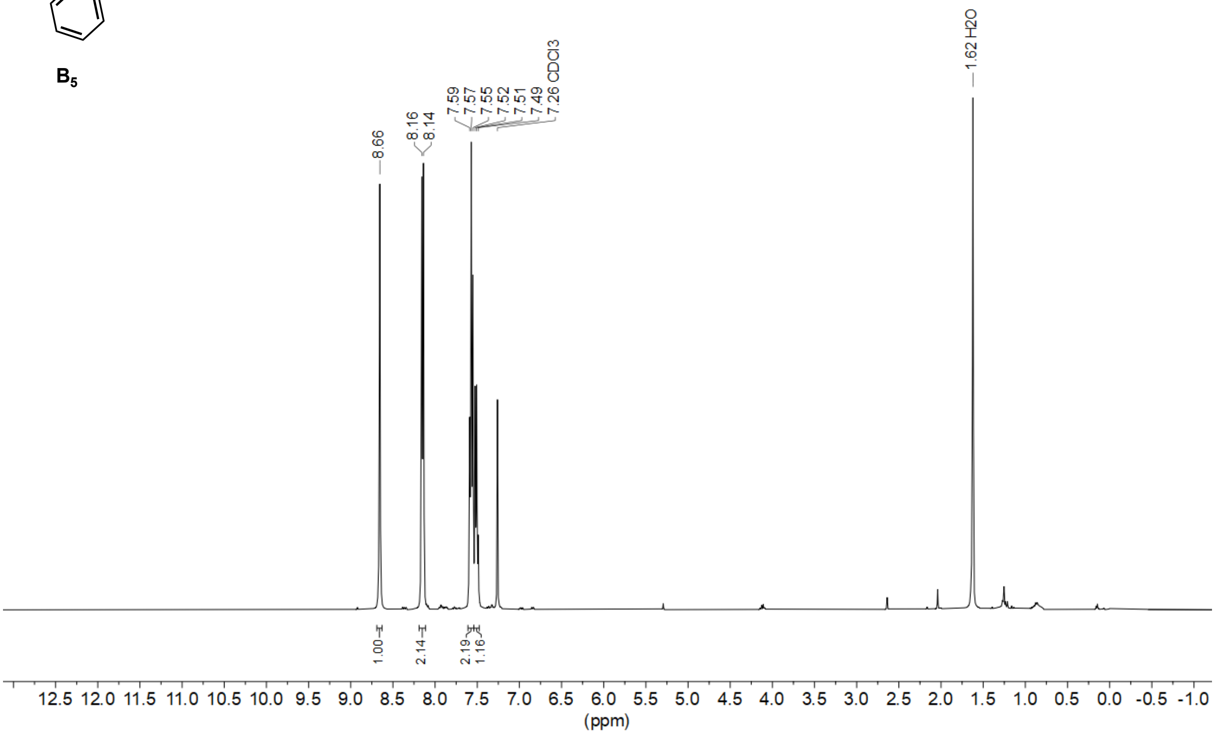

Figure S27: ^{1}H-NMR spectrum of **B$_5$** in $CDCl_3$.

3.24 2-(*p*-Tolyl)-2*H*-tetrazole B_6

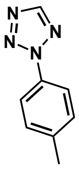

B_6

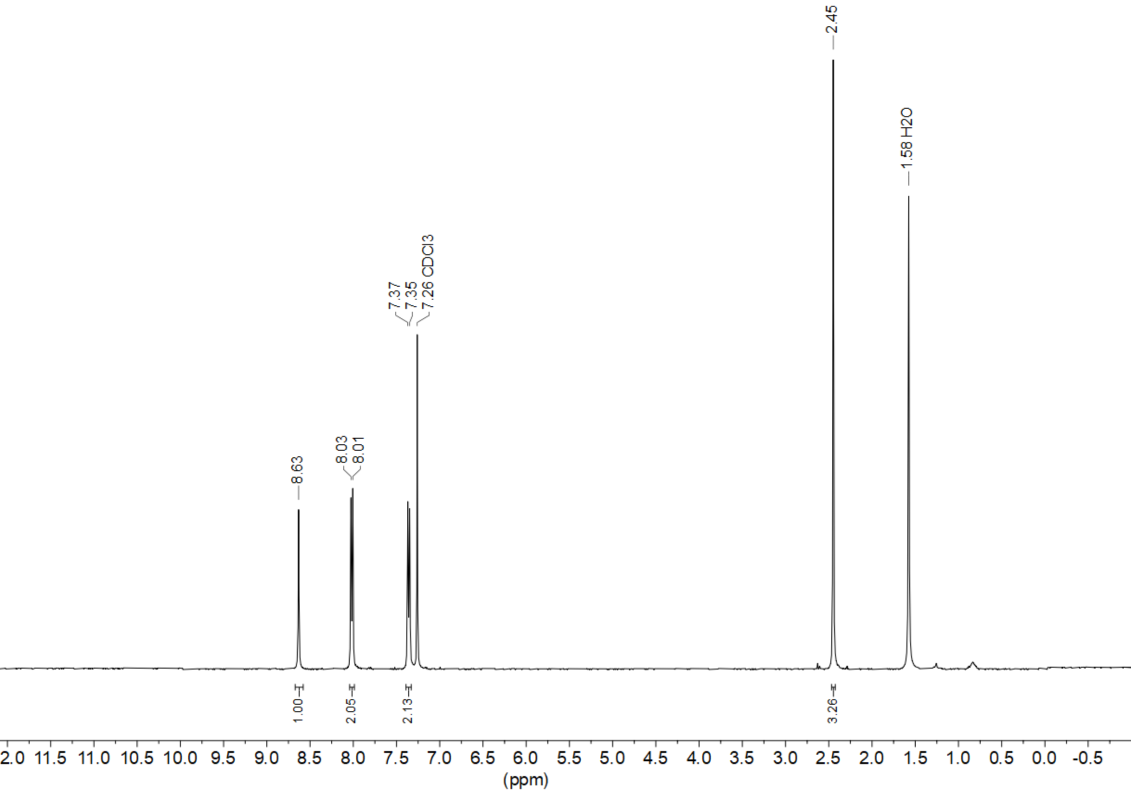

Figure S28: ^{1}H-NMR spectrum of **B_6** in $CDCl_3$.

3.25 1-Methyl-3-phenyl-2*H*-tetrazol-4-ium Tetrafluoroborate L_5

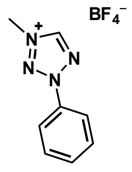

L5

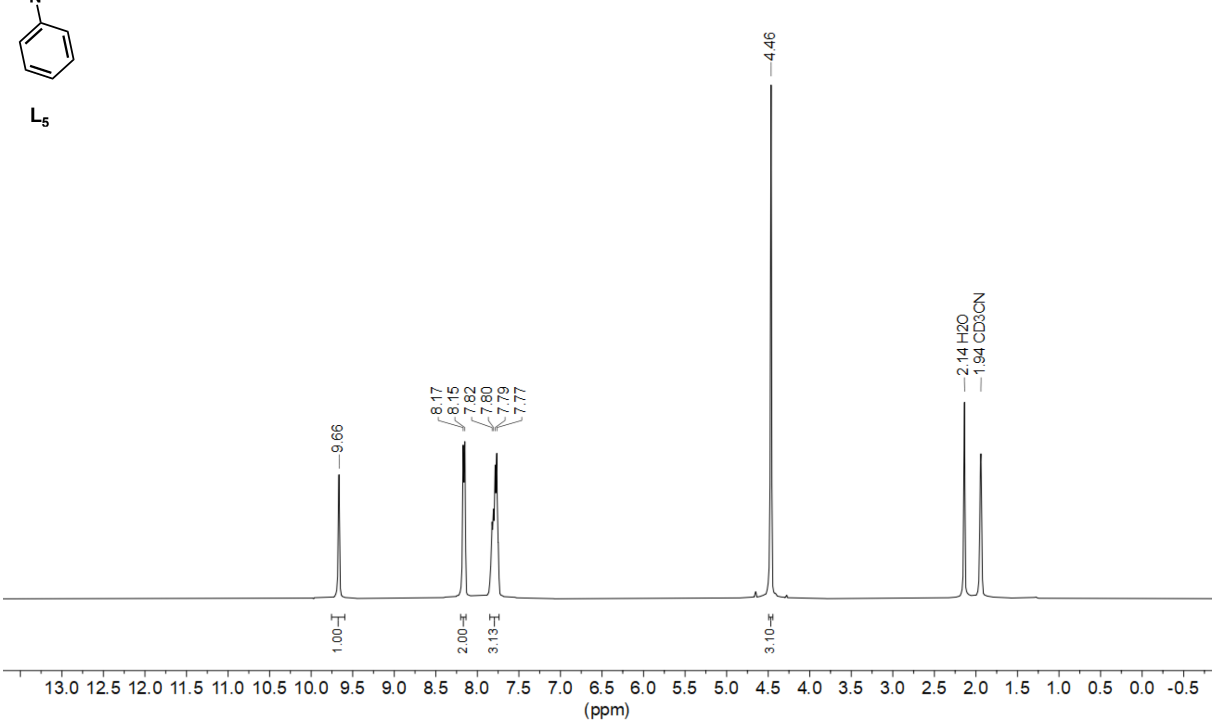

Figure S29: ^{1}H-NMR spectrum of $\mathbf{L_5}$ in MeCN-d_3.

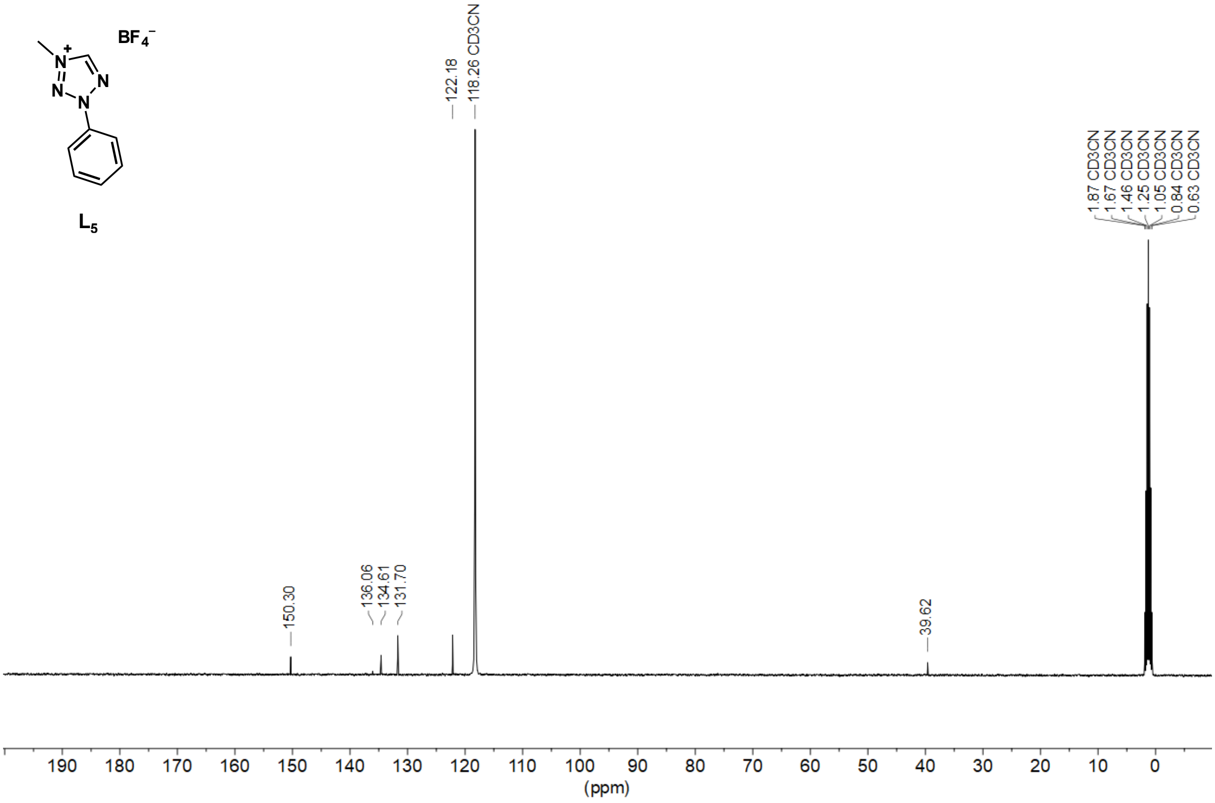

Figure S30: ^{13}C-NMR spectrum of **L_5** in MeCN-d_3.

3.26 1-Methyl-3-tolyl-2*H*-tetrazol-4-ium Tetrafluoroborate L6

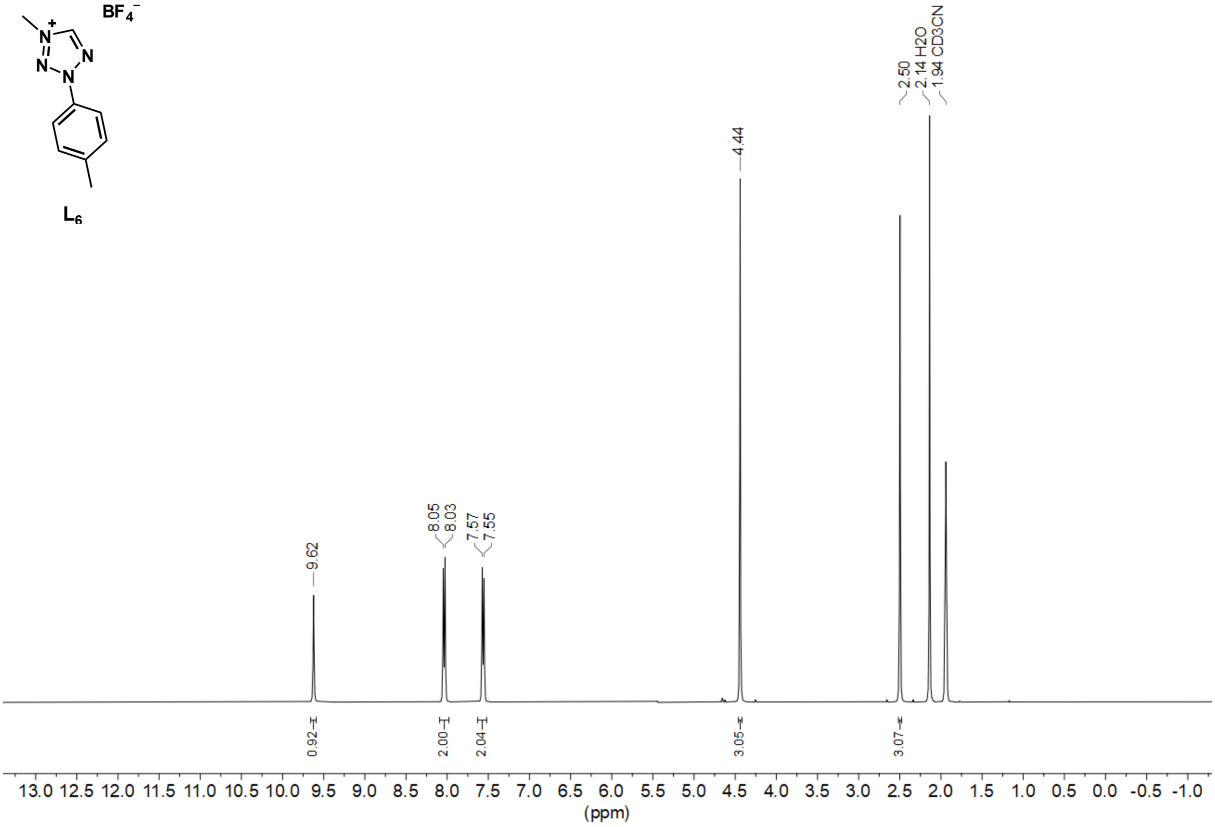

Figure S31: ^{1}H-NMR spectrum of **L6** in MeCN-d_3.

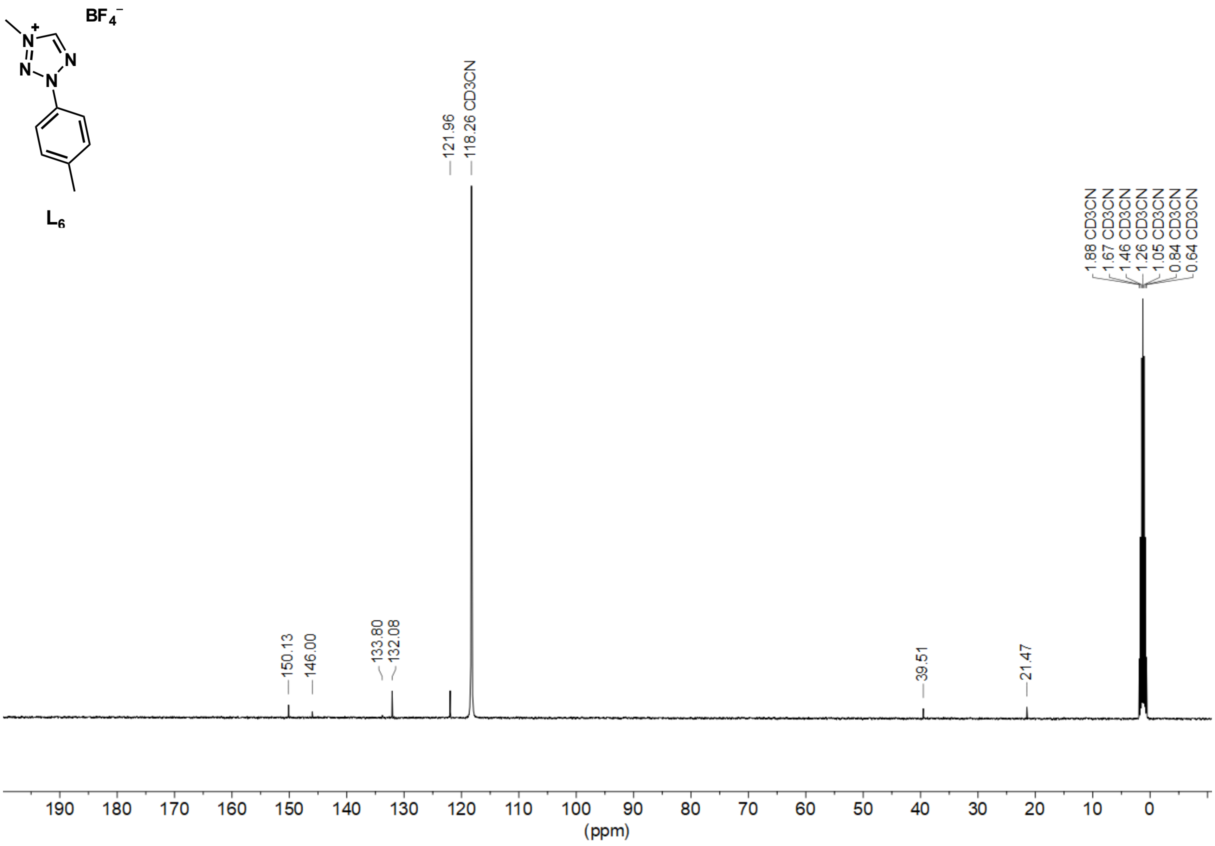

Figure S32: ^{13}C-NMR spectrum of **L6** in MeCN-d_3.

3.27 1*H*-Tetrazole A_7

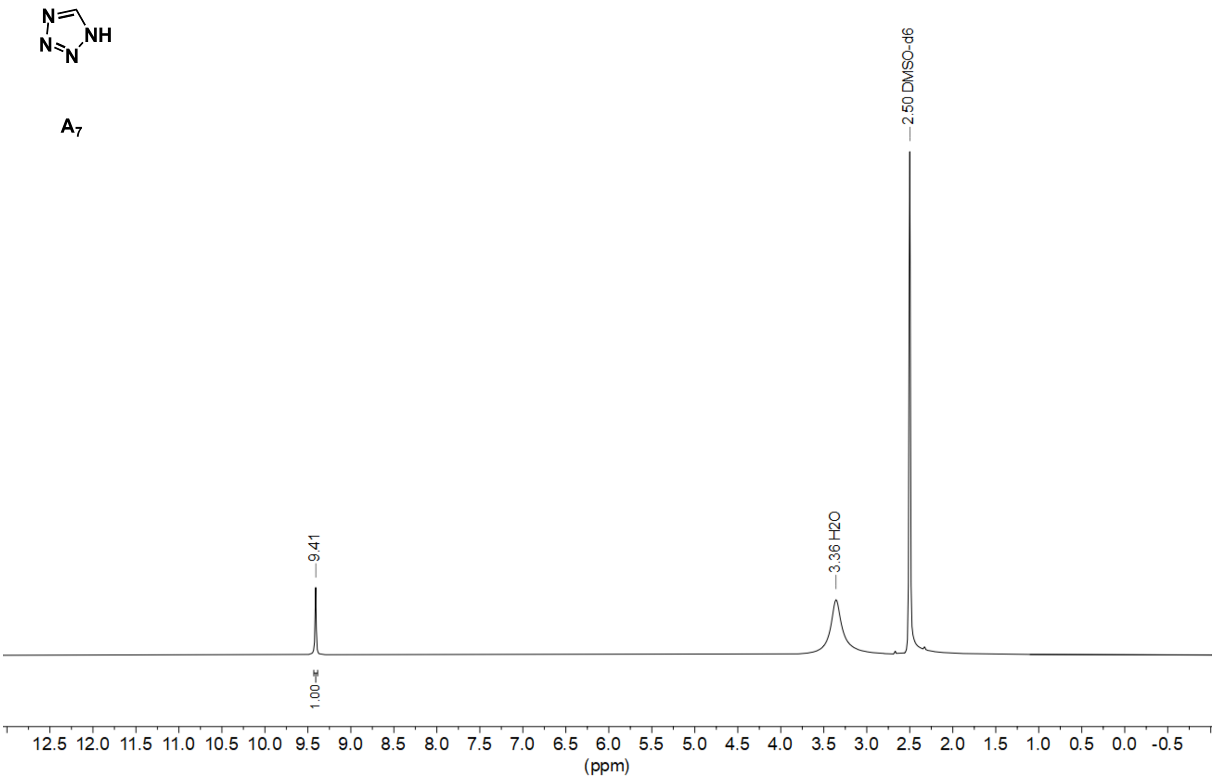

Figure S33: ^{1}H-NMR spectrum of $\mathbf{A_7}$ in DMSO-d_6.

3.28 2-Isopropyl-2*H*-tetrazole B_7

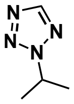

B_7

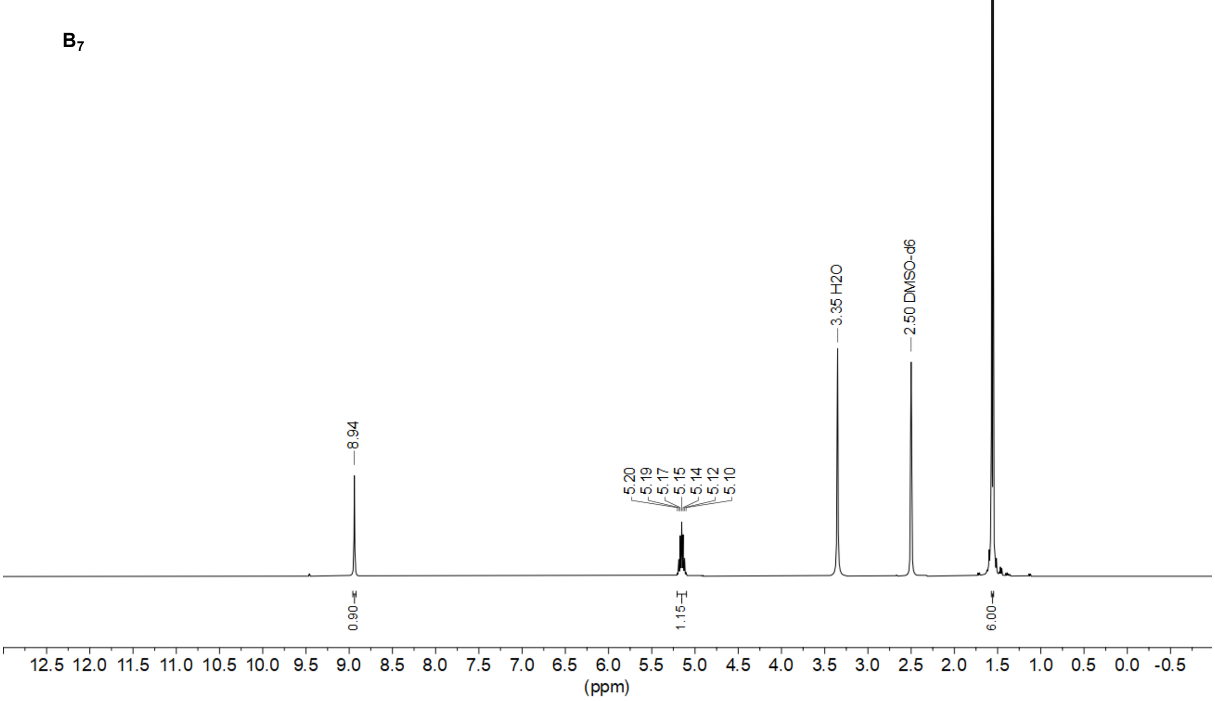

Figure S34: ^{1}H-NMR spectrum of **B_7** in DMSO-d_6.

3.29 1-*t*-Butyl-3-isopropyl-2*H*-tetrazolium Hexafluorophosphate L_7

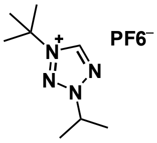

L_7

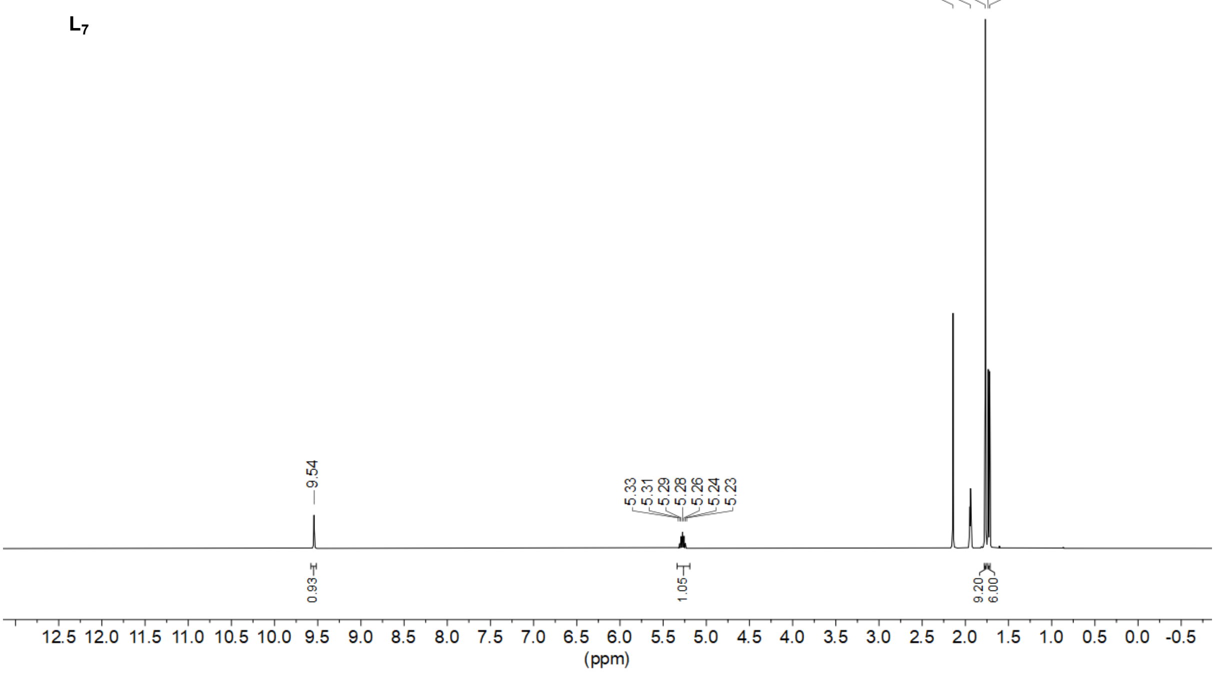

Figure S35: ^{1}H-NMR spectrum of $\mathbf{L_7}$ in MeCN-d_3.

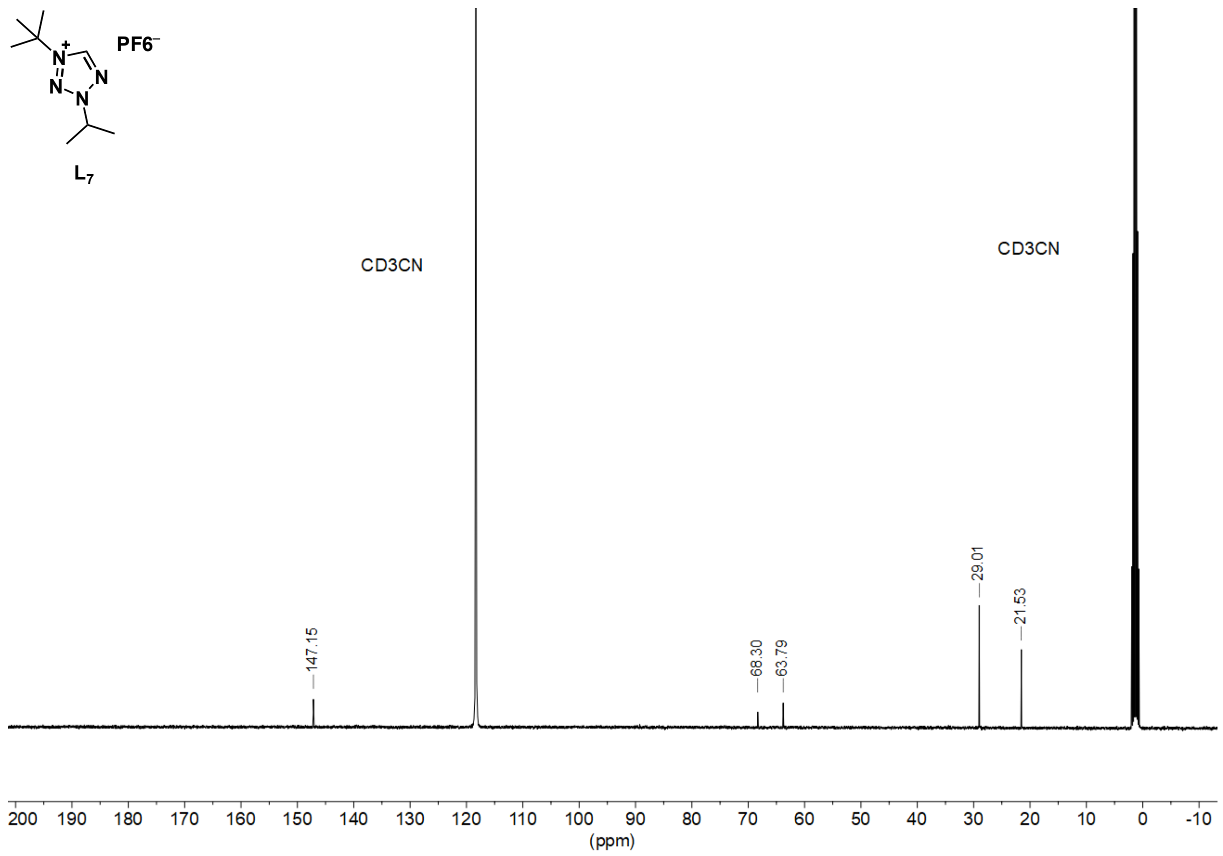

Figure S36: ^{13}C-NMR spectrum of **L$_7$** in MeCN-*d$_3$*.

3.30 1,3-Diphenyl-tetrazolylidene Gold(I) Chloride 1

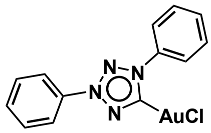

1

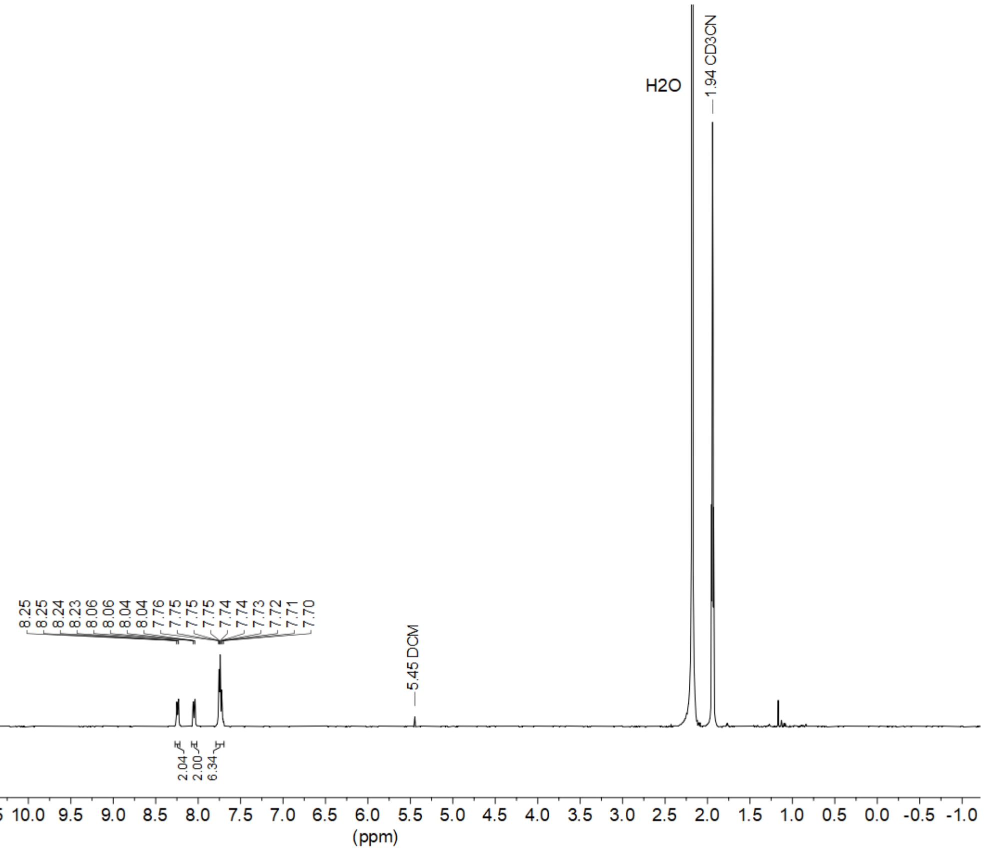

Figure S37: ^{1}H-NMR spectrum of **1** in MeCN-d_3.

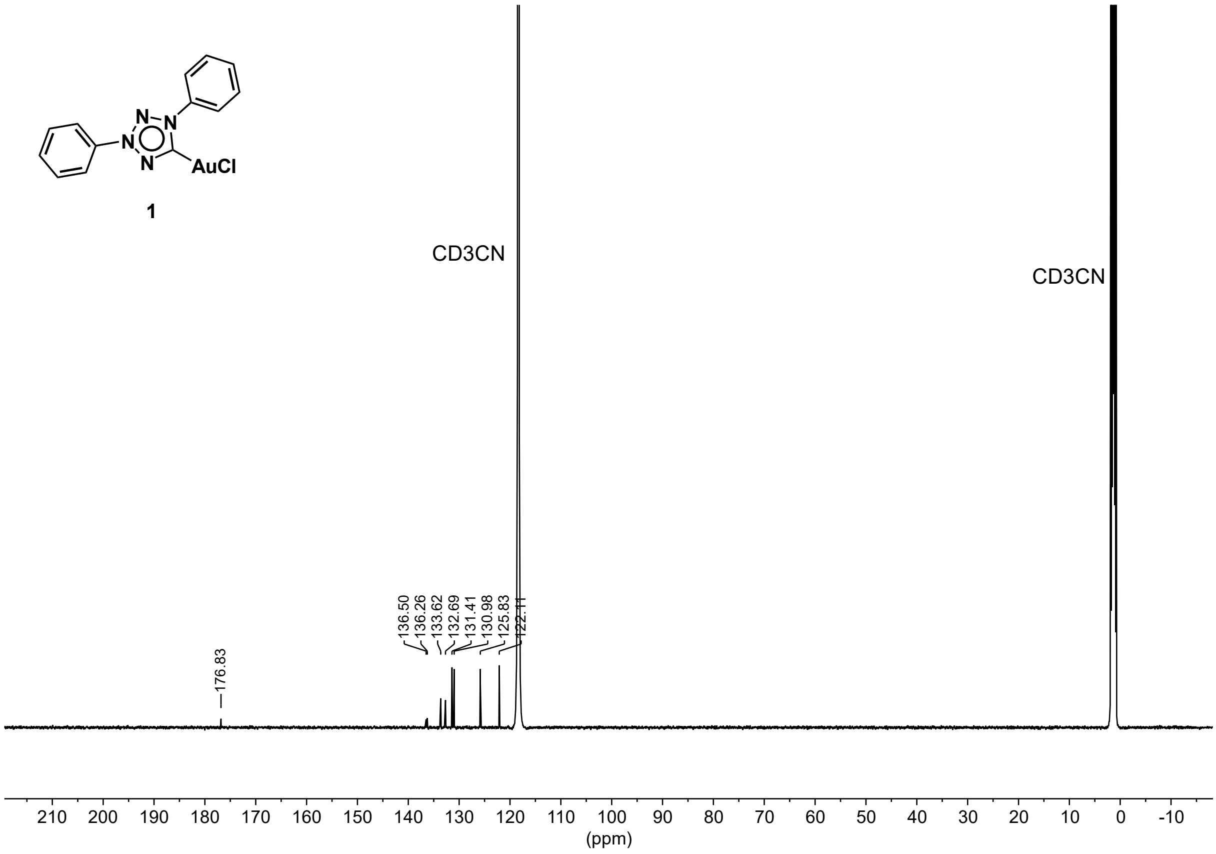

Figure S38: ^{13}C-NMR spectrum of **1** in MeCN-d_3.

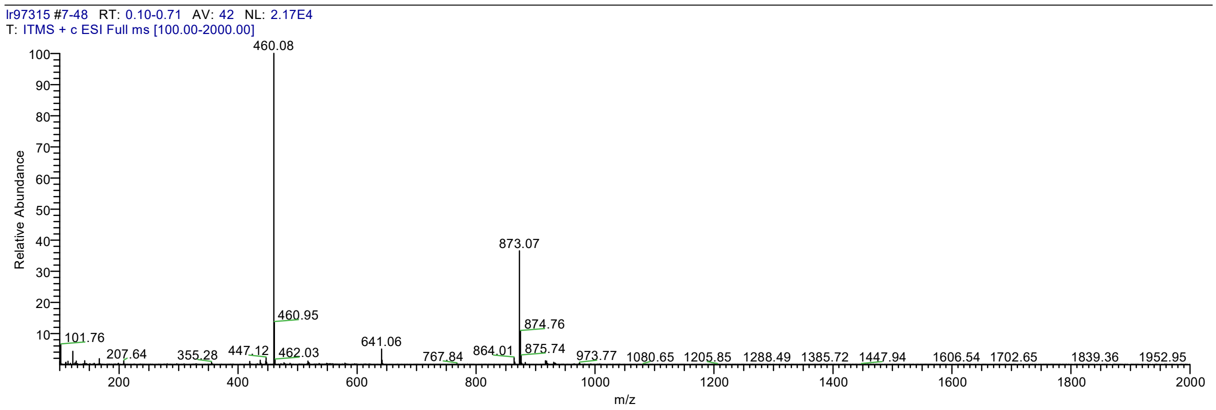

Figure S39: ESI mass spectrum of **1** in MeCN as solvent.

3.31 1-(*p*-Tolyl)-3-phenyl-tetrazolylidene Gold(I) Chloride 2

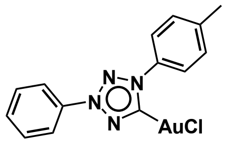

2

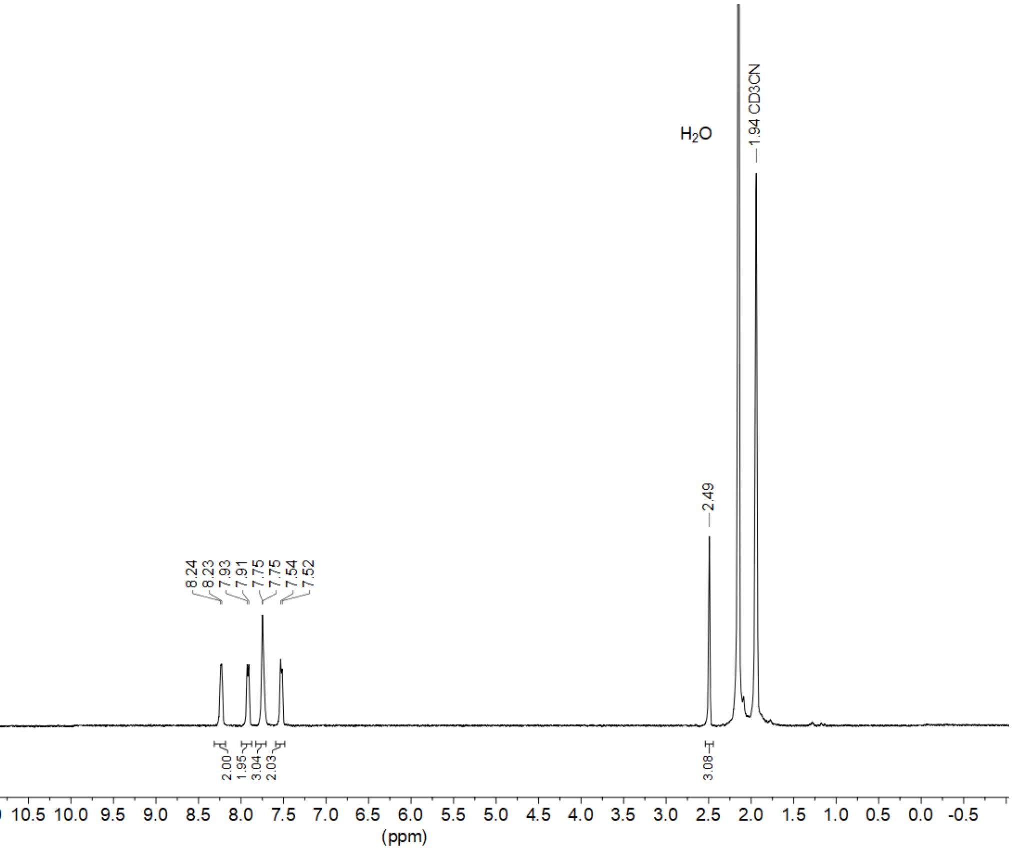

Figure S40: ^{1}H-NMR spectrum of **2** in MeCN-d_3.

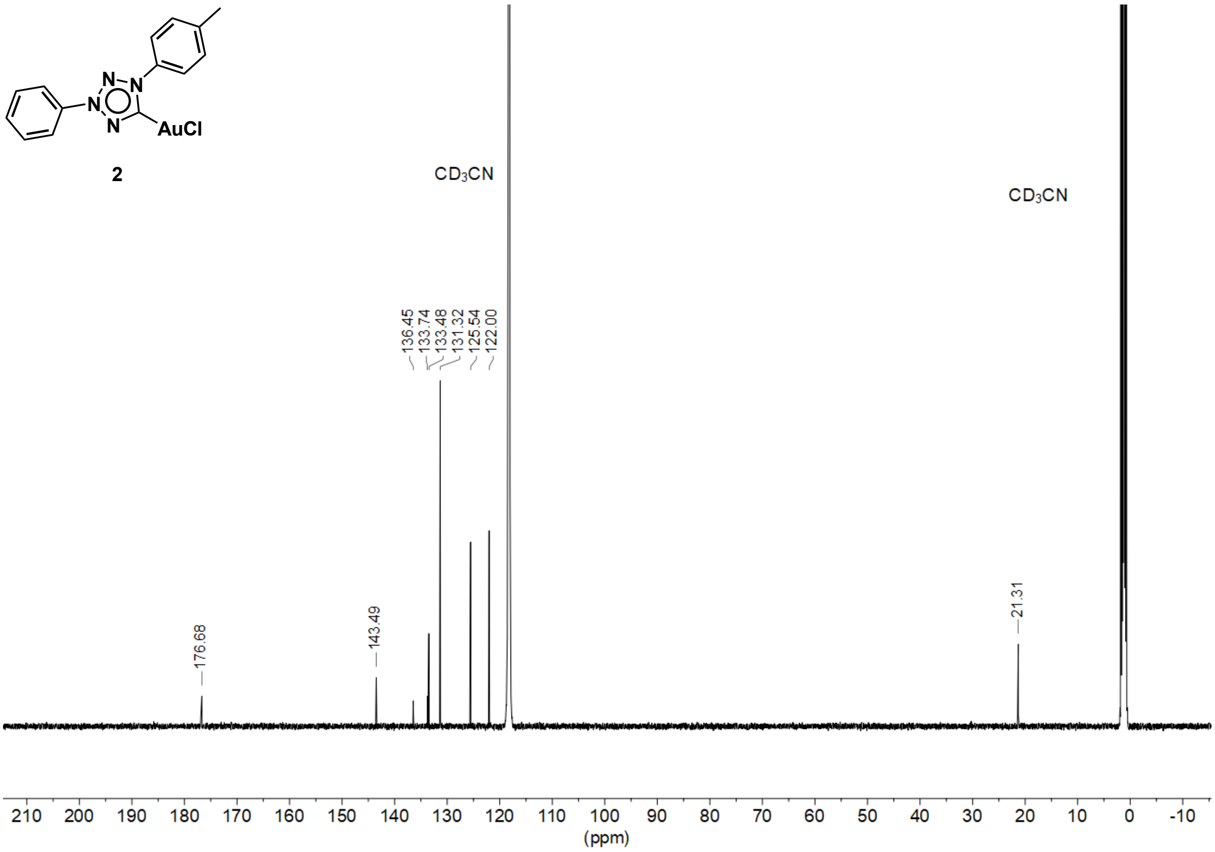

Figure S41: ^{13}C-NMR spectrum of **2** in MeCN-d_3.

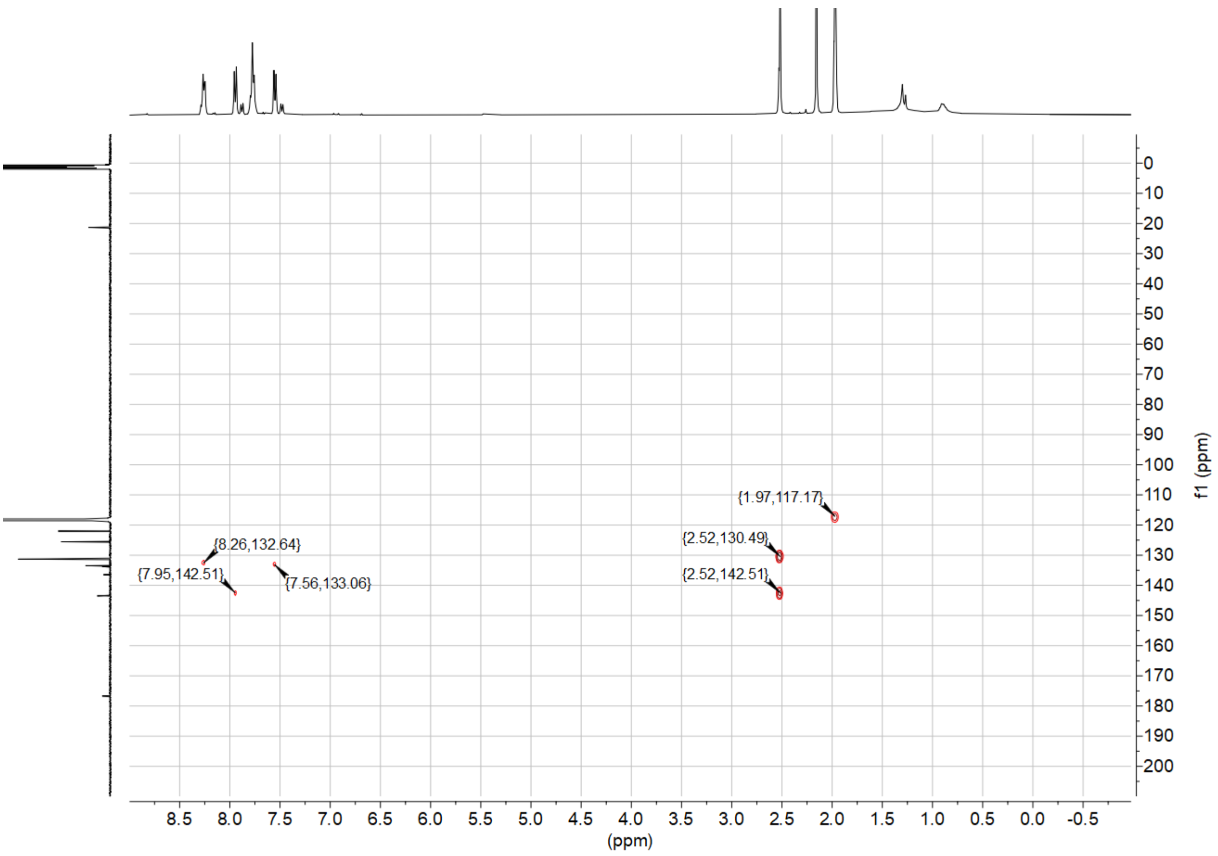

Figure S42: HMBC spectrum of **2** in MeCN-d_3.

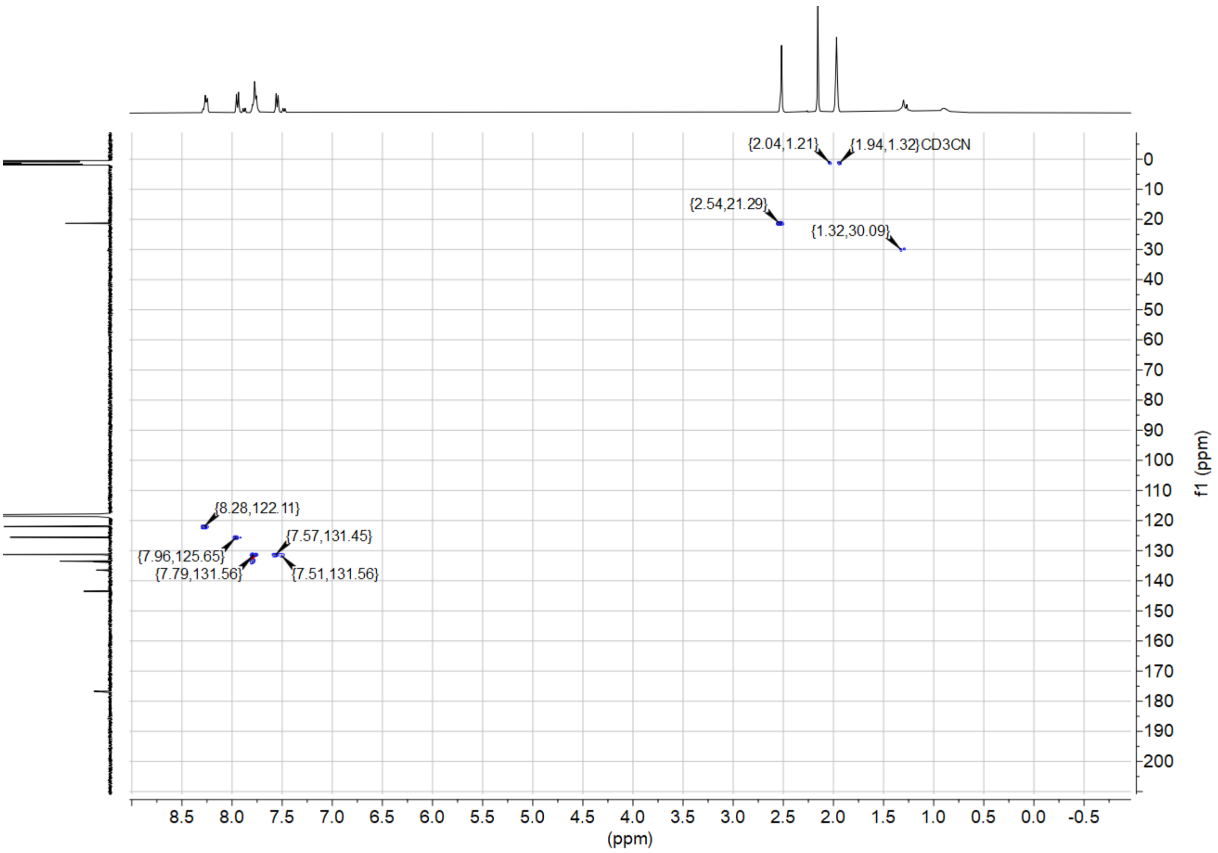

Figure S43: HSQC spectrum of **2** in MeCN-d_3.

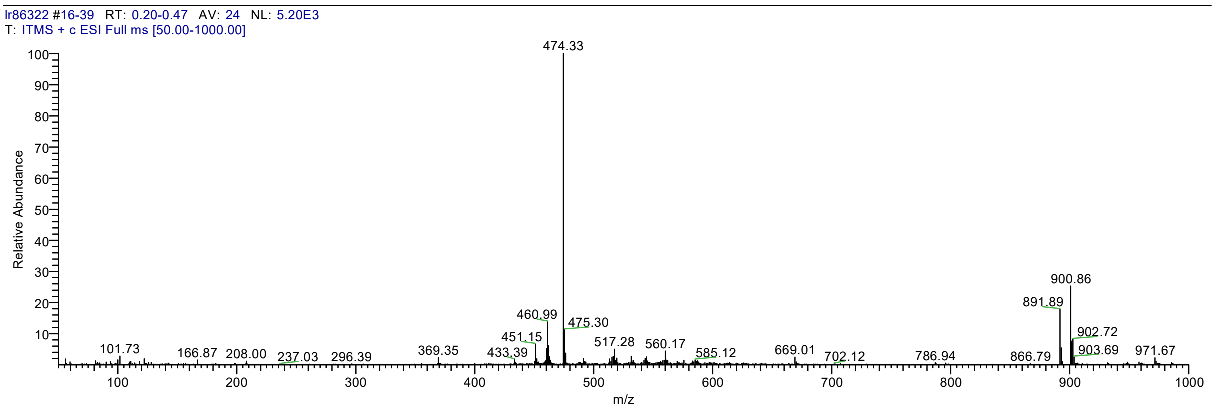

Figure S44: ESI mass spectrum of **2** in MeCN as solvent.

3.32 1,3-Dimesityl-tetrazolylidene Gold(I) Chloride 3

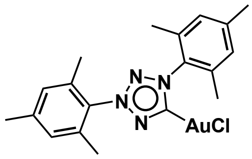

3

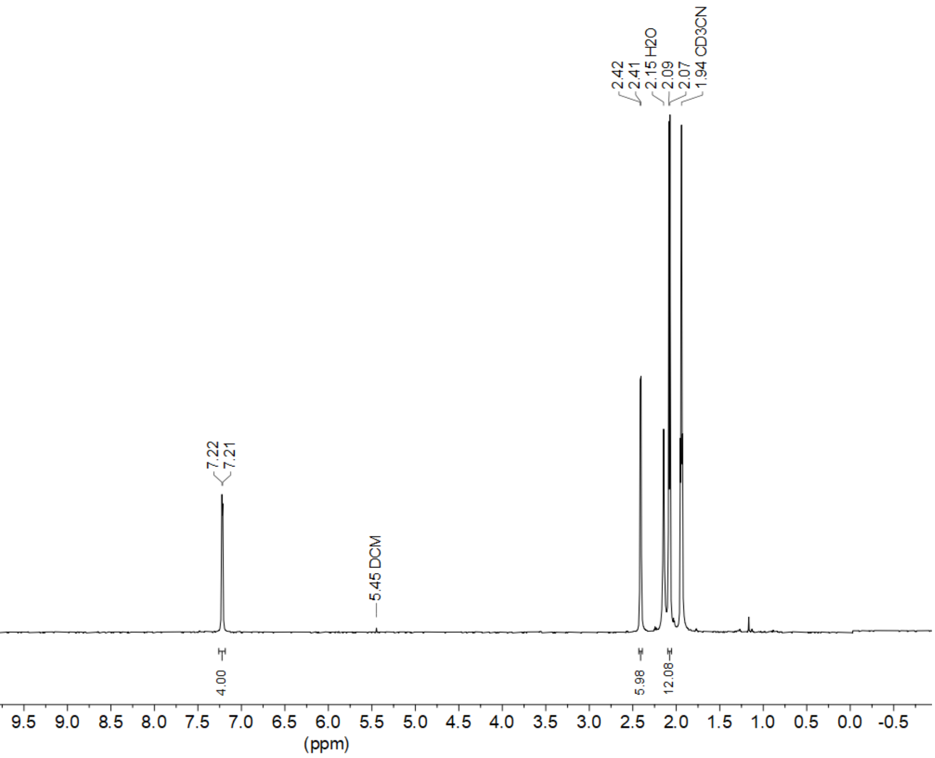

Figure S45: ^{1}H-NMR spectrum of **3** in MeCN-d_3.

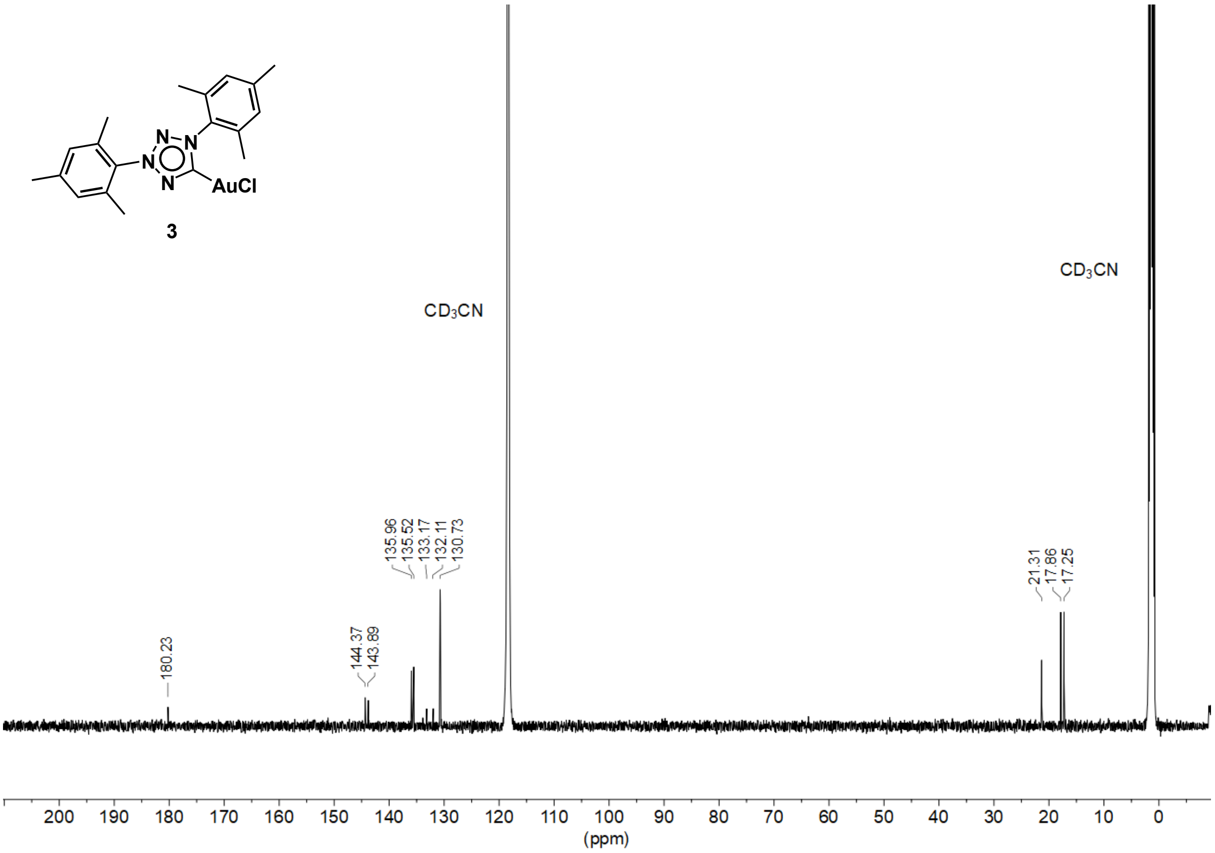

Figure S46: ^{13}C-NMR spectrum of **3** in MeCN-d_3.

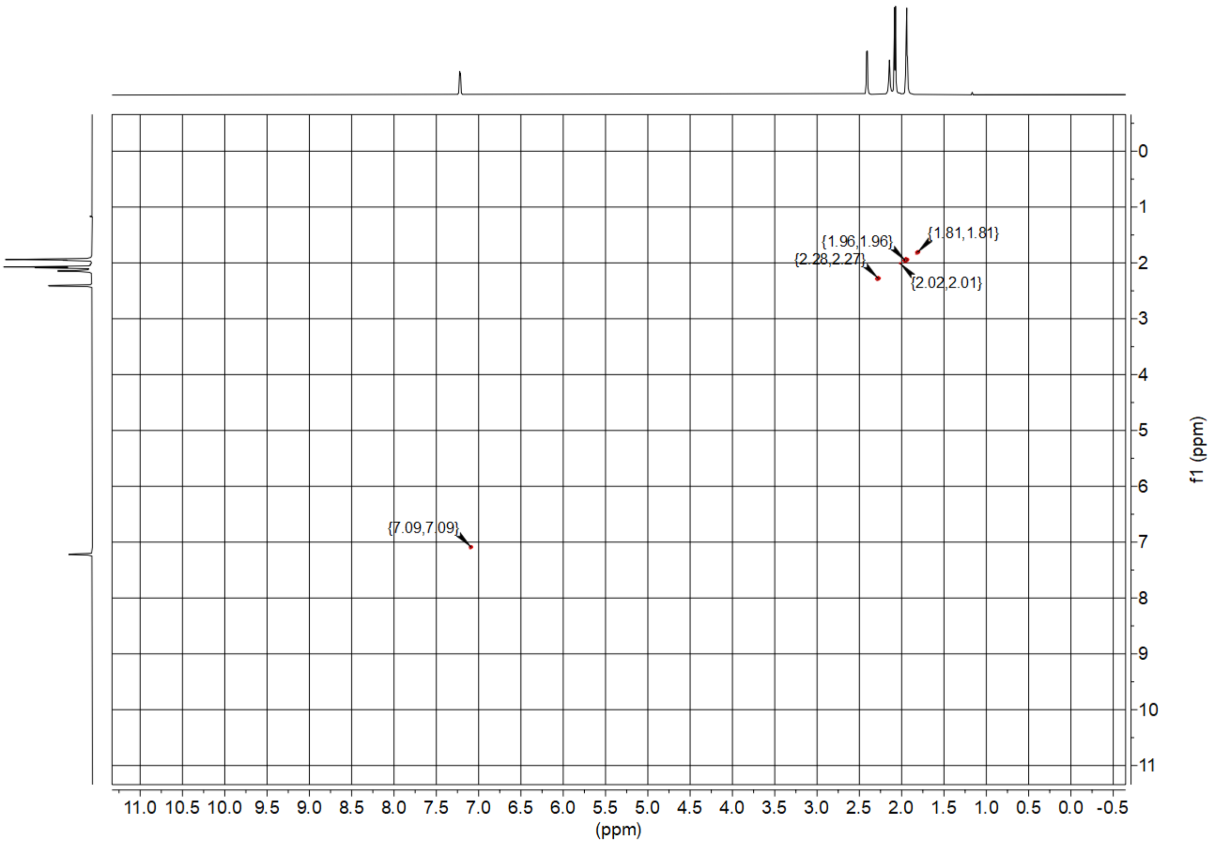

Figure S47: COSY spectrum of **3** in MeCN-d_3.

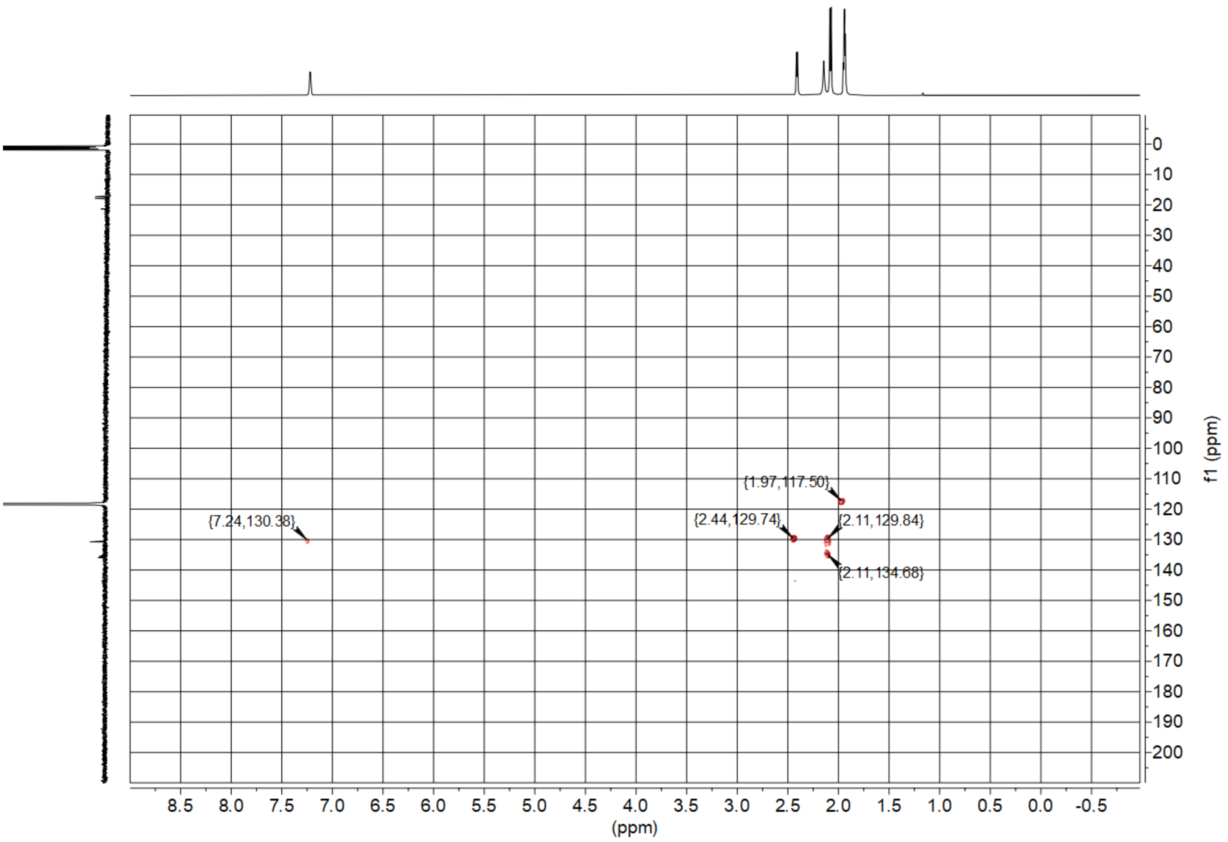

Figure S48: HMBC spectrum of **3** in MeCN-d_3.

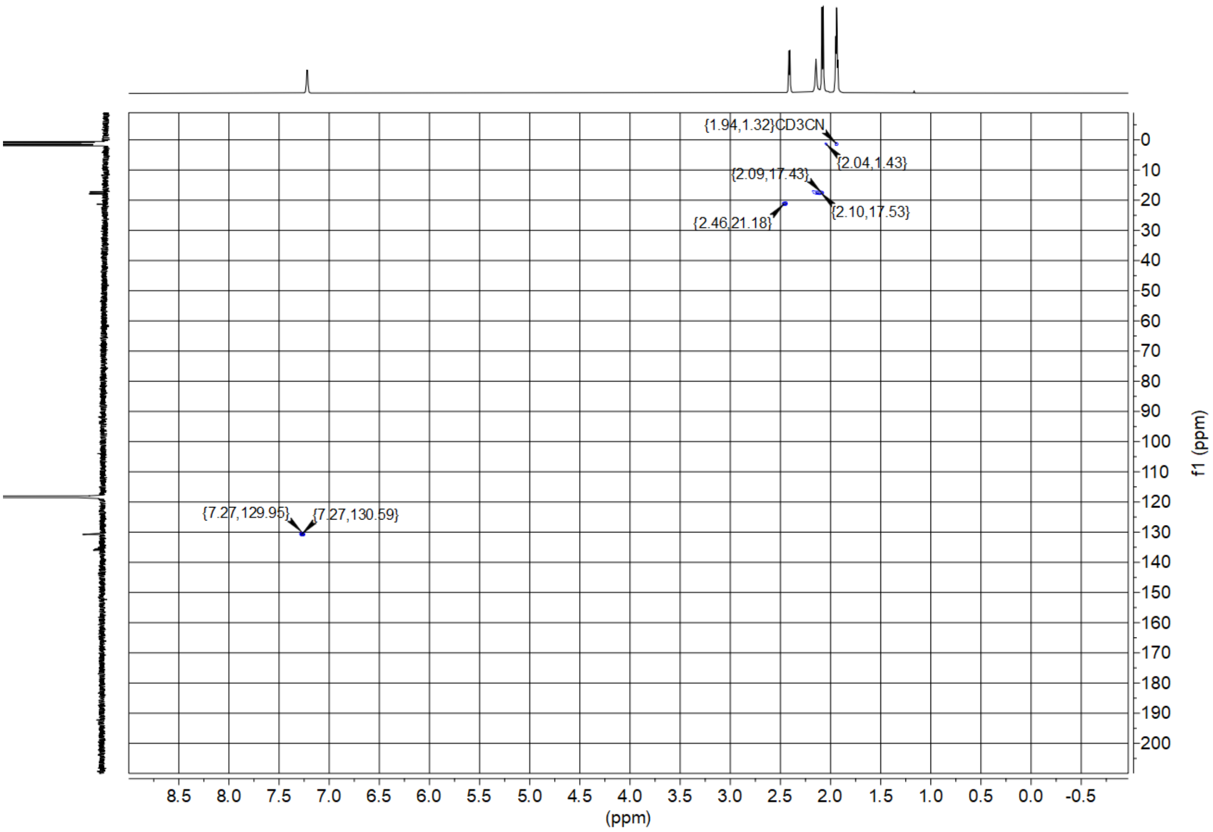

Figure S49: HSQC spectrum of **3** in MeCN-d_3.

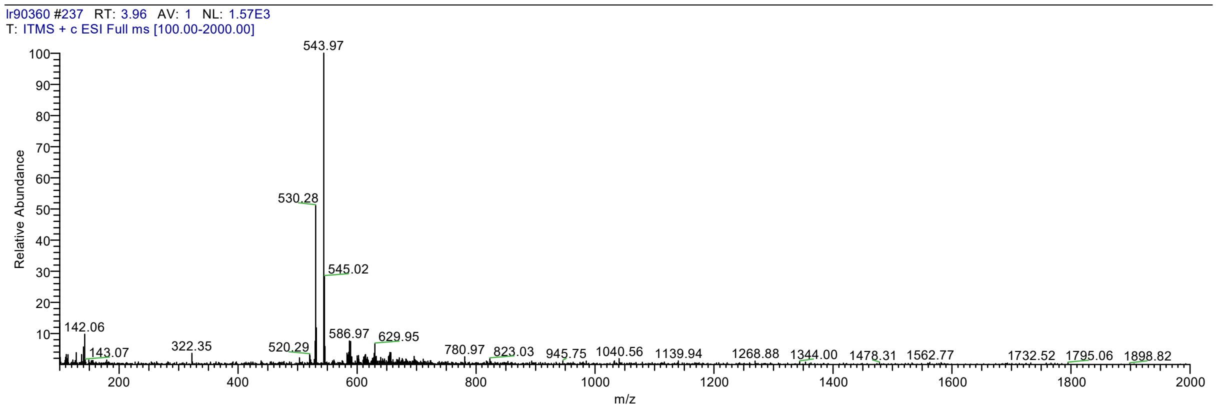

Figure S50: ESI mass spectrum of **3** in MeCN as solvent.

3.33 1-(2,6-Diisopropylphenyl)-3-phenyl-tetrazolylidene Gold(I) Chloride 4

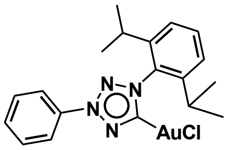

4

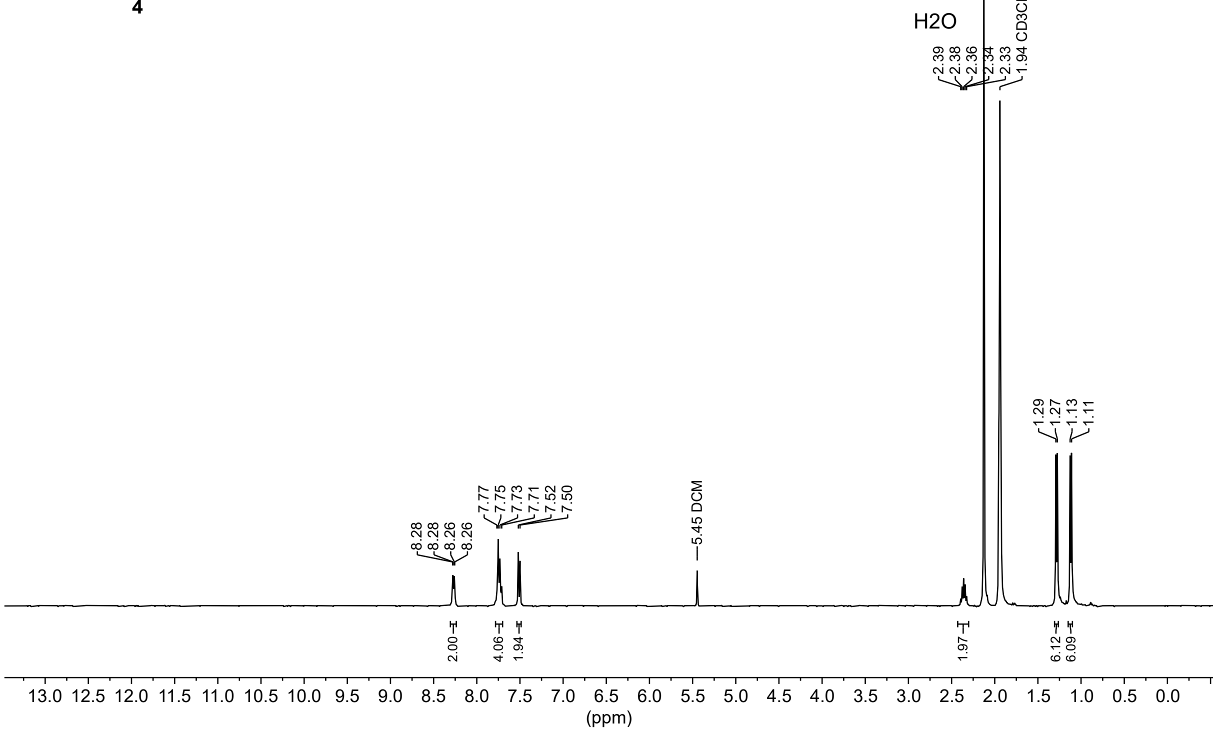

Figure S51: ^{1}H-NMR spectrum of **4** in MeCN-d_3.

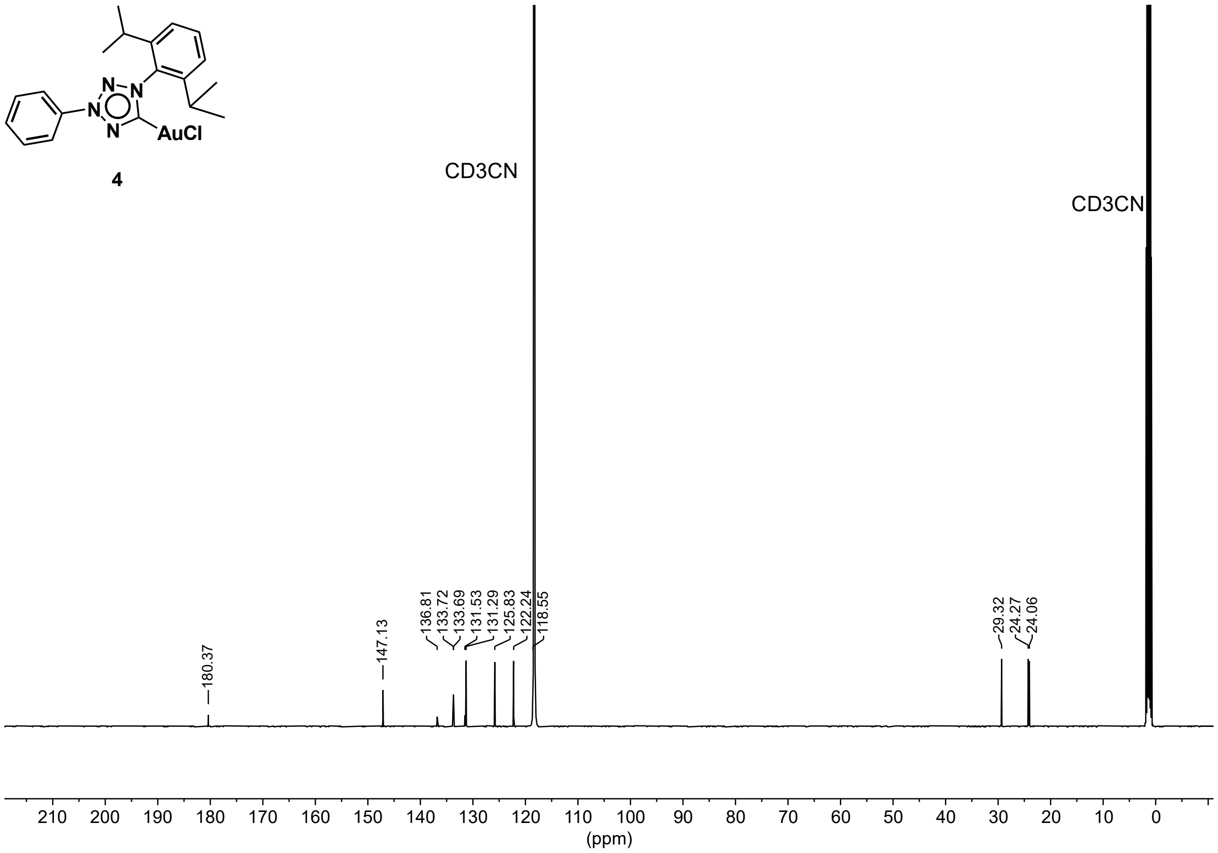

Figure S52: ^{13}C-NMR spectrum of **4** in MeCN-d_3.

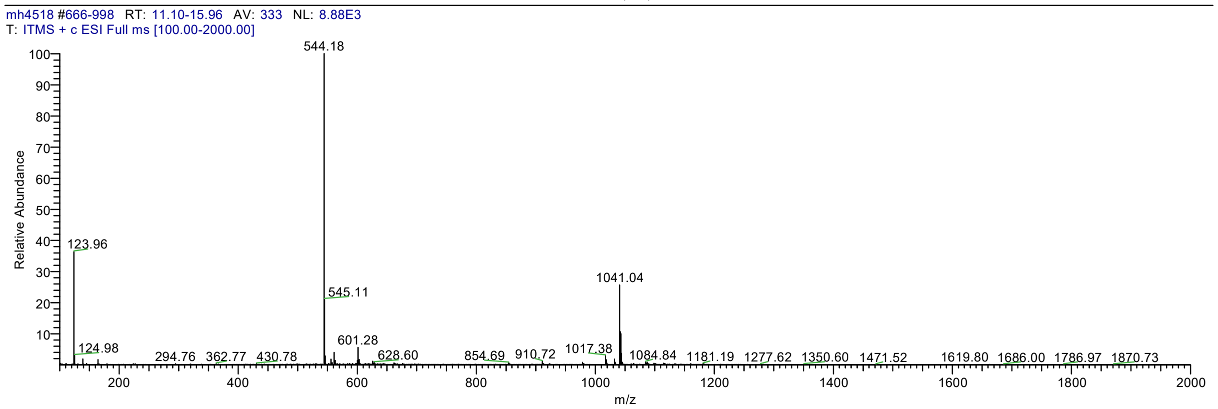

Figure S53: ESI mass spectrum of **4** in MeCN as solvent.

3.34 1-Methyl-3-phenyl-tetrazolylidene Gold(I) Chloride 5

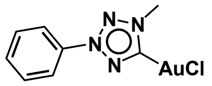

5

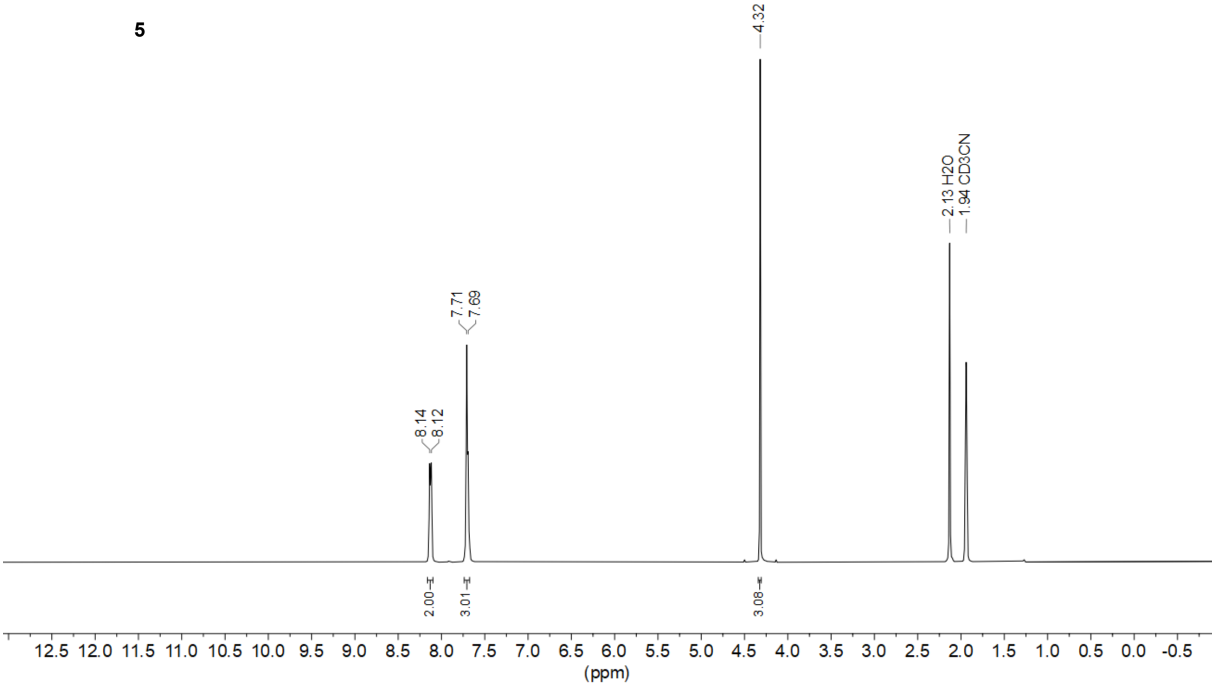

Figure S54: ^{1}H-NMR spectrum of **5** in MeCN-d_3.

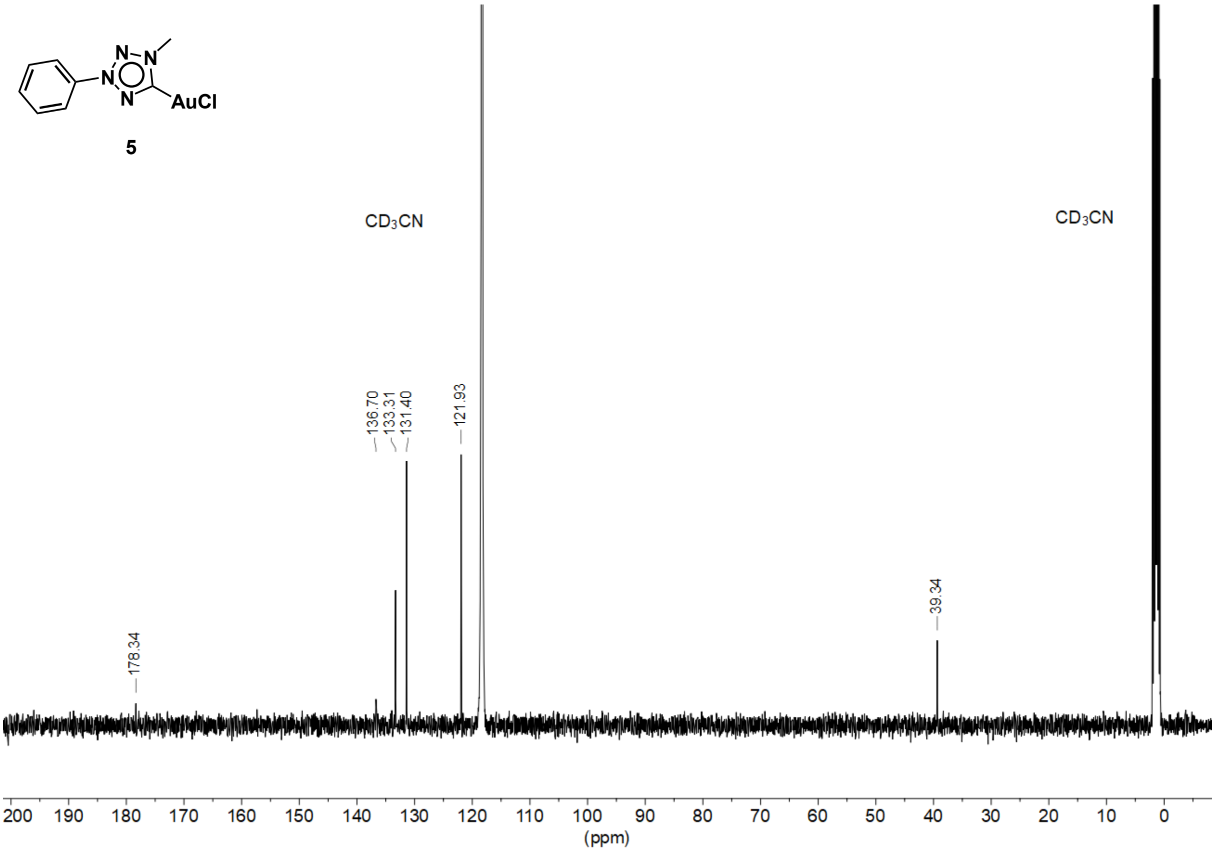

Figure S55: ^{13}C-NMR spectrum of **5** in MeCN-d_3.

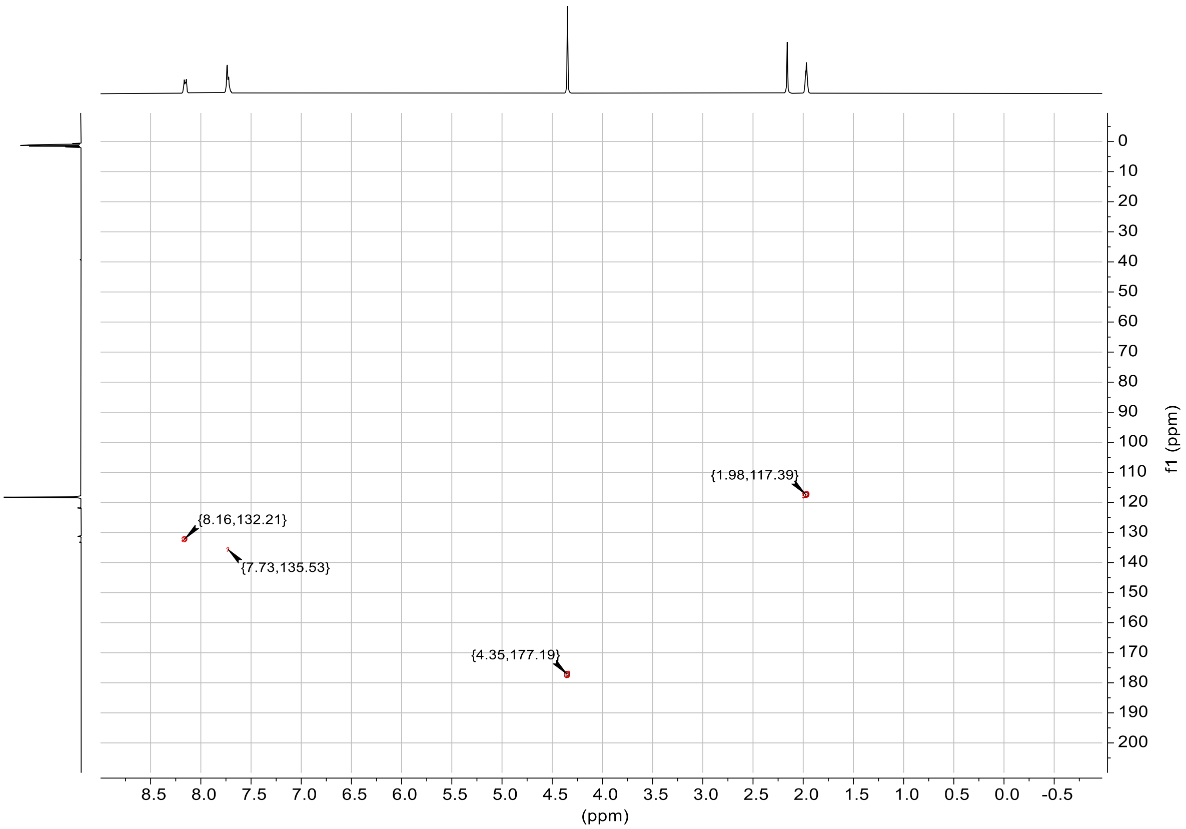

Figure S56: HMBC spectrum of **5** in MeCN-d_3.

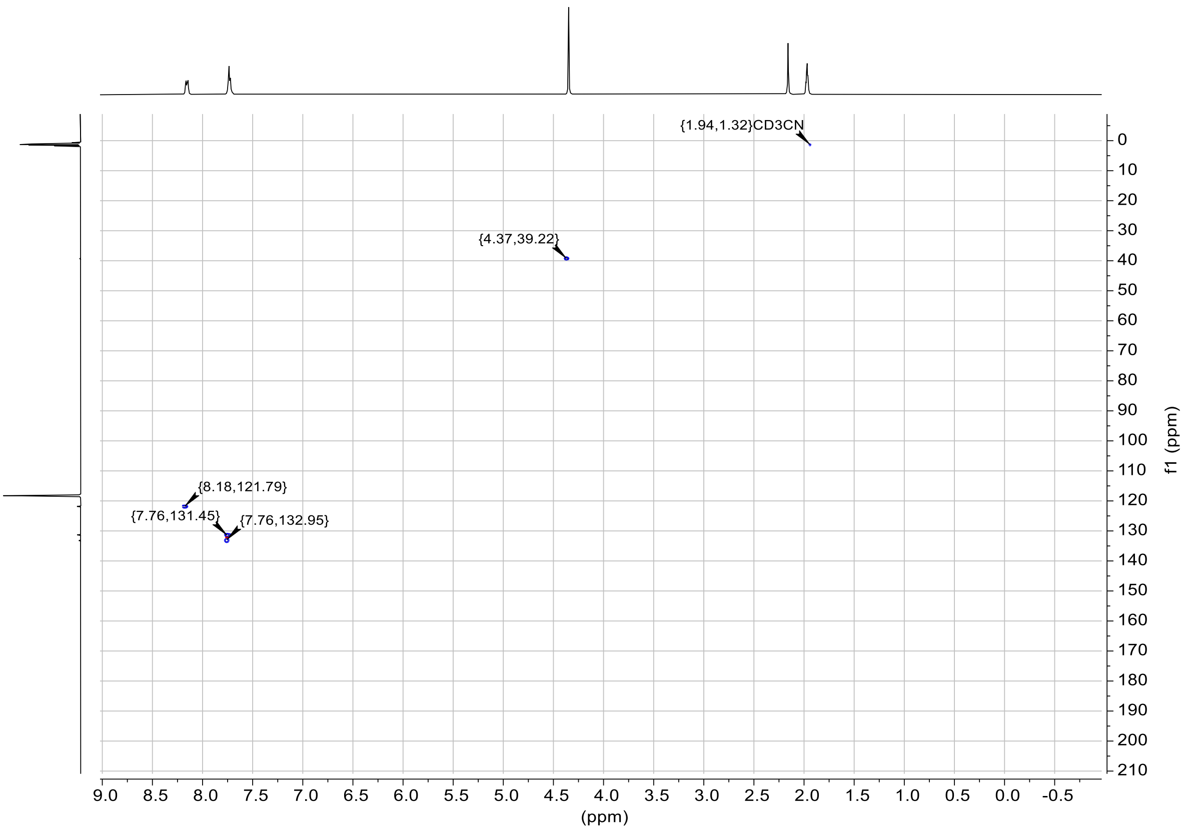

Figure S57: HSQC spectrum of **5** in MeCN-d_3.

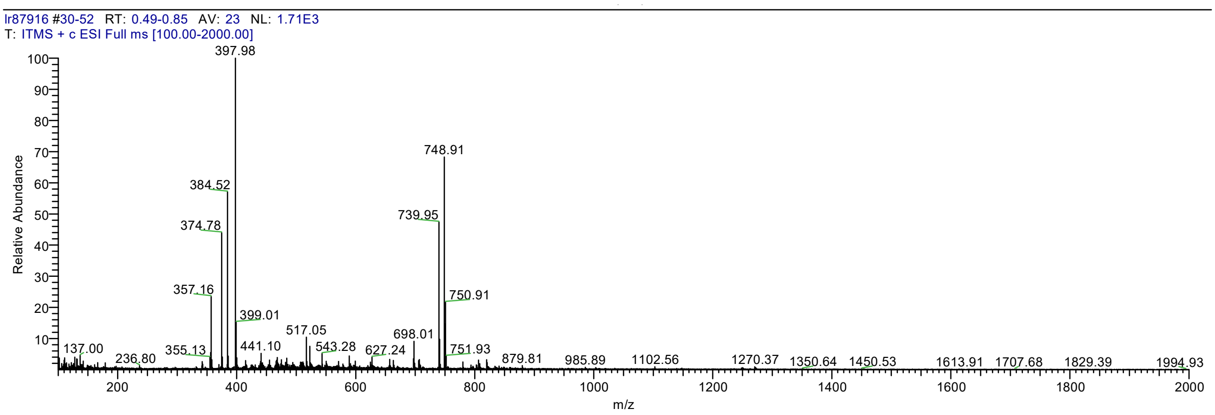

Figure S58: ESI mass spectrum of **5** in MeCN as solvent.

3.35 1-Methyl-3-*p*-tolyl-tetrazolylidene Gold(I) Chloride 6

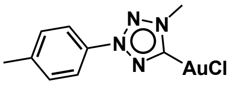

6

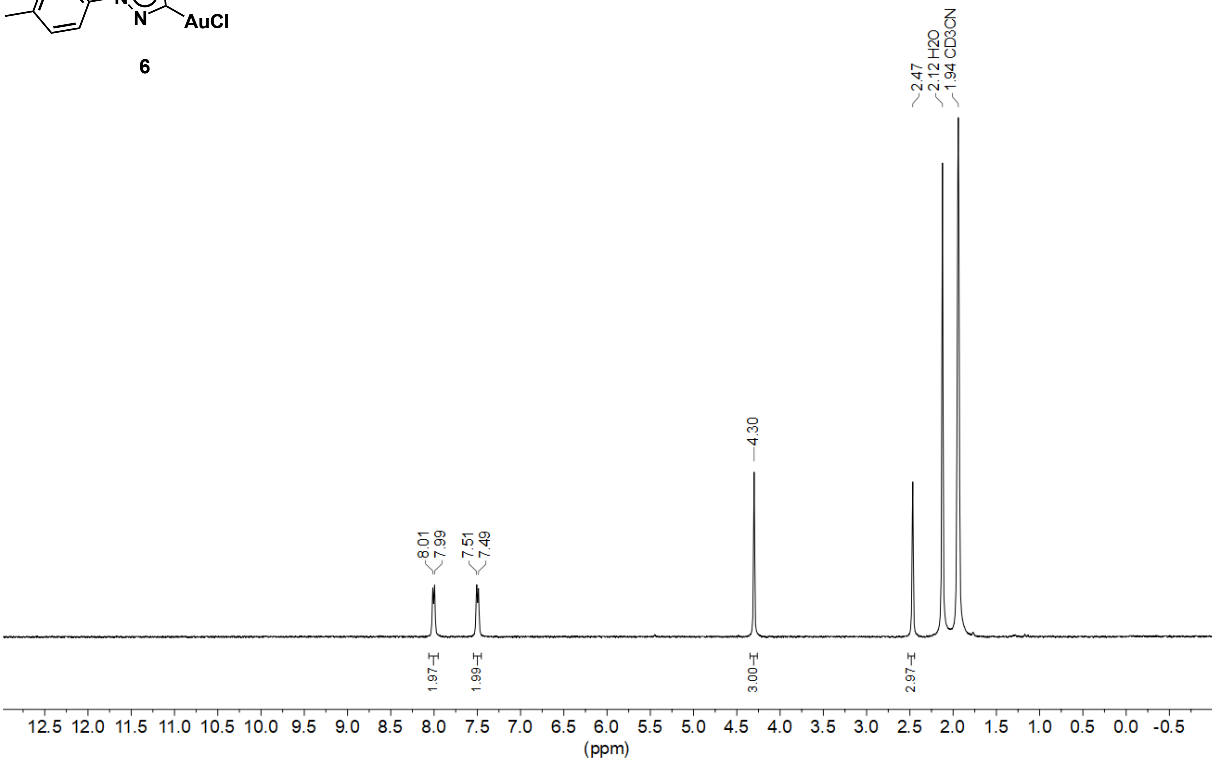

Figure S59: ^{1}H-NMR spectrum of **6** in MeCN-d_3.

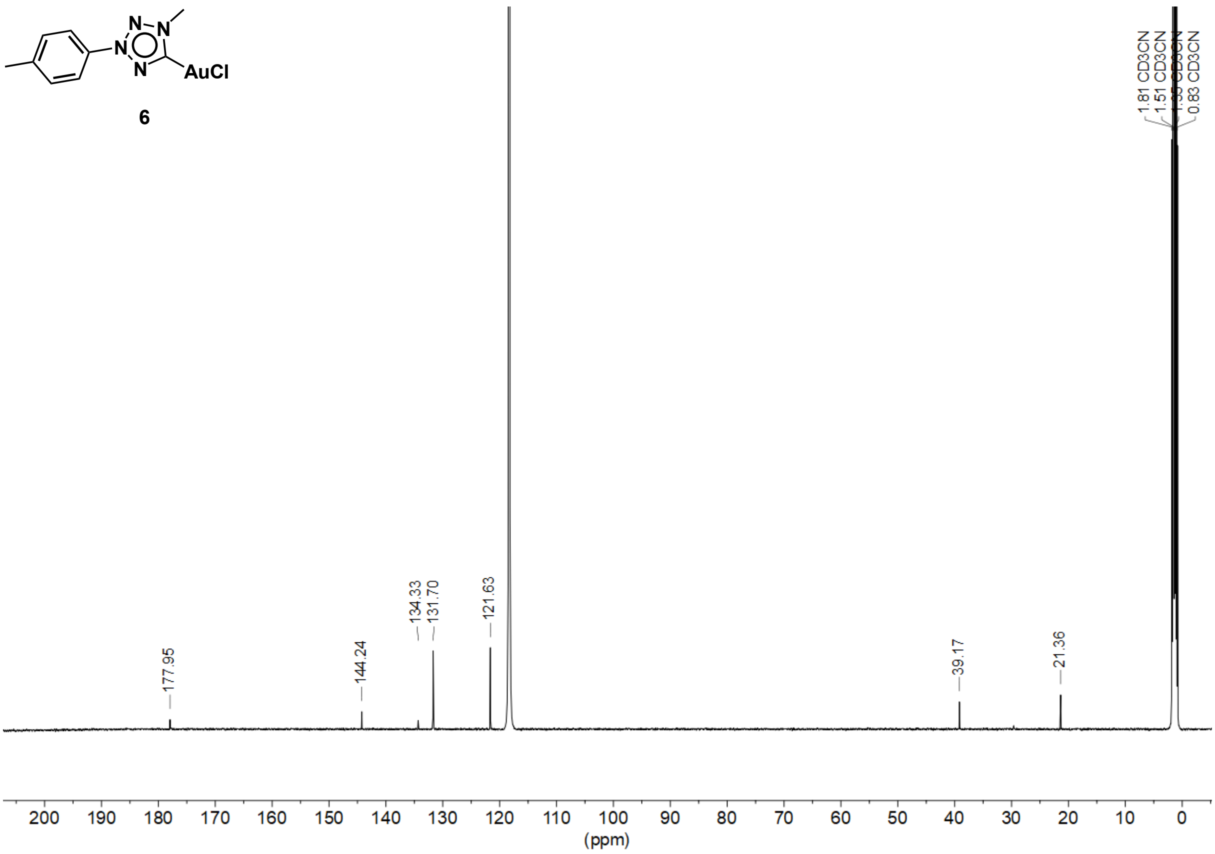

Figure S60: ^{13}C-NMR spectrum of **6** in MeCN-d_3.

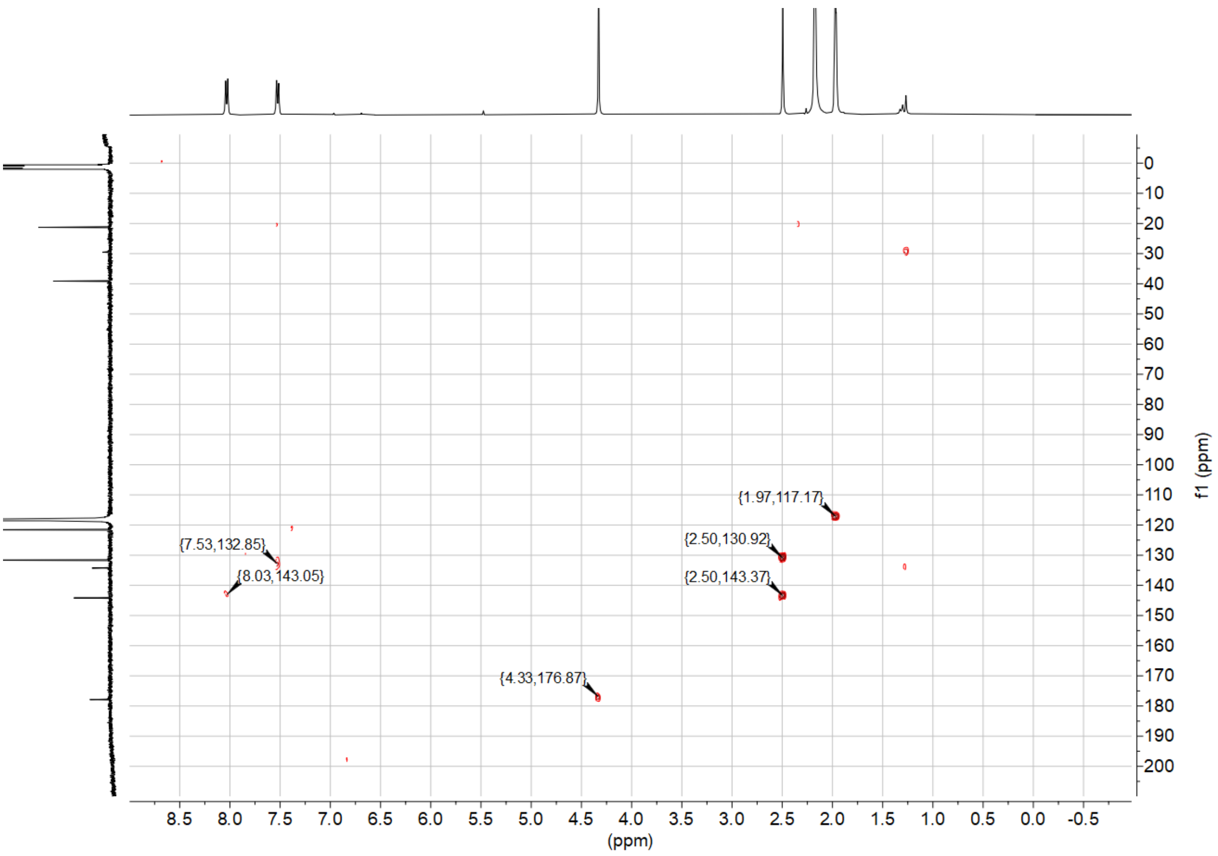

Figure S61: HMBC spectrum of **6** in MeCN-d_3.

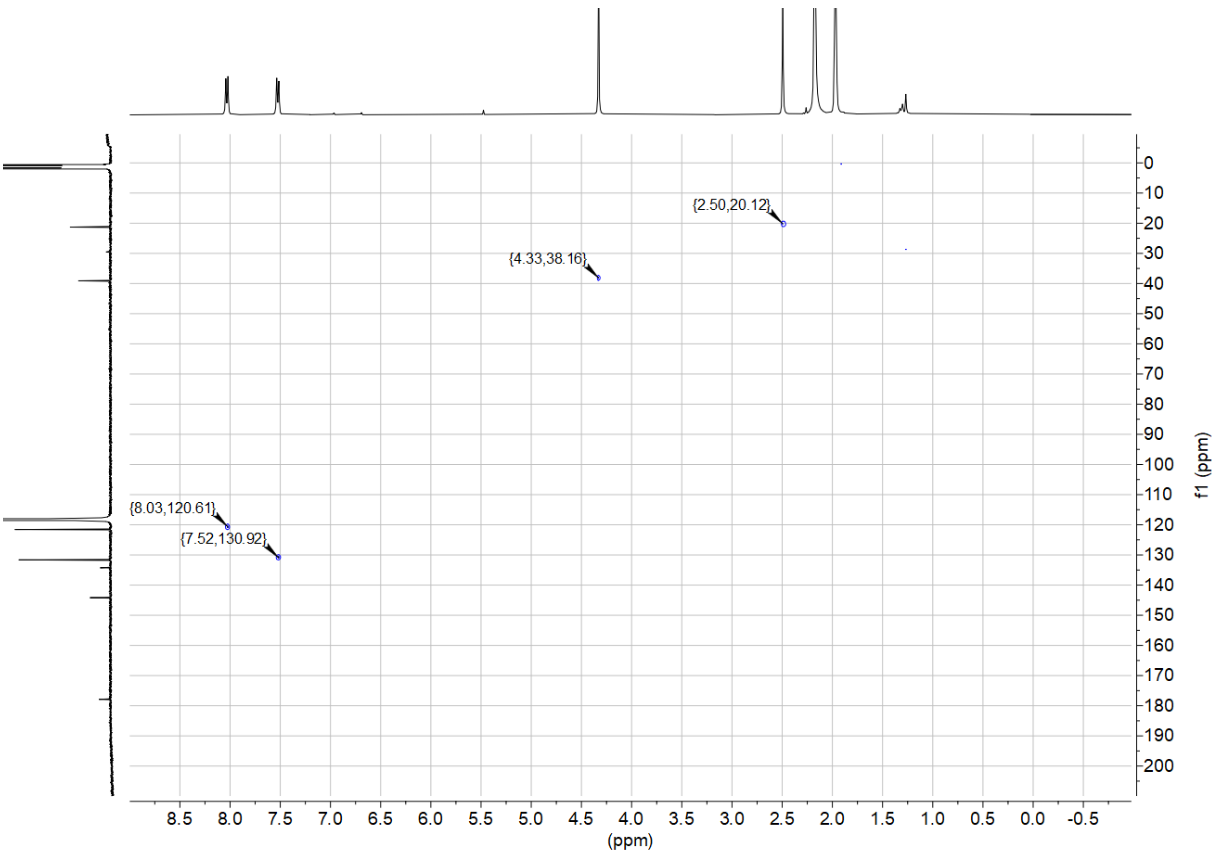

Figure S62: HSQC spectrum of **6** in MeCN-d_3.

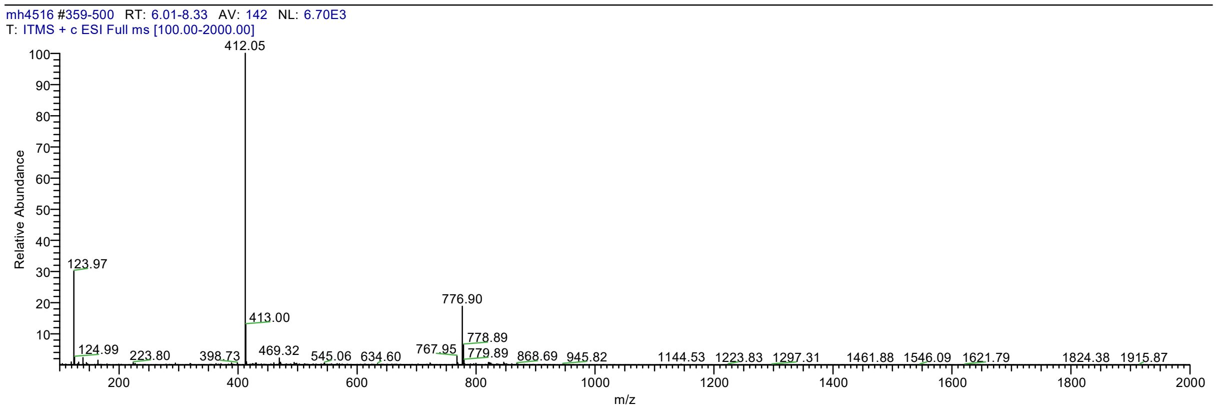

Figure S63: ESI mass spectrum of **6** in MeCN as solvent.

3.36 1-*t*-Butyl-3-isopropyl-tetrazolylidene Gold(I) Chloride 7

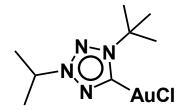

7

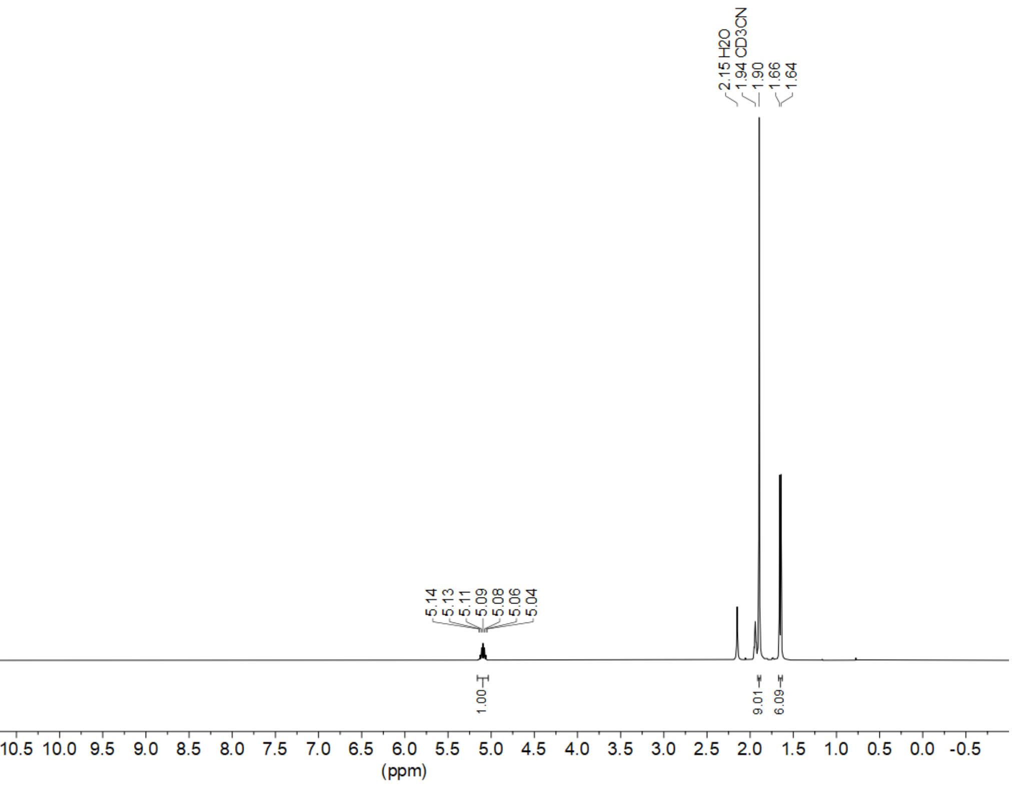

Figure S64: ^{1}H-NMR spectrum of **7** in MeCN-d_3.

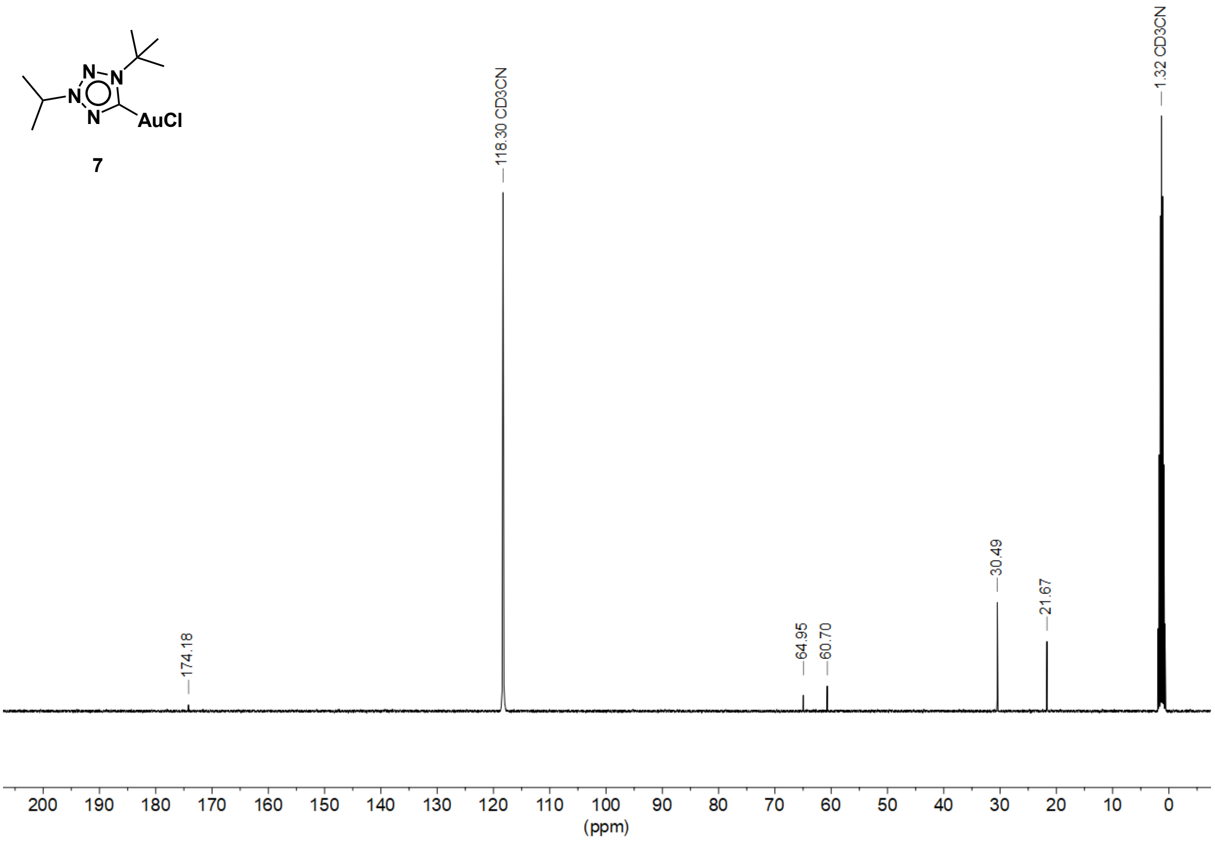

Figure S65: ^{13}C-NMR spectrum of **7** in MeCN-d_3.

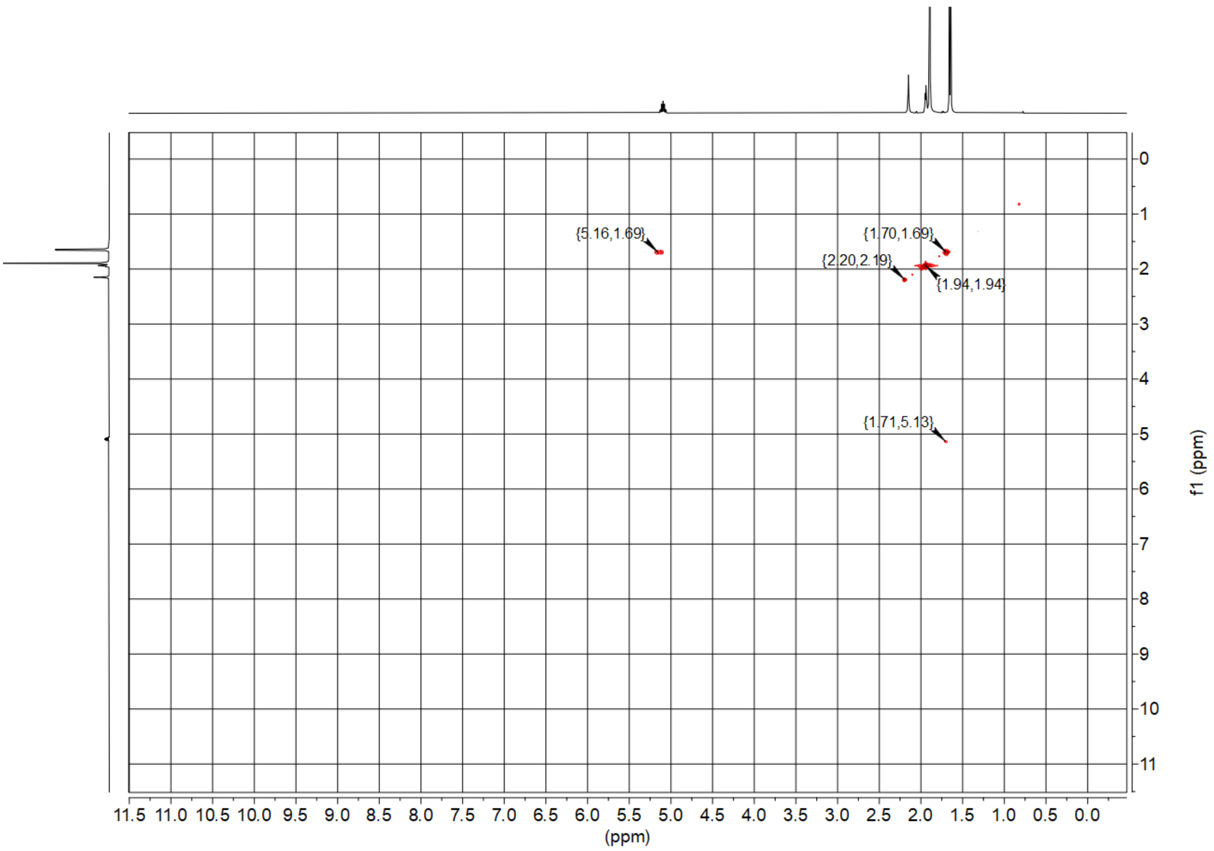

Figure S66: COSY spectrum of **7** in MeCN-d_3.

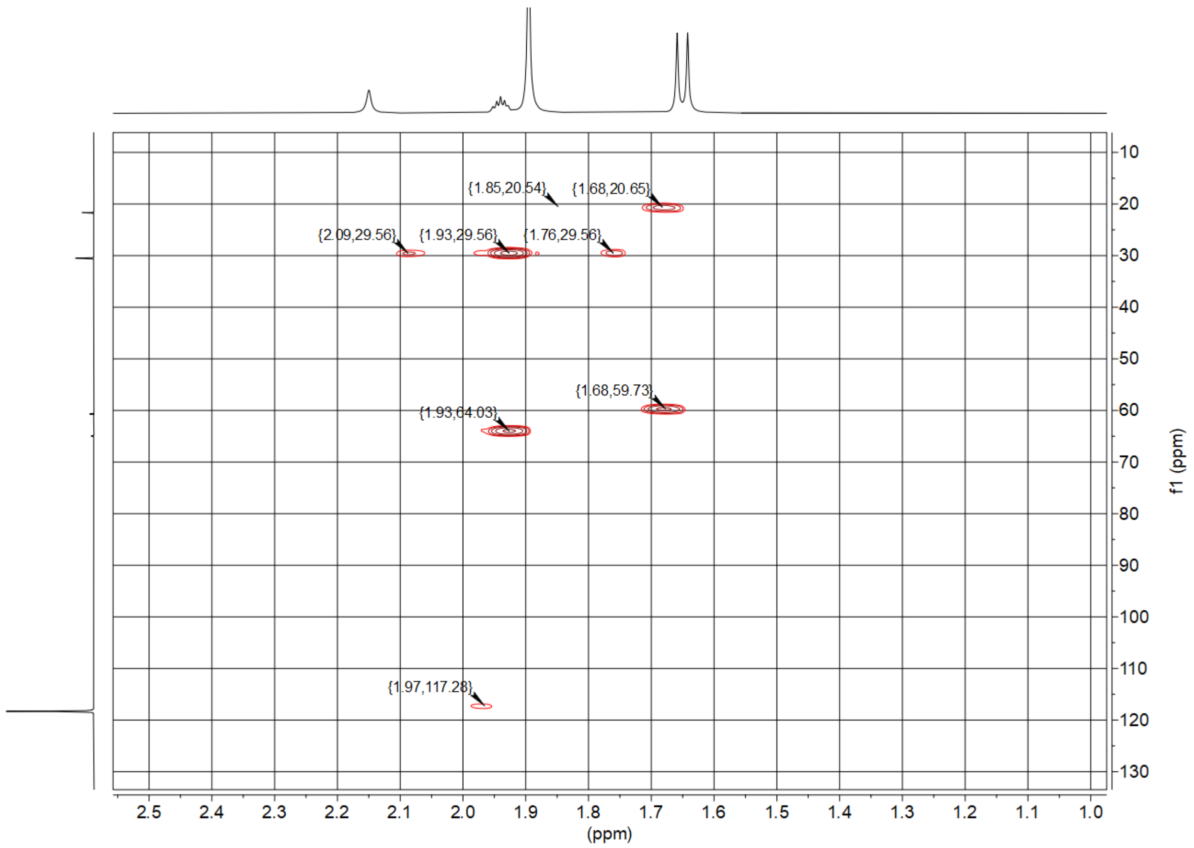

Figure S67: HMBC spectrum of **7** in MeCN-d_3.

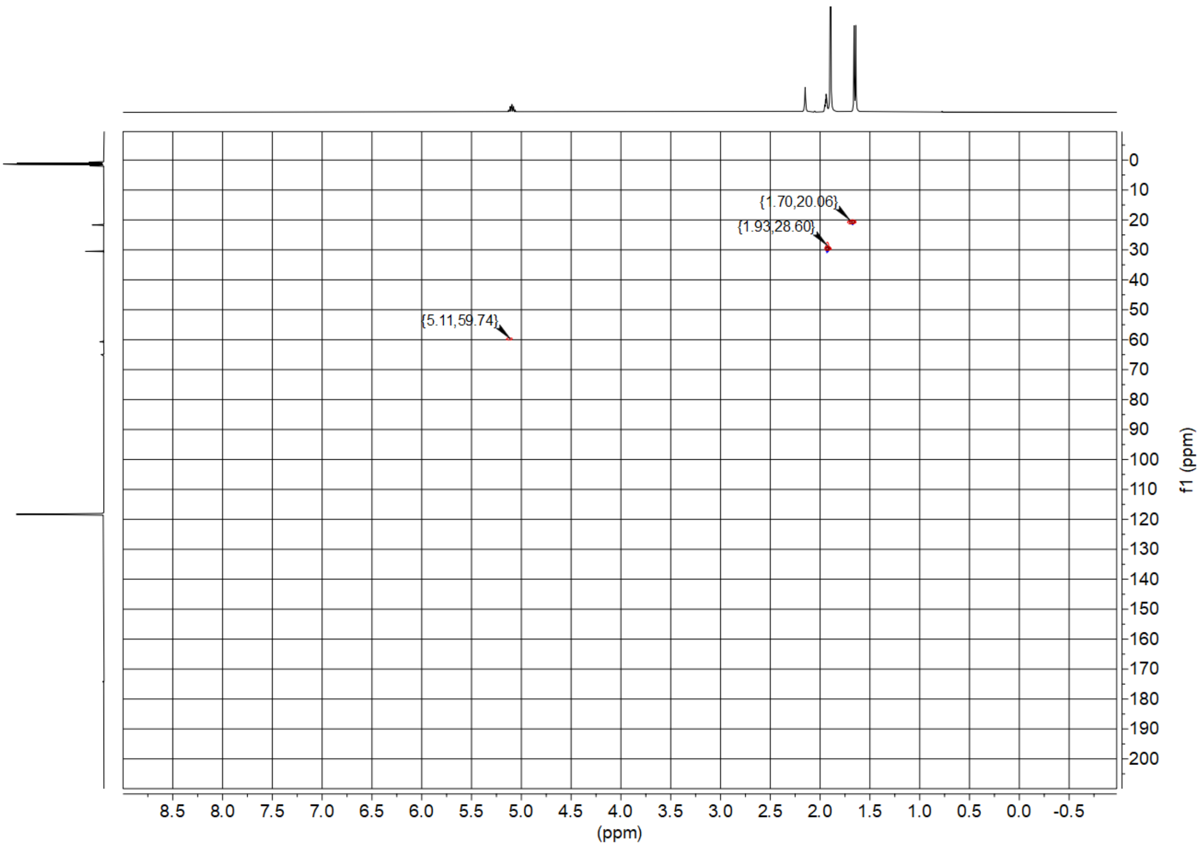

Figure S68: HSQC spectrum of **7** in MeCN-d_3.

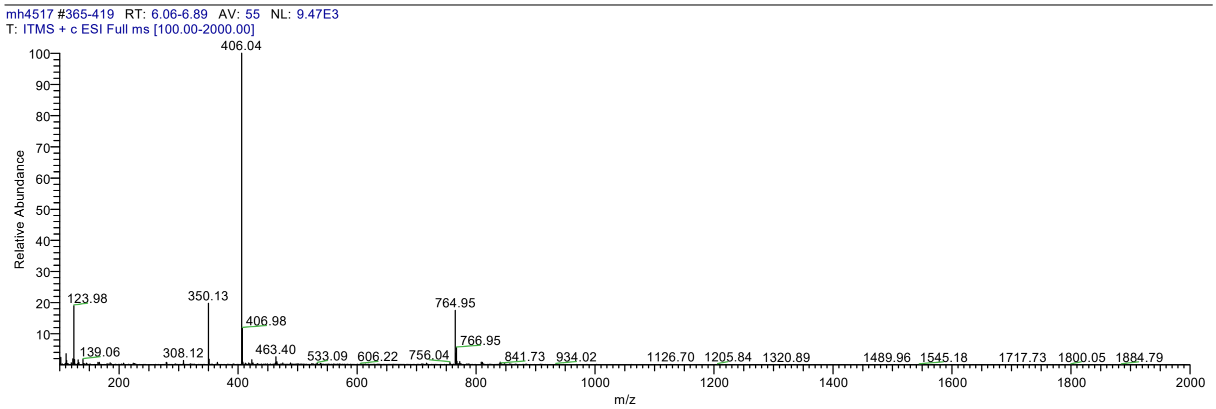

Figure S69: ESI mass spectrum of **7** in MeCN as solvent.

3.37 Bis-(1,3-diphenyl-tetrazoylylidene) Gold(I) Hexafluorophosphate B1

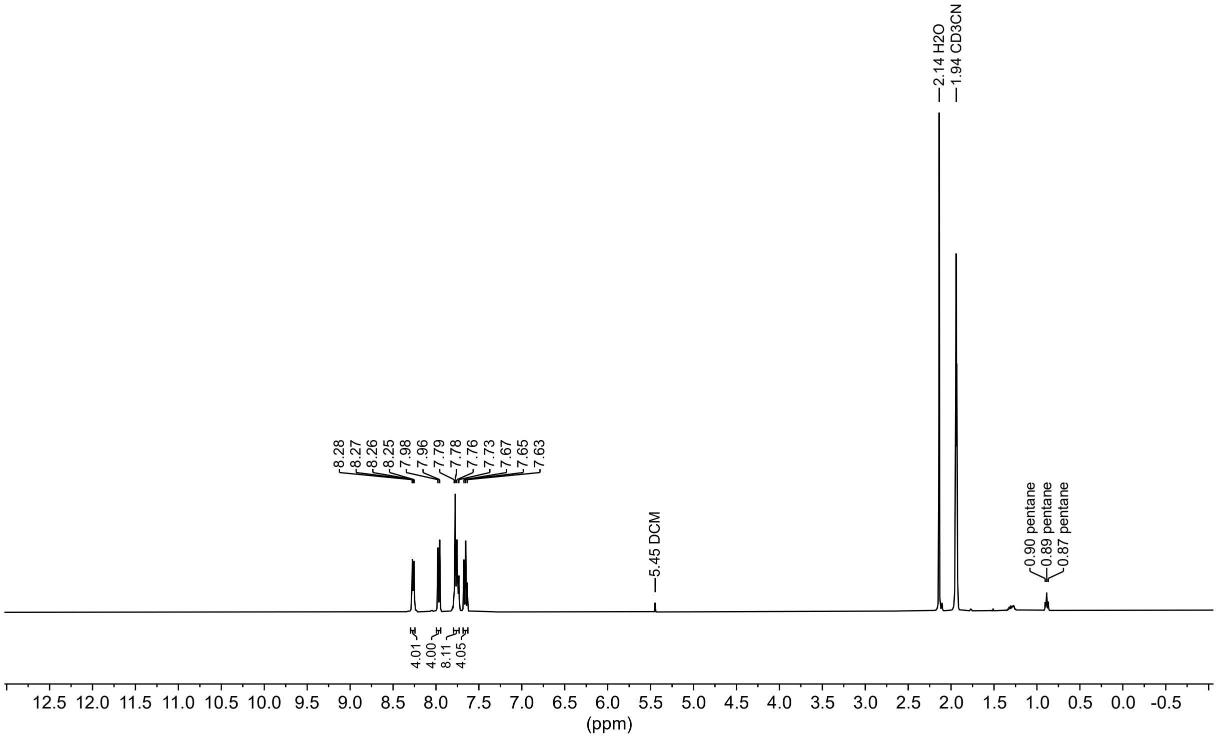

Figure S70: ^{1}H-NMR spectrum of **B1** in MeCN-d_3.

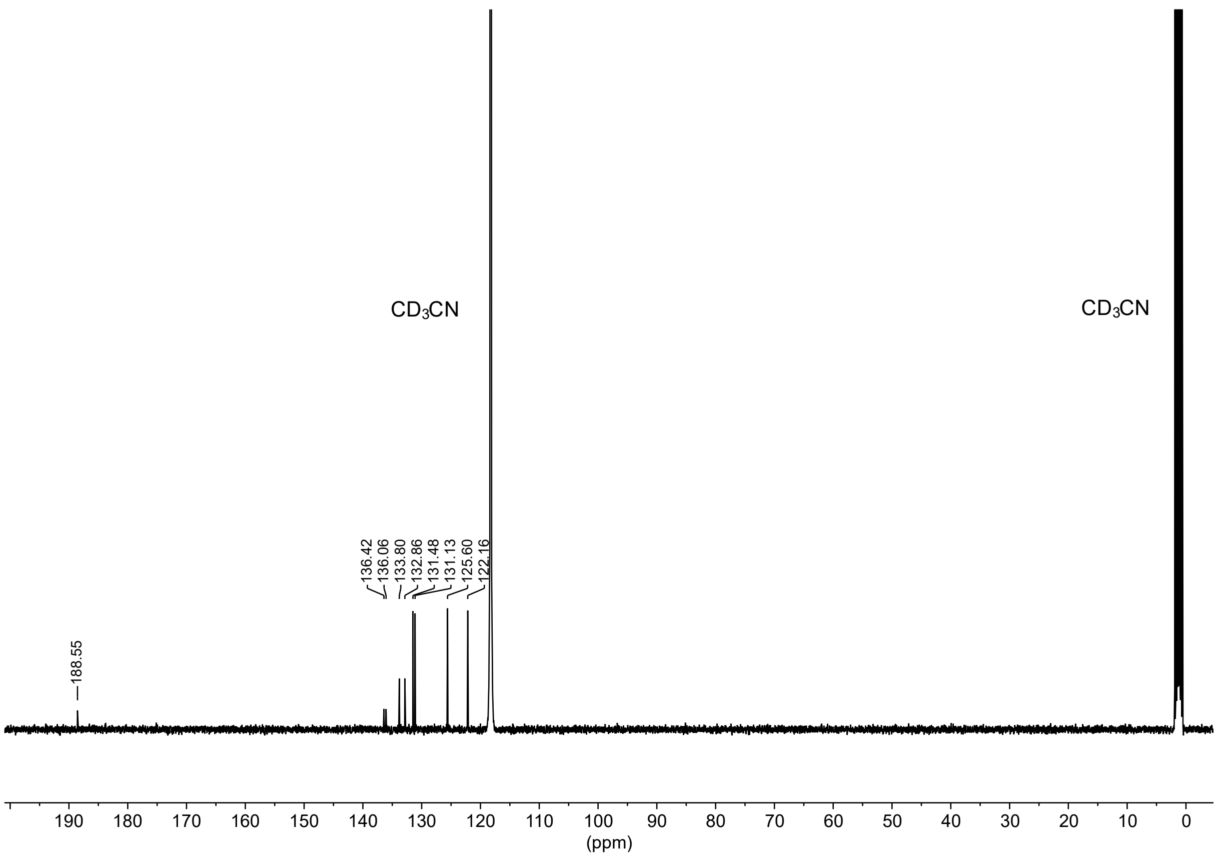

Figure S71: ^{13}C-NMR spectrum of **B1** in MeCN-d_3.

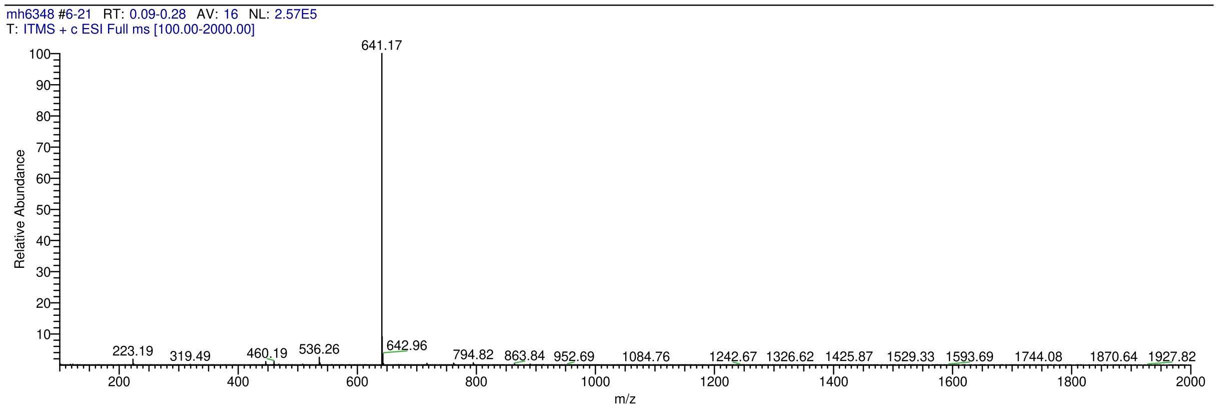

Figure S72: ESI mass spectrum of **B1** in MeCN as solvent.

3.38 Bis-(1-(*p*-tolyl)-3-phenyl-tetrazolylidene) Gold(I) Hexafluorophosphate B2

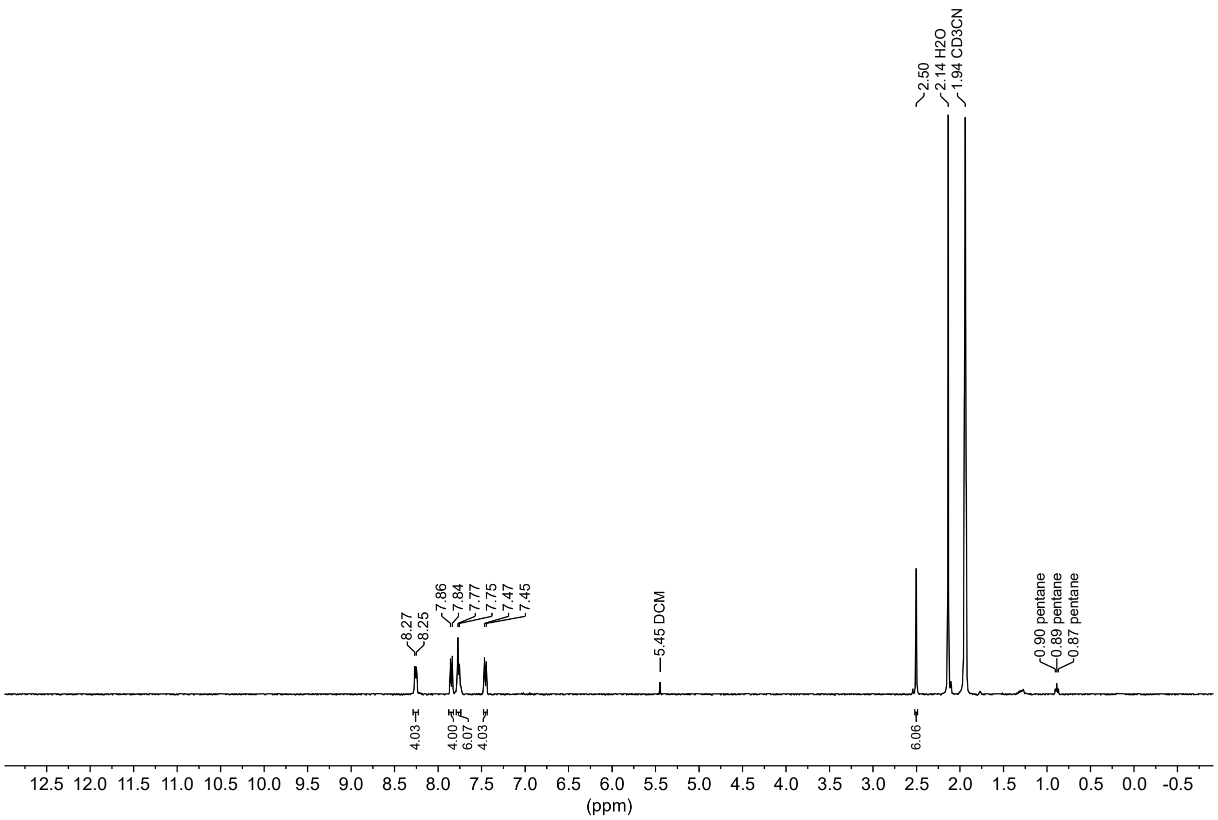

Figure S73: ^{1}H-NMR spectrum of **B2** in MeCN-d_3.

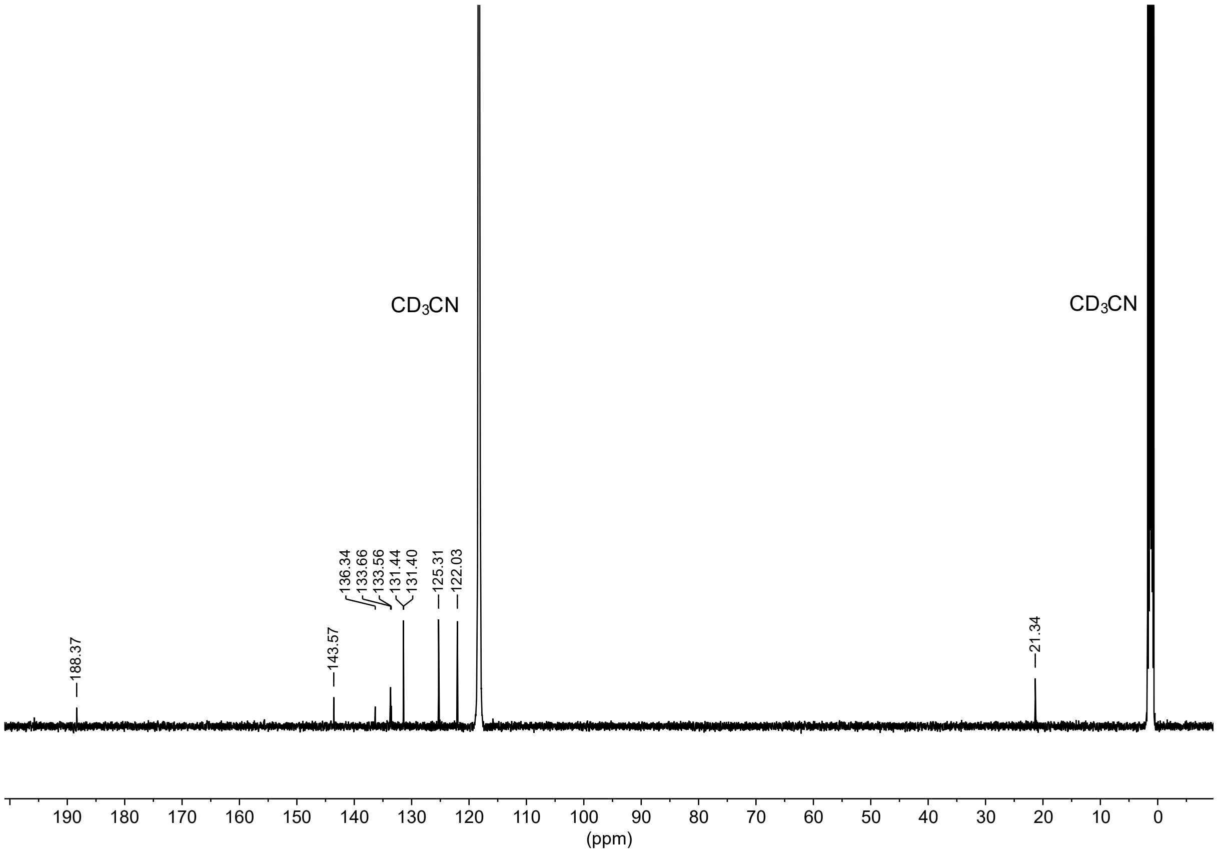

Figure S74: ^{13}C-NMR spectrum of **B2** in MeCN-d_3.

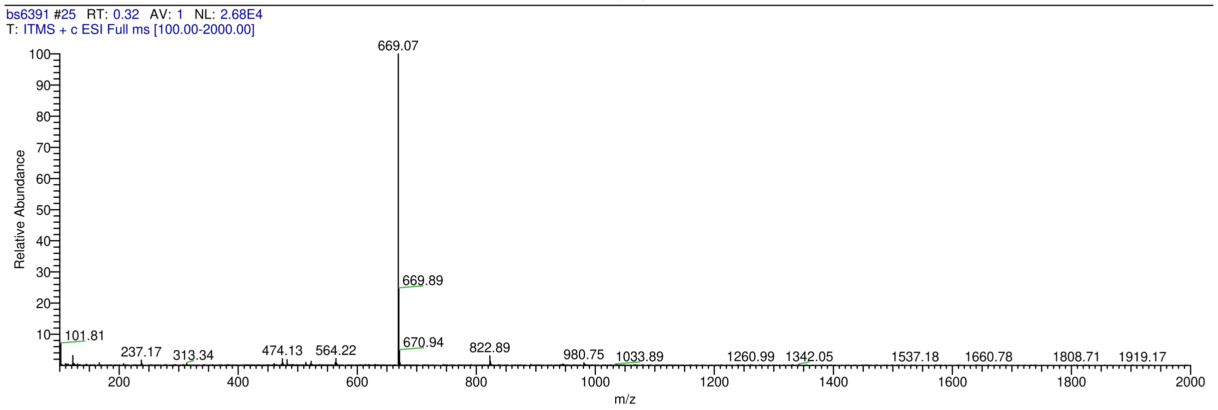

Figure S75: ESI mass spectrum of **B2** in MeCN as solvent.

4 Stability Studies

The stability of the 1,3-disubstituted tetrazolylidene gold(I) complexes (**1**–**7**) was investigated in the presence of biologically relevant thiols, using *L*-cysteine and glutathione (GSH) as model nucleophiles. Reactivity studies were carried out using a solvent system consisting of degassed DMSO-d_6 and D_2O (4:1), suitable to dissolve both the complex and the respective thiol species. To investigate the nature of newly formed species, the reaction mixtures were incubated at 37°C and ^{1}H NMR spectra were recorded over time. Prior to the addition of the thiol containing compound, the complexes were tested for solvent stability. It should be noted that excess cysteine and GSH signals decrease due to autoxidation and formation of cystine and glutathione disulfide (GSSG).

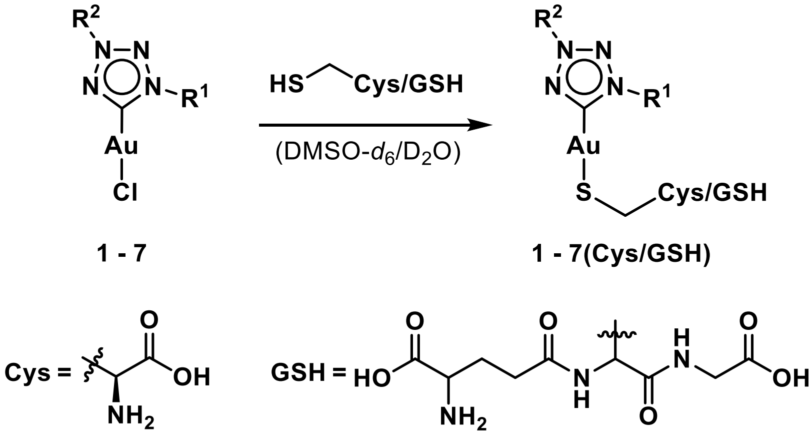

Scheme S14: Reaction of tetrazolylidene gold(I) chloride complexes with thiol containing compounds (*L*-cysteine, GSH).

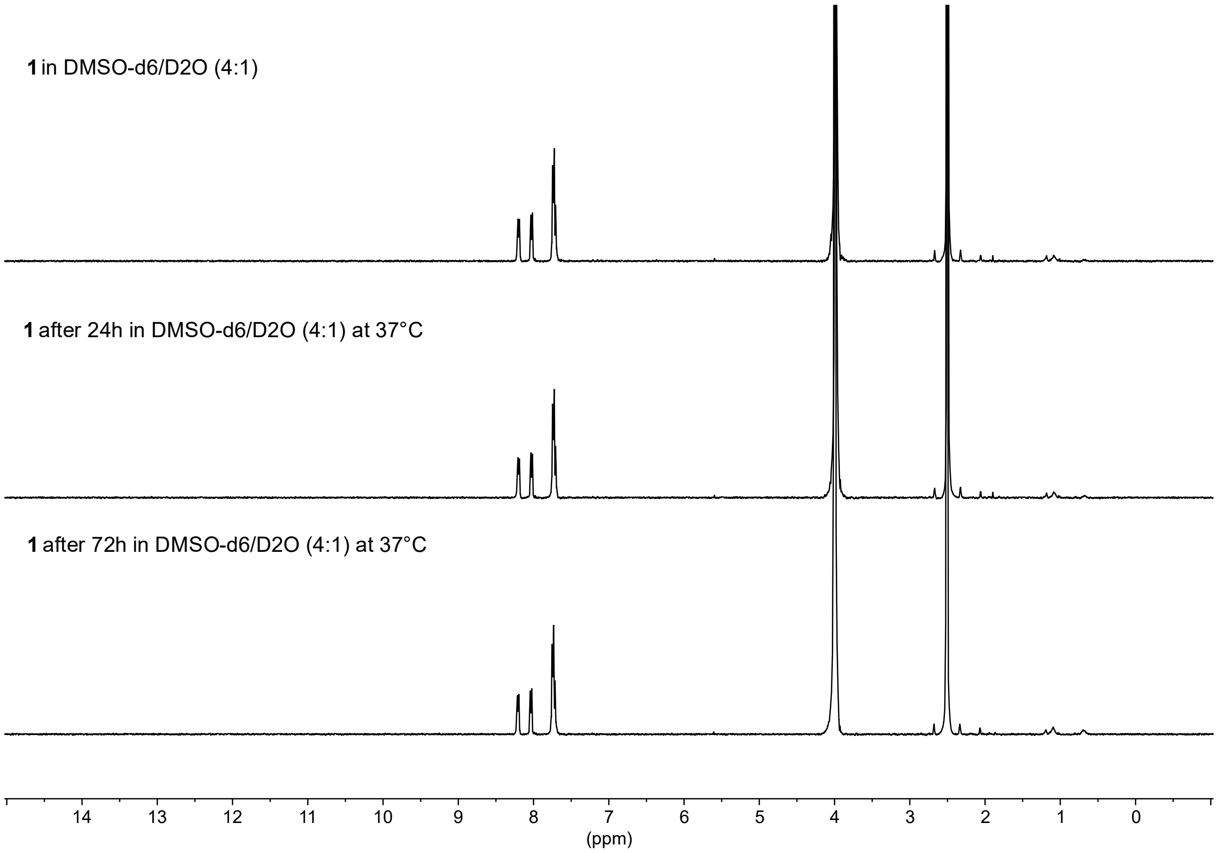

Figure S76: Stability of **1** in DMSO-d_6/D_2O (4:1) at 37°C at different time points (0 h, 24 h, 72 h).

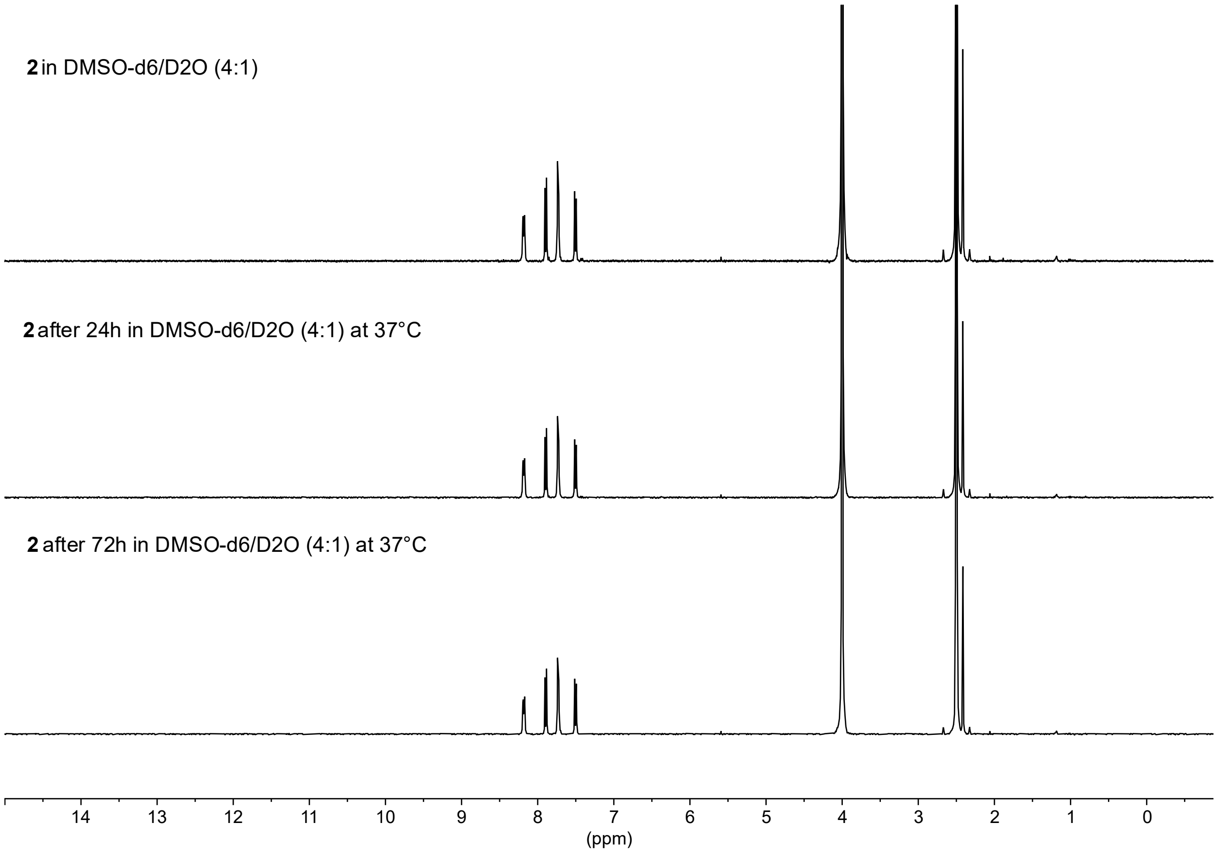

Figure S77: Stability of **2** in DMSO-d_6/D_2O (4:1) at 37°C at different time points (0 h, 24 h, 72 h).

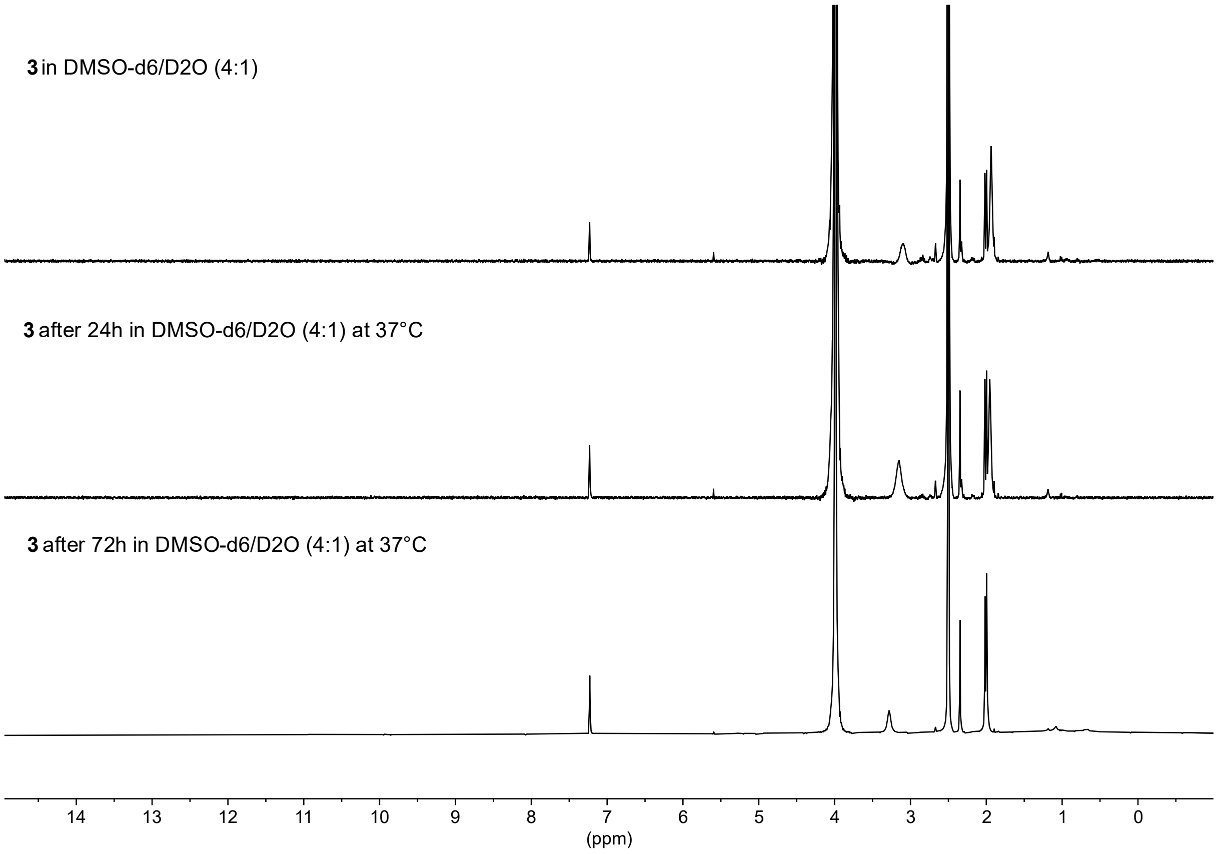

Figure S78: Stability of **3** in DMSO-d_6/D_2O (4:1) at 37°C at different time points (0 h, 24 h, 72 h).

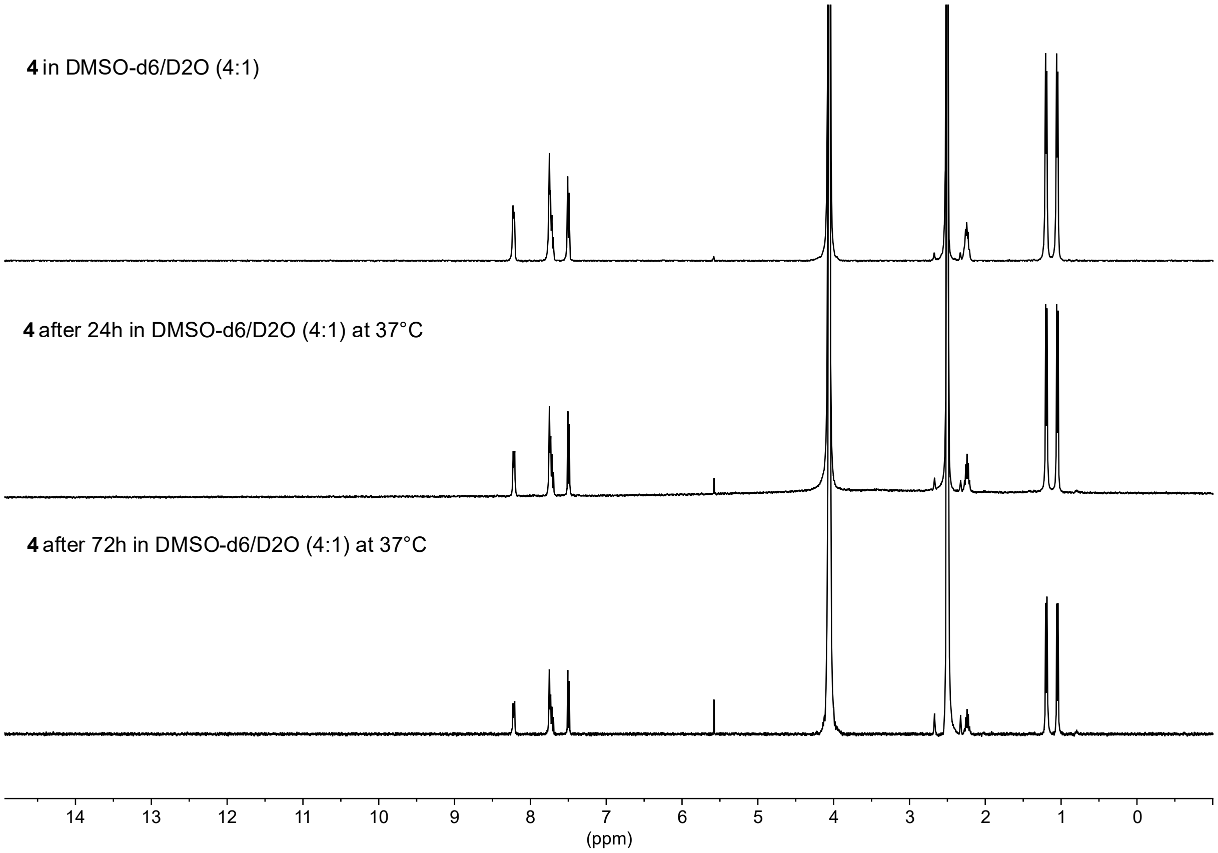

Figure S79: Stability of **4** in DMSO-d_6/D_2O (4:1) at 37°C at different time points (0 h, 24 h, 72 h).

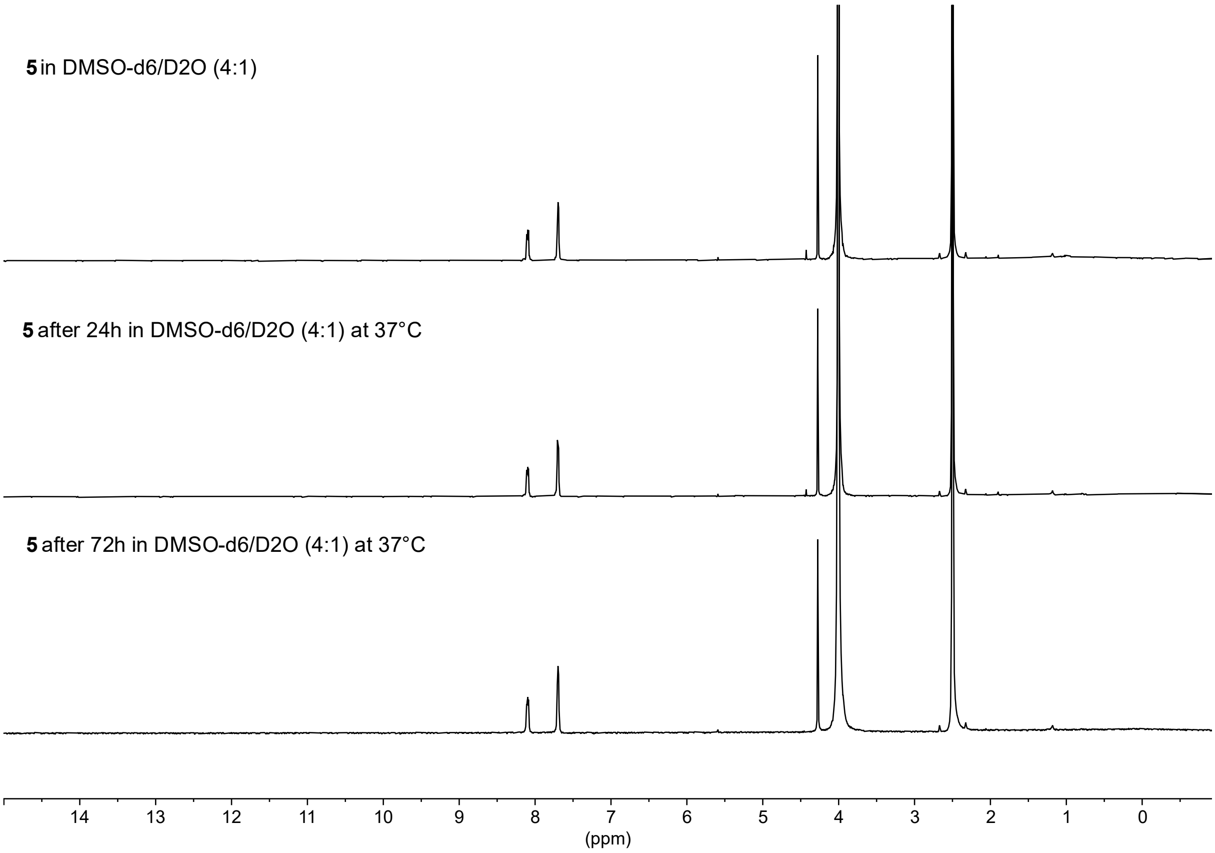

Figure S80: Stability of **5** in DMSO-d_6/D_2O (4:1) at 37°C at different time points (0 h, 24 h, 72 h).

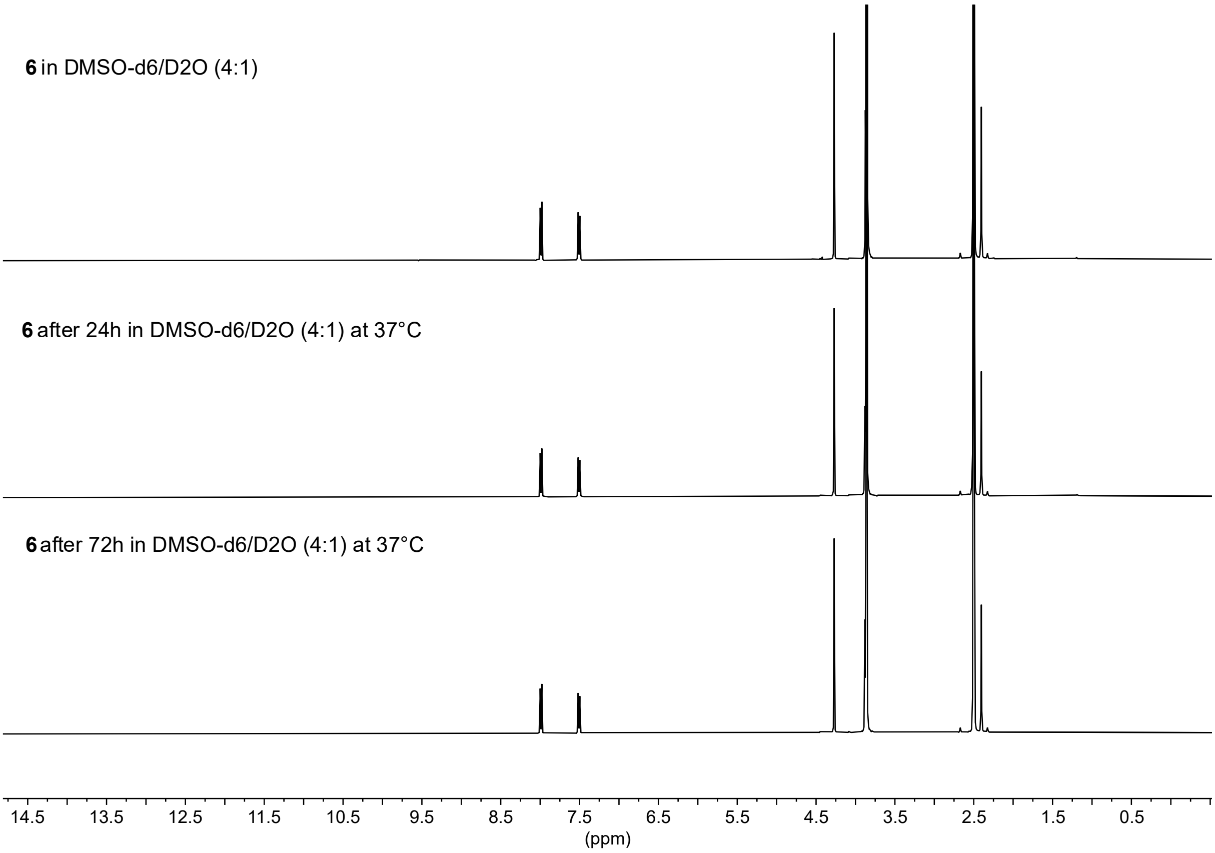

Figure S81: Stability of **6** in DMSO-d_6/D_2O (4:1) at 37°C at different time points (0 h, 24 h, 72 h).

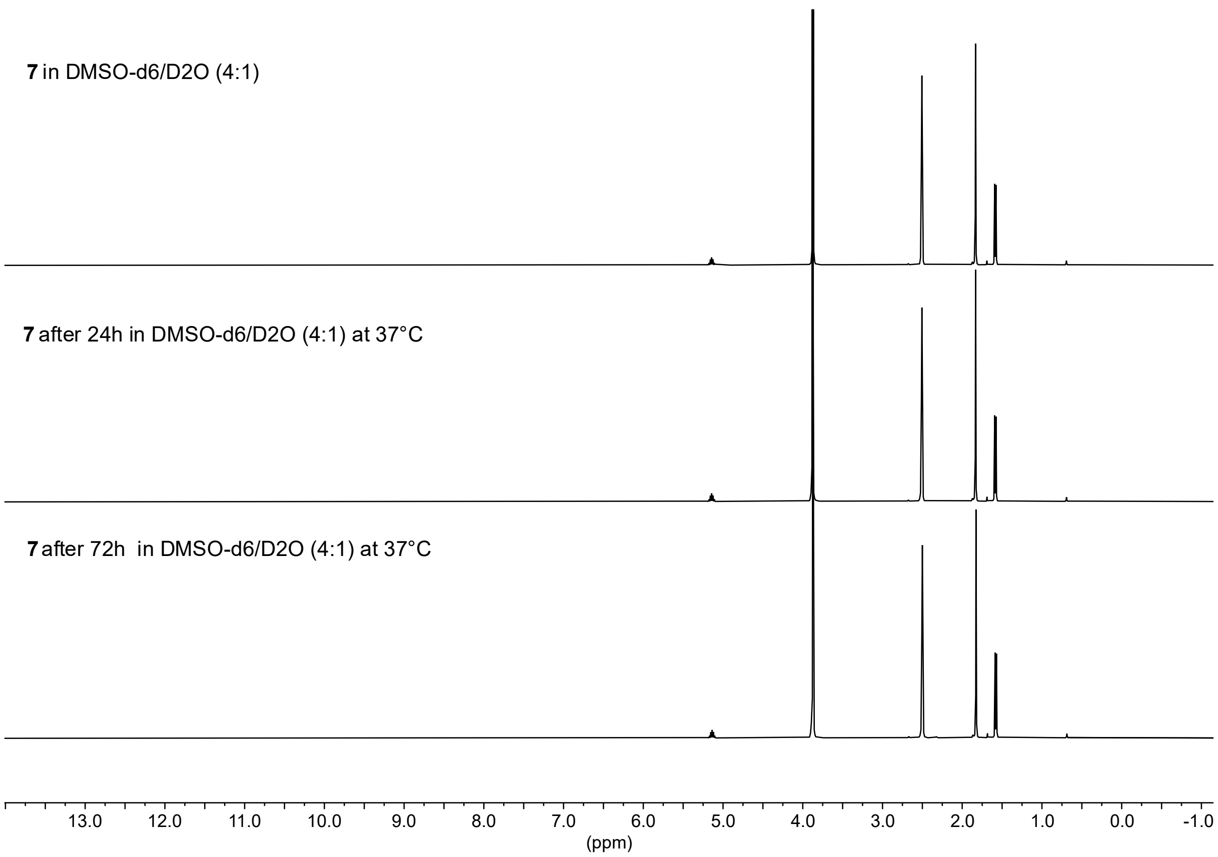

Figure S82: Stability of **7** in DMSO-d_6/D_2O (4:1) at 37°C at different time points (0 h, 24 h, 72 h).

4.1 Analysis of the Stability of Compounds 1-7 against *L*-Cysteine and GSH

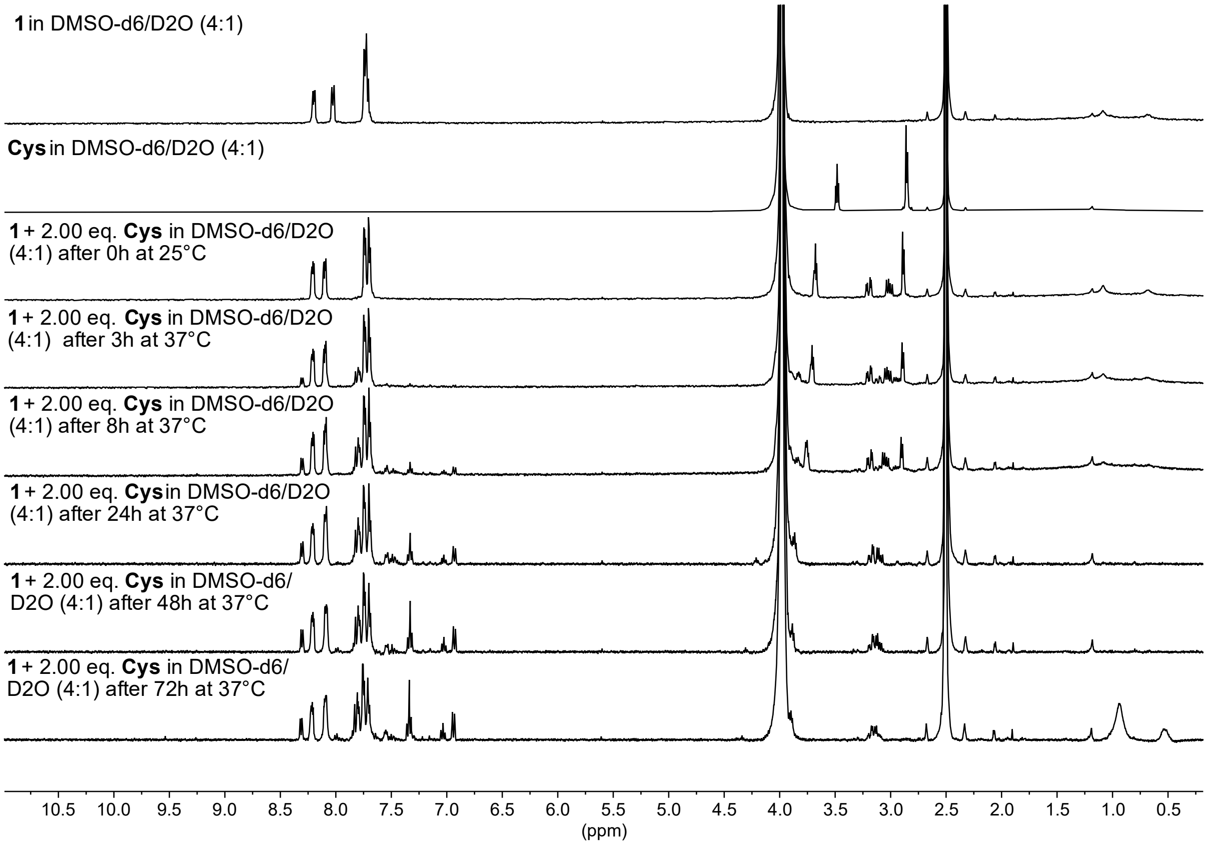

Figure S83: Stability of **1** in DMSO-d_6/D_2O (4:1) after the addition of 2.00 eq. Cys at 37°C at different time points. Spectra of **1** and Cys in DMSO-d_6/D_2O (4:1) are included for reference.

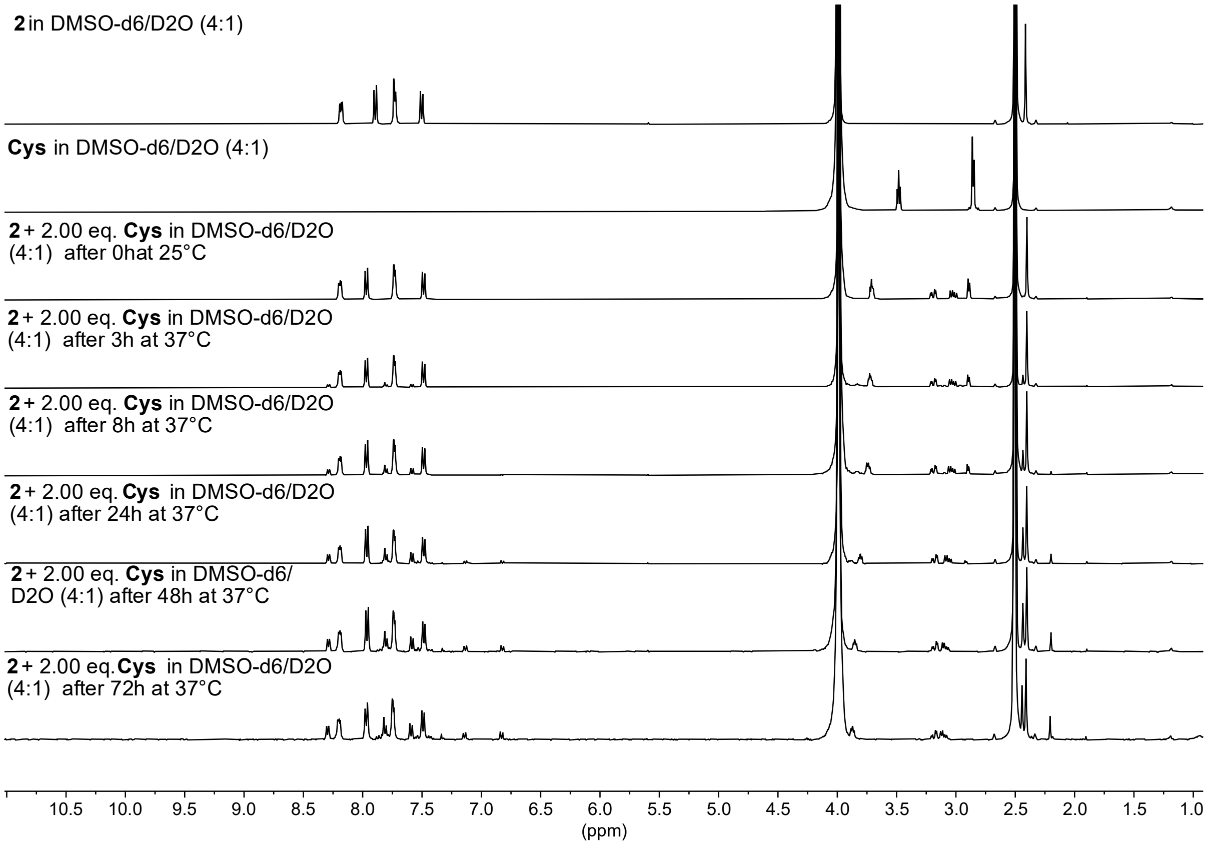

Figure S84: Stability of **2** in DMSO-d_6/D_2O (4:1) after the addition of 2.00 eq. Cys at 37°C at different time points. Spectra of **2** and Cys in DMSO-d_6/D_2O (4:1) are included for reference.

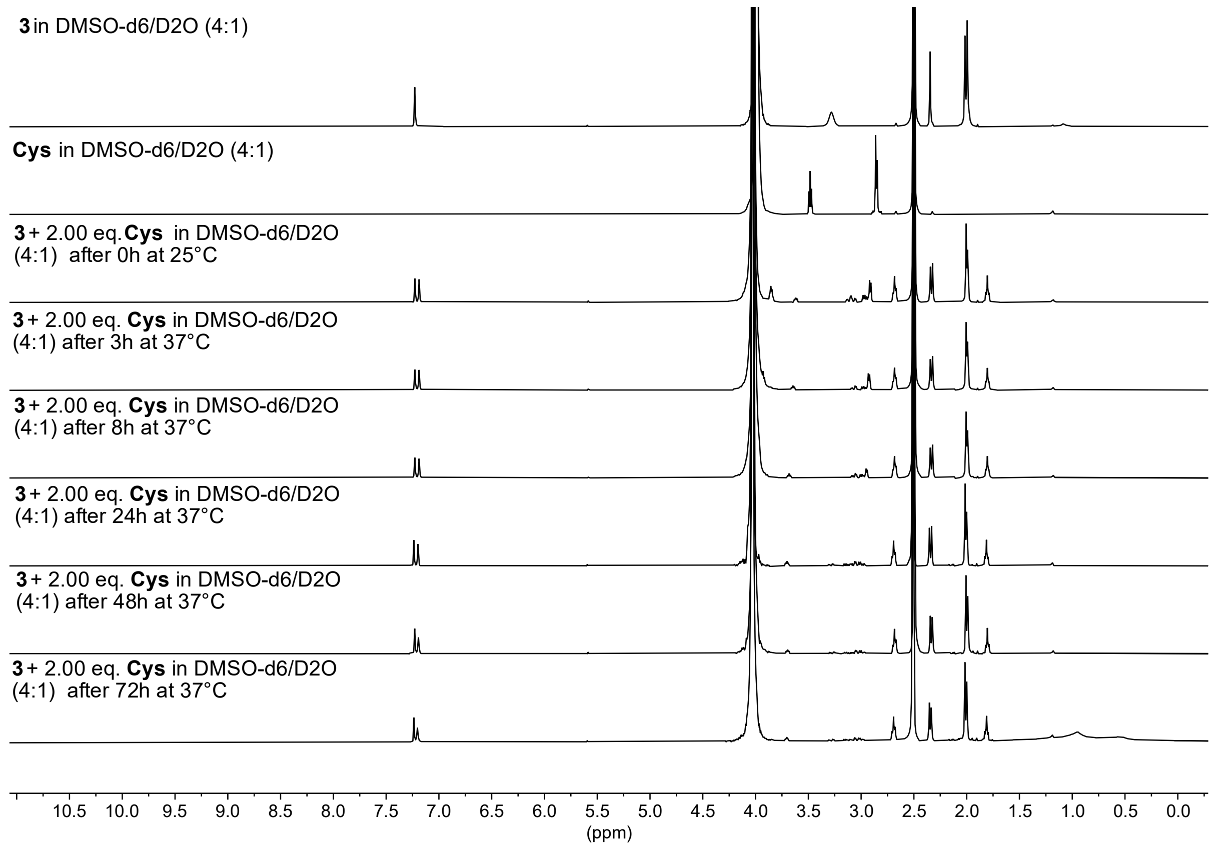

Figure S85: Stability of **3** in DMSO-d_6/D_2O (4:1) after the addition of 2.00 eq. Cys at 37°C at different time points. Spectra of **3** and Cys in DMSO-d_6/D_2O (4:1) are included for reference.

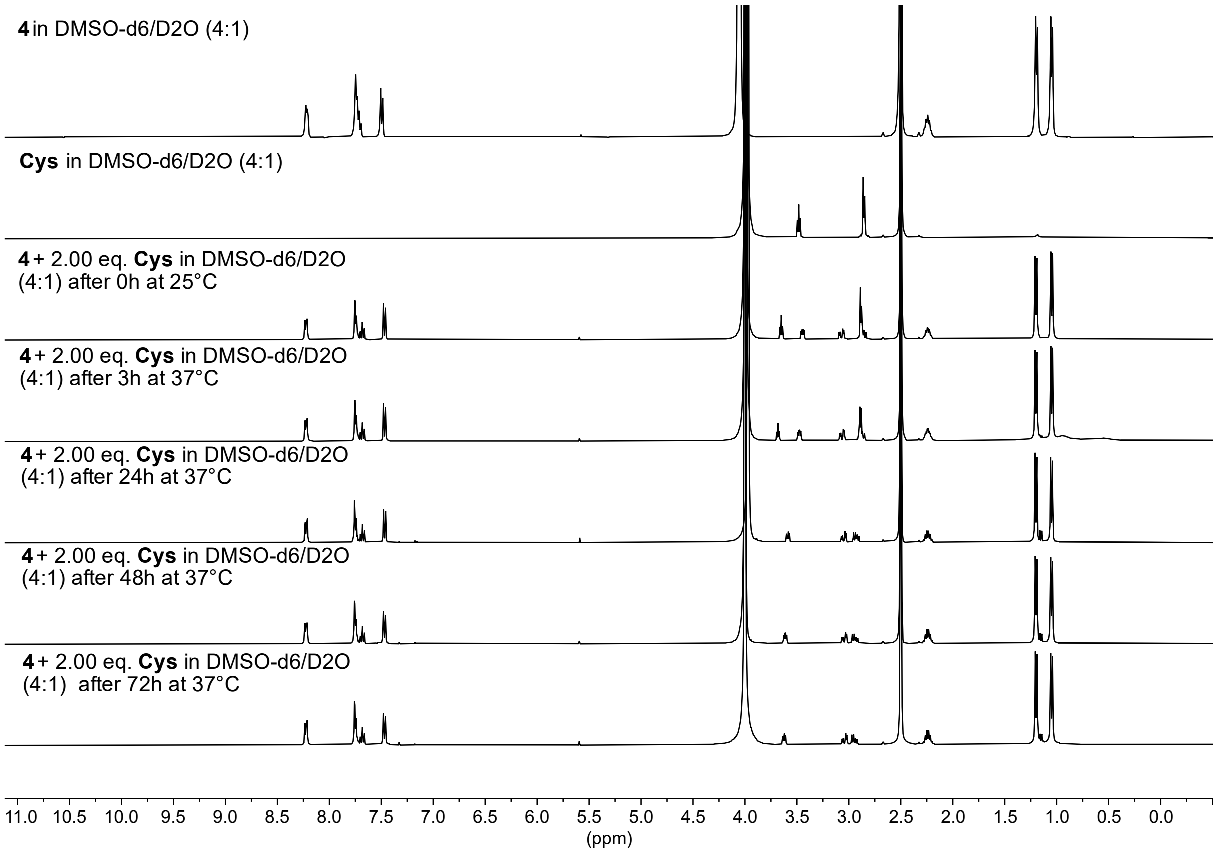

Figure S86: Stability of **4** in DMSO-d_6/D_2O (4:1) after the addition of 2.00 eq. Cys at 37°C at different time points. Spectra of **4** and Cys in DMSO-d_6/D_2O (4:1) are included for reference.

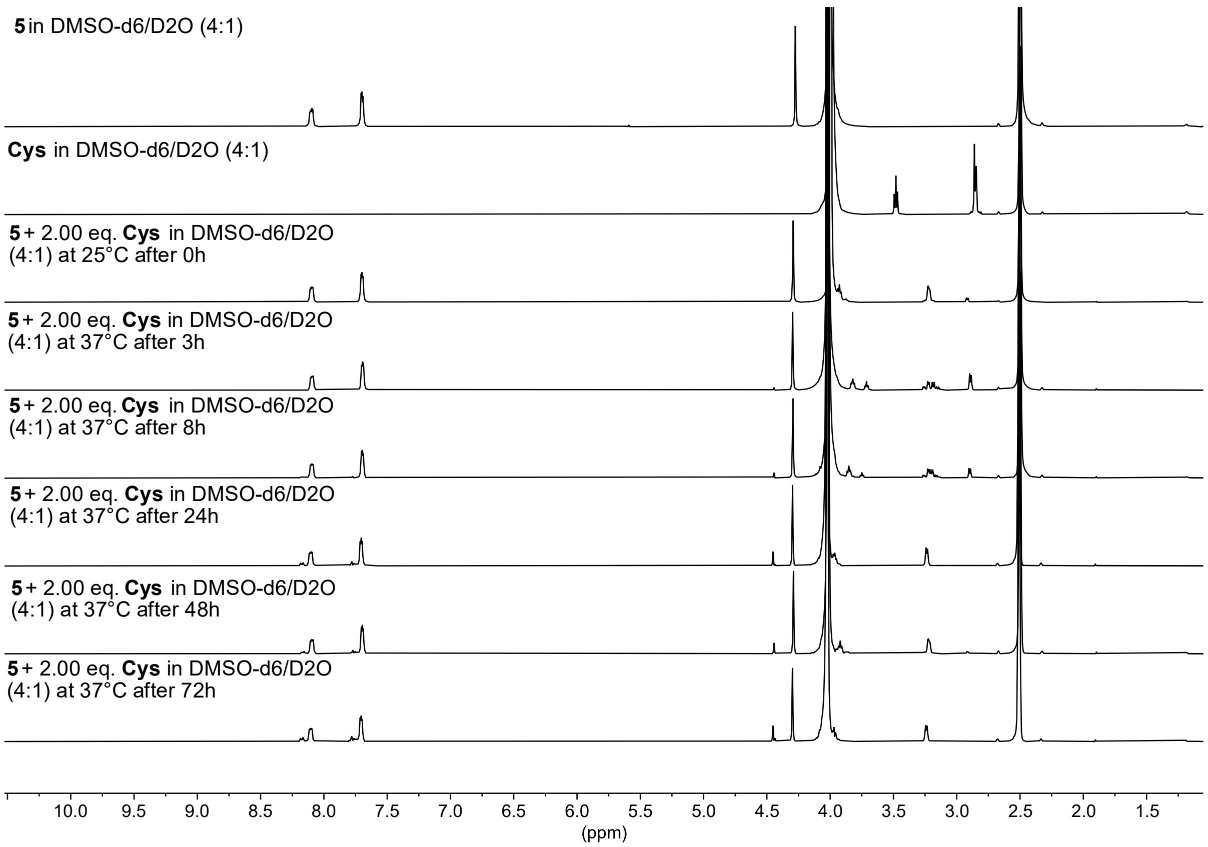

Figure S87: Stability of **5** in DMSO-d_6/D_2O (4:1) after the addition of 2.00 eq. Cys at 37°C at different time points. Spectra of **5** and Cys in DMSO-d_6/D_2O (4:1) are included for reference.

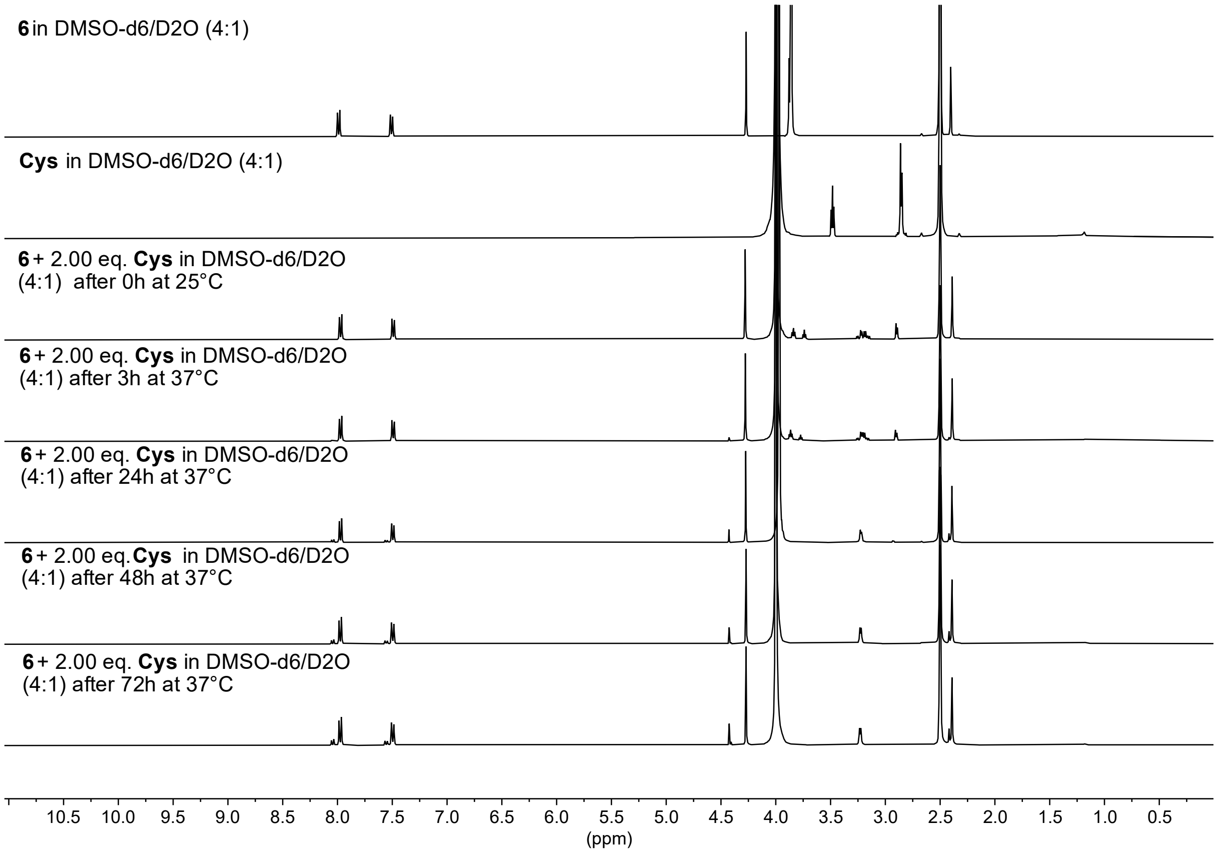

Figure S88: Stability of **6** in DMSO-d_6/D_2O (4:1) after the addition of 2.00 eq. Cys at 37°C at different time points. Spectra of **6** and Cys in DMSO-d_6/D_2O (4:1) are included for reference.

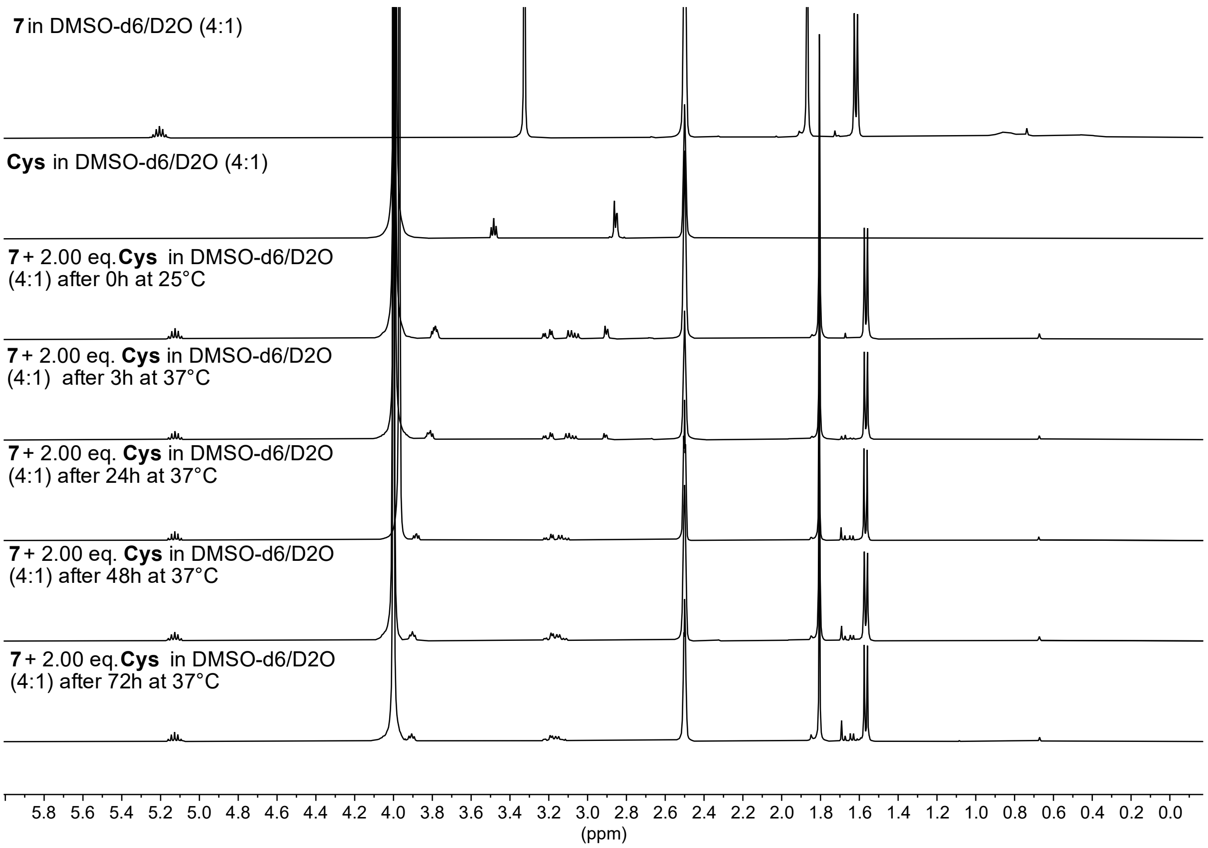

Figure S89: Stability of **7** in DMSO-d_6/D_2O (4:1) after the addition of 2.00 eq. Cys at 37°C at different time points. Spectra of **7** and Cys in DMSO-d_6/D_2O (4:1) are included for reference.

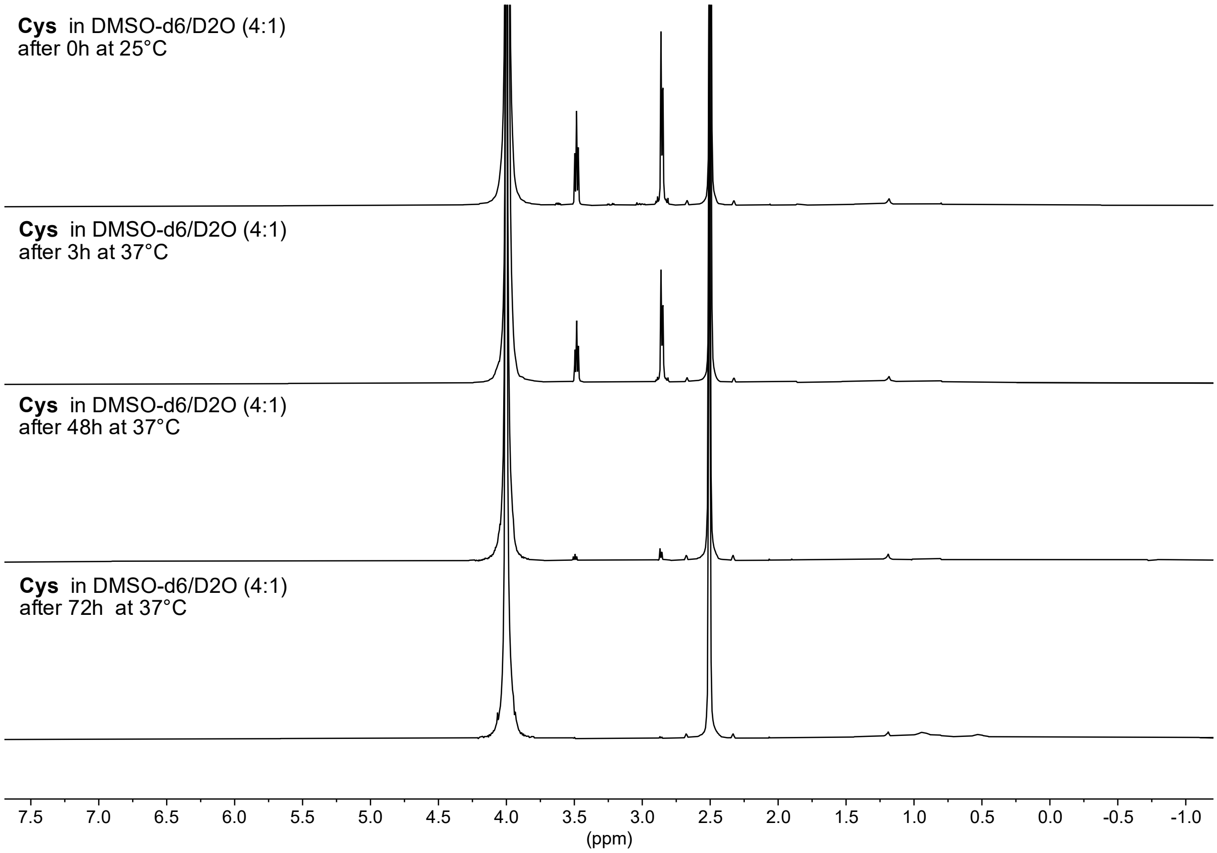

Figure S90: Stability of Cys in DMSO-d_6/D_2O (4:1) at 37°C at different time points.

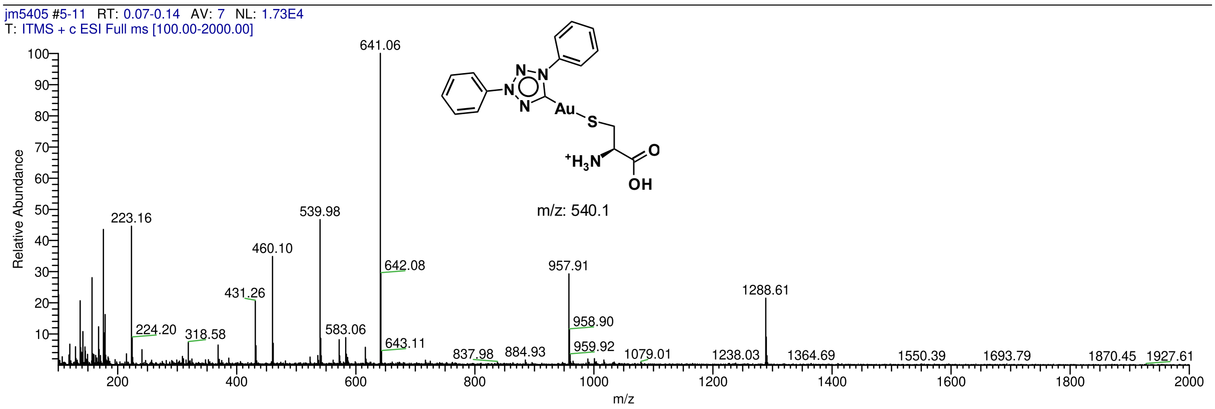

Figure S91: ESI mass spectrum of **1(Cys)** in MeCN/DMSO (90:10) as solvent.

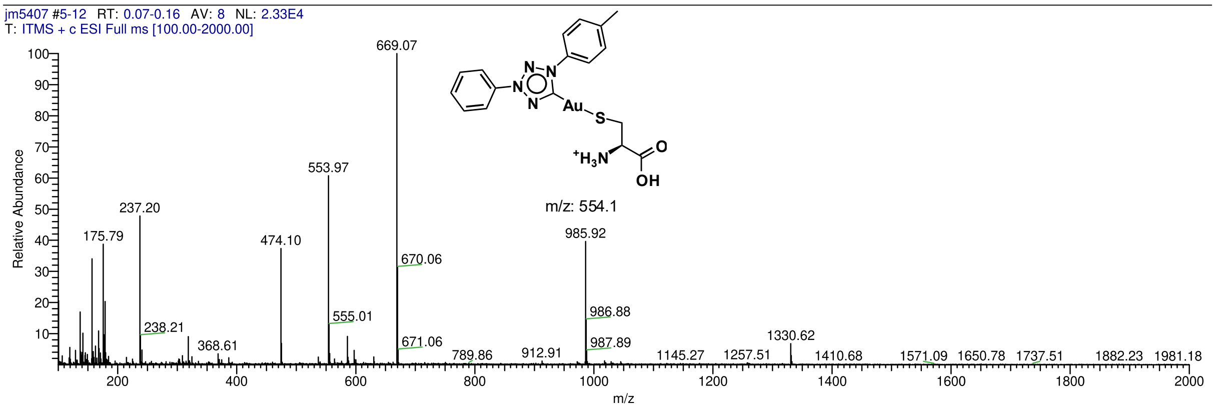

Figure S92: ESI mass spectrum of **2(Cys)** in MeCN/DMSO (90:10) as solvent.

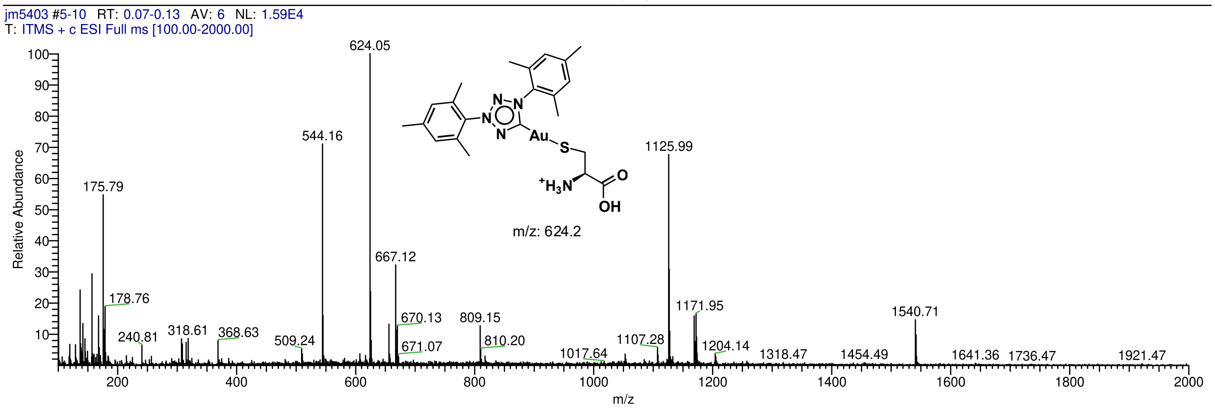

Figure S93: ESI mass spectrum of **3(Cys)** in MeCN/DMSO (90:10) as solvent.

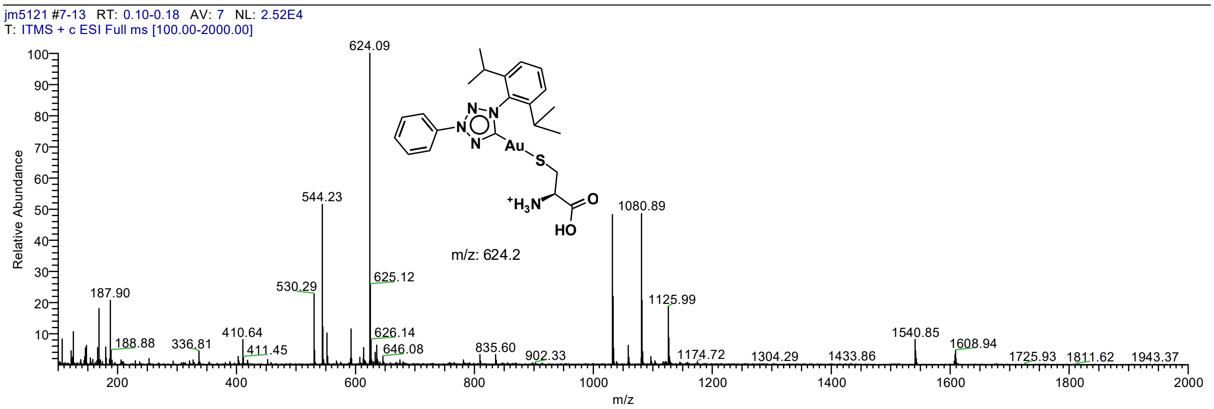

Figure S94: ESI mass spectrum of **4(Cys)** with MeCN/DMSO (90:10) as solvent.

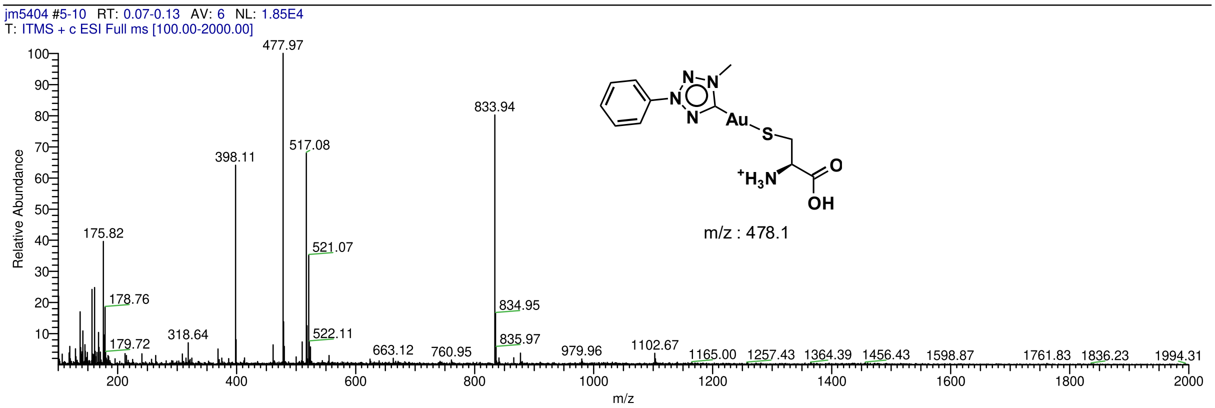

Figure S95: ESI mass spectrum of **5(Cys)** in MeCN/DMSO (90:10) as solvent.

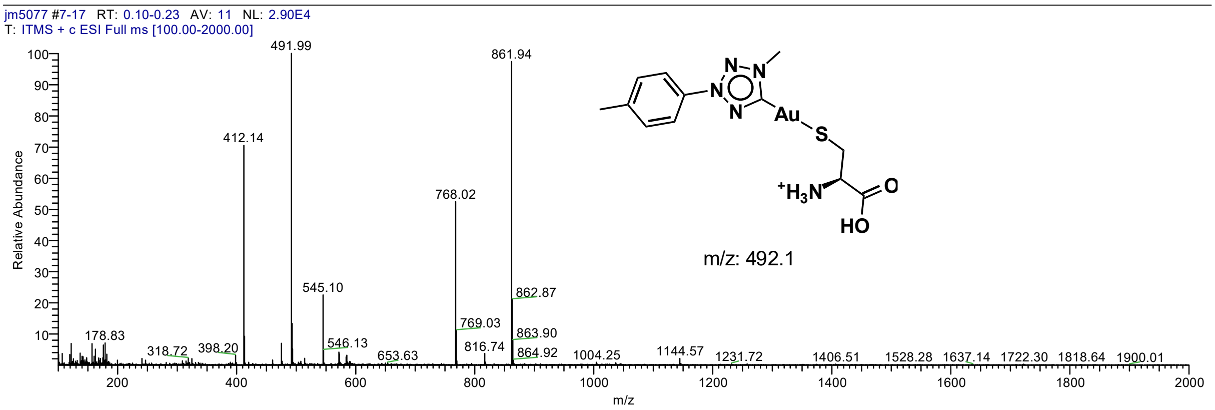

Figure S96: ESI mass spectrum of **6(Cys)** with MeCN/DMSO (90:10) as solvent.

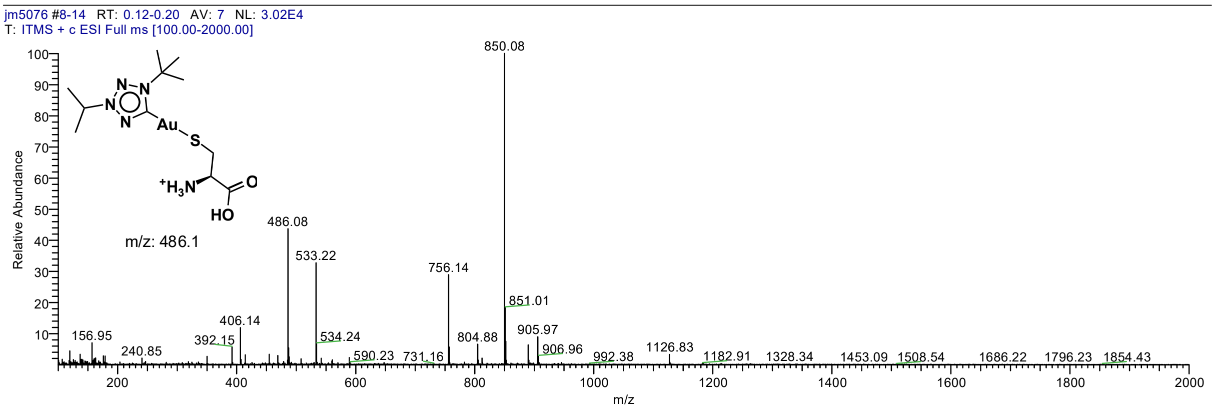

Figure S97: ESI mass spectrum of **7(Cys)** with MeCN/DMSO (90:10) as solvent.

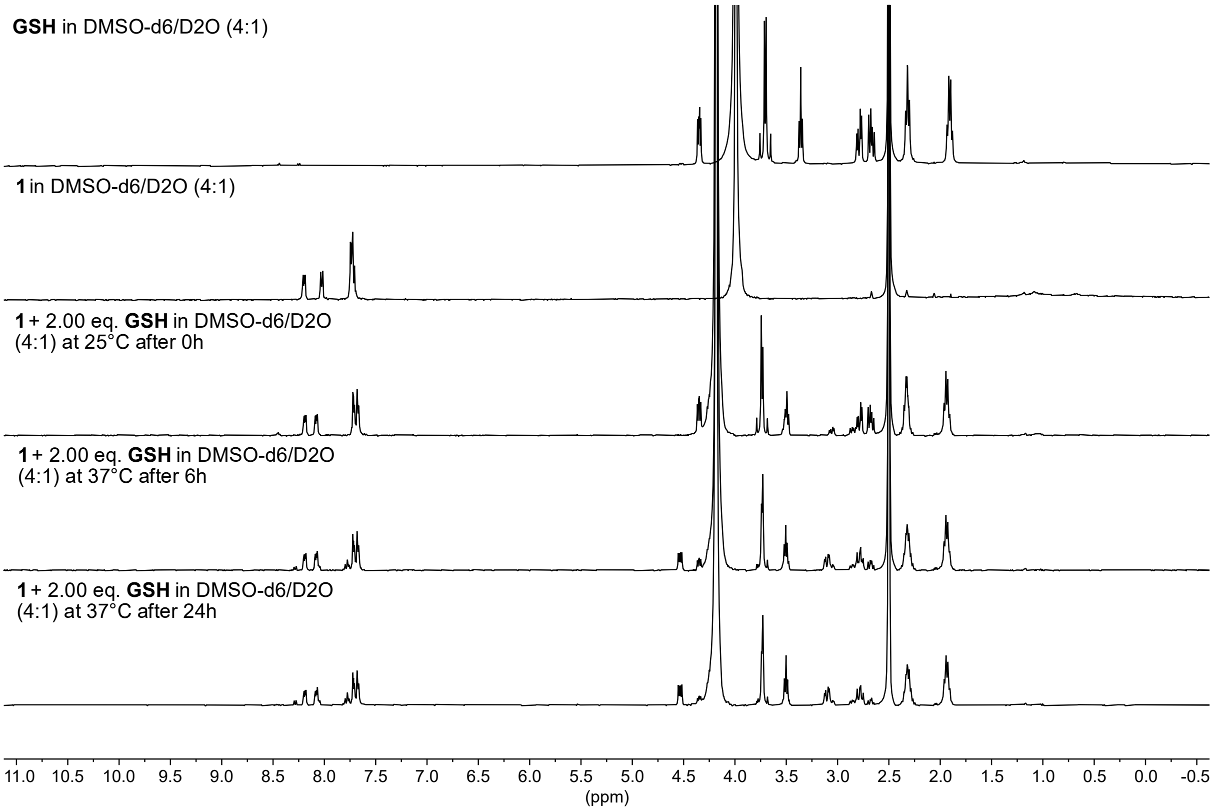

Figure S98: Stability of **1** in DMSO-d_6/D_2O (4:1) after the addition of 2.00 eq. GSH at 37°C at different time points. Spectra of **1** and GSH in DMSO-d_6/D_2O (4:1) are included for reference.

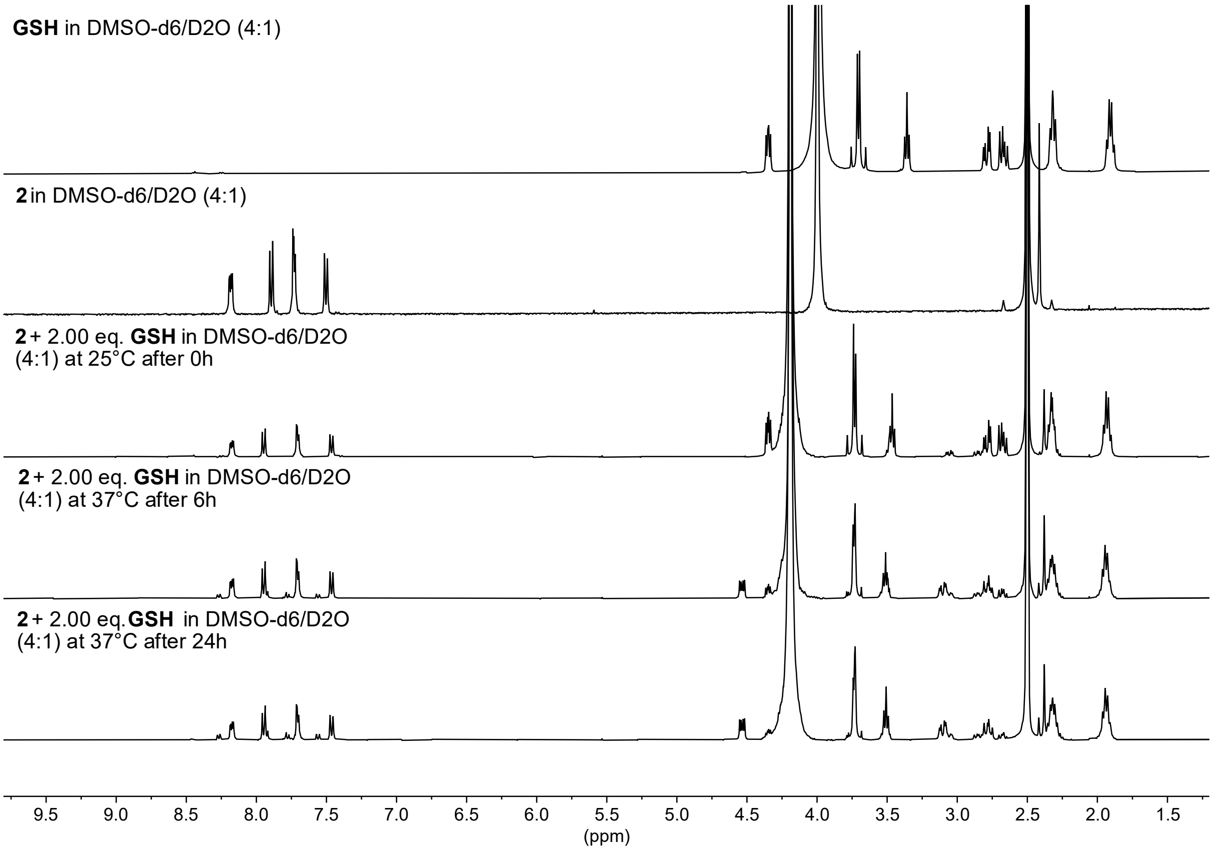

Figure S99: Stability of **2** in DMSO-d_6/D_2O (4:1) after the addition of 2.00 eq. GSH at 37°C at different time points. Spectra of **2** and GSH in DMSO-d_6/D_2O (4:1) are included for reference.

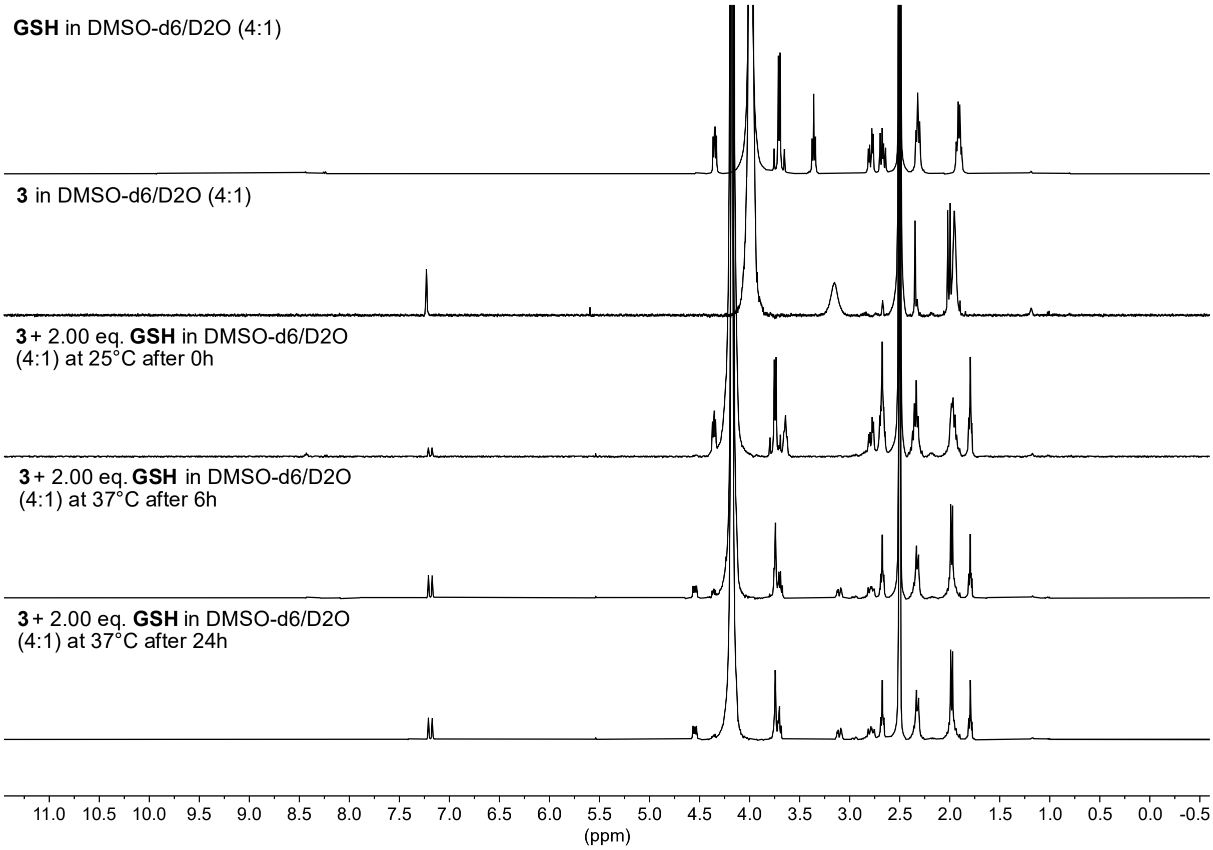

Figure S100: Stability of **3** in DMSO-d_6/D_2O (4:1) after the addition of 2.00 eq. GSH at 37°C at different time points. Spectra of **3** and GSH in DMSO-d_6/D_2O (4:1) are included for reference.

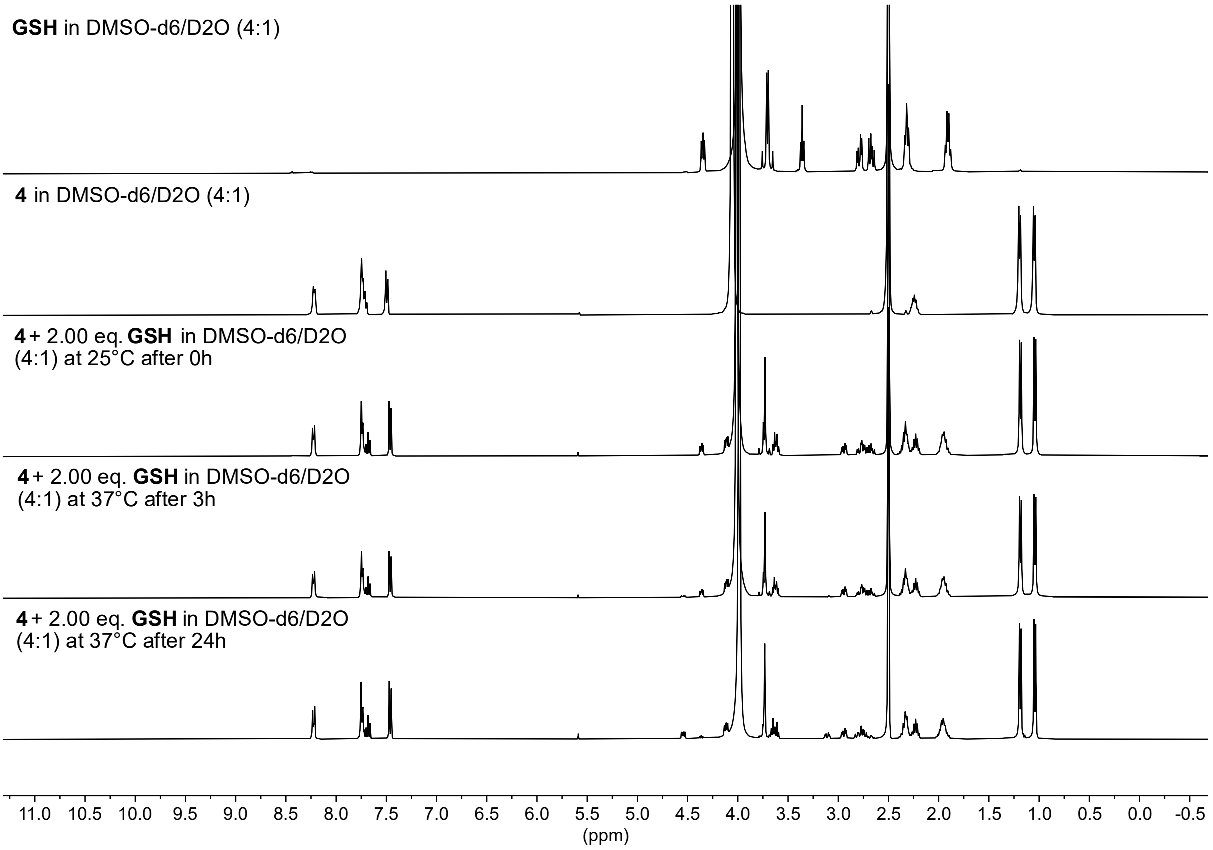

Figure S101: Stability of **4** in DMSO-d_6/D_2O (4:1) after the addition of 2.00 eq. GSH at 37°C at different time points. Spectra of **4** and GSH in DMSO-d_6/D_2O (4:1) are included for reference.

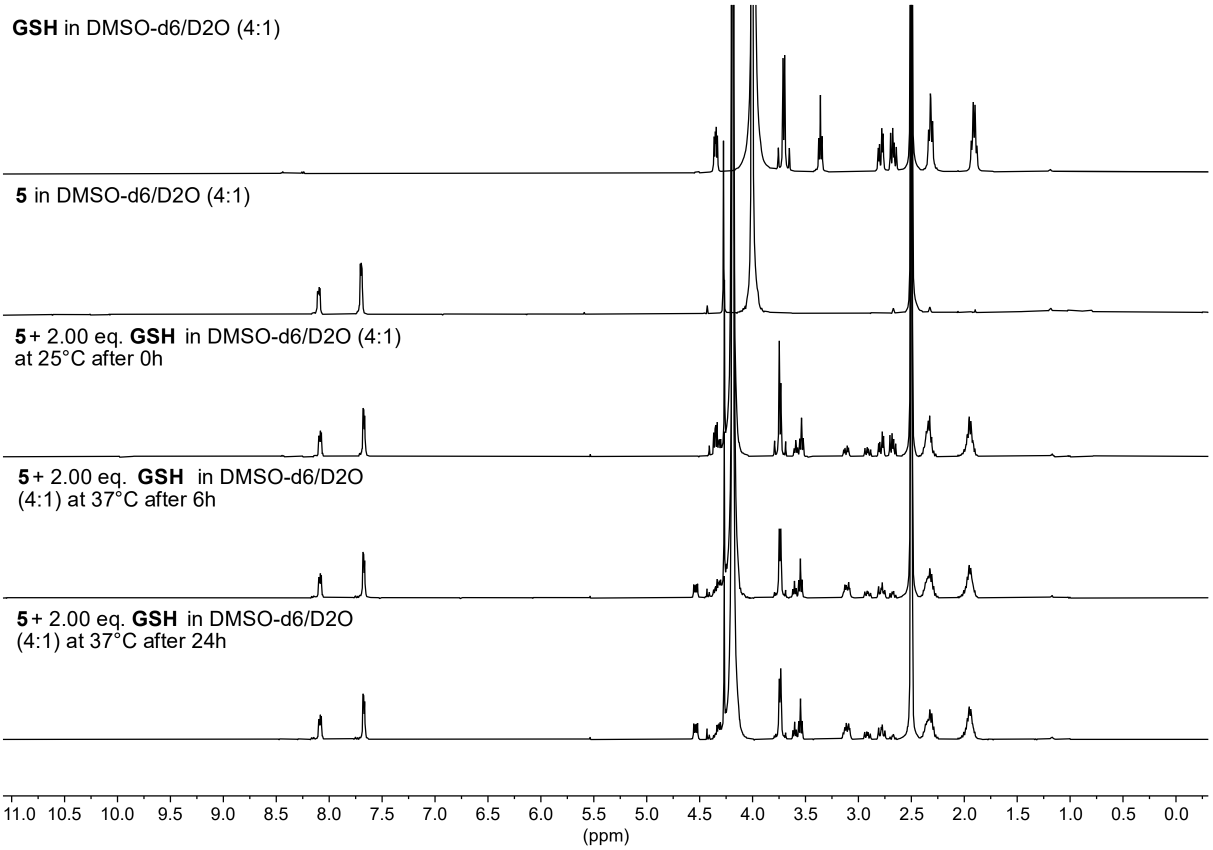

Figure S102: Stability of **5** in DMSO-d_6/D_2O (4:1) after the addition of 2.00 eq. GSH at 37°C at different time points. Spectra of **5** and GSH in DMSO-d_6/D_2O (4:1) are included for reference.

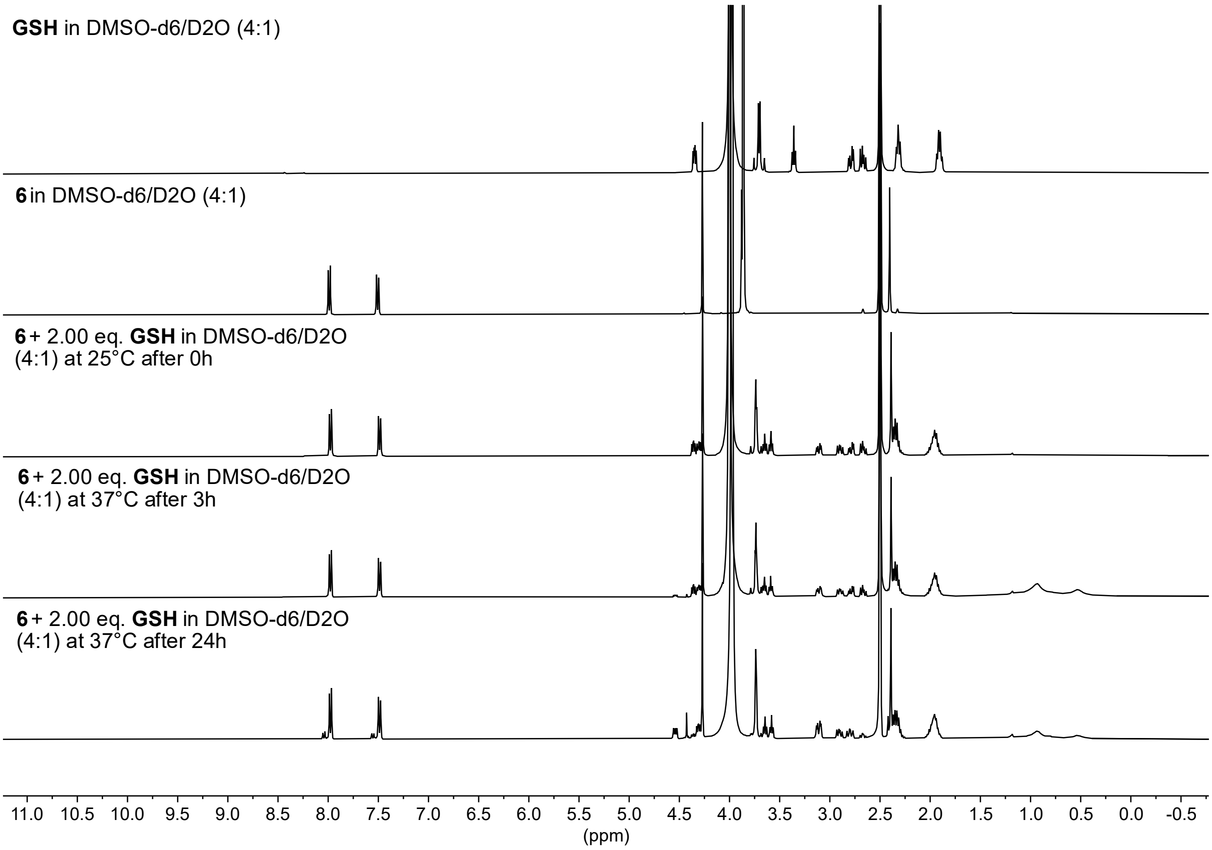

Figure S103: Stability of **6** in DMSO-d_6/D_2O (4:1) after the addition of 2.00 eq. GSH at 37°C at different time points. Spectra of **6** and GSH in DMSO-d_6/D_2O (4:1) are included for reference.

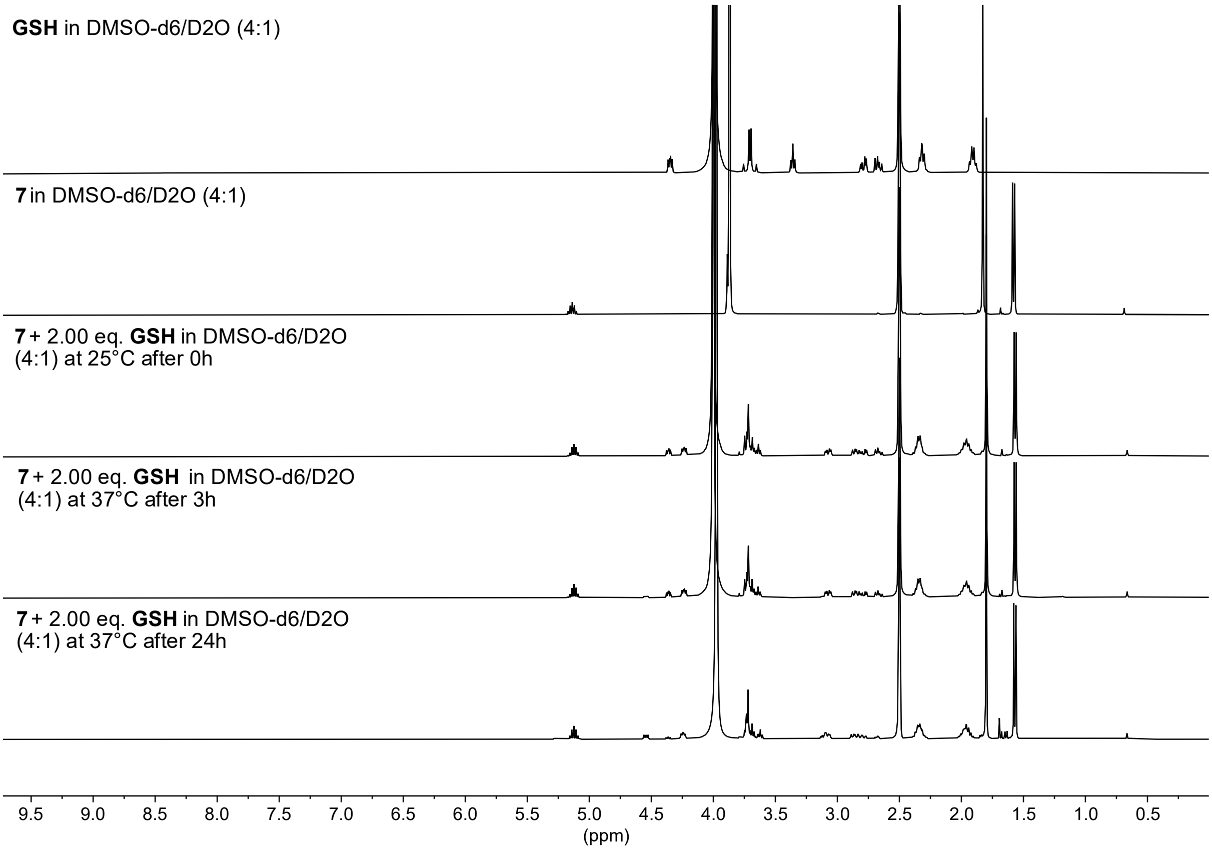

Figure S104: Stability of **7** in DMSO-d_6/D_2O (4:1) after the addition of 2.00 eq. GSH at 37°C at different time points. Spectra of **7** and GSH in DMSO-d_6/D_2O (4:1) are included for reference.

5 Crystallographic Data

SC-XRD Data were collected on a Bruker D8 Venture single crystal X-ray diffractometer equipped with a CMOS detector (Bruker Photon-100), an IMS microfocus source with MoKα radiation (λ = 0.71073 Å) and a Helios optic using the APEX 2/4 software package. Measurements were performed on single crystals coated with perfluorinated ether. The crystals were fixed on top of a Kapton micro sampler and frozen under a stream of cold nitrogen. A matrix scan was used to determine the initial lattice parameters. Reflections were corrected for Lorentz and polarization effects, scan speed, and background using SAINT. Absorption corrections including odd and even ordered spherical harmonics were performed using SADABS. Space group assignments were based upon systematic absences, E statistics, and successful refinement of the structures. The structures were dissolved using SHELXT with the aid of successive difference Fourier maps and were refined against all data using SHELXL in conjunction with SHELXLE. Hydrogen atoms were placed in calculated positions and refined using a riding model, with methylene, aromatic, and other C-H distances of 0.99 Å, 0.95 Å and 1.00 Å, respectively, and U_{iso}(H) = 1.2 Ueq(C). Non-hydrogen atoms were refined with anisotropic displacement parameters. Full-matrix least-squares refinements were performed by minimizing $\sum w({F_c}^2 - {F_o}^2)^2$ with the SHELXL weighting scheme. Neutral atom scattering factors for all atoms and anomalous dispersion corrections for the non-hydrogen atoms were taken from *International Tables for Crystallography.* The unit cell of **2479326** contains four molecules of dichloromethane which were treated as a diffuse contribution to the overall scattering without specific atom positions using the PLATON/SQUEEZE procedure. Images of the crystal structures were generated with Ortep3. Data are provided free of charge by the joint Cambridge Crystallographic Data Centre and *Fachinformationszentrum Karlsruhe* Access Structures service www.ccdc.cam.ac.uk/structures. CCDC: **2479322-2479327**.

Table S1: Crystallographic data for **1**, **2** and **3**.

	**1**	**2**	**3**
CCDC	2479325	2479324	2479323
formula	$C_{13}H_{10}AuClN_4$	$C_{14}H_{12}AuClN_4$	$C_{19}H_{22}AuClN_4$
formula weight [g mol^{-1}]	454.67	468.69	538.82
space group	P 1 21/c 1	P b c a	P 1 21/c 1
a [Å]	16.2014(7)	13.2004(4)	11.3643(6)
b [Å]	5.9953(2)	6.7105(2)	14.3775(7)
c [Å]	13.9742(6)	32.3000(9)	12.4263(6)
α [°]	90	90	90
β [°]	108.839(2)	90	108.202(2)
γ [°]	90	90	90
V [Å^3]	1284.63(9)	2861.18(15)	1928.74(17)
Z	4	8	4
F(000)	848	1760	1040
T [K]	123(2)	123(2)	100(2)
D_{calc} [g cm^{-3}]	2.351	2.176	1.856
μ [mm^{-1}]	11.649	10.464	7.775
R_1 (I>2σ(I))	0.0124	0.0245	0.0130
wR_2 (all data)	0.0273	0.0502	0.0303
$\Delta\rho_{peak}$, $\Delta\rho_{hole}$ [e Å^{-3}]	0.407, -0.460	0.971, -1.238	1.005, -0.472

Table S2: Crystallographic data for **4**, **5** and **6**.

	**4**	**5**	**6**
CCDC	2479327	2479326	2479322
formula	$C_{19}H_{22}AuClN_4$	$C_8H_8AuClN_4O_0$	$C_9H_{10}Ag_0AuClN_4P_0$
formula weight [g mol^{-1}]	538.82	392.60	406.63
space group	P -1	C 1 2/c 1	P 1 21/c 1
a [Å]	9.5762(4)	19.0949(10)	5.9900(2)
b [Å]	10.4423(4)	6.7804(4)	9.5209(3)
c [Å]	11.5979(4)	18.0938(10)	19.5473(7)
α [°]	111.019(2)	90	90
β [°]	102.503(2)	91.624(3)	91.7690(10)
γ [°]	108.278(2)	90	90
V [Å^3]	954.54(7)	2341.7(2)	1114.25(6)
Z	2	8	4
F(000)	520	1440	752
T [K]	123(2)	123(2)	299(2)
D_{calc} [g cm^{-3}]	1.875	2.227	2.424
μ [mm^{-1}]	7.855	12.761	13.414
R_1 (I>2σ(I))	0.0166	0.0182	0.0334
wR_2 (all data)	0.0370	0.0395	0.0858
$\Delta\rho_{peak}$, $\Delta\rho_{hole}$ [e Å^{-3}]	0.564, -0.945	1.192, -0.598	1.512, -1.443

6 Stability Towards Biomolecules and Bis-NHC Formation in Cell Culture Medium

Table S3. EC_{50} values (µM) for A549 cells, determined by MTT assay under different incubation conditions: (i) 72 h continuous exposure; (ii) 48 h preincubation in medium containing FCS followed by 24 h incubation on cells; and (iii) 48 h preincubation in medium without FCS followed by 24 h incubation on cells. (n.d. = not determined; n = 3).

Compound	(i) 72h (+FCS)	(ii) 48h + 24h (+FCS)	(iii) 48h + 24h (-FCS)
**1**	3.34 ± 0.39	12.16 ± 1.59	9.17 ± 1.29
**2**	3.71 ± 0.77	11.01 ± 1.14	6.95 ± 0.90
**B1**	1.27 ± 0.16	n.d.	n.d.
**B2**	0.61 ± 0.09	n.d.	n.d.
